# Synthesis of functionalised isochromans: epoxides as aldehyde surrogates in hexafluoroisopropanol†

**DOI:** 10.1039/d2sc06692k

**Published:** 2023-02-15

**Authors:** Cyprien Muller, Filip Horký, Marie Vayer, Andrei Golushko, David Lebœuf, Joseph Moran

**Affiliations:** a Institut de Science et d’Ingénierie Supramoléculaires (ISIS), CNRS UMR 7006, Université de Strasbourg 8 allée Gaspard Monge 67000 Strasbourg France dleboeuf@unistra.fr moran@unistra.fr

## Abstract

The oxa-Pictet–Spengler reaction is arguably the most straightforward and modular way to construct the privileged isochroman motif, but its scope is largely limited to benzaldehyde derivatives and to electron-rich β-phenylethanols that lack substitution along the aliphatic chain. Here we describe a variant of this reaction starting from an epoxide, rather than an aldehyde, that greatly expands the scope and rate of the reaction (<1 h, 20 °C). Besides facilitating the initial Meinwald rearrangement, the use of hexafluoroisopropanol (HFIP) as a solvent expands the electrophile scope to include partners equivalent to ketones, aliphatic aldehydes, and phenylacetyl aldehydes, and the nucleophile scope to include modestly electronically deactivated and highly substituted β-phenylethanols. The products could be easily further derivatised in the same pot by subsequent ring-opening, reductions, and intra- and intermolecular Friedel–Crafts reactions, also in HFIP. Finally, owing to the high pharmacological relevance of the isochroman motif, the synthesis of drug analogues was demonstrated.

## Abstract 2

The use of epoxides as aldehyde surrogates allows isochromans to be constructed with greater scope than previously reported oxa-Pictet–Spengler reactions. The initial Meinwald rearrangement and subsequent steps are promoted by HFIP and triflic acid.

## Graphical abstract

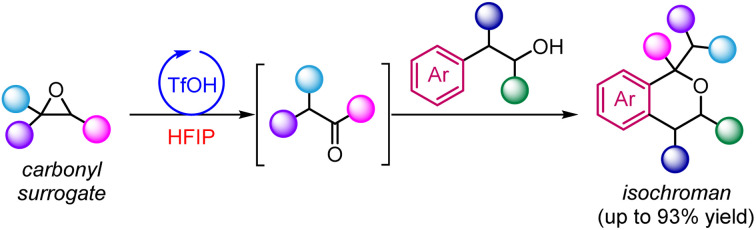

## Introduction

The search for alternative reagents and reaction conditions is key for the development of new processes and the refinement of existing ones.^1^ Beyond efficiency, reducing costs, and improving the practicality of a given methodology have become priorities to broaden its utilization. In this regard, one area of growing interest has been the development of surrogate reagents that can be manipulated without requiring special precautions. This approach, in particular, allows the *in situ* release of otherwise difficult-to-handle, expensive and/or hazardous compounds.^1^

During our recent work on epoxide reduction using hexafluoroisopropanol (HFIP),^2^ we found that the mechanism involved an initial Meinwald rearrangement,^3^ in which epoxides were first converted *in situ* into the corresponding aldehydes before reacting further.^4^ This observation triggered our interest, as aldehydes, particularly the aliphatic ones that are enolizable, are notoriously prone to side-reactions such as self-condensation, which render them unstable and difficult to store in pure form. As a result, some commercially available aldehyde derivatives are expensive and must be freshly purified before use. On the other hand, epoxides are readily accessed from ketones or alkenes,^5^ generally air-stable and store well. Thus, their ability to undergo the Meinwald rearrangement in HFIP makes them an ideal and more versatile surrogate reagent for aldehydes than others reported in the literature.^6^

**Scheme 1:**
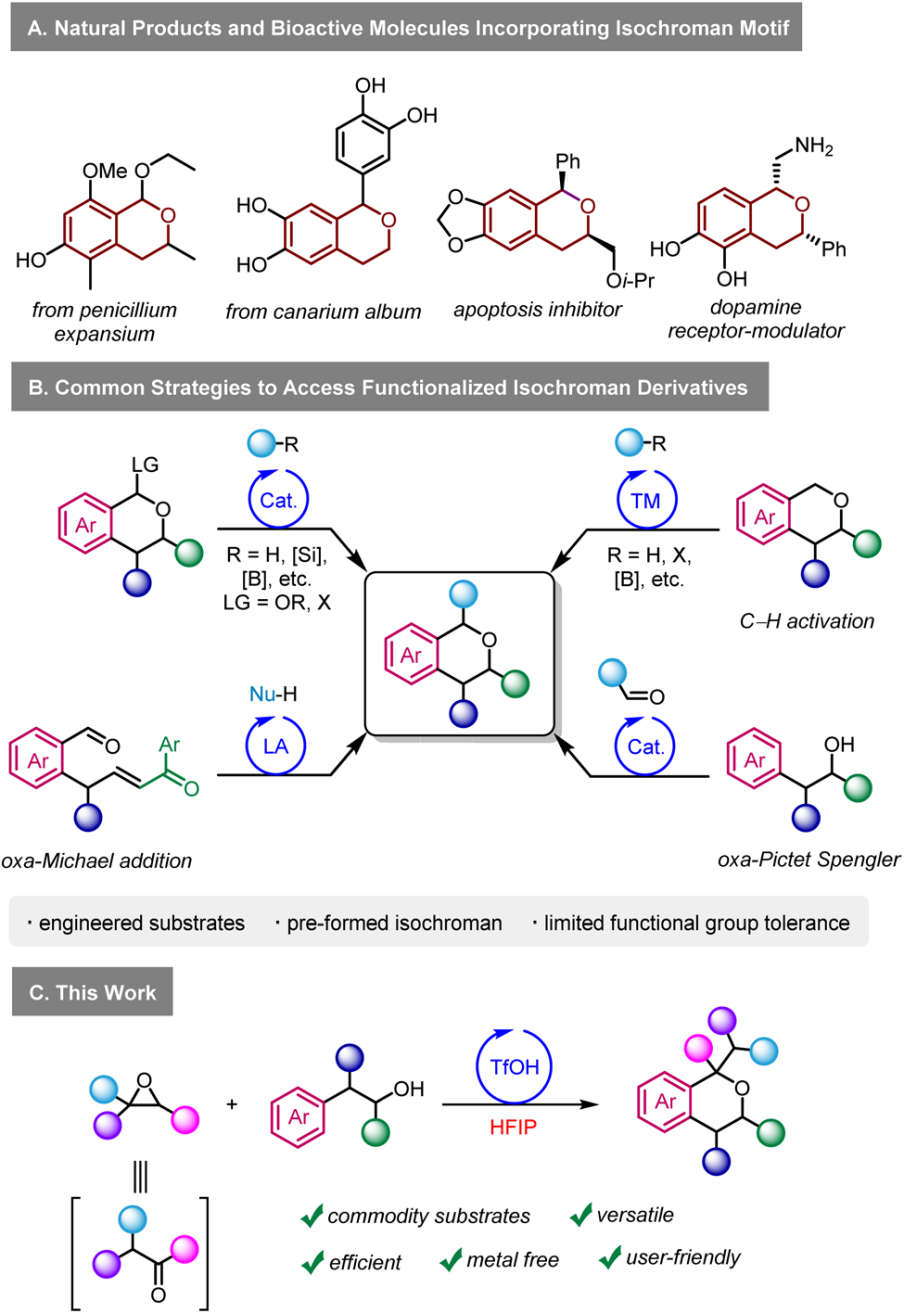
Biological relevance of the isochroman motif and synthetic pathways.

In that context, we became interested in developing a practical methodology to access densely functionalised isochromans using epoxides in HFIP. Isochromans are widespread in nature and in synthetic pharmaceuticals (Scheme 1A).^7^ Various methods exist to access α-functionalised isochroman derivatives (Scheme 1B),^8^ including transition metal catalysed C–H activation,^9^ alkylation/arylation of oxocarbenium cations generated from existing isochromans,^10^ oxa-Michael reactions,^11^ and others.^12^ One of the major limitations of these methods is their versatility, as they require engineered starting materials. Arguably, the oxa-Pictet Spengler reaction starting from aldehydes and alcohols represents the most direct way to prepare isochromans.^7*a*^ This transformation is particularly efficient with benzaldehyde derivatives since they cannot enolize and since they generate relatively stable oxonium intermediates during the reaction mechanism. On the other hand, examples of ketone and aliphatic aldehyde derivatives in the oxa-Pictet Spengler reaction are limited.^13^ Similarly, the use of phenylacetyl aldehydes was scarcely reported,^13*f*,14^ with moderate functional group tolerance, especially regarding electron-withdrawing groups. The limitations of the oxa-Pictet Spengler reaction do not only concern aldehydes but also the alcohol partner, as sporadic examples exist for 2,2-disubstituted and 1,2-disubstituted alcohols.^15^ Furthermore, it is important to stress that high temperatures and long reaction times are often required in those protocols. Re-imagining the oxa-Pictet Spengler as starting from epoxides in HFIP could circumvent these limitations on three fronts. First, reactions involving cationic intermediates often show a much larger scope in HFIP than in traditional solvents.^2*e*,*g*^ Second, HFIP could greatly accelerate the generation of reactive intermediates in the mechanism, even at low temperatures, resulting in much shorter reaction times. Finally, the ability to access enolizable aldehydes *in situ* from bench-stable epoxides represents a highly practical and user-friendly alternative. Such an approach has the potential to complement the traditional scope of the oxa-Pictet–Spengler, which has so far largely been centred around benzaldehydes. Herein, we report our investigations on the reactivity of epoxides with *O*-nucleophiles in HFIP, with the aim of reaching densely functionalised isochroman derivatives (Scheme 1C).

## Results and discussion

### Optimisation studies

We first evaluated the reaction between 2-phenylethan-1-ol and 2-(4-nitrophenyl)oxirane 1a in HFIP in the presence of 10 mol% of trifluoromethanesulfonic acid (TfOH) (Table 1). Based on our previous work on the nucleophilic ring-opening of epoxides,^16^ it was unclear whether the desired Meinwald rearrangement/oxa-Pictet–Spengler pathway could outcompete the direct nucleophilic substitution of the epoxide by an alcohol. Under those conditions, the reaction occurred in 70% yield at room temperature within 1 h to form the target isochroman 2a (entry 1). We therefore proceeded with the optimisation of the reaction. Performing the reaction at higher concentrations (entries 2 and 3) as well as lowering the catalyst loading (entries 4 and 5) or using an alternative Brønsted acid (entry 6) led to lower yields. Of note, the use of Bi(OTf)_3_ which has been previously reported as a practical source to release *in situ* TfOH^17^ was also compatible with our methodology, albeit in a slightly lower yield (entry 7). Increasing the temperature (entry 8) or lowering the equivalents of nucleophile (entry 9) also resulted in lesser efficacies. Importantly, the use of other solvents did not result in the formation of the isochroman but rather in the direct opening of the epoxide by the nucleophile or the solvent (entries 10–13), illustrating the crucial role of HFIP in the transformation. In the case of another fluorinated alcohol such as trifluoroethanol (TFE), we only observed the direct addition of TFE to the epoxide (entry 14). Finally, no reaction occurred in the absence of acid as a control experiment (entry 15).

**Table 1:** Optimisation of the reaction conditions

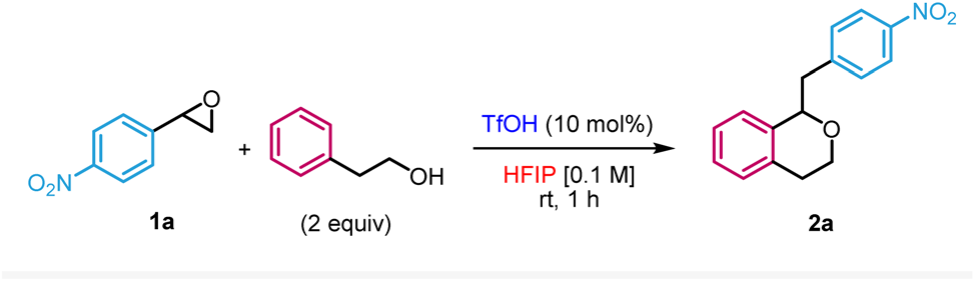		
Entry	Variation from standard conditions^a^	Yield^b^ (%)
**1**	**None**	**70**
2	0.4 M instead of 0.1 M	41
3	0.2 M instead of 0.1 M	46
4	5 mol% of TfOH instead of 10 mol%	49
5	1 mol% of TFOH instead of 10 mol%	16
6	HNTf_2_ instead of TfOH	35
7	Bi(OTf)_3_ instead of TfOH	59
8	80 °C instead of room temperature	52
9	1.1 equiv. of nucleophile instead of 2 equiv.	43
10	CH_2_Cl_2_ instead of HFIP	0^c^
11	MeNO_2_ instead of HFIP	0^c^
12	Toluene instead of HFIP	0^c^
13	HFIP–Me instead of HFIP	0^c^
14	TFE instead of HFIP	0^d^
15	Without TfOH	nr

a Standard reaction conditions: 1a (0.2 mmol), 2-phenylethan-1-ol (2 equiv.), TfOH (10 mol%) in HFIP (0.1 M), rt, 1 h (in a sealed tube).

b Isolated yields.

c Direct ring-opening by nucleophile.

d Direct ring-opening by solvent.

### Scope and limitations

**Scheme 2:**
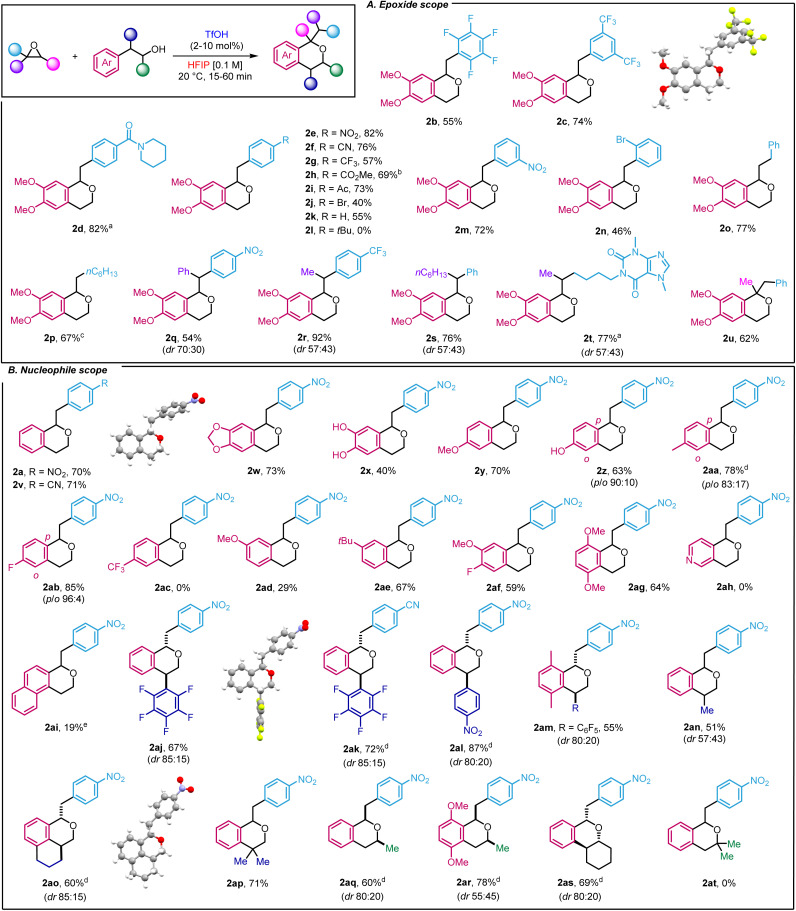
Scope of the reaction. ^*a*^TfOH 40 mol% (increments of 10 mol%). ^*b*^Corrected yield. ^*c*^3 equiv. epoxide. ^*d*^Diastereoisomers were separated. ^*e*^16 h.

Next, we investigated the scope of the transformation by using 2-(3,4-dimethoxyphenyl)ethan-1-ol as a model nucleophile (Scheme 2A). We started by studying the functional group tolerance of our method towards strong electron-withdrawing groups, which had not been described with existing oxa-Pictet–Spengler methods. Satisfyingly, highly electron-deficient styrene oxides such as 2-(perfluorophenyl)oxirane and 2-(3,5-bis(trifluoromethyl)phenyl)oxirane were well-tolerated, affording the products in good yield (2b–c). Moving to *para*-substituted styrene oxides, we found that substrates incorporating amide, nitro, cyano, trifluoromethyl, ester and ketone groups performed well (2d–i). Less electron-deficient substrates such as *p*-Br styrene oxide and simple styrene oxide were also tolerated (2j–k), but *p-t*Bu substitution proved unsuccessful (2l). In that case, we observed, by ^1^H NMR, the direct formation of the product resulting from the ring-opening of the isochroman by water, which could not be isolated. Finally, the reaction was compatible with the introduction of substituents at the *meta* and *ortho* positions, using nitro and bromine as examples (2m–n). Moving away from styrene oxides, alkyl epoxides 2-benzyloxirane and 2-hexyloxirane provided the corresponding compounds in 77 and 67%, respectively (2o–p). In the latter case, an excess of epoxide such phenyl and alkyl substitution did not have any impact on the reactivity (2q–s). To explore drug derivatives under our reaction conditions, we prepared the corresponding epoxide from pentoxyfilline, a drug used against muscle pain. After subjecting it to our conditions, the product was obtained in 77% yield as a mixture of two diastereoisomers (2t). Importantly, 1,2-disubstituted epoxides as surrogates for ketones could also be used to afford 2u in 62% yield.

Our interest then shifted towards the alcohol scope (Scheme 2B). 2-Phenylethanol produced the corresponding isochromans in good yields (2a, 2v). As expected, the presence of electron-donating groups on the phenyl ethanol ring proved effective. Indeed, the methylenedioxy group, often found in bioactive compounds, led to the target product in 73% yield (2w) whereas the unprotected di-phenol also delivered isochroman 2x, albeit in a lower yield (40%). Methoxy, hydroxy and methyl substitution in the *meta* position of the phenyl ethanol ring yielded the products in yields ranging from 63 to 78% along with an excellent *p*-selectivity (2y–2aa). Remarkably, while the oxa-Pictet Spengler reaction has only been described with electron-donating groups, our method was also compatible with the presence of moderate electron-withdrawing group such as fluorine (2ab and 2af, 85 and 59%, respectively). On the other hand, a stronger electron-withdrawing group such as a CF_3_ group was unsuccessful (2ac). We then investigated *p*-substituted phenyl ethanols, starting with methoxy substitution (2ad) and observed a larger difference in yield compared to the *m*-substituted equivalent, highlighting the importance of substituents in this transformation. In this case, *p-t*Bu substitution was compatible with our methodology (2ae). Finally disubstituted 2-(3-fluoro-4-methoxyphenyl)ethan-1-ol and 2-(2,5-dimethoxyphenyl)ethan-1-ol also proved efficient (2af–2ag). Of note, the use of pyridyl and naphthyl ethanols was detrimental to the reaction (2ah–2ai). We next took advantage of our recently described method for the ring-opening arylation of aryl epoxides to use more elaborate nucleophiles.^16^ Thus, 2-(perfluorophenyl)-2-phenylethan-1-ol and 2-(4-nitrophenyl)-2-phenylethan-1-ol derivatives were prepared and subjected to our reaction conditions to yield densely functionalised products in high yields (up to 87%) and drs between 85 : 15 and 80 : 20 (2aj–2am). Using NOESY allowed us to determine the configuration of the major diastereoisomer as *trans* in the case of 2ak (ESI, Section 5†). The reaction was not limited to aryl substituent as nucleophiles bearing alkyl substituents et the same position, led to desired product in 51% yield (2an), albeit with a lower dr. Using (1,2,3,4-tetrahydronaphthalen-1-yl)methanol, more complex structures such as 2ao could be reached in 60% yield (dr 85 : 15). Both diastereoisomers were separated by flash column chromatography and the structure of the major one was identified by X-ray crystallography (2ao, ESI Section 6†). Of note, a dimethyl group at that position also proved efficient which only constitutes the second example of use of 2,2-disubstituted alcohol for isochroman synthesis in an oxa-Pictet Spengler reaction (2ap). Secondary alcohols were also well-tolerated (2aq–as). In those cases, the relative configuration was determined by NOESY (ESI, Section 5†). It thus constitutes one of the rare examples of such reactivity. Unfortunately, tertiary alcohols were not compatible with our methodology as the alcohol decomposed under the reaction conditions (2at).

**Scheme 3:**
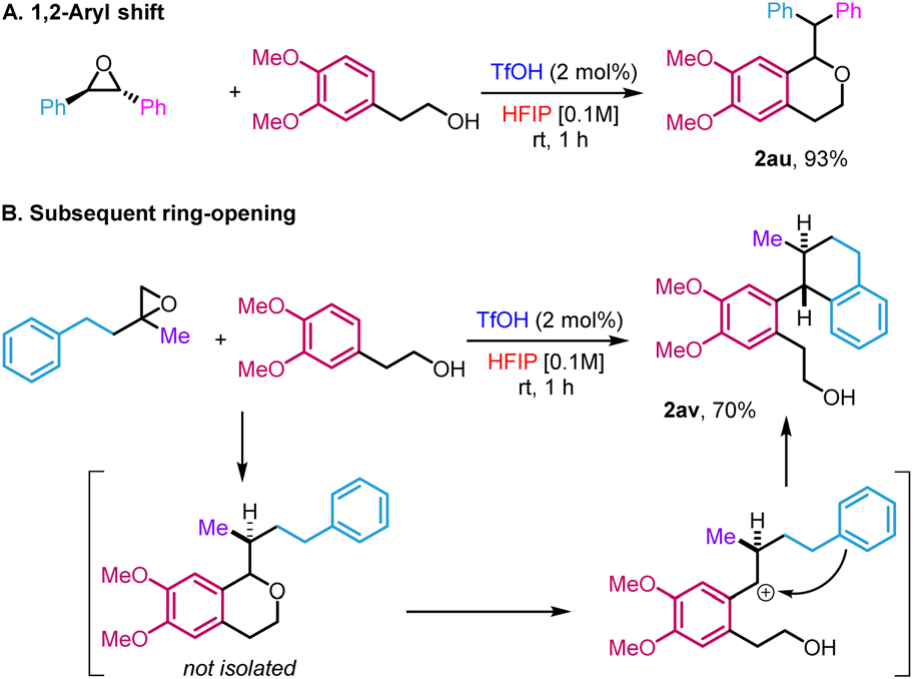
Variations of the standard reaction.

The high reactivity in HFIP allowed us to access new variations of the reactivity that fall outside the scope of the traditional oxa-Pictet–Spengler reaction described above (Scheme 3). In the case of the reaction of *trans*-stilbene oxide with phenyl ethanol derivatives, the isochroman formed arose from an initial 1,2-aryl shift, providing 2au in 93% yield (Scheme 3A). This is not completely surprising as such displacement has already been observed in previous studies on the Meinwald rearrangement.^18^ In the case of 2-methyl-2-phenethyloxirane, the initially formed isochroman unexpectedly ring-opened under the reaction conditions following an intramolecular Friedel–Crafts reaction, leading to primary alcohol 2av in 70% yield (Scheme 3B, *trans* relative configuration ascertained by NOESY, ESI Section 5†).

**Scheme 4:**
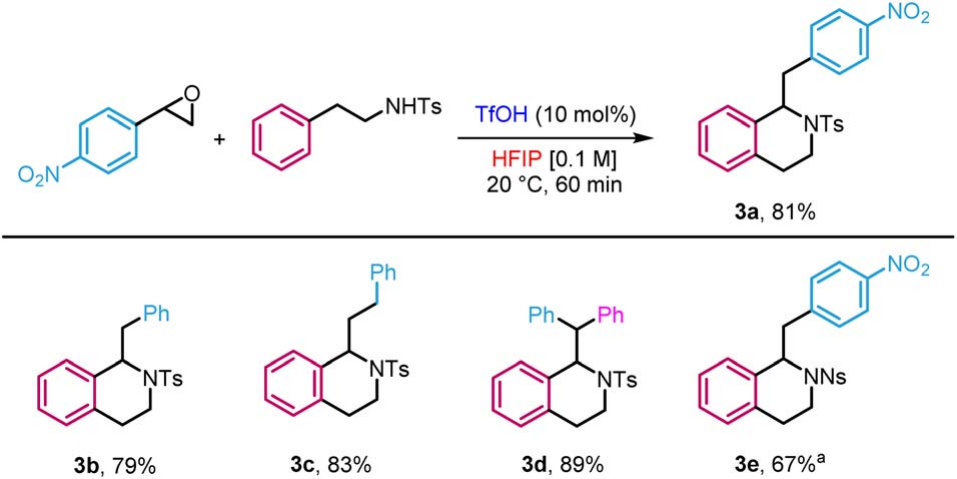
Application to the synthesis of tetrahydroisoquinolines. ^*a*^TfOH (30 mol%), 60 °C, 72 h.

Lastly, we extended the reactivity to dinucleophiles beyond the O and C motif embodied by 2-phenylethanols. For instance, *N*-tosylphenethylamine gave tetrahydroisoquinoline 3a in 81% yield (Scheme 4). The reaction was also applied to a variety of substrates to provide the target products in yields ranging from 79 to 89% (3b–e). However, in the case of the unprotected 2-phenylethylamine, no reaction was observed, likely due to the trapping of the catalyst by a more basic alkylamine.

Diols also reacted to form acetals in high yields (3f–g) as a protection *in situ* of aldehydes (eqn (1)). In the same vein, *N*-tosyl-3-aminopropan-1-ol produced the corresponding hemiaminal ether in 63% yield (3h).1
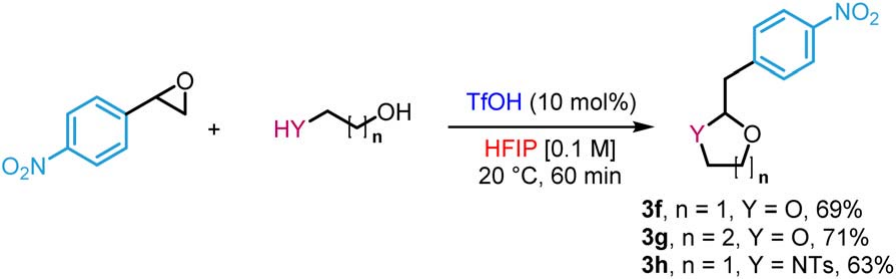


**Scheme 5:**
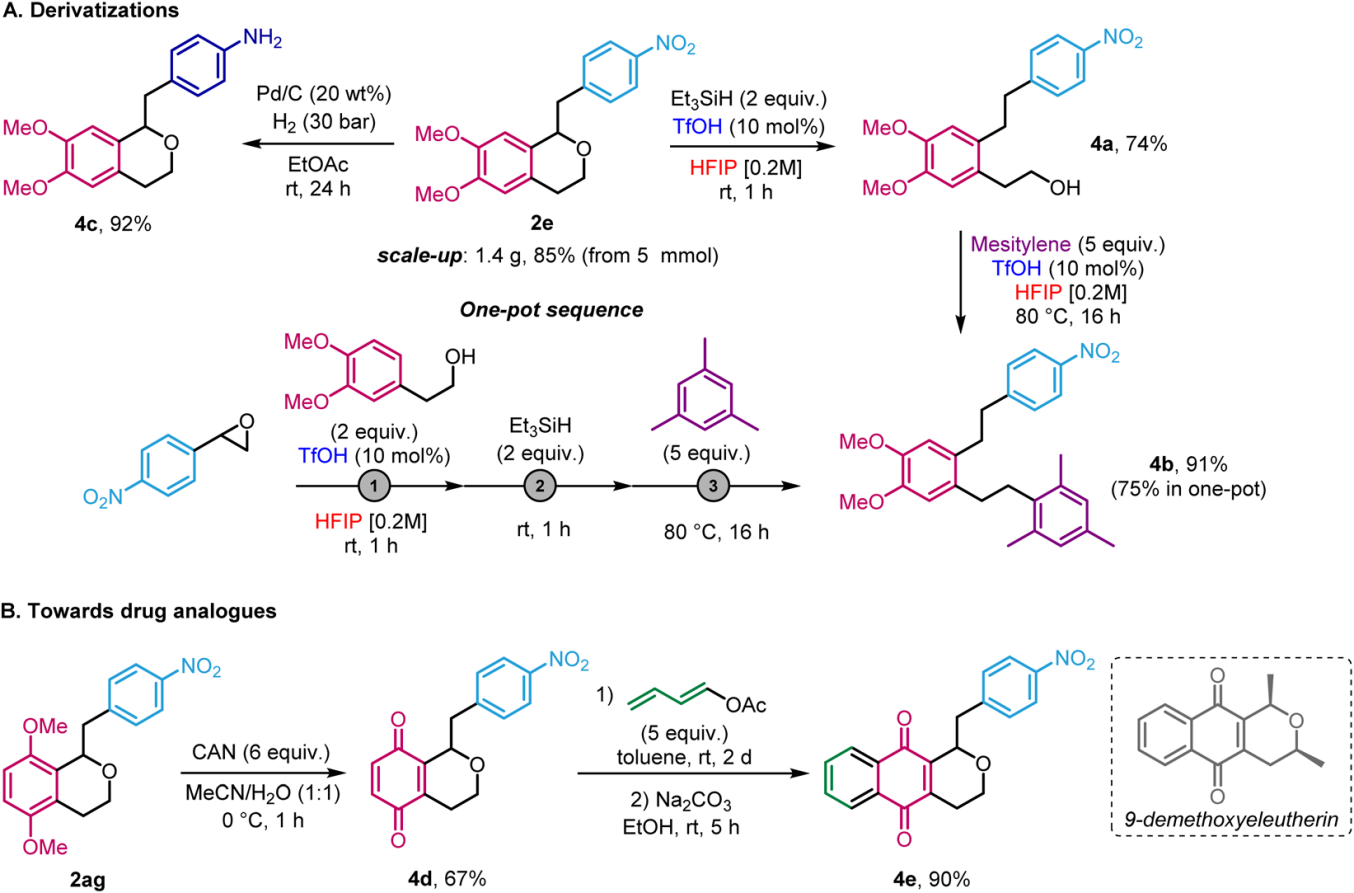
Post-functionalisation of the isochroman products.

To show the utility of our method, isochroman 2e was prepared on large scale (85%, 1.4 g, 4.25 mmol) starting from 5 mmol of 2-(4-nitrophenyl)oxirane 1a (Scheme 5). We then explored post-functionalization pathways. First, driven by the serendipitous formation of 2au and our recent study on the reduction of epoxides in HFIP,^4^ the reductive ring-opening of isochroman 2e ring was achieved, using triethylsilane in HFIP in the presence of TfOH (Scheme 5A). The resulting alcohol 4a was obtained in 74% yield and further reacted with mesitylene to afford Friedel–Crafts product 4b in 91% yield, again using HFIP and TfOH at 80 °C. Gratifyingly, the whole sequence could be achieved in a sequential one-pot to give 4b in 75% yield. This approach enables access to 1,2,4,5-substituted arenes in a regiocontrolled fashion that would be challenging to accomplish by other means. In another application, the limitation that electron-rich epoxides are not compatible with the titled reaction can, at least in the case of nitrostyrene oxide derivatives, be circumvented by recognising that nitro groups can be viewed as masked anilines. Indeed, reduction of was reduced to 2e furnished the corresponding free aniline 4c in 92% yield. Finally, in the guise of synthesising analogues of the bioactive agent 9-demethoxyeleutherin,^19^ compound 2ag was first used to prepare benzoquinone 4d (67%) in the presence of cerium ammonium nitrate (CAN) (Scheme 5b). Then, upon Diels–Alder reaction with buta-1,3-dien-1-yl acetate and elimination,^15*b*^ pyranonaphthoquinone 4e was obtained in 90% yield.

## Conclusions

In conclusion, we have developed the first method to access isochromans starting from epoxides. Through the Meinwald rearrangement, empowered by HFIP, these epoxides generate otherwise unstable aldehydes *in situ* which readily reacted with phenyl ethanols in an oxa-Pictet Spengler reaction in less than one hour at room temperature. The use of epoxides as aldehyde surrogate provided unprecedented functional group diversity, notably the inclusion of ketones, aliphatic aldehydes, and phenyl acetyl aldehydes as electrophiles and the inclusion of 2,2-disubstituted and 1,1-disubstituted alcohols as nucleophiles. The resulting scope allows all positions of the isochroman scaffold to be functionalised, often with good drs, and allows a variety of dinucleophiles to be used. In select cases, serendipitous further reactivity of the isochroman products was observed, inspiring us to explore divergent derivatisation, including sequential one-pot functionalisation. Finally, due to the high biological relevance of isochromans, our method allows to envision the facile synthesis of drug analogues.

## Data availability

All experimental procedures, characterisation data, mechanistic investigations and NMR spectra for all new compounds can be found in the ESI.†

## Author contributions

Conceptualization, D. L. and J. M.; methodology, D. L. and J. M.; investigation, C. M., F. H., M. V. and A. G.; writing – original draft, C. M.; writing – review & editing, D. L. and J. M.; funding acquisition, D. L. and J. M.; supervision, D. L. and J. M.

## Conflicts of interest

There are no conflicts to declare.

## Supplementary Material

SC-014-D2SC06692K-s001

SC-014-D2SC06692K-s002
