# Supplementary material for: Synthesis of functionalised isochromans: epoxides as aldehyde surrogates in hexafluoroisopropanol
Source: Chem Sci. 2023 Feb 15;14(11):2983–9. doi: 10.1039/d2sc06692k (PMC10016621; doi:10.1039/d2sc06692k)
Supplement: SC-014-D2SC06692K-s001 [file SC-014-D2SC06692K-s001.pdf]

Electronic Supplementary Material (ESI) for Chemical Science.
This journal is © The Royal Society of Chemistry 2023

Synthesis of Functionalised Isochromans: Epoxides as Aldehyde Surrogates in HFIP

Supporting Information

Cyprien Muller, Filip Horký, Marie Vayer, Andrei Golushko, David Lebœuf,* Joseph Moran*

Université de Strasbourg, CNRS, ISIS UMR 7006, Strasbourg, 67000, France

moran@unistra.fr

dleboeuf@unistra.fr

Table of contents

Supplemental Experimental Procedures 2
1. General Remarks 2
2. Procedures for the Synthesis of Functionalized Isochromans 3
   2.1 Optimization of Reaction Parameters 3
   2.2 General Procedure 4
   2.3 Characterization Data 5
3. Procedures and Characterization of Post-Functionalization Products 39
4. NMR Spectra 43
5. NOESY Analyses 106
6. XRD 113

Supplemental Experimental Procedures

1. General Remarks

All reagents were used as received from commercial suppliers (*Alfa Aesar*, *Sigma Aldrich, abcr, TCI* or *FluoroChem*) unless otherwise stated. Triflic acid (TfOH) *ReagentPlus*®, ≥99% (CAS: 1493-13-6) was purchased from Sigma Aldrich, and HFIP (CAS: 920-66-1) from FluoroChem. Reaction progress was monitored by thin layer chromatography (TLC) performed on aluminum plates coated with silica gel F_{254} with 0.2 mm thickness. Chromatograms were visualized by fluorescence quenching with UV light at 254 nm and/or by staining using vanilin. Flash column chromatography (FC) was performed using silica gel 60 (230-400 mesh, Merck and co.). Yields refer to chromatographically and spectroscopically pure compounds. When stated, NMR yields were calculated by using mesitylene or 1,3,5-trimethoxybenzene as an internal standard.

^{1}H NMR, ^{13}C NMR and ^{19}F NMR spectra were recorded using a Bruker UltraShield 400, 500 at 300K. ^{1}H NMR chemical shifts are reported in ppm using residual solvent peak as reference ($CDCl_3$: δ = 7.26 ppm, CD_2Cl_2: δ = 5.32 ppm, or acetone-d_6: δ = 2.09 ppm). Data for ^{1}H NMR are presented as follows: chemical shift δ (ppm), multiplicity (s = singlet, d = doublet, t = triplet, m = multiplet, br = broad), coupling constant J (Hz) and integration; ^{13}C NMR spectra were recorded at 100, 125 or using broadband proton decoupling and chemical shifts are reported in ppm using residual solvent peaks as reference ($CDCl_3$: δ = 77.16 ppm, CD_2Cl_2 : δ = 53.84 ppm, or acetone-d_6: δ = 30.60 ppm). Multiplicity was defined by recording a ^{13}C NMR spectra using the attached proton test (APT). ^{19}F NMR spectra were recorded at 376.5 or 471 MHz at ambient temperature. High-resolution mass spectrometry (HRMS) analysis was performed on instruments GCT 1er Waters (EI and IC), MicroTOF-Q Bruker (ESI) and a GC Thermo Scientific Trace 1300 GC unit coupled to an APPI MasCom source mounted on a Thermo Scientific Exactive Plus EMR mass unit (Orbitrap FT-HRMS analyzer).

Melting points were measured using a Melting Point Apparatus SMP10 from Stuart.

2. Procedures for the Synthesis of Functionalized Isochromans

2.1 Optimization of Reaction Parameters

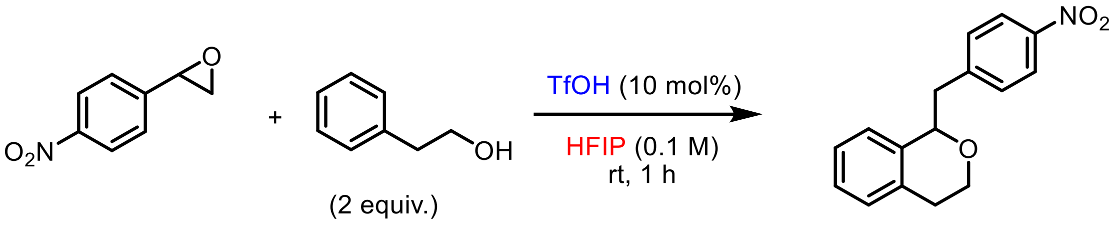

2a

Table S1. Concentration and catalyst loading screening for isochroman synthesis

Entry	Deviation from Standard	Yield [%]
1	none	70
2	[0.2 M]	46
3	[0.4 M]	41
4	[0.6 M]	25
5	TfOH (5 mol%)	47
6	TfOH (1 mol%)	48

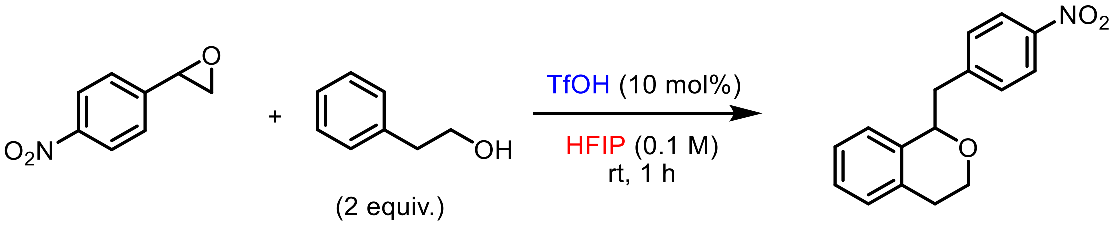

2a

Table S2. Optimization of isochroman synthesis at 0.1 M

Entry	Deviation from Standard	Yield [%]
1	none	70
2	$HNTf_2$	35
3	$Bi(OTf)_3$	59
4	$Sc(OTf)_3$	17
5	$Bi(OTf)_3$/$n$$Bu_4NPF_6$	60
6	$Ca(NTf_2)_2$/$n$$Bu_4NPF_6$	32
7	$B(C_6F_5)_3$	-

8	Molecular sieves	54
9	80 °C	52
10	1.1 equiv	43

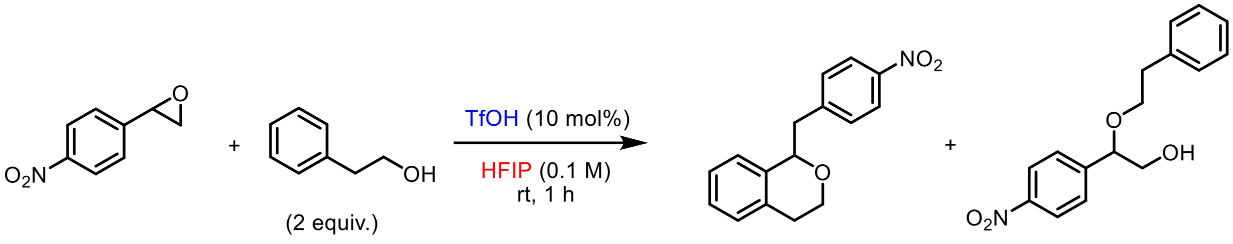

2a 2'a

Table S3. Solvent screening for isochroman synthesis

Entry	Deviation from Standard	NMR Yield [%] 2a/2'a
1	none	70[a]/0
2	$MeNO_2$	0/20
3	Toluene	0/39
4	DCM	0/46
5	TFE	0/-[b]
6	HFIP-Me	0/36

[a] Isolated yield. [b] product of addition of TFE obtained:

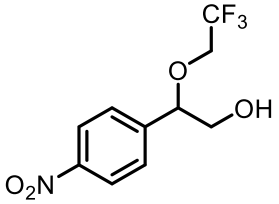

2.2 General Procedure

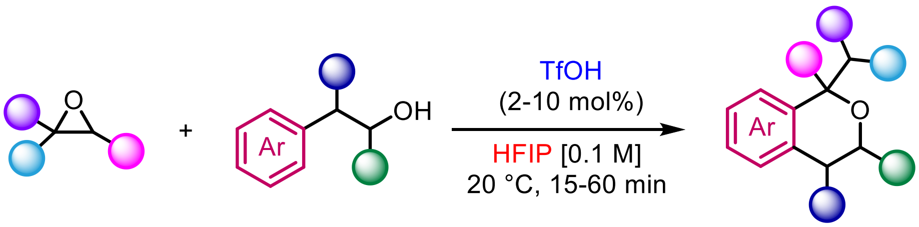

Epoxide (1.0 equiv.) and nucleophile (2.0 equiv.) were charged (in air) in a 10 mL screw-cap tube equipped with a Teflon-coated magnetic stir bar. HFIP (0.1 M) and TfOH (1.0 – 20.0 mol%) were

added (addition of TfOH at 0 °C), and the glass tube was sealed. The reaction mixture was stirred at 25 °C for the indicated time (0.25–1 h). Upon completion, the reaction mixture was quenched with a saturated solution of $NaHCO_3$ (10 mL) and extracted with EtOAc (10 mL × 3). The combined organic layers were washed with brine (10 mL), dried over Na_2SO_4, filtered, and concentrated under reduced pressure. The crude reaction mixture was purified by FC over silica gel to furnish the target products **2**.

2.3 Characterization Data

1-(4-Nitrobenzyl)isochromane **(2a)**

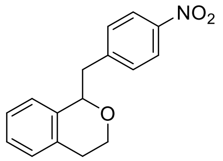

Chemical Formula: $C_{16}H_{15}NO_3$
Exact Mass: 269.11

The general procedure was followed with 2-(4-nitrophenyl)oxirane (33.0 mg, 0.2 mmol) and phenylethanol (48 µL, 0.4 mmol) in the presence of TfOH (1.8 µL, 0.020 mmol, 10 mol%) in HFIP (2.0 mL). The reaction mixture was stirred at 25 °C for 1 h. Purification by FC over silica gel (*n*-pentane/EtOAc 100:0 to 90:10 gradient) afforded **2a** (37.8 mg, 70% yield) as a yellow solid.

m.p. = 122 – 124 °C

^{1}H NMR (400 MHz, $CDCl_3$) δ 8.12 (d, *J* = 8.8 Hz, 2H), 7.41 (d, *J* = 8.8 Hz, 2H), 7.25 – 7.09 (m, 4H), 5.08 (dd, *J* = 8.4, 3.6 Hz, 1H), 4.12 (ddd, *J* = 11.2, 5.3, 3.6 Hz, 1H), 3.74 (ddd, *J* = 11.2, 9.5, 3.6 Hz, 1H), 3.36 (dd, *J* = 14.4, 3.6 Hz, 1H), 3.16 (dd, *J* = 14.4, 8.4 Hz, 1H), 2.88 (ddd, *J* = 14.9, 9.5, 5.3 Hz, 1H), 2.66 (ddd, *J* = 14.9, 3.6, 3.6 Hz, 1H).

^{13}C NMR (126 MHz, $CDCl_3$) δ 146.7, 146.7, 136.9, 134.4, 130.6 (2C), 129.2, 126.8, 126.3, 124.9, 123.4 (2C), 76.1, 63.4, 42.3, 29.1.

HRMS (ESI): m/z calcd. for $C_{16}H_{16}O_3N$ $[M+H]^+$ 270.1027, found 270.1114.

Gram-scale synthesis: 2-(4-nitrophenyl)oxirane (825.0 mg, 5.0 mmol) and 2-(3,4-dimethoxyphenyl)ethanol (1.83 g, 10.0 mmol) were charged (in air) in a 100 mL round-bottom flask equipped with a Teflon-coated magnetic stir bar. HFIP (50 mL, 0.1 M) and TfOH (44.0 µL, 0.500 mmol, 10.0 mol%) were added (addition of TfOH at 0 °C), and the flask was sealed. The reaction mixture was stirred at 25 °C for 1 h. Upon completion, the reaction mixture was quenched with a saturated solution of $NaHCO_3$ (50 mL) and extracted with EtOAc (50 mL × 3). The combined organic layers were washed with brine (50 mL), dried over Na_2SO_4, filtered, and concentrated under reduced pressure. The crude reaction mixture was purified by FC over silica gel (*n*-pentane/EtOAc, 95:5 to 80:20 gradient) to furnish **2e** (1.40 g, 85% yield) as a yellow solid.

***6,7-Dimethoxy-1-((perfluorophenyl)methyl)isochromane* (2b)**

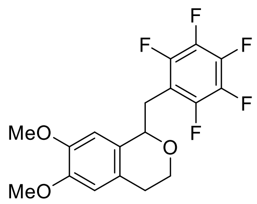

Chemical Formula: $C_{18}H_{15}F_5O_3$
Exact Mass: 374.09

The general procedure was followed with 2-(perfluorophenyl)oxirane (42.0 mg, 0.2 mmol) and 2-(3,4-dimethoxyphenyl)ethan-1-ol (73.0 mg, 0.4 mmol) in the presence of TfOH (1.8 µL, 0.020 mmol, 10 mol%) in HFIP (2.0 mL). The reaction mixture was stirred at 25 °C for 1 h. Purification by FC over silica gel (*n*-pentane/EtOAc 100:0 to 90:10 gradient) afforded **2b** (41.0 mg, 55% yield) as a colorless oil.

^{1}H NMR (400 MHz, CDCl$_3$): δ 6.65 (s, 2H), 4.91 (dd, *J* = 9.7, 4.0 Hz, 1H), 4.06 (ddd, *J* = 11.3, 6.2, 4.7 Hz, 1H), 3.91 (s, 3H), 3.90 (s, 3H), 3.74 (ddd, *J* = 11.3, 6.6, 4.6 Hz, 1H), 3.23 – 3.08 (m, 2H), 2.82 – 2.66 (m, 2H)

^{13}C NMR (126 MHz, CDCl$_3$): δ 148.3, 147.8, 145.7 (dm, *J* = 245.9 Hz, 2C), 140.1 (dm, *J* = 251.9 Hz), 137.6 (dm, *J* = 250.6 Hz, 2C), 128.5, 126.3, 112.5 (td, *J* = 18.8, 3.8 Hz), 111.7, 108.2, 74.2, 61.9, 56.3, 56.1, 29.6 (d, *J* = 1.4 Hz), 28.5.

^{19}F NMR (471 MHz, CDCl$_3$): δ -142.9 (dd, *J* = 22.6, 8.4 Hz), -157.2 (t, *J* = 20.8 Hz), -163.0 (td, *J* = 22.2, 8.0 Hz).

HRMS (ESI): m/z calcd. for $C_{18}H_{16}O_3F_5$ $[M+H]^+$ 375.1014, found 375.1004.

***1-(3,5-Bis(trifluoromethyl)benzyl)-6,7-dimethoxyisochromane* (2c)**

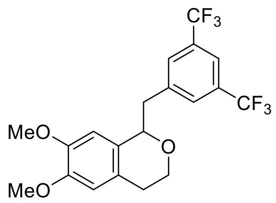

Chemical Formula: $C_{20}H_{18}F_6O_3$
Exact Mass: 420.12

The general procedure was followed with 2-(3,5-bis(trifluoromethyl)phenyl)oxirane (51.2 mg, 0.2 mmol) and 2-(3,4-dimethoxyphenyl)ethan-1-ol (73.0 mg, 0.4 mmol) in the presence of TfOH (1.8 µL, 0.020 mmol, 10 mol%) in HFIP (2.0 mL). The reaction mixture was stirred at 25 °C for 1 h. Purification by FC over silica gel (*n*-pentane/EtOAc 100:0 to 85:15 gradient) afforded **2c** (62.0 mg, 74% yield) as a white solid.

m.p. = 143 – 145 °C

^{1}H NMR (400 MHz, CDCl$_3$): δ 7.71 (m, *J* = 4.5 Hz, 3H), 6.59 (s, 2H), 4.99 (dd, *J* = 8.3, 3.5 Hz, 1H), 4.11 (ddd, *J* = 11.2, 5.7, 3.7 Hz, 1H), 3.86 (s, 3H), 3.86 (s, 3H), 3.70 (ddd, *J* = 11.2, 9.5, 3.7 Hz, 1H), 3.32 (dd, *J* = 14.4, 3.5 Hz, 1H), 3.13 (dd, *J* = 14.4, 8.3 Hz, 1H), 2.77 (ddd, *J* = 15.5, 9.5, 5.7 Hz, 1H), 2.56 (ddd, *J* = 15.5, 3.7, 3.7 Hz, 1H).

^{13}C NMR (126 MHz, CDCl$_3$): δ 148.0, 147.7, 141.0, 131.1 (q, *J* = 33.0 Hz, 2C), 129.9 (m), 128.3, 126.7, 123.5 (q, *J* = 272.6 Hz, 2C), 120.3 (p, *J* = 3.8 Hz, 2C), 111.7, 107.9, 75.5, 63.3, 56.1, 55.9, 42.2, 28.5.

^{19}F NMR (471 MHz, CDCl$_3$): δ -62.8.

HRMS (ESI): m/z calcd. for $C_{20}H_{19}O_3F_6$ [M+H] + 421.1233, found 421.1221.

(4-((6,7-Dimethoxyisochroman-1-yl)methyl)phenyl)(piperidin-1-yl)methanone **(2d)**

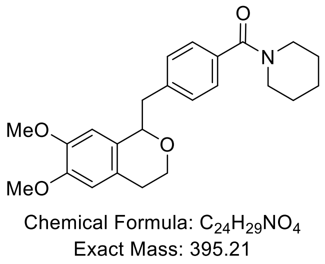

The general procedure was followed with 4-(oxiran-2-yl)phenyl(piperidin-1-yl)methanone (46.0 mg, 0.20 mmol) and 2-(3,4-dimethoxyphenyl)ethan-1-ol (73.0 mg, 0.4 mmol) in the presence of TfOH (1.8 µL, 0.020 mmol, 10 mol%) in HFIP (2.0 mL). The reaction mixture was stirred at 25 °C for 10 min and then TfOH (1.8 µL, 0.020 mmol, 10 mol%) was added. The operation was repeated twice for a reaction time of 1 h. Purification by FC over silica gel (*n*-pentane/EtOAc 80:20 to 35:65 gradient) afforded **2d** (64.0 mg, 82% yield) as a colorless oil.

^{1}H NMR (400 MHz, CDCl$_3$): δ 7.33 – 7.27 (m, 4H), 6.58 (s, 1H), 6.52 (s, 1H), 4.97 (dd, *J* = 8.2, 4.2 Hz, 1H), 4.10 (ddd, *J* = 11.2, 5.1, 4.2 Hz, 1H), 3.85 (s, 3H), 3.80 (s, 3H), 3.73 (ddd, *J* = 11.2, 8.7, 4.2 Hz, 1H), 3.70 (brs, 2H), 3.37 (brs, 2H), 3.18 (dd, *J* = 14.3, 4.2 Hz, 1H), 3.07 (dd, *J* = 14.3, 8.2 Hz, 1H), 2.81 (ddd, *J* = 15.9, 8.7, 5.1 Hz, 1H), 2.60 (ddd, *J* = 15.9, 4.2, 4.2 Hz, 1H), 1.72 – 1.44 (m, 6H).

^{13}C NMR (100 MHz, CDCl$_3$): 170.5, 147.8, 147.4, 140.4, 134.4, 129.6 (2C), 129.4, 127.0 (2C), 126.4, 111.7, 108.3, 76.2, 63.0, 56.1, 56.0, 48.9, 43.3, 42.6, 28.7, 26.6, 25.8, 24.7.

HRMS (ESI): *m/z* calcd. For $C_{24}H_{30}NO_4$ $[M+H]^+$ 396.2169, found 396.2161.

***6,7-Dimethoxy-1-(4-nitrobenzyl)isochromane* (2e)**

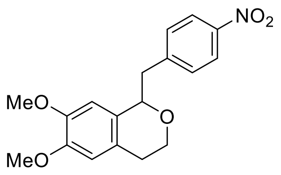

Chemical Formula: $C_{18}H_{19}NO_5$
Exact Mass: 329.13

The general procedure was followed with 2-(4-nitrophenyl)oxirane (33.0 mg, 0.2 mmol) and 2-(3,4-dimethoxyphenyl)ethanol (73.0 mg, 0.4 mmol) in the presence of TfOH (1.8 µL, 0.020 mmol, 10 mol%) in HFIP (2.0 mL). The reaction mixture was stirred at 25 °C for 0.25 h. Purification by FC over silica gel (*n*-pentane/EtOAc 95:5 to 80:20 gradient) afforded **2e** (54.0 mg, 82% yield) as a yellow oil.

^{1}H NMR (400 MHz, CDCl$_3$): δ 8.11 (d, *J* = 8.7 Hz, 2H), 7.40 (d, *J* = 8.7 Hz, 2H), 6.58 (s, 1H), 6.59 (s, 1H), 5.01 (dd, *J* = 8.3, 3.7 Hz, 1H), 4.08 (ddd, *J* = 11.1, 5.2, 3.8 Hz, 1H), 3.85 (br s, 6H), 3.70 (ddd, *J* = 11.1, 9.5, 3.8 1H), 3.30 (dd, *J* = 14.3, 3.7 Hz, 1H), 3.13 (dd, *J* = 14.3, 8.3 Hz, 1H), 2.78 (ddd, *J* = 15.1, 9.5, 5.2 Hz, 1H), 2.56 (ddd, *J* = 15.1, 3.8, 3.8 Hz, 1H).

^{13}C NMR (100 MHz, CDCl$_3$): δ 148.0, 147.6, 146.7, 146.7, 130.6 (2C), 128.6, 126.7, 123.4 (2C), 111.7, 108.0, 75.7, 63.4, 56.2, 56.0, 42.5, 28.6.

HRMS (ESI): *m/z* calcd. For $C_{18}H_{20}NO_5$ $[M+H]^+$ 330.1336, found 330.1329.

***4-((6,7-Dimethoxyisochroman-1-yl)methyl)benzonitrile* (2f)**

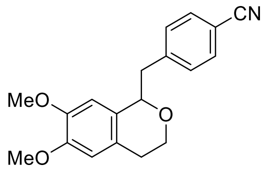

Chemical Formula: $C_{19}H_{19}NO_3$
Exact Mass: 309.14

The general procedure was followed with 2-(4-cyanophenyl)oxirane (29.0 mg, 0.20 mmol) and 2-(3,4-dimethoxyphenyl)ethanol (73.0 mg, 0.4 mmol) in the presence of TfOH (1.8 µL, 0.020 mmol, 10 mol%) in HFIP (2.0 mL). The reaction mixture was stirred at 25 °C for 1 h. Purification by FC over silica gel (*n*-pentane/EtOAc 90:10 to 80:20 gradient) afforded **2f** (47.0 mg, 76% yield) as a colorless oil.

^{1}H NMR (400 MHz, CDCl$_3$): δ 7.55 (d, *J* = 8.3 Hz, 2H), 7.35 (d, *J* = 8.3 Hz, 2H), 6.58 (s, 1H), 6.56 (s, 1H), 4.98 (dd, *J* = 8.3, 3.8 Hz, 1H), 4.08 (ddd, *J* = 11.2, 5.2, 3.8 Hz, 1H), 3.86 (s, 3H), 3.84 (s, 3H), 3.70 (ddd, *J* = 11.2, 9.3, 3.8 Hz, 1H), 3.25 (dd, *J* =14.3, 3.8 Hz, 1H), 3.08 (dd, *J* = 14.3, 8.3 Hz, 1H), 2.77 (ddd, *J* = 15.9, 9.3, 5.2, 1H), 2.56 (ddd, *J* = 15.9, 3.8, 3.8 Hz, 1H).

^{13}C NMR (100 MHz, CDCl$_3$): δ 148.0, 147.6, 144.5, 132.0 (2C), 130.6 (2C), 128.7, 126.6, 119.2, 111.7, 110.2, 108.1, 75.7, 63.3, 56.2, 56.0, 42.8, 28.6.

HRMS (ESI): *m/z* calcd. For $C_{19}H_{20}NO_3$ $[M+H]^+$ 310.1432, found 310.1438.

***6,7-Dimethoxy-1-(4-(trifluoromethyl)benzyl)isochromane* (2g)**

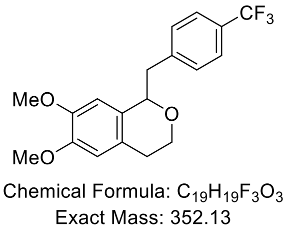

The general procedure was followed with 2-(4-trifluoromethylphenyl)oxirane (37.6 mg, 0.20 mmol) and 2-(3,4-dimethoxyphenyl)ethanol (73.0 mg, 0.4 mmol) in the presence of TfOH (1.8 µL, 0.020 mmol, 10 mol%) in HFIP (2.0 mL). The reaction mixture was stirred at 25 °C for 0.25 h. Purification by FC over silica gel (*n*-pentane/EtOAc 100:0 to 90:10 gradient) afforded **2g** (40.0 mg, 57% yield) as a white solid.

Mp = 84 − 86 °C

^{1}H NMR (400 MHz, CDCl$_3$): δ 7.54 (d, *J* = 8.0 Hz, 2H), 7.37 (d, *J* = 8.0 Hz, 2H), 6.60 (s, 1H), 6.52 (s, 1H), 4.98 (dd, *J* = 8.3, 4.1 Hz, 1H), 4.10 (ddd, *J* = 11.2, 4.9, 4.1 Hz, 1H), 3.86 (s, 3H), 3.81 (s, 3H), 3.72 (ddd, *J* = 11.2, 8.8, 4.1 Hz, 1H), 3.23 (dd, *J* = 14.2 Hz, 4.1 Hz, 1H), 3.11 (dd, *J* = 14.2 Hz, 8.3 Hz, 1H), 2.81 (ddd, *J* = 15.7, 8.8, 4.9 Hz, 1H), 2.60 (ddd, *J* = 15.7, 4.1, 4.1 Hz, 1H).

^{13}C NMR (100 MHz, CDCl$_3$): δ 147.9, 147.5, 143.0 (q, *J* = 1.4 Hz), 130.0 (2C), 129.1, 128.7 (q, *J* = 32.3 Hz), 126.5, 125.2 (q, *J* = 3.8 Hz, 2C), 124.5 (q, *J* = 271.8 Hz), 111.7, 108.2, 76.0, 63.2, 56.1, 56.0, 42.6, 28.7.

^{19}F NMR (377 MHz, CDCl$_3$): δ -62.3.

HRMS (ESI): *m/z* calcd. For $C_{19}H_{20}O_3F_3$ $[M+H]^+$ 353.1353, found 353.1359.

***Methyl 4-((6,7-dimethoxyisochroman-1-yl)methyl)benzoate* (2h)**

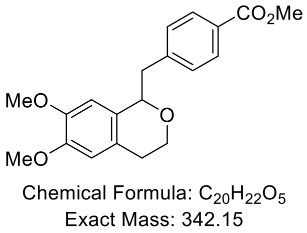

The general procedure was followed with methyl 4-(oxiran-2-yl)benzoate (35.6 mg, 0.16 mmol) and 2-(3,4-dimethoxyphenyl)ethan-1-ol (58.4 mg, 0.32 mmol) in the presence of TfOH (1.4 µL, 0.016 mmol, 10 mol%) in HFIP (2.0 mL). The reaction mixture was stirred at 25 °C for 1 h. Purification by FC over silica gel (*n*-pentane/EtOAc 100:0 to 80:20 gradient) afforded **2h** (41.0 mg, 77% yield) as a colorless oil. The product was contaminated with a side-product resulting from a transesterification with HFIP. The corrected yield is 69%.

^{1}H NMR (400 MHz, CDCl$_3$): δ 7.95 (d, *J* = 8.3 Hz, 2H), 7.33 (d, *J* = 8.3 Hz, 2H), 6.58 (s, 1H), 6.53 (s, 1H), 4.99 (dd, *J* = 8.2, 4.1 Hz, 1H), 4.09 (ddd, *J* = 11.2, 5.2, 4.1 Hz, 1H), 3.89 (s, 3H), 3.85 (s, 3H), 3.80 (s, 3H), 3.71 (ddd, *J* = 11.2, 9.0, 4.1 Hz, 1H), 3.23 (dd, *J* = 14.2, 4.1 Hz, 1H), 3.10 (dd, *J* = 14.2, 8.2 Hz, 1H), 2.79 (ddd, *J* = 14.6, 9.0, 5.2 Hz, 1H), 2.58 (ddd, *J* = 14.6, 4.1, 4.1 Hz, 1H).

^{13}C NMR (126 MHz, CDCl$_3$): δ 167.2, 147.8, 147.4, 144.3, 129.7 (2C), 129.6 (2C), 129.1, 128.2, 126.4, 111.6, 108.1, 76.0, 63.1, 56.1, 55.9, 52.1, 42.8, 28.6.

HRMS (ESI): m/z calcd. for $C_{20}H_{23}O_5$ [M+H]$^+$ 343.1540, found 343.1533.

1-(4-((6,7-Dimethoxyisochroman-1-yl)methyl)phenyl)ethan-1-one **(2i)**

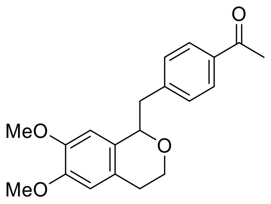

Chemical Formula: $C_{20}H_{22}O_4$
Exact Mass: 326.15

The general procedure was followed with 2-(4-acetylphenyl)oxirane (32.4 mg, 0.20 mmol) and 2-(3,4-dimethoxyphenyl)ethan-1-ol (73.0 mg, 0.4 mmol) in the presence of TfOH (1.8 µL, 0.020 mmol, 10 mol%) in HFIP (2.0 mL). The reaction mixture was stirred at 25 °C for 0.25 h. A first purification by FC over silica gel (*n*-pentane/EtOAc 95:5 to 65:35 gradient) followed by a second purification (*n*-pentane/EtOAc 100:0 to 80:20) afforded **2i** (47.7 mg, 73% yield) as a colorless oil.

^{1}H NMR (400 MHz, CDCl$_3$): δ 7.88 (d, *J* = 8.3 Hz, 2H), 7.36 (d, *J* = 8.3 Hz, 2H), 6.59 (s, 1H), 6.55 (s, 1H), 4.99 (dd, *J* = 8.3, 4.0 Hz, 1H), 4.10 (ddd, *J* = 11.2, 4.6, 4.6 Hz, 1H), 3.86 (s, 3H), 3.82 (s, 3H), 3.72 (ddd, *J* = 11.2, 9.0, 3.9 Hz, 1H), 3.24 (dd, *J* = 14.2, 4.0 Hz, 1H), 3.10 (dd, *J* = 14.2, 8.3 Hz, 1H), 2.81 (ddd, *J* = 16.8, 9.0, 4.6 Hz, 1H), 2.58 (s, 3H), 2.62 – 2.56 (ddd, *J* = 16.8, 4.6, 3.9 Hz, 1H).

^{13}C NMR (100 MHz, CDCl$_3$): δ 198.0, 147.9, 147.5, 144.7, 135.5, 129.9 (2C), 129.2, 128.4 (2C), 126.5, 111.7, 108.2, 76.0, 63.2, 56.2, 56.0, 42.8, 28.7, 26.7.

HRMS (ESI): *m/z* calcd. For $C_{20}H_{23}O_4$ [M+H]$^+$ 327.1571, found 327.1591.

1-(4-Bromobenzyl)-6,7-dimethoxyisochromane **(2j)**

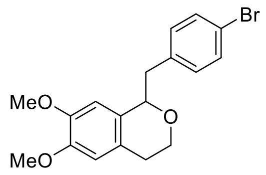

Chemical Formula: $C_{18}H_{19}BrO_3$
Exact Mass: 362.05

The general procedure was followed with 2-(4-bromophenyl)oxirane (39.8 mg, 0.2 mmol) and 2-(3,4-dimethoxyphenyl)ethan-1-ol (73.0 mg, 0.4 mmol) in the presence of TfOH (1.8 µL, 0.020 mmol, 10 mol%) in HFIP (2.0 mL). The reaction mixture was stirred at 25 °C for 0.25 h. Purification by FC over silica gel (*n*-pentane/EtOAc 100:0 to 85:15 gradient) afforded **2j** (29.0 mg, 40% yield) as a colorless oil. Spectral data are in accordance with those found in the literature.[1]

^{1}H NMR (400 MHz, CDCl$_3$): δ 7.40 (d, *J* = 8.3 Hz, 2H), 7.13 (d, *J* = 8.3 Hz, 2H), 6.58 (s, 1H), 6.51 (s, 1H), 4.93 (dd, *J* = 8.1, 4.2 Hz, 1H), 4.09 (ddd, *J* = 11.2, 5.0, 4.2 Hz, 1H), 3.86 (s, 3H), 3.81 (s, 3H), 3.70 (ddd, *J* = 11.2, 9.0, 4.2 Hz, 1H), 3.13 (dd, *J* = 14.2, 4.2 Hz, 1H), 3.00 (dd, *J* = 14.2, 8.1 Hz, 1H), 2.80 (ddd, *J* = 15.9, 9.0, 5.0 Hz, 1H), 2.59 (ddd, *J* = 15.9, 4.2, 4.2 Hz, 1H).

^{13}C NMR (126 MHz, CDCl$_3$): δ 147.7, 147.3, 137.6, 131.4 (2C), 131.2 (2C), 129.1, 126.4, 120.1, 111.5, 108.2, 76.0, 63.0, 56.0, 55.9, 42.1, 28.6.

HRMS (ESI): m/z calcd. for $C_{18}H_{20}O_3Br$ $[M+H]^+$ 363.0590, found 363.0572.

1-Benzyl-6,7-dimethoxyisochromane **(2k)**

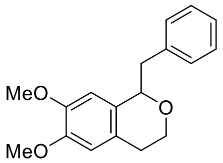

Chemical Formula: $C_{18}H_{20}O_3$
Exact Mass: 284.14

The general procedure was followed with 2-phenyloxirane (24.0 mg, 0.20 mmol) and 2-(3,4-dimethoxyphenyl)ethanol (73.0 mg, 0.4 mmol) in the presence of TfOH (0.4 µL, 0.004 mmol, 2 mol%) in HFIP (2.0 mL). The reaction mixture was stirred at 25 °C for 0.25 h. Purification by FC over silica gel (*n*-pentane/EtOAc 100:0 to 95:5 gradient) afforded **2k** (31.2 mg, 55% yield) as a colorless oil. Spectral data are in accordance with those found in the literature.[1]

^{1}H NMR (400 MHz, CDCl$_3$): δ 7.30 – 7.18 (m, 5H), 6.57 (s, 1H), 6.44 (s, 1H), 4.95 (dd, *J* = 7.8, 5.0 Hz, 1H), 4.09 (ddd, *J* = 11.2, 5.0, 4.3 Hz, 1H), 3.83 (s, 3H), 3.73 (s, 3H), 3.75 – 3.70 (ddd, *J* = 11.2, 8.4, 4.3 Hz, 1H), 3.13 (dd, *J* = 14.2, 5.0 Hz, 1H), 3.06 (dd, *J* = 14.2, 7.8 Hz, 1H), 2.80 (ddd, *J* = 15.9, 8.4, 5.0 Hz, 1H), 2.60 (ddd, *J* = 15.9, 4.3, 4.3 Hz, 1H).

^{13}C NMR (100 MHz, CDCl$_3$): δ 147.7, 147.3, 138.9, 129.7 (3C), 128.4 (2C), 126.4, 126.3, 111.6, 108.5, 76.4, 62.9, 56.0, 56.0, 42.9, 28.8.

HRMS (ESI): *m/z* calcd. For $C_{18}H_{21}O_3$ $[M+H]^+$ 285.1480, found 285.1485.

6,7-Dimethoxy-1-(3-nitrobenzyl)isochromane **(2m)**

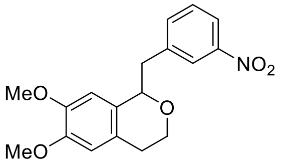

The general procedure was followed with 2-(3-nitrophenyl)oxirane (33.0 mg, 0.2 mmol) and 2-(3,4-dimethoxyphenyl)ethan-1-ol (73.0 mg, 0.4 mmol) in the presence of TfOH (1.8 µL, 0.020 mmol, 10 mol%) in HFIP (2.0 mL). The reaction mixture was stirred at 25 °C for 0.25 h. Purification by FC over silica gel (*n*-pentane/EtOAc 100:0 to 80:20 gradient) afforded **2m** (47.4 mg, 72% yield) as a yellow oil.

^{1}H NMR (400 MHz, CDCl$_3$): δ 8.16 (t, *J* = 2.0 Hz, 1H), 8.07 (ddd, *J* = 8.2, 2.3, 1.1 Hz, 1H), 7.58 (dt, *J* = 7.7, 1.4 Hz, 1H), 7.43 (t, *J* = 7.9 Hz, 1H), 6.60 (s, 1H), 6.59 (s, 1H), 4.99 (dd, *J* = 8.5, 3.5 Hz, 1H), 4.11 (ddd, *J* = 11.2, 5.1, 3.8 Hz, 1H), 3.86 (s, 3H), 3.86 (s, 3H), 3.71 (ddd, *J* = 11.2, 9.4, 3.8 Hz, 1H), 3.30 (dd, *J* = 14.4, 3.5 Hz, 1H), 3.12 (dd, *J* = 14.4, 8.5 Hz, 1H), 2.81 (ddd, *J* = 15.9, 9.4, 5.1 Hz, 1H), 2.58 (ddd, *J* = 15.9, 3.8, 3.8 Hz, 1H).

^{13}C NMR (101 MHz, CDCl$_3$): δ 148.3, 148.0, 147.7, 140.8, 136.1, 129.0, 128.7, 126.7, 124.7, 121.6, 111.8, 108.1, 75.8, 63.3, 56.3, 56.0, 42.2, 28.7.

HRMS (ESI): *m/z* calcd. For $C_{18}H_{20}NO_5$ $[M+H]^+$ 330.1336, found 330.1329.

1-(2-Bromobenzyl)-6,7-dimethoxyisochromane **(2n)**

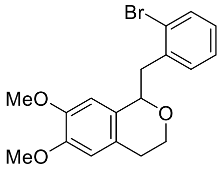

The general procedure was followed with 2-(2-bromophenyl)oxirane (39.8 mg, 0.20 mmol) and 2-(3,4-dimethoxyphenyl)ethan-1-ol (73.0 mg, 0.4 mmol) in the presence of TfOH (1.8 µL, 0.020 mmol, 10 mol%) in HFIP (2.0 mL). The reaction mixture was stirred at 25 °C for 0.25 h. Purification by FC over silica gel (*n*-pentane/EtOAc 100:0 to 80:20 gradient) afforded **2n** (33.0 mg, 46% yield) as a colorless oil. Spectral data are in accordance with those found in the literature.[2]

^{1}H NMR (400 MHz, CDCl$_3$): δ 7.58 (dd, *J* = 8.3, 1.3 Hz, 1H), 7.35 (dd, *J* = 7.5, 1.7 Hz, 1H), 7.26 (ddd, *J* = 7.5, 1.7, 1.3 Hz, 1H), 7.11 (ddd, *J* = 7.6, 1.7, 1.3 Hz, 1H), 6.68 (s, 1H), 6.62 (s, 1H), 5.02 (dd, *J* = 9.5, 3.3 Hz, 1H), 4.12 (ddd, *J* = 11.3 Hz, 4.9, 4.7 Hz, 1H), 3.87 (s, 3H), 3.83 (s, 3H), 3.76 (ddd, *J* = 11.3, 7.8, 4.7 Hz, 1H), 3.38 (dd, *J* = 14.2, 3.3 Hz, 1H), 3.09 (dd, *J* = 14.3, 9.5 Hz, 1H), 2.90 – 2.84 (ddd, *J* = 15.9, 7.8, 4.9 1H), 2.69 (ddd, *J* = 15.9, 4.7, 4.7 Hz, 1H).

^{13}C NMR (100 MHz, CDCl$_3$): δ 147.8, 147.5, 138.4, 132.8, 132.4, 129.6, 128.3, 127.4, 126.2, 124.9, 111.5, 108.4, 74.8, 62.6, 56.1, 56.0, 43.0, 28.7.

HRMS (ESI): *m/z* calcd. For $C_{18}H_{20}O_3Br$ $[M+H]^+$ 363.0590, found 363.0571.

6,7-Dimethoxy-1-phenethylisochromane **(2o)**

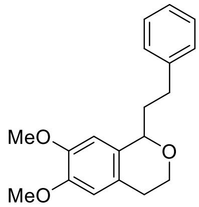

Chemical Formula: $C_{19}H_{22}O_3$
Exact Mass: 298.16

The general procedure was followed with 2-benzyloxirane (26.3 µL, 0.2 mmol) and 2-(3,4-dimethoxyphenyl)ethan-1-ol (73.0 mg, 0.4 mmol) in the presence of TfOH (0.4 µL, 0.004 mmol, 2 mol%) in HFIP (2.0 mL). The reaction mixture was stirred at 25 °C for 0.25 h. Purification by FC over silica gel (*n*-pentane/EtOAc 100:0 to 80:20 gradient) afforded **2o** (46.0 mg, 77% yield) as a yellow oil.

^{1}H NMR (400 MHz, CDCl$_3$): δ 7.38 – 7.14 (m, 5H), 6.62 (s, 1H), 6.54 (s, 1H), 4.73 (dd, *J* = 8.5, 3.7 Hz, 1H), 4.19 (ddd, *J* = 11.3, 5.4, 3.8 Hz, 1H), 3.87 (s, 3H), 3.84 (s, 3H), 3.80 (ddd, *J* = 11.3, 9.5, 3.8 Hz, 1H), 2.96 (ddd, *J* = 15.7, 9.5, 5.4 1H), 2.85 – 2.73 (m, 2H), 2.64 (ddd, *J* = 15.7, 3.8, 3.8 Hz, 1H), 2.27 – 2.00 (m, 2H).

^{13}C NMR (101 MHz, CDCl$_3$): δ 147.6, 147.6, 142.6, 130.1, 128.6 (2C), 128.5 (2C), 126.2, 125.8, 111.6, 107.9, 75.0, 63.4, 56.1, 56.0, 37.9, 31.6, 28.8.

HRMS (ESI): m/z calcd. for $C_{19}H_{23}O_3$ $[M+H]^+$ 299.1642, found 299.1633.

***1-Heptyl-6,7-dimethoxyisochromane* (2p)**

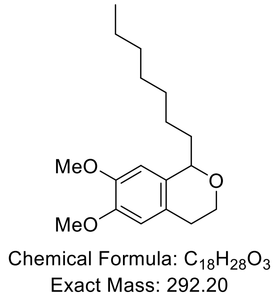

A modified version of the general procedure was followed with 2-hexyloxirane (183.4 µL, 1.2 mmol) and 2-(3,4-dimethoxyphenyl)ethan-1-ol (73.0 mg, 0.4 mmol) in the presence of TfOH (3.6 µL, 0.040 mmol, 10 mol%) in HFIP (4.0 mL). The reaction mixture was stirred at 25 °C for 16 h. Purification by FC over silica gel (*n*-pentane/EtOAc 100:0 to 85:15 gradient) afforded **2p** (78.0 mg, 67% yield) as a colorless oil.

^{1}H NMR (400 MHz, CDCl$_3$): δ 6.58 (s, 1H), 6.55 (s, 1H), 4.67 (dd, *J* = 8.5, 3.2 Hz, 1H), 4.11 (ddd, *J* = 11.2, 5.3, 3.7 Hz, 1H), 3.85 (s, 3H), 3.84 (s, 3H), 3.73 (ddd, *J* = 11.2, 9.4, 3.7 Hz, 1H), 2.88 (ddd, *J* = 15.9, 9.4, 5.3 Hz, 1H), 2.59 (ddd, *J* = 15.9, 3.7, 3.7 Hz, 1H), 1.91 – 1.72 (m, 2H), 1.50 – 1.41 (m, 2H), 1.29 (m, 8H), 0.93 – 0.80 (m, 3H).

^{13}C NMR (101 MHz, CDCl$_3$): δ 147.5 (2C), 130.6, 126.1, 111.6, 108.1, 75.7, 63.3, 56.1, 56.0, 36.2, 32.0, 29.9, 29.4, 28.8, 25.4, 22.8, 14.2.

HRMS (ESI): m/z calcd. for $C_{18}H_{29}O_3$ $[M+H]^+$ 293.2111, found 293.2102.

***6,7-Dimethoxy-1-((4-nitrophenyl)(phenyl)methyl)isochromane* (2q)**

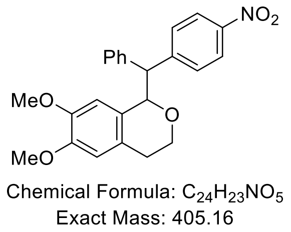

The general procedure was followed with 2-phenyl-2-(4-nitrophenyl)oxirane (48.0 mg, 0.20 mmol) and 2-(3,4-dimethoxyphenyl)ethan-1-ol (73.0 mg, 0.4 mmol) in the presence of TfOH (1.8 µL, 0.020 mmol, 10 mol%) in HFIP (2.0 mL). The reaction mixture was stirred at 25 °C for 0.25 h. Purification by FC over silica gel (*n*-pentane/EtOAc 90:10 to 70:30 gradient) afforded **2q** as two diastereoisomers (44.0 mg, 54% yield, 70:30 *dr*) as a yellow oil.

^{1}H NMR (400 MHz, CDCl$_3$): δ 8.15 (d, *J* = 8.6 Hz, 2H, minor), 8.02 (d, *J* = 8.6 Hz, 2H, major), 7.58 (d, *J* = 8.6 Hz, 2H, major), 7.56 (d, *J* = 8.6 Hz, 2H, minor), 7.40 – 7.12 (m, 5H, major + minor), 6.51 (s, 1H, major), 6.49 (s, 1H, minor), 6.44 (s, 1H, minor), 6.31 (s, 1H, major), 5.52 (d, *J* = 5.1 Hz, 1H, major), 5.48 (d, *J* = 3.9 Hz, 1H, minor), 4.63 (d, *J* = 3.9 Hz, 1H, minor), 4.57 (d,

J = 5.1 Hz, 1H, major), 4.07 (m, 1H, major + minor), 3.83 (s, 3H, major), 3.82 (s, 3H, minor), 3.71 (s, 3H, minor), 3.64 (s, 3H, major), 3.69 – 3.61 (m, 1H, major + minor), 2.58 (ddd, *J* = 15.9, 9.5, 5.1 Hz, 1H, major + minor), 2.49 (ddd, *J* = 15.9, 3.9, 3.9 Hz, 1H, major), 2.42 (ddd, *J* = 15.9, 3.1, 3.1 Hz, 1H, minor).

^{13}C NMR (100 MHz, CDCl$_3$): δ 150.7 (minor), 149.1 (major), 147.8 (major), 147.7 (minor), 147.3 (minor), 147.2 (major), 146.6 (minor), 146.5 (major), 141.5 (major), 139.2 (minor), 130.7 (2C, major), 130.3 (2C, minor), 130.1 (2C, minor), 129.3 (2C, major), 128.8 (2C, major), 128.1 (2C, minor), 127.7 (major), 127.7 (minor), 127.6 (minor), 127.2 (major), 127.1 (major), 126.8 (minor), 123.5 (2C, minor), 123.0 (2C, major), 111.4 (both), 108.7 (major), 108.4 (minor), 77.8 (minor), 77.3 (major), 64.0 (minor), 63.4 (major), 57.3 (major), 56.9 (minor), 56.0 (minor), 55.9 (major + minor), 55.9 (major), 28.6 (major + minor).

HRMS (ESI): *m/z* calcd. For $C_{24}H_{23}NO_5Na$ $[M+Na]^+$ 428.1468, found 428.1430.

6,7-Dimethoxy-1-(1-(4-(trifluoromethyl)phenyl)ethyl)isochromane (2r)

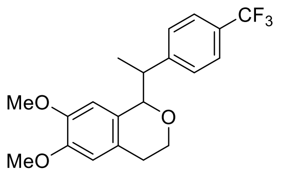

Chemical Formula: $C_{20}H_{21}F_3O_3$
Exact Mass: 366.14

The general procedure was followed with 2-methyl-2-(4-(trifluoromethyl)phenyl)oxirane (40.4 mg, 0.2 mmol) and 2-(3,4-dimethoxyphenyl)ethan-1-ol (73.0 mg, 0.4 mmol) in the presence of TfOH (1.8 µL, 0.020 mmol, 10 mol%) in HFIP (2.0 mL). The reaction mixture was stirred at 25 °C for 1 h. Purification by FC over silica gel (*n*-pentane/EtOAc 100:0 to 80:20 gradient) afforded **2r** (67.0 mg, 92% yield, 57:43 *dr*) as a yellow oil.

^{1}H NMR (400 MHz, CDCl$_3$): δ 7.58 (d, *J* = 8.3 Hz, 2H, major), 7.48 (d, *J* = 8.3 Hz, 2H, major), 7.39 (d, *J* = 8.3 Hz, 2H, minor), 7.26 (d, *J* = 8.3 Hz, 2H, minor), 6.61 (s, 1H, major), 6.45 (s, 2H, minor), 6.37 (s, 1H, major), 4.99 (d, *J* = 4.1 Hz, 1H, major), 4.84 (d, *J* = 3.2 Hz, 1H, minor), 4.11 (dd, *J* = 11.1, 5.4 Hz, 1H, major + minor), 3.86 (s, 3H, major), 3.80 (s, 3H, minor), 3.77 (s, 3H, minor), 3.73 (s, 3H, major), 3.63 (m, 1H, major + minor), 3.41 (dd, *J* = 7.2, 3.2 Hz, 1H, minor), 3.23 (dd, *J* = 7.1, 4.1 Hz, 1H, major), 2.91 (ddd, *J* = 15.6, 10.0, 5.3 Hz, 1H, major), 2.54 (m, 1H, major + minor), 2.33 (m, 1H, minor), 1.38 (d, *J* = 7.2 Hz, 3H, minor), 1.11 (d, *J* = 7.1 Hz, 3H, major).

^{13}C NMR (126 MHz, CDCl$_3$): δ 149.0 (q, *J* = 1.3 Hz), 147.7, 147.4, 147.4, 147.2, 146.9 (q, *J* = 1.3 Hz), 129.4 (2C), 128.9 (2C), 128.6 (q, *J* = 32.3 Hz, 2C), 128.4, 128.3 (q, *J* = 32.2 Hz, 2C), 128.0, 127.5, 127.3, 125.1 (q, *J* = 3.9 Hz), 124.5 (q, *J* = 3.8 Hz), 124.5 (q, *J* = 271.7 Hz, 2C), 111.5, 111.3, 108.4, 108.2, 79.6, 79.3, 64.1, 63.5, 56.0, 55.9, 55.9, 55.8, 45.4, 45.3, 28.8 (2C), 17.8, 14.3.

^{19}F NMR (471 MHz, CDCl$_3$): δ -62.27 (minor), -62.28 (major).

HRMS (ESI): m/z calcd. for $C_{20}H_{21}O_3F_3Na$ [M+Na] + 389.1335, found 389.1322.

6,7-Dimethoxy-1-(1-phenylheptyl)isochromane **(2s)**

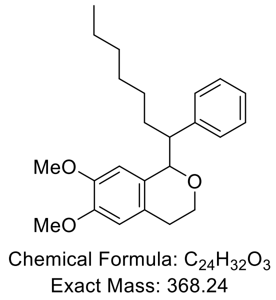

The general procedure was followed with 2-hexyl-2-phenyloxirane (40.8 mg, 0.2 mmol) and 2-(3,4-dimethoxyphenyl)ethan-1-ol (73.0 mg, 0.4 mmol) in the presence of TfOH (0.4 µL, 0.004 mmol, 2 mol%) in HFIP (2.0 mL). The reaction mixture was stirred at 25 °C for 1 h. Purification by FC over silica gel (*n*-pentane/EtOAc 100:0 to 90:10 gradient) afforded **2s** as two diastereoisomers (56.0 mg, 76% yield, 57:43 *dr*) as a yellow oil.

^{1}H NMR (400 MHz, $CDCl_3$): δ 7.29 – 7.22 (m, 2H major, 3H minor), 7.20 – 7.14 (m, 1H, major), 7.10 – 6.97 (m, 2H, major + minor), 6.52 (s, 1H, major), 6.48 (s, 1H, minor), 6.33 (s, 1H, minor), 6.12 (s, 1H, major), 5.01 – 4.90 (m, 1H, minor), 4.72 (d, *J* = 5.3 Hz, 1H, major), 4.10 – 4.02 (m, 1H, major + minor), 3.79 (s, 3H, major), 3.77 (s, 3H, minor), 3.72 (s, 3H, minor), 3.63 (ddd, *J* = 11.2, 8.5, 4.1 Hz, 1H, major), 3.59 – 3.51 (m, 3H major, 1H minor), 3.07 – 2.91 (m, 1H, major + minor), 2.84 – 2.73 (m, 1H, major), 2.61 – 2.41 (m, 1H, major + minor), 1.92 – 1.54 (m, 2H, major + minor), 1.31 – 0.91 (m, 8H, major + minor), 0.85 – 0.70 (m, 3H, major + minor).

^{13}C NMR (101 MHz, $CDCl_3$): δ 147.4, 147.0, 147.0, 146.8, 143.1, 141.3, 129.7 (2C), 129.4 (2C), 129.1, 128.7, 128.2 (2C), 127.4 (2C), 127.3, 126.6, 126.4, 125.9, 111.3, 111.1, 108.7, 108.5, 79.6, 78.9, 64.2, 62.7, 56.0, 55.8, 55.7 (2C), 51.8, 51.5, 32.4, 31.8, 31.7, 29.6, 29.3, 29.3, 28.8, 28.7, 28.0, 27.5, 22.7, 22.6, 14.1, 14.1.

HRMS (ESI): m/z calcd. for $C_{24}H_{33}O_3$ $[M+H]^+$ 369.2424, found 369.2433.

1-(4-(6,7-Dimethoxyisochroman-1-yl)pentyl)-3,7-dimethyl-3,7-dihydro-1H-purine-2,6-dione **(2t)**

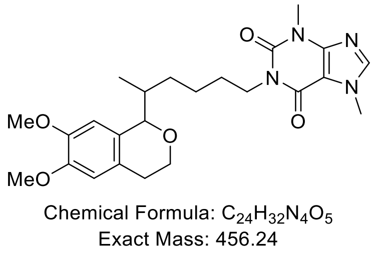

The general procedure was followed with 3,7-dimethyl-1-(4-(2-methyloxiran-2-yl)butyl)-3,7-dihydro-1H-purine-2,6-dione (59.0 mg, 0.2 mmol) and 2-(3,4-dimethoxyphenyl)ethan-1-ol (73.0 mg, 0.4 mmol) in the presence of TfOH (1.8 µL, 0.020 mmol, 10 mol%) in HFIP (2.0 mL). The reaction mixture was stirred at 25 °C for 10 min and then TfOH (1.8 µL, 0.020 mmol, 10 mol%) was added. The operation was repeated twice for a reaction time of 1 h Purification by FC over silica gel (*n*-pentane/EtOAc 100:0 to 85:15 gradient) afforded **2t** as two diastereoisomers (70.0 mg, 77% yield, 57:43 *dr*) as a colorless oil.

^{1}H NMR (400 MHz, CDCl$_3$): δ 7.48 (d, *J* = 0.7 Hz, 1H, major), δ 7.46 (d, *J* = 0.7 Hz, 1H, minor) 6.54 (s, 1H, minor), 6.54 (s, 1H, minor), 6.53 (s, 1H, major), 6.53 (s, 1H, major), 4.65 – 4.63 (m, 1H, major), 4.60 – 4.56 (m, 1H, minor), 4.12 – 4.06 (m, 2H, minor), 4.03 – 3.97 (m, 2H, major), 3.95 (d, *J* = 0.7 Hz, 3H, major), 3.93 (d, *J* = 0.7 Hz, 3H, minor), 3.87 (ddd, *J* = 8.8, 6.1, 5.2 Hz, 1H, major + minor), 3.83 – 3.81 (m, 6H major, 3H minor), 3.79 (s, 3H, minor), 3.62 – 3.55 (m, 1H, major + minor), 3.54 (s, 3H, major), 3.51 (s, 3H, minor), 2.95 – 2.85 (m, 1H, major + minor), 2.45 – 2.42 (m, 1H, major), 2.41 – 2.38 (m, 1H, minor), 2.02 – 1.92 (m, 1H, major + minor), 1.73 – 1.60 (m, 2H, major + minor), 1.51 – 1.43 (m, 3H, major + minor), 1.16 – 1.12 (m, 1H, major + minor), 1.09 (d, *J* = 6.9 Hz, 3H, minor), 0.63 (d, *J* = 6.9 Hz, 3H, major).

^{13}C NMR (101 MHz, CDCl$_3$): δ 155.3, 155.2, 151.5, 151.4, 148.7, 148.7, 147.5, 147.4, 147.2, 147.2, 141.4, 141.4, 129.8, 129.5, 127.2, 127.2, 111.4, 111.4, 107.8, 107.7 (2C), 107.6, 80.5, 78.5, 64.4, 64.3, 56.1, 56.0, 55.8 (2C), 41.5, 41.5, 38.4, 38.2, 33.6, 33.6, 33.5, 29.7, 29.6, 29.1, 29.0, 29.0, 28.2, 28.1, 25.2, 25.2, 17.1, 12.9.

HRMS (ESI): m/z calcd. for $C_{24}H_{33}N_4O_5$ $[M+H]^+$ 457.2445, found 457.2432.

1-Benzyl-6,7-dimethoxy-1-methylisochromane **(2u)**

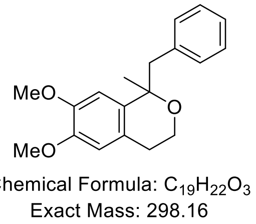

The general procedure was followed with 2-methyl-3-phenyloxirane (26.8 mg, 0.2 mmol) and 2-(3,4-dimethoxyphenyl)ethan-1-ol (73.0 mg, 0.4 mmol) in the presence of TfOH (1.8 µL, 0.020 mmol, 10 mol%) in HFIP (2.0 mL). The reaction mixture was stirred at 25 °C for 1 h. Purification by FC over silica gel (*n*-pentane/EtOAc 100:0 to 85:15 gradient) afforded **2u** (37.0 mg, 62% yield) as a colorless oil.

^{1}H NMR (400 MHz, CDCl$_3$): δ 7.23 – 7.17 (m, 3H), 7.11 (dd, *J* = 7.6, 1.8 Hz, 2H), 6.52 (s, 1H), 6.51 (s, 1H), 3.98 (ddd, *J* = 11.0, 6.4, 4.4 Hz, 1H), 3.89 (ddd, *J* = 11.0, 6.6, 4.4 Hz, 1H), 3.85 (s, 3H), 3.83 (s, 3H), 3.18 (d, *J* = 13.7 Hz, 1H), 2.98 (d, *J* = 13.7 Hz, 1H), 2.65 (ddd, *J* = 15.7, 6.4, 4.4 Hz, 1H), 2.54 (ddd, *J* = 15.7, 6.6, 4.4 Hz, 1H), 1.47 (s, 3H).

^{13}C NMR (101 MHz, CDCl$_3$): δ 147.4, 147.2, 137.8, 133.4, 130.9 (2C), 127.7 (2C), 126.4, 126.1, 111.2, 109.4, 76.7, 59.9, 56.1, 55.9, 48.0, 29.3, 27.9.

HRMS (ESI): m/z calcd. for $C_{19}H_{23}O_3$ $[M+H]^+$ 299.1642, found 299.1637.

4-(Isochroman-1-ylmethyl)benzonitrile **(2v)**

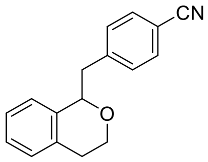

Chemical Formula: $C_{17}H_{15}NO$
Exact Mass: 249.12

The general procedure was followed with 4-(oxiran-2-yl)benzonitrile (29.0 mg, 0.2 mmol) and phenylethanol (48.0 µL, 0.4 mmol) in the presence of TfOH (1.8 µL, 0.020 mmol, 10 mol%) in HFIP (2.0 mL). The reaction mixture was stirred at 25 °C for 1 h. Purification by FC over silica gel (*n*-pentane/EtOAc 100:0 to 85:15 gradient) afforded **2v** (35.5 mg, 71% yield) as a white solid. Spectral data are in accordance with those found in the literature.[3]

m.p. = 109 – 111 °C

^{1}H NMR (400 MHz, CDCl$_3$): δ 7.56 (d, *J* = 8.4 Hz, 2H), 7.36 (d, *J* = 8.4 Hz, 2H), 7.25 – 7.01 (m, 4H), 5.05 (dd, *J* = 8.6, 3.4 Hz, 1H), 4.11 (ddd, *J* = 11.2, 5.3, 3.8 Hz, 1H), 3.73 (ddd, *J* = 11.2, 9.5, 3.8 Hz, 1H), 3.30 (dd, *J* = 14.3, 3.4 Hz, 1H), 3.10 (dd, *J* = 14.3, 8.6 Hz, 1H), 2.88 (ddd, *J* = 16.1, 9.5, 5.3 Hz, 1H), 2.66 (ddd, *J* = 16.1, 3.8, 3.8 Hz, 1H).

^{13}C NMR (126 MHz, CDCl$_3$): δ 144.4, 136.9, 134.3, 131.9 (2C), 130.4 (2C), 129.1, 126.7, 126.2, 124.9, 119.2, 110.1, 76.0, 63.2, 42.5, 29.0.

HRMS (ESI): m/z calcd. for $C_{17}H_{14}ON$ [M+H] + 250.1133, found 250.1226.

5-(4-Nitrobenzyl)-7,8-dihydro-5H-[1,3]dioxolo[4,5-g]isochromene **(2w)**

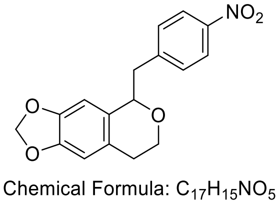

Chemical Formula: $C_{17}H_{15}NO_5$
Exact Mass: 313.10

The general procedure was followed with 2-(4-nitrophenyl)oxirane (33.0 mg, 0.2 mmol) and 2-(benzo[d][1,3]dioxol-5-yl)ethanol (66.5 mg, 0.40 mmol) in the presence of TfOH (1.8 µL, 0.020 mmol, 10 mol%) in HFIP (2.0 mL). The reaction mixture was stirred at 25 °C for 0.25 h. Purification by FC over silica gel (*n*-pentane/EtOAc 95:5 to 85:15 gradient) afforded **2w** (46.0 mg, 73% yield) as a yellow solid.

m.p. = 167 − 169 °C.

^{1}H NMR (400 MHz, CDCl$_3$): δ 8.12 (d, *J* = 8.7 Hz, 2H), 3.93 (d, *J* = 8.7 Hz, 2H), 6.62 (s, 1H), 6.55 (s, 1H), 5.93 – 5.92 (m, 2H), 4.96 (dd, *J* = 8.3, 3.5 Hz, 1H), 4.06 (ddd, *J* = 11.2, 5.1, 3.7 Hz, 1H), 3.66 (ddd, *J* = 11.2, 9.5, 3.7 Hz, 1H), 3.27 (dd, *J* = 14.3, 3.5 Hz, 1H), 3.09 (dd, *J* = 14.3, 8.3 Hz, 1H), 2.75 (ddd, *J* = 16.0, 9.5, 5.1 Hz, 1H), 2.53 (ddd, *J* = 16.0, 3.7, 3.7 Hz, 1H).

^{13}C NMR (100 MHz, CDCl$_3$): δ 146.8, 146.6, 146.4, 146.3, 130.6 (2C), 129.7, 127.8, 123.4 (2C), 108.9, 105.0, 101.0, 76.1, 63.3, 42.5, 29.2.

HRMS (ESI): *m/z* calcd. For $C_{17}H_{15}NO_5Na$ $[M+Na]^+$ 336.0830, found 336.0842.

***1-(4-Nitrobenzyl)isochromane-6,7-diol* (2x)**

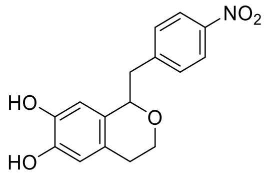

The general procedure was followed with 2-(4-nitrophenyl)oxirane (33.0 mg, 0.2 mmol) and 4-(2-hydroxyethyl)benzene-1,2-diol (61.6 mg, 0.40 mmol) in the presence of TfOH (1.8 µL, 0.020 mmol, 10 mol%) in HFIP (2.0 mL). The reaction mixture was stirred at 25 °C for 1 h. Purification by FC over silica gel (*n*-pentane/EtOAc 80:20 to 50:50 gradient) afforded **2x** (24.0 mg, 40% yield) as a yellow solid.

m.p. = decomposition at 190 °C.

^{1}H NMR (400 MHz, acetone-*d*$_6$): δ 8.13 (d, *J* = 8.7 Hz, 1H), 7.79 (brs, 2H), 7.58 (d, *J* = 8.7 Hz, 2H), 6.77 (s, 1H), 6.57 (s, 1H), 4.92 (dd, *J* = 8.9, 3.2 Hz, 1H), 4.02 (ddd, *J* = 11.2, 5.1, 4.0 Hz, 1H), 3.61 (ddd, *J* = 11.2, 9.2, 3.8 Hz, 1H), 3.34 (dd, *J* = 14.2, 3.2 Hz, 1H), 3.10 (dd, *J* = 14.2, 8.9 Hz, 1H), 2.68 (ddd, *J* = 15.7, 9.2, 5.1 Hz, 1H), 2.48 (ddd, *J* = 15.7, 4.0, 3.8 Hz, 1H).

^{13}C NMR (100 MHz, acetone-*d*$_6$): δ 149.4, 148.1, 145.4, 145.21, 132.5 (2C), 130.0, 127.0, 124.4 (2C), 116.7, 113.4, 77.3, 64.4, 43.4, 29.7.

HRMS (ESI): *m/z* calcd. For $C_{16}H_{15}NO_5Na$ $[M+Na]^+$ 324.0842, found 324.0829.

***6-Methoxy-1-(4-nitrobenzyl)isochromane* (2y)**

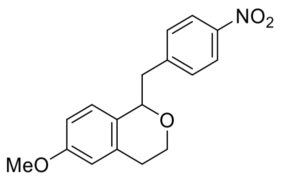

Chemical Formula: $C_{17}H_{17}NO_4$
Exact Mass: 299.12

The general procedure was followed with 2-(4-nitrophenyl)oxirane (33.0 mg, 0.2 mmol) and 2-(3-methoxyphenyl)ethanol (60.9 mg, 0.40 mmol) in the presence of TfOH (1.8 µL, 0.020 mmol, 10 mol%) in HFIP (2.0 mL). The reaction mixture was stirred at 25 °C for 1 h. Purification by FC over silica gel (*n*-pentane/EtOAc 100:0 to 85:15 gradient) afforded **2y** (42.0 mg, 70% yield) as a yellow solid.

m.p. = 66 – 68 °C.

^{1}H NMR (400 MHz, CDCl$_3$): δ 8.12 (d, *J* = 8.7 Hz, 2H), 7.40 (d, *J* = 8.7 Hz, 2H), 7.07 (d, *J* = 8.5 Hz, 1H), 6.78 (dd, *J* = 8.5, 2.7 Hz, 1H), 6.63 (d, *J* = 2.6 Hz, 1H), 5.04 – 5.01 (dd, *J* = 8.3, 3.5 Hz, 1H), 4.09 (ddd, *J* = 11.2, 5.3, 3.7 Hz, 1H), 3.80 (s, 3H), 3.71 (ddd, *J* = 11.2, 9.7, 3.7 Hz, 1H), 3.32 (dd, *J* = 14.3, 3.5 Hz, 1H), 3.11 (dd, *J* = 14.3, 8.3 Hz, 1H), 2.85 (ddd, *J* = 16.3, 9.7, 5.3 Hz, 1H), 2.62 (ddd, *J* = 16.3, 3.7, 3.7 Hz, 1H).

^{13}C NMR (100 MHz, CDCl$_3$): δ 158.3, 146.8, 146.7, 135.8, 130.6 (2C), 129.1, 126.0, 123.4 (2C), 113.6, 112.8, 75.9, 63.4, 55.4, 42.5, 29.5.

HRMS (ESI): *m/z* calcd. For $C_{17}H_{17}NO_4Na$ $[M+Na]^+$ 322.1038, found 322.1050.

***1-(4-Nitrobenzyl)isochroman-6-ol* (2z)**

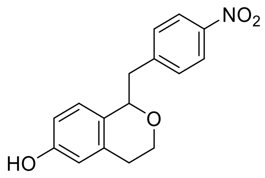

Chemical Formula: $C_{16}H_{15}NO_4$
Exact Mass: 285.10

The general procedure was followed with 2-(4-nitrophenyl)oxirane (33.0 mg, 0.2 mmol) and 3-(2-hydroxyethyl)phenol (55.2 mg, 0.40 mmol) in the presence of TfOH (1.8 µL, 0.020 mmol, 10 mol%) in HFIP (2.0 mL). The reaction mixture was stirred at 25 °C for 1 h. Purification by FC over silica gel (*n*-pentane/EtOAc 80:20 to 60:40 gradient) afforded a mixture of two regioisomers (35.4 mg, 63% yield, *p/o* 90:10). After re-crystallization from $CHCl_3$ + acetone/*n*-hexane, a pure sample of **2z** was obtained as yellow crystals.

m.p. = 154 – 156 °C.

^{1}H NMR (400 MHz, acetone-d_6): δ 8.22 (s, 1H), 8.16 (d, *J* = 8.6 Hz, 2H), 7.61 (d, *J* = 8.6 Hz, 2H), 7.20 (d, *J* = 8.3 Hz, 1H), 6.75 (d, *J* = 8.3 Hz, 1H), 6.63 (s, 1H), 5.01 (dd, *J* = 8.8, 3.3 Hz, 1H), 4.09 (ddd, *J* = 11.0, 5.2, 4.0 Hz, 1H), 3.69 (ddd, *J* = 11.0, 9.2, 4.0 Hz, 1H), 3.44 (dd, *J* = 14.2, 3.3 Hz, 1H), 3.12 (dd, *J* = 14.2, 8.8 Hz, 1H), 2.81 (ddd, *J* = 16.3, 9.2, 5.2 Hz, 1H), 2.61 (ddd, *J* = 16.3, 4.0, 4.0 Hz, 1H).

^{13}C NMR (100 MHz, acetone-d_6): δ 157.4, 149.3, 148.2, 137.2, 132.5 (2C), 129.9, 127.9, 124.4 (2C), 116.6, 115.2, 77.5, 64.2, 43.4. One CH_2 signal overlaps with the signal of the solvent.

HRMS (ESI): *m/z* calcd. For $C_{16}H_{15}NO_4Na$ $[M+Na]^+$ 308.0881, found 308.0893.

6-Methyl-1-(4-nitrobenzyl)isochromane **(2aa)**

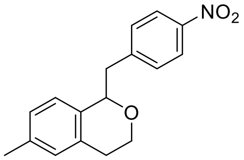

Chemical Formula: $C_{17}H_{17}NO_3$
Exact Mass: 283.12

The general procedure was followed with 2-(4-nitrophenyl)oxirane (33.0 mg, 0.2 mmol) and 2-(3-methylphenyl)ethanol (54.5 mg, 0.4 mmol) in the presence of TfOH (1.8 µL, 0.020 mmol, 10 mol%) in HFIP (2.0 mL). The reaction mixture was stirred at 25 °C for 1 h. Purification by FC over silica gel (*n*-pentane/EtOAc 100:0 to 90:10 gradient) afforded two regioisomers (44.4 mg, 78% yield, 83:17 *p*:*o*) as a yellow oil. The two regioisomers were separated by a second FC with a slower gradient.

^{1}H NMR (400 MHz, $CDCl_3$): δ 8.12 (d, *J* = 8.7 Hz, 2H), 7.41 (d, *J* = 8.7 Hz, 2H), 7.07 – 7.02 (m, 2H), 6.93 (s, 1H), 5.04 (dd, *J* = 8.5, 3.3 Hz, 1H), 4.10 (ddd, *J* = 11.2, 5.2, 3.7 Hz, 1H), 3.71 (ddd, *J* = 11.2, 9.7, 3.7 Hz, 1H), 3.33 (dd, *J* = 14.3, 3.3 Hz, 1H), 3.13 (dd, *J* = 14.3, 8.5 Hz, 1H), 2.85 (ddd, *J* = 15.4, 9.7, 5.2 Hz, 1H), 2.62 (ddd, *J* = 15.4, 3.7, 3.7 Hz, 1H), 2.32 (s, 3H).

^{13}C NMR (126 MHz, $CDCl_3$): δ 146.8, 146.7, 136.4, 134.2, 133.9, 130.6 (2C), 129.8, 127.2, 124.8, 123.4 (2C), 76.1, 63.5, 42.4, 29.1, 21.1.

HRMS (ESI): *m/z* calcd. For $C_{17}H_{17}NO_3Na$ $[M+Na]^+$ 306.1096, found 306.1101.

6-Fluoro-1-(4-nitrobenzyl)isochromane **(2ab)**

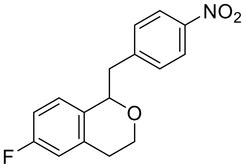

Chemical Formula: $C_{16}H_{14}FNO_3$
Exact Mass: 287.10

The general procedure was followed with 2-(4-nitrophenyl)oxirane (33.0 mg, 0.2 mmol) and 2-(3-fluorophenyl)ethan-1-ol (49.6 µL, 0.4 mmol) in the presence of TfOH (1.8 µL, 0.020 mmol, 10 mol%) in HFIP (2.0 mL). The reaction mixture was stirred at 25 °C for 1 h. Purification by FC over silica gel (*n*-pentane/EtOAc 100:0 to 90:10 gradient) afforded two regioisomers (48.7 mg, 85% yield, 96:4 *p*:*o*) as a yellow solid.

^{1}H NMR (400 MHz, CDCl$_3$): δ 8.12 (d, *J* = 8.8 Hz, 2H), 7.40 (d, *J* = 8.8 Hz, 2H), 7.13 (dd, *J* = 8.5, 5.5 (HF) Hz, 1H), 6.92 (ddd, *J* = 8.5, 8.5 (HF), 2.7 Hz, 1H), 6.80 (dd, *J* = 9.3 (HF), 2.7 Hz, 1H), 5.03 (dd, *J* = 8.4, 3.5 Hz, 1H), 4.10 (ddd, *J* = 11.4, 5.4, 3.7 Hz, 1H), 3.70 (ddd, *J* = 11.4, 9.7, 3.7 Hz, 1H), 3.33 (dd, *J* = 14.4, 3.5 Hz, 1H), 3.13 (dd, *J* = 14.3, 8.4 Hz, 1H), 2.86 (ddd, *J* = 16.4, 9.7, 5.4 Hz, 1H), 2.63 (ddd, *J* = 16.4, 3.7, 3.7 Hz, 1H).

^{13}C NMR (126 MHz, CDCl$_3$): δ 161.3 (d, *J* = 245.7 Hz), 146.6, 146.3, 136.6 (d, *J* = 7.6 Hz), 132.5 (d, *J* = 3.1 Hz), 130.5 (2C), 126.5 (d, *J* = 8.3 Hz), 123.3 (2C), 115.4 (d, *J* = 20.7 Hz), 113.5 (d, *J* = 21.6 Hz), 75.7, 63.0, 42.2, 29.1.

^{19}F NMR (471 MHz, CDCl$_3$): δ -115.9 (ddd, *J* = 9.3, 8.5, 5.3 Hz).

HRMS (ESI): m/z calcd. for $C_{16}H_{13}O_3NF$ [M+H] + 288.0932, found 288.1021.

7-Methoxy-1-(4-nitrobenzyl)isochromane **(2ad)**

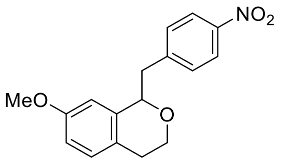

Chemical Formula: $C_{17}H_{17}NO_4$
Exact Mass: 299.12

The general procedure was followed with 2-(4-nitrophenyl)oxirane (33.0 mg, 0.2 mmol) and 2-(4-methoxyphenyl)ethan-1-ol (61 mg, 0.4 mmol) in the presence of TfOH (1.8 µL, 0.020 mmol, 10 mol%) in HFIP (2.0 mL). The reaction mixture was stirred at 25 °C for 0.25 h. Purification by FC over silica gel (*n*-pentane/EtOAc 100:0 to 90:10 gradient) afforded **2ad** (17.7 mg, 29% yield) as a yellow solid.

m.p. = 79 – 81 °C

^{1}H NMR (400 MHz, CDCl$_3$): δ 8.13 (d, *J* = 8.7 Hz, 2H), 7.41 (d, *J* = 8.7 Hz, 2H), 7.03 (d, *J* = 8.4 Hz, 1H), 6.76 (d, *J* = 11.1 Hz, 1H), 6.69 (s, 1H), 5.03 (dd, *J* = 8.5, 3.7 Hz, 1H), 4.17 – 4.03 (ddd, *J* = 11.0, 5.3, 3.9 Hz, 1H), 3.80 (s, 3H), 3.69 (ddd, *J* = 11.0, 9.7, 3.9 Hz, 1H), 3.33 (dd, *J* = 14.3, 3.7 Hz, 1H), 3.14 (dd, *J* = 14.3, 8.5 Hz, 1H), 2.80 (ddd, *J* = 15.9, 9.7, 5.3, 1H), 2.59 (d, *J* = 15.9, 3.9, 3.9 Hz, 1H).

^{13}C NMR (101 MHz, CDCl$_3$): δ 158.0, 146.6, 146.5, 137.8, 130.5 (2C), 130.0, 126.4, 123.3 (2C), 112.4, 110.3, 76.0, 63.5, 55.4, 42.2, 28.2.

HRMS (ESI): m/z calcd. for $C_{17}H_{17}O_4NNa$ [M+Na] + 322.1050, found 322.1038.

7-(Tert-butyl)-1-(4-nitrobenzyl)isochromane **(2ae)**

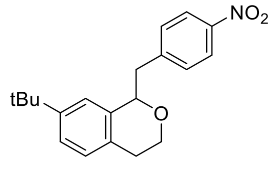

Chemical Formula: $C_{20}H_{23}NO_3$
Exact Mass: 325.17

The general procedure was followed with 2-(4-nitrophenyl)oxirane (33.0 mg, 0.2 mmol) and 2-(3-(tert-butyl)phenyl)ethan-1-ol (71.3 mg, 0.4 mmol) in the presence of TfOH (1.8 µL, 0.020 mmol, 10 mol%) in HFIP (2.0 mL). The reaction mixture was stirred at 25 °C for 1 h. Purification by FC over silica gel (*n*-pentane/EtOAc 100:0 to 95:5 gradient) afforded **2ae** (43.7 mg, 67% yield) as a colorless oil.

^{1}H NMR (500 MHz, CDCl$_3$): δ 8.13 (d, *J* = 8.9 Hz, 2H), 7.42 (d, *J* = 8.9 Hz, 2H), 7.23 (dd, *J* = 8.0, 2.4 Hz, 1H), 7.13 (d, *J* = 2.4 Hz, 1H), 7.06 (d, *J* = 8.0 Hz, 1H), 5.09 (dd, *J* = 8.5, 3.7 Hz, 1H), 4.12 (ddd, *J* = 11.3, 5.3, 3.8 Hz, 1H), 3.73 (ddd, *J* = 11.3, 9.5, 3.8 Hz, 1H), 3.36 (dd, *J* = 14.3, 3.7 Hz, 1H), 3.18 (dd, *J* = 14.3, 8.5 Hz, 1H), 2.85 (ddd, *J* = 16.0, 9.5, 5.3 Hz, 1H), 2.64 (ddd, *J* = 16.0, 3.8, 3.8 Hz, 1H), 1.32 (s, 9H).

^{13}C NMR (126 MHz, CDCl$_3$): δ 149.2, 146.7, 146.6, 136.2, 131.3, 130.5 (2C), 128.8, 123.9, 123.3 (2C), 121.5, 76.2, 63.3, 42.4, 34.6, 31.4 (3C), 28.6.

HRMS (ESI): m/z calcd. for $C_{20}H_{23}O_3NNa$ $[M+Na]^+$ 348.1570, found 348.1555.

6-Fluoro-7-methoxy-1-(4-nitrobenzyl)isochromane **(2af)**

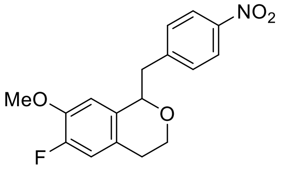

Chemical Formula: $C_{17}H_{16}FNO_4$
Exact Mass: 317.11

The general procedure was followed with 2-(4-nitrophenyl)oxirane (33.0 mg, 0.2 mmol) and 2-(3-fluoro-4-methoxyphenyl)ethan-1-ol (68.0 mg, 0.4 mmol) in the presence of TfOH (1.8 µL, 0.020 mmol, 10 mol%) in HFIP (2.0 mL). The reaction mixture was stirred at 25 °C for 1 h. Purification by FC over silica gel (*n*-pentane/EtOAc 100:0 to 75:25 gradient) afforded **2af** (37.0 mg, 59% yield) as a yellow solid.

m.p. = 129 – 131 °C

^{1}H NMR (400 MHz, CDCl$_3$): δ 8.13 (d, *J* = 8.8 Hz, 2H), 7.41 (d, *J* = 8.8 Hz, 2H), 6.81 (d, *J* = 11.6 Hz (HF), 1H), 6.69 (d, *J* = 8.5 Hz (HF), 1H), 5.00 (dd, *J* = 8.3, 3.7 Hz, 1H), 4.08 (ddd, *J* =

11.1, 5.2, 3.8 Hz, 1H), 3.86 (s, 3H), 3.68 (ddd, *J* = 11.1, 9.4, 3.8 Hz, 1H), 3.31 (dd, *J* = 14.3, 3.7 Hz, 1H), 3.13 (dd, *J* = 14.3, 8.3 Hz, 1H), 2.77 (ddd, *J* = 16.0, 9.4, 5.2 Hz, 1H), 2.55 (ddd, *J* = 16.0, 3.8, 3.8 Hz, 1H).

^{13}C NMR (101 MHz, CDCl$_3$): δ 151.3 (d, *J* = 246 Hz), 146.8, 146.4, 146.1 (d, *J* = 11.1 Hz), 132.5 (d, *J* = 3.8 Hz), 130.5 (2C), 127.2 (d, *J* = 6.5 Hz), 123.4 (2C), 116.3 (d, *J* = 17.9 Hz), 110.3 (d, *J* = 2.2 Hz), 75.7, 63.1, 56.6, 42.3, 28.2.

^{19}F NMR (471 MHz, CDCl$_3$): δ -137.3 (dd, *J* = 11.6, 8.5 Hz).

HRMS (ESI): m/z calcd. for $C_{17}H_{16}O_4NF$ $[M+Na]^+$ 340.0956, found 340.0964.

***5,8-Dimethoxy-1-(4-nitrobenzyl)isochromane* (2ag)**

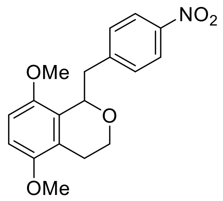

Chemical Formula: $C_{18}H_{19}NO_5$
Exact Mass: 329.13

The general procedure was followed with 2-(4-nitrophenyl)oxirane (33.0 mg, 0.2 mmol) and 2-(2,5-dimethoxyphenyl)ethan-1-ol (73.0 mg, 0.4 mmol) in the presence of TfOH (1.8 µL, 0.020 mmol, 10 mol%) in HFIP (2.0 mL). The reaction mixture was stirred at 25 °C for 0.25 h. Purification by FC over silica gel (*n*-pentane/EtOAc 100:0 to 90:10 gradient) afforded **2ag** (42.0 mg, 64% yield) as a yellow solid.

m.p. = 115 – 117 °C

^{1}H NMR (400 MHz, CDCl$_3$): δ 8.13 (d, *J* = 8.6 Hz, 2H), 7.41 (d, *J* = 8.6 Hz, 2H), 6.71 (s, 2H), 5.12 (dd, *J* = 8.8, 2.7 Hz, 1H), 4.06 (ddd, *J* = 11.8, 7.7, 4.9 Hz, 1H), 3.86 (s, 3H), 3.81 – 3.75 (m, 1H), 3.78 (s, 3H), 3.35 (dd, *J* = 14.1, 2.7 Hz, 1H), 3.15 (dd, *J* = 14.1, 8.8 Hz, 1H), 2.76 – 2.66 (m, 1H), 2.58 (ddd, *J* = 17.1, 4.9, 4.9 Hz, 1H).

^{13}C NMR (101 MHz, CDCl$_3$): δ 151.0, 149.6, 148.3, 146.6, 130.3 (2C), 126.8, 124.7, 123.4 (2C), 108.2, 107.6, 72.9, 59.9, 55.8, 55.6, 39.3, 23.2.

HRMS (ESI): m/z calcd. for $C_{18}H_{20}O_5N$ $[M+H]^+$ 330.1336, found 330.1341.

4-(4-nitrobenzyl)-1,4-dihydro-2H-benzo[f]isochromene **(2ai)**

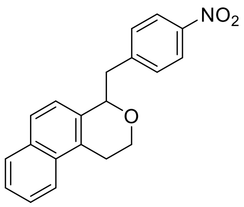

Chemical Formula: $C_{20}H_{17}NO_3$
Exact Mass: 319.12

The general procedure was followed with 2-(4-nitrophenyl)oxirane (33.0 mg, 0.2 mmol) and 2-(naphthalen-1-yl)ethan-1-ol (68.9 mg, 0.4 mmol) in the presence of TfOH (0.35 µL, 0.004 mmol, 2 mol%) in HFIP (2.0 mL). The reaction mixture was stirred at 25 °C for 16 h. Purification by FC over silica gel (*n*-pentane/EtOAc 100:0 to 90:10 gradient) afforded **2ai** (12.0 mg, 19% yield) as a light-yellow solid.

m.p. = 137 – 139 °C

^{1}H NMR (400 MHz, $CDCl_3$): δ 8.11 (d, *J* = 8.7 Hz, 2H), 7.92 (dd, *J* = 8.4, 1.3 Hz, 1H), 7.84 (dd, *J* = 7.7, 1.6 Hz, 1H), 7.74 (d, *J* = 8.6 Hz, 1H), 7.53 (m, 2H), 7.43 (d, *J* = 8.7 Hz, 2H), 7.29 (d, *J* = 8.6 Hz, 1H), 5.22 (m, 1H), 4.30 (ddd, *J* = 11.3, 4.8, 4.8 Hz, 1H), 3.88 (ddd, *J* = 11.3, 8.1, 4.8 Hz, 1H), 3.44 (dd, *J* = 14.4, 3.5 Hz, 1H), 3.21 (dd, *J* = 14.4, 8.4 Hz, 1H), 3.14 – 3.03 (m, 1H).

^{13}C NMR (101 MHz, $CDCl_3$): δ 146.8, 146.6, 133.8, 132.2, 132.0, 130.6 (2C), 130.0, 128.6, 126.7, 126.7, 125.9, 123.4 (2C), 123.0, 123.0, 76.3, 62.9, 42.1, 25.6.

HRMS (ESI): m/z calcd. for $C_{20}H_{17}O_3NNa$ $[M+Na]^+$ 342.1101, found 342.1091.

1-(4-Nitrobenzyl)-4-(perfluorophenyl)isochromane **(2aj)**

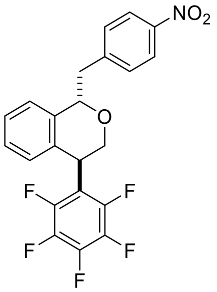

Chemical Formula: $C_{22}H_{14}F_5NO_3$
Exact Mass: 435.0894

The general procedure was followed with 2-(4-nitrophenyl)oxirane (33.0 mg, 0.2 mmol) and 2-(perfluorophenyl)-2-phenylethan-1-ol (115.2 mg, 0.4 mmol) in the presence of TfOH (1.8 µL, 0.020 mmol, 10 mol%) in HFIP (2.0 mL). The reaction mixture was stirred at 25 °C for 1 h. Purification by FC over silica gel (*n*-pentane/EtOAc 100:0 to 92:8 gradient) afforded **2aj** as two diastereoisomers (58.3 mg, 67% yield, 85:15 *dr*) as a yellow solid.

^{1}H NMR (400 MHz, $CDCl_3$, major product): δ 8.14 (d, *J* = 8.8 Hz, 2H), 7.44 (d, *J* = 8.8 Hz, 2H), 7.32 – 7.27 (m, 2H), 7.17 (m, 1H), 6.79 (m, 1H), 5.23 (dd, *J* = 8.5, 3.3 Hz, 1H), 4.66 (dd, *J* = 10.3, 5.6 Hz, 1H), 4.12 (dd, *J* = 11.0, 5.6 Hz, 1H), 3.89 (dd, *J* = 11.0, 10.3 Hz, 1H), 3.48 (dd, *J* = 14.3, 3.3 Hz, 1H), 3.17 (dd, *J* = 14.3, 8.5 Hz, 1H).

^{13}C NMR (100 MHz, $CDCl_3$, major product): δ 146.8, 146.1, 145.7 (dm, *J* = 247 Hz), 140.5 (dm, *J* = 252 Hz, 2C), 137.9 (dm, *J* = 251 Hz, 2C), 136.3, 134.7, 130.7 (2C), 127.5, 127.3, 127.1, 124.8, 123.4 (2C), 114.0 (m), 77.0, 66.9, 42.0, 34.9.

^{19}F NMR (471 MHz, $CDCl_3$, major product): δ -140.5 – -141.0 (m), -154.9 (t, *J* = 20.8 Hz), -161.3 (td, *J* = 20.8, 8.1 Hz).

HRMS (ESI): m/z calcd. for $C_{22}H_{15}O_3NF_5$ $[M+H]^+$ 436.0872, found 436.0956.

4-((4-(Perfluorophenyl)isochroman-1-yl)methyl)benzonitrile (2ak)

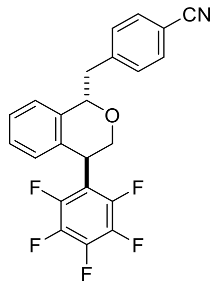

Chemical Formula: $C_{23}H_{14}F_5NO$
Exact Mass: 415.0996

The general procedure was followed with 2-(4-cyanophenyl)oxirane (29.0 mg, 0.2 mmol) and 2-(perfluorophenyl)-2-phenylethanol (115.3 mg, 0.4 mmol) in the presence of TfOH (1.8 μL, 0.020 mmol, 10 mol%) in HFIP (2.0 mL). The reaction mixture was stirred at 25 °C for 1 h. Purification by FC over silica gel (*n*-pentane/EtOAc 100:0 to 95:5 gradient) afforded **2ak** as two diastereoisomers (50.9 mg, 72% yield, 85:15 *dr*) as a white solid. The two diastereoisomers were separated by a second FC with a slower gradient.

m.p. = 125 − 127 °C

^{1}H NMR (400 MHz, $CDCl_3$, major product): δ 7.58 (d, *J* = 8.1 Hz, 2H), 7.39 (d, *J* = 8.1 Hz, 2H), 7.28 – 7.25 (m, 2H), 7.18 – 7.14 (m, 1H), 6.79 (d, *J* = 7.7 Hz, 1H), 5.20 (dd, *J* = 8.6, 3.3 Hz, 1H), 4.66 (dd, *J* = 10.2, 5.6 Hz, 1H), 4.12 (dd, *J* = 11.0, 5.6 Hz, 1H), 3.89 (dd, *J* = 11.0, 10.2 Hz, 1H), 3.43 (dd, *J* = 14.4, 3.3 Hz, 1H), 3.12 (dd, *J* = 14.4, 8.6 Hz, 1H).

^{13}C NMR (100 MHz, $CDCl_3$, major product): δ 145.7 (dm, *J* = 249 Hz), 143.9, 140.5 (dm, *J* = 254 Hz, 2C), 137.9 (dm, *J* = 252 Hz, 2C), 136.4, 134.7, 132.1 (2C), 130.6 (2C), 127.4, 127.3, 127.1, 124.9, 119.2, 114.1 (m), 110.4, 77.0, 66.9, 42.4, 34.9 (d, *J* = 1 Hz).

^{19}F NMR (377 MHz, $CDCl_3$, major product): δ -140.8 (brs), -154.9 (t, *J* = 21.0 Hz), -161.4 (td, *J* = 21.0, 8.0 Hz).

HRMS (ESI): *m/z* calcd. For $C_{23}H_{14}NOF_5Na$ $[M+Na]^+$ 438.0876, found 438.0888.

1-(4-Nitrobenzyl)-4-(4-nitrophenyl)isochromane **(2al)**

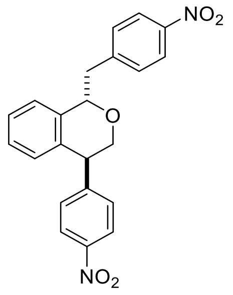

Chemical Formula: $C_{22}H_{18}N_2O_5$
Exact Mass: 390.1216

The general procedure was followed with 2-(4-nitrophenyl)oxirane (33.0 mg, 0.2 mmol) and 2-(4-nitrophenyl)-2-phenylethan-1-ol (97.2 mg, 0.4 mmol) in the presence of TfOH (1.8 µL, 0.020 mmol, 10 mol%) in HFIP (2.0 mL). The reaction mixture was stirred at 25 °C for 1 h. Purification by FC over silica gel (*n*-pentane/EtOAc 100:0 to 95:5 gradient) afforded **2al** as two diastereoisomers (67.9 mg, 87% yield, 80:20 *dr*) as a yellow solid. The two diastereoisomers were separated by a second FC with a slower gradient.

m.p. = 158 – 160 °C

^{1}H NMR (400 MHz, $CDCl_3$, major product): δ 8.15 (d, *J* = 8.7 Hz, 4H), 7.43 (d, *J* = 8.7 Hz, 2H), 7.33 – 7.26 (m, 4H), 7.16 (ddd, *J* = 7.2, 7.2, 1.6 Hz, 1H), 6.82 (d, *J* = 7.2 Hz, 1H), 5.25 (dd, *J* = 8.5, 3.4 Hz, 1H), 4.26 – 4.19 (m, 2H), 3.74 (dd, *J* = 12.6, 9.1 Hz, 1H), 3.40 (dd, *J* = 14.4, 3.4 Hz, 1H), 3.25 (dd, *J* = 14.4, 8.5 Hz, 1H).

^{13}C NMR (100 MHz, $CDCl_3$, major product): δ 149.7, 147.2, 146.9, 146.2, 136.7, 136.1, 130.6 (2C), 130.0 (2C), 129.5, 127.5, 127.4, 125.1, 124.0 (2C), 123.5 (2C), 76.6, 68.9, 45.0, 42.0.

HRMS (ESI): m/z calcd. for $C_{22}H_{19}O_5N_2$ $[M+H]^+$ 391.1299, found 391.1302.

5,8-Dimethyl-1-(4-nitrobenzyl)-4-(perfluorophenyl)isochromane **(2am)**

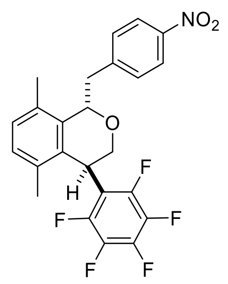

Chemical Formula: $C_{24}H_{18}F_5NO_3$
Exact Mass: 463.1207

The general procedure was followed with 2-(4-nitrophenyl)oxirane (33.0 mg, 0.2 mmol) and 2-(2,5-dimethylphenyl)-2-(perfluorophenyl)ethan-1-ol (126.4 mg, 0.4 mmol) in the presence of TfOH (1.8 µL, 0.020 mmol, 10 mol%) in HFIP (2.0 mL). The reaction mixture was stirred at 25 °C for 1 h. Purification by FC over silica gel (*n*-pentane/EtOAc 100:0 to 90:10 gradient) afforded **2am** as two diastereoisomers (51.0 mg, 55% yield, 80:20 *dr*) as a yellow oil.

^{1}H NMR (400 MHz, CDCl$_3$, major product): δ 8.20 (d, *J* = 8.8 Hz, 2H), 7.51 (d, *J* = 8.8 Hz, 2H), 7.11 (d, *J* = 7.8 Hz, 1H), 7.03 (d, *J* = 7.8 Hz, 1H), 5.23 (dd, *J* = 10.5, 2.4 Hz, 1H), 4.54 (dd, *J* = 12.0, 3.9 Hz, 1H), 4.40 (d, *J* = 3.9 Hz, 1H), 3.91 (d, *J* = 12.0 Hz, 1H), 3.32 (dd, *J* = 15.3, 10.5 Hz, 1H), 3.14 (dd, *J* = 15.3, 2.4 Hz, 1H), 2.42 (s, 3H), 2.06 (s, 3H).

^{13}C NMR (126 MHz, CDCl$_3$, major product): δ 147.3, 146.9, 145.6 (dm, *J* = 247.1 Hz), 140.1 (dm, *J* = 253.0 Hz, 2C), 137.8 (dm, *J* = 260.2 Hz, 2C), 135.9, 133.9, 131.0, 130.5, 130.3, 129.9 (2C), 129.2, 123.8 (2C), 116.7 (td; *J* = 15.1, 3.9 Hz), 74.9, 63.5, 37.6, 32.8, 19.5, 18.6.

^{19}F NMR (471 MHz, CDCl$_3$, major product) δ -139.98, -143.90, -156.17 (dd, *J* = 21.0, 21.0 Hz), -161.56, -162.57.

***4-Methyl-1-(4-nitrobenzyl)isochromane* (2an)**

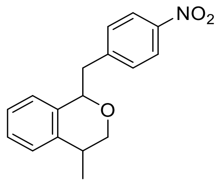

Chemical Formula: $C_{17}H_{17}NO_3$
Exact Mass: 283.12

The general procedure was followed with 2-(4-nitrophenyl)oxirane (33.0 mg, 0.2 mmol) and 2-methyl-2-phenylethanol (54.5 mg, 0.40 mmol) in the presence of TfOH (1.8 µL, 0.020 mmol, 10 mol%) in HFIP (2.0 mL). The reaction mixture was stirred at 25 °C for 0.25 h. Purification by FC over silica gel (*n*-pentane/EtOAc 100:0 to 85:15 gradient) afforded **2an** as two diastereoisomers (29.0 mg, 51% yield, 57:43 *dr*) as a yellow oil.

^{1}H NMR (400 MHz, CDCl$_3$): δ 8.15 – 8.09 (m, 2H, major + minor), 7.42 – 7.38 (m, 2H, major + minor), 7.26 – 7.14 (m, 4H, major + minor), 5.11 (dd, *J* = 7.9, 3.5 Hz, 1H, major), 5.05 (dd, *J* = 7.9, 3.6 Hz, 1H, minor), 4.05 (dd, *J* = 11.2, 4.7 Hz, 1H, major), 3.81 (d, *J* = 3.0 Hz, 2H, minor), 3.43 (dd, *J* = 11.2, 7.8 Hz, 1H, major), 3.37 (dd, *J* = 14.3, 3.6 Hz, 1H, minor), 3.31 (dd, *J* = 14.3, 3.5 Hz, 1H, major), 3.18 (dd, 14.3, 7.9 Hz, 1H, major + minor), 2.94 – 2.85 (m, 1H, major), 2.78 – 2.71 (m, 1H, minor), 1.22 (d, *J* = 7.0 Hz, 3H, major), 1.17 (d, *J* = 7.1 Hz, 3H, minor).

^{13}C NMR (100 MHz, CDCl$_3$): δ 146.8 (2C, major + minor ; 1C, major), 146.6 (minor),140.0 (minor), 139.7 (major), 136.3 (major), 136.2 (minor), 130.8 (2C, minor), 130.6 (2C, major), 128.8 (minor), 127.3 (major), 127.1 (major), 127.0 (minor), 126.3 (minor), 126.2 (major), 124.9 (major), 124.8 (minor), 123.4 (2C, major), 123.3 (2C, minor), 76.5 (major), 76.3 (minor), 69.3 (minor), 69.0 (major), 42.3 (major), 42.3 (minor), 33.0 (minor), 32.1 (major), 21.0 (minor), 17.5 (major).

HRMS (ESI): *m/z* calcd. For $C_{17}H_{17}NO_3Na$ $[M+Na]^+$ 306.1101, found 306.1083.

1-(4-Nitrobenzyl)-3a,4,5,6-tetrahydro-1H,3H-benzo[de]isochromene **(2ao)**

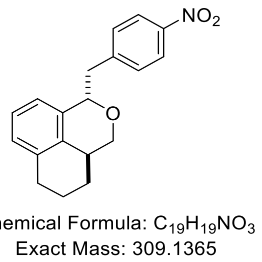

The general procedure was followed with 2-(4-nitrophenyl)oxirane (33.0 mg, 0.2 mmol) and (1,2,3,4-tetrahydronaphthalen-1-yl)methanol (64.9 mg, 0.40 mmol) in the presence of TfOH (1.8 µL, 0.020 mmol, 10 mol%) in HFIP (2.0 mL). The reaction mixture was stirred at 25 °C for 0.25 h. Purification by FC over silica gel (*n*-pentane/EtOAc 100:0 to 93:7 gradient) afforded **2ao** as two diastereoisomers (37.1 mg, 60% yield, 85:15 *dr*) as a yellow solid. The two diastereoisomers were separated by a second FC with a slower gradient. The compound was recrystallized from DCM/hexane.

m.p. = 142 − 144 °C.

^{1}H NMR (400 MHz, $CDCl_3$): δ 8.09 (d, *J* = 8.7 Hz, 2H), 7.36 (d, *J* = 8.7 Hz, 2H), 7.15 (dd, *J* = 7.6, 7.6 Hz, 1H), 7.02 – 6.94 (m, 2H), 5.17 (dd, *J* = 7.4, 3.7 Hz, 1H), 3.99 (dd, *J* =10.5 Hz, 4.7 Hz, 1H), 3.36 (dd, *J* = 14.1, 3.7 Hz, 1H), 3.30 (dd, *J* = 11.1, 10.5 Hz, 1H), 3.14 (dd, *J* = 14.1, 7.4 Hz, 1H), 2.87 (dd, *J* = 16.2, 5.6 Hz, 1H), 2.77 (ddd, *J* = 16.2, 11.5, 6.8 Hz, 1H), 2.65 (m, 1H), 2.00 – 1.94 (m, 1H), 1.81-1.66 (m, 2H), 1.08 (m, 1H).

^{13}C NMR (100 MHz, $CDCl_3$): δ 146.7, 146.6, 136.0, 135.5, 135.4, 130.8 (2C), 127.3, 126.1, 123.2 (2C), 122.1, 77.1, 69.7, 43.7, 35.7, 28.8, 24.5, 22.0.

HRMS (ESI): *m/z* calcd. For $C_{19}H_{19}NO_3Na$ $[M+Na]^+$ 332.1257, found 332.1248.

4,4-Dimethyl-1-(4-nitrobenzyl)isochromane **(2ap)**

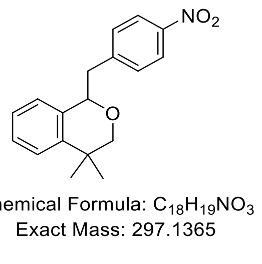

The general procedure was followed with 2-(4-nitrophenyl)oxirane (33.0 mg, 0.2 mmol) and 2,2-dimethyl-2-phenylethanol (60.1 mg, 0.40 mmol) in the presence of TfOH (1.8 µL, 0.020 mmol, 10 mol%) in HFIP (2.0 mL). The reaction mixture was stirred at 25 °C for 0.25 h. Purification by

FC over silica gel (*n*-pentane/EtOAc 100:0 to 85:15 gradient) afforded **2ap** (42.0 mg, 71% yield) as a yellow oil.

^{1}H NMR (400 MHz, CDCl$_3$): δ 8.12 (d, *J* = 8.7 Hz, 2H), 7.41 (d, *J* = 8.7 Hz, 2H), 7.33 (dd, *J* = 7.4, 1.8 Hz, 1H), 7.26 – 7.16 (m, 3H), 5.10 (dd, *J* = 8.1, 3.6 Hz, 1H), 3.67 (d, *J* = 11.1 Hz, 1H), 3.49 (d, *J* = 11.1 Hz, 1H), 3.35 (dd, *J* = 14.3, 3.6 Hz, 1H), 3.22 (dd, *J* = 14.3, 8.1 Hz, 1H), 1.20 (s, 3H), 1.17 (s, 3H).

^{13}C NMR (100 MHz, CDCl$_3$): δ 146.7, 146.6, 143.9, 135.4, 130.7 (2C), 127.2, 125.9, 125.9, 124.7, 123.3 (2C), 76.8, 74.4, 42.1, 33.8, 28.5, 25.9.

HRMS (ESI): *m/z* calcd. For $C_{18}H_{19}NO_3Na$ $[M+Na]^+$ 320.1245, found 320.1257.

3-Methyl-1-(4-nitrobenzyl)isochromane **(2aq)**

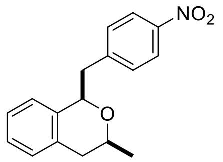

The general procedure was followed with 2-(4-nitrophenyl)oxirane (33.0 mg, 0.2 mmol) and 1-phenylpropan-2-ol (56.0 µL, 0.4 mmol) in the presence of TfOH (1.8 µL, 0.020 mmol, 10 mol%) in HFIP (2.0 mL). The reaction mixture was stirred at 25 °C for 1 h. Purification by FC over silica gel (*n*-pentane/EtOAc 100:0 to 90:10 gradient) afforded **2aq** as a single diastereoisomer (27.0 mg, 47% yield) as a yellow oil. 60% NMR yield with a ratio of 80/20 were determined by ^{1}H NMR using mesitylene as an external standard.

^{1}H NMR (400 MHz, CDCl$_3$): δ 8.08 (d, *J* = 8.7 Hz, 2H), 7.39 (d, *J* = 8.7 Hz, 2H), 7.24 – 7.13 (m, 3H), 7.04 (d, *J* = 7.3 Hz, 1H), 5.11 (dd, *J* = 7.3, 3.5 Hz, 1H), 3.74 (m, 1H), 3.41 (dd, *J* = 14.2, 3.5 Hz, 1H), 3.12 (dd, *J* = 14.2, 7.3 Hz, 1H), 2.55 (m, 2H), 1.29 (d, *J* = 6.1 Hz, 3H).

^{13}C NMR (101 MHz, CDCl$_3$): δ 146.6, 146.6, 136.6, 134.9, 130.9 (2C), 129.0, 126.7, 126.3, 124.5, 123.0 (2C), 76.5, 70.4, 42.5, 36.7, 21.8.

HRMS (ESI): m/z calcd. for $C_{17}H_{17}O_3NNa$ $[M+Na]^+$ 306.1101, found 306.1108.

5,8-dimethoxy-3-methyl-1-(4-nitrobenzyl)isochromane **(2ar)**

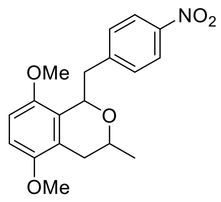

Chemical Formula: $C_{19}H_{21}NO_5$
Exact Mass: 343.14

The general procedure was followed with 2-(4-nitrophenyl)oxirane (47.3 mg, 0.285 mmol) and 1-(2,5-dimethoxyphenyl)propan-2-ol (112.0 mg, 0.57 mmol) in the presence of TfOH (2.5 µL, 0.029 mmol, 10 mol%) in HFIP (2.9 mL). The reaction mixture was stirred at 25 °C for 1 h. Purification by FC over silica gel (*n*-pentane/EtOAc 100:0 to 70:30 gradient) afforded **2ar** as two isolated diastereoisomers (*cis*: 41.7 mg, 43% yield and *trans*: 34.0 mg, 35% yield) as yellow solids.

m.p. (*cis*) = 96 – 98 °C

^{1}H NMR (400 MHz, $CDCl_3$, *cis* diastereoisomer): δ 8.00 (d, *J* = 8.7 Hz, 2H), 7.25 (d, *J* = 8.7 Hz, 2H), 6.70 – 6.61 (m, 2H), 5.21 (m, 1H), 3.85 (s, 3H), 3.72 (s, 3H), 3.55 (dqd, *J* = 12.1, 6.1, 2.2 Hz, 1H), 3.40 (dd, *J* = 13.6, 3.2 Hz, 1H), 3.23 (d, *J* = 13.6, 5.9 Hz, 1H), 2.68 (ddd, *J* = 16.4, 2.2, 1.3 Hz, 1H), 1.89 (m, 1H), 1.28 (d, *J* = 6.1 Hz, 3H).

^{13}C NMR (101 MHz, $CDCl_3$, *cis* diastereoisomer): δ 150.6, 149.9, 147.8, 146.5, 130.9 (2C), 126.3, 126.1, 122.8 (2C), 108.3, 107.7, 74.1, 69.0, 55.8, 55.5, 41.1, 30.9, 21.7.

HRMS (ESI *cis*): m/z calcd. for $C_{19}H_{21}O_5NNa$ $[M+Na]^+$ 366.1312, found 366.1306.

m.p. (*trans*) = 124 – 126 °C

^{1}H NMR (400 MHz, $CDCl_3$, *trans* diastereoisomer): δ 8.19 (d, *J* = 8.7 Hz, 2H), 7.46 (d, *J* = 8.7 Hz, 2H), 6.77 – 6.66 (m, 2H), 5.10 (dd, *J* = 10.2, 2.6 Hz, 1H), 4.15 (dqd, *J* = 12.2, 6.1, 3.4 Hz, 1H), 3.86 (s, 3H), 3.80 (s, 3H), 3.29 (dd, *J* = 14.2, 2.6 Hz, 1H), 3.12 (dd, *J* = 14.2, 10.2 Hz, 1H), 2.83 (dd, *J* = 17.2, 3.4 Hz, 1H), 2.34 (dd, *J* = 17.2, 12.2 Hz, 1H), 1.31 (d, *J* = 6.1 Hz, 3H).

^{13}C NMR (101 MHz, $CDCl_3$, trans diastereoisomer): δ 151.0, 149.4, 148.6, 146.6, 130.2 (2C), 126.8, 123.9, 123.6 (2C), 108.2, 107.5, 73.5, 62.8, 55.8, 55.7, 38.5, 30.3, 21.7.

HRMS (ESI trans): m/z calcd. for $C_{19}H_{21}O_5NNa$ $[M+Na]^+$ 366.1312, found 366.1304.

(4aR,6S,10bR)-6-(4-Nitrobenzyl)-2,3,4,4a,6,10b-hexahydro-1H-benzo[c]chromene **(2as)**

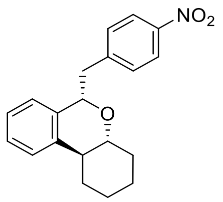

Chemical Formula: $C_{20}H_{21}NO_3$
Exact Mass: 323.15

The general procedure was followed with 2-(4-nitrophenyl)oxirane (33.0 mg, 0.2 mmol) and (1*S*,2*S*)-2-phenylcyclohexan-1-ol (70.4 mg, 0.4 mmol) in the presence of TfOH (1.8 µL, 0.020 mmol, 10 mol%) in HFIP (2.0 mL). The reaction mixture was stirred at 25 °C for 1 h. Purification by FC over silica gel (*n*-pentane/EtOAc 100:0 to 90:10 gradient) afforded a mixture of diastereoisomers (27.6 mg, 69% yield, 80:20 *dr*) as a white solid. The two diastereoisomers were separated by a second FC with toluene as eluent.

m.p. = 94 – 97 °C

^{1}H NMR (400 MHz, CDCl$_3$): δ 8.04 (d, *J* = 8.7 Hz, 2H), 7.31 (d, *J* = 8.7 Hz, 2H), 7.23 – 7.12 (m, 4H), 5.23 (dd, *J* = 6.6, 3.7 Hz, 1H), 3.37 (dd, *J* = 14.1, 3.7 Hz, 1H), 3.20 (m, 1H), 3.14 (dd, *J* = 14.1, 6.6 Hz, 1H), 2.36 (m, 1H), 2.24 – 2.16 (m, 1H), 2.05 – 1.95 (m, 1H), 1.82 (m, 2H), 1.47 – 1.21 (m, 3H), 1.07 (m, 1H).

^{13}C NMR (101 MHz, CDCl$_3$): δ 146.6, 146.5, 138.9, 136.6, 131.0 (2C), 126.8, 126.1, 125.0, 124.7, 123.0 (2C), 77.7, 76.5, 43.3, 41.8, 32.7, 28.3, 25.7, 24.6.

HRMS (ESI): m/z calcd. for $C_{20}H_{21}O_3NNa$ $[M+Na]^+$ 346.1414, found 346.1422.

1-Benzhydryl-6,7-dimethoxyisochromane **(2au)**

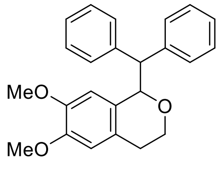

Chemical Formula: $C_{24}H_{24}O_3$
Exact Mass: 360.17

The general procedure was followed with (2*S*,3*R*)-2,3-diphenyloxirane (39.3 mg, 0.2 mmol) and 2-(3,4-dimethoxyphenyl)ethan-1-ol (73.0 mg, 0.4 mmol) in the presence of TfOH (0.4 µL, 0.004 mmol, 2 mol%) in HFIP (2.0 mL). The reaction mixture was stirred at 25 °C for 1 h. Purification by FC over silica gel (*n*-pentane/EtOAc 100:0 to 90:10 gradient) afforded **2au** (67.0 mg, 93% yield) as a colorless oil. Spectral data are in accordance with those found in the literature.[4]

^{1}H NMR (400 MHz, CDCl$_3$): δ 7.37 – 7.33 (m, 2H), 7.31 – 7.25 (m, 4H), 7.23 – 7.16 (m, 3H), 7.15 – 7.10 (m, 1H), 6.51 (s, 1H), 6.17 (s, 1H), 5.48 (d, *J* = 6.2 Hz, 1H), 4.47 (d, *J* = 6.2 Hz, 1H),

4.05 (ddd, J = 11.2, 5.0, 4.6 Hz, 1H), 3.81 (s, 3H), 3.66 (ddd, J = 11.3, 8.2, 4.6 Hz, 1H), 3.54 (s, 3H), 2.62 (ddd, J = 15.9, 8.2, 5.0 Hz, 1H), 2.53 (ddd, J = 15.9, 4.6, 4.6 Hz, 1H).

^{13}C NMR (126 MHz, CDCl$_3$): δ 147.4, 146.6, 142.9, 141.3, 129.5 (2C), 129.4 (2C), 128.5, 128.4 (2C), 128.0 (2C), 126.9, 126.5, 126.2, 111.2, 109.1, 77.5, 62.7, 57.3, 55.8, 55.6, 28.5.

HRMS (ESI): m/z calcd. for $C_{24}H_{25}O_3$ $[M+H]^+$ 361.1798, found 361.1790.

2-(4,5-Dimethoxy-2-(2-methyl-1,2,3,4-tetrahydronaphthalen-1-yl)phenyl)ethan-1-ol **(2av)**

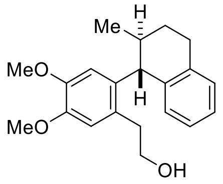

Chemical Formula: $C_{21}H_{26}O_3$
Exact Mass: 326.19

The general procedure was followed with 2-methyl-2-phenethyloxirane (32.4 mg, 0.2 mmol) and 2-(3,4-dimethoxyphenyl)ethan-1-ol (73.0 mg, 0.4 mmol) in the presence of TfOH (0.4 µL, 0.004 mmol, 2 mol%) in HFIP (2.0 mL). The reaction mixture was stirred at 25 °C for 1 h. The 95:5 *dr* was determined by NMR and purification by FC over silica gel (*n*-pentane/EtOAc 90:10 to 70:30 gradient) afforded **2av** (45.0 mg, 70% yield) as a colorless oil.

^{1}H NMR (400 MHz, CDCl$_3$): δ 7.14 – 7.03 (m, 2H), 6.97 (m, 1H), 6.75 (s, 1H), 6.62 (m, 1H), 6.42 (s, 1H), 3.88 (s, 3H), 3.87 – 3.77 (m, 3H), 3.69 (s, 3H), 3.01 (m, 2H), 2.91 (ddd, J = 16.8, 5.6, 3.3 Hz, 1H), 2.86 – 2.76 (m, 1H), 2.07 – 1.93 (m, 2H), 1.70 – 1.55 (m, 2H), 0.92 (d, J = 6.4 Hz, 3H).

^{13}C NMR (101 MHz, CDCl$_3$): δ 147.9, 147.1, 140.4, 136.9, 136.5, 129.6, 129.3, 128.7, 125.8, 125.6, 112.8, 112.5, 63.6, 56.0, 55.9, 49.0, 37.2, 36.1, 31.3, 29.5, 20.5.

HRMS (ESI): m/z calcd. for $C_{21}H_{25}O_2$ $[M+H]^+$ - H_2O 309.1849, found 309.1861.

1-(4-Nitrobenzyl)-2-tosyl-1,2,3,4-tetrahydroisoquinoline **(3a)**

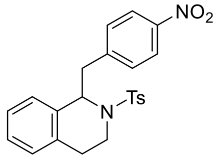

Chemical Formula: $C_{23}H_{22}N_2O_4S$
Exact Mass: 422.13

The general procedure was followed with 2-(4-nitrophenyl)oxirane (33.0 mg, 0.2 mmol) and 4-methyl-N-phenethylbenzenesulfonamide (110.0 mg, 0.4 mmol) in the presence of TfOH (1.8 µL, 0.020 mmol, 10 mol%) in HFIP (2.0 mL). The reaction mixture was stirred at 25 °C for 1 h.

Purification by FC over silica gel (*n*-pentane/DCM 20:80 to 10:90 gradient) afforded **3a** (68.0 mg, 81% yield) as a white solid.

m.p. = 145 – 147 °C

^{1}H NMR (400 MHz, CDCl$_3$): δ 8.04 (d, *J* = 8.7 Hz, 2H), 7.49 (d, *J* = 8.3 Hz, 2H), 7.18 (d, *J* = 8.7 Hz, 2H), 7.15 – 7.07 (m, 4H), 7.00 – 6.88 (m, 2H), 5.20 (m, 1H), 3.60 (ddd, *J* = 13.4, 5.8, 4.7 Hz, 1H), 3.46 (ddd, *J* = 13.4, 9.7, 4.7 Hz, 1H), 3.25 (m, 2H), 2.65 (ddd, *J* = 15.8, 9.7, 5.8 Hz, 1H), 2.46 (ddd, *J* = 15.8, 4.7, 4.7 Hz, 1H), 2.32 (s, 3H).

^{13}C NMR (101 MHz, CDCl$_3$): δ 146.9, 145.5, 143.5, 136.7, 134.9, 133.7, 130.8 (2C), 129.6 (2C), 129.0, 127.4, 127.1 (2C), 127.0, 126.4, 123.4 (2C), 57.9, 44.4, 40.3, 27.2, 21.5.

HRMS (ESI): m/z calcd. for $C_{23}H_{23}O_4N_2S$ $[M+H]^+$ 423.1373, found 423.1382.

1-benzyl-2-tosyl-1,2,3,4-tetrahydroisoquinoline **(3b)**

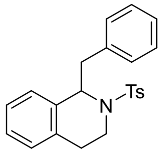

Chemical Formula: $C_{23}H_{23}NO_2S$
Exact Mass: 377.14

The general procedure was followed with 2-phenyloxirane (22.8 µL, 0.2 mmol) and 4-methyl-N-phenethylbenzenesulfonamide (110.0 mg, 0.4 mmol) in the presence of TfOH (1.8 µL, 0.020 mmol, 10 mol%) in HFIP (2.0 mL). The reaction mixture was stirred at 25 °C for 1 h. Purification by FC over silica gel (*n*-pentane/EA 100:0 to 90:10 gradient) afforded **3b** (60.0 mg, 79% yield) as a white solid.

m.p. = 128 – 131 °C

^{1}H NMR (400 MHz, CDCl$_3$): δ 7.49 (d, *J* = 8.4 Hz, 2H), 7.26 – 7.20 (m, 3H), 7.15 – 7.02 (m, 6H), 7.00 – 6.95 (m, 1H), 6.84 (dd, *J* = 7.4, 1.6 Hz, 1H), 5.24 (dd, *J* = 6.6, 6.6 Hz, 1H), 3.57 (ddd, *J* = 13.4, 6.0, 4.6 Hz, 1H), 3.43 (ddd, *J* = 13.4, 9.8, 4.6 Hz, 1H), 3.24 – 3.06 (m, 2H), 2.71 (ddd, *J* = 16.4, 9.8, 6.0 Hz, 1H), 2.49 (ddd, *J* = 16.4, 4.6, 4.6 Hz, 1H), 2.34 (s, 3H).

^{13}C NMR (101 MHz, CDCl$_3$): δ 143.1, 137.7, 137.1, 135.7, 133.7, 130.0 (2C), 129.5 (2C), 128.8, 128.3 (2C), 127.3, 127.2 (2C), 127.0, 126.6, 126.0, 58.0, 44.6, 40.0, 27.3, 21.5.

HRMS (ESI): m/z calcd. for $C_{23}H_{24}O_2NS$ $[M+H]^+$ 378.1522, found 378.1514.

1-phenethyl-2-tosyl-1,2,3,4-tetrahydroisoquinoline **(3c)**

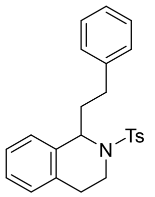

Chemical Formula: $C_{24}H_{25}NO_2S$
Exact Mass: 391.16

The general procedure was followed with 2-benzyloxirane (26.3 µL, 0.2 mmol) and 4-methyl-N-phenethylbenzenesulfonamide (110.0 mg, 0.4 mmol) in the presence of TfOH (1.8 µL, 0.020 mmol, 10 mol%) in HFIP (2.0 mL). The reaction mixture was stirred at 25 °C for 1 h. Purification by FC over silica gel (*n*-pentane/EA 100:0 to 90:10 gradient) afforded **3c** (65.0 mg, 83% yield) as a colorless oil.

^{1}H NMR (400 MHz, $CDCl_3$): δ 7.59 (d, *J* = 8.3 Hz, 2H), 7.29 – 7.12 (m, 5H), 7.12 – 6.97 (m, 5H), 6.84 (dd, *J* = 7.5, 1.4 Hz, 1H), 5.04 (dd, *J* = 9.5, 4.8 Hz, 1H), 3.94 – 3.84 (m, 1H), 3.56 – 3.43 (m, 1H), 2.90 – 2.73 (m, 2H), 2.55 – 2.47 (m, 2H), 2.29 (s, 3H), 2.18 – 1.96 (m, 2H).

^{13}C NMR (101 MHz, $CDCl_3$): δ 143.1, 141.8, 137.9, 136.5, 132.7, 129.4 (2C), 128.9, 128.5 (2C), 128.4 (2C), 127.1 (2C), 126.9, 126.7, 126.2, 125.9, 56.7, 39.4, 39.0, 32.9, 26.2, 21.5.

HRMS (ESI): m/z calcd. for $C_{24}H_{26}O_2NS$ $[M+H]^+$ 392.1679, found 392.1669.

1-benzhydryl-2-tosyl-1,2,3,4-tetrahydroisoquinoline **(3d)**

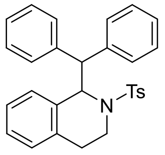

Chemical Formula: $C_{29}H_{27}NO_2S$
Exact Mass: 453.18

The general procedure was followed with (2*S*,3*R*)-2,3-diphenyloxirane (39.3 mg, 0.2 mmol) and 4-methyl-N-phenethylbenzenesulfonamide (110.0 mg, 0.4 mmol) in the presence of TfOH (1.8 µL, 0.020 mmol, 10 mol%) in HFIP (2.0 mL). The reaction mixture was stirred at 25 °C for 16 h. Purification by FC over silica gel (*n*-pentane/EA 100:0 to 90:10 gradient) afforded **3c** (81.0 mg, 89% yield) as a white solid.

m.p. = 74 – 78 °C

^{1}H NMR (400 MHz, CD_2Cl_2): δ 7.60 – 7.54 (m, 2H), 7.40 – 7.34 (m, 2H), 7.33 – 7.24 (m, 3H), 7.24 – 7.11 (m, 5H), 6.98 – 6.90 (m, 3H), 6.74 (d, *J* = 7.5 Hz, 1H), 6.71 – 6.64 (m, 1H), 6.15 (d, *J* = 7.5 Hz, 1H), 5.75 (d, *J* = 10.5 Hz, 1H), 4.22 (d, *J* = 10.5 Hz, 1H), 3.65 – 3.56 (m, 1H), 3.41 (ddd, *J* = 15.1, 10.1, 7.3 Hz, 1H), 2.66 – 2.48 (m, 2H), 2.25 (s, 3H).

^{13}C NMR (101 MHz, CD_2Cl_2): δ 143.5, 141.9, 141.7, 137.3, 135.0, 133.1, 129.6 (2C), 129.4 (2C), 129.3, 129.0 (2C), 128.9 (2C), 128.8 (2C), 128.7, 127.5 (2C), 127.3, 127.2, 127.1, 124.9, 60.0, 58.8, 38.6, 25.2, 21.5.

HRMS (ESI): m/z calcd. for $C_{29}H_{28}O_2NS$ $[M+H]^+$ 454.1835, found 454.1825.

1-(4-nitrobenzyl)-2-((4-nitrophenyl)sulfonyl)-1,2,3,4-tetrahydroisoquinoline **(3e)**

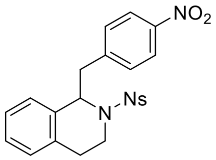

Chemical Formula: $C_{22}H_{19}N_3O_6S$
Exact Mass: 453.10

The general procedure was followed with 2-(4-nitrophenyl)oxirane (66.0 mg, 0.4 mmol) and 4-nitro-N-phenethylbenzenesulfonamide (61.1 mg, 0.2 mmol) in the presence of TfOH (5.4 µL, 0.060 mmol, 30 mol%) in HFIP (2.0 mL). The reaction mixture was stirred at 60 °C for 72 h. Purification by FC over silica gel (toluene/EA 100:0 to 95:5 gradient) afforded **3e** (60.0 mg, 67% yield) as a white solid.

m.p. = 174 – 176 °C

^{1}H NMR (400 MHz, $CDCl_3$): δ 8.15 (d, *J* = 8.9 Hz, 2H), 8.10 (d, *J* = 8.6 Hz, 2H), 7.81 (d, *J* = 8.9 Hz, 2H), 7.24 (d, *J* = 8.6 Hz, 2H), 7.17 – 7.10 (m, 2H), 6.99 – 6.93 (m, 1H), 6.93 – 6.87 (m, 1H), 5.23 (dd, *J* = 6.7, 6.7 Hz, 1H), 3.68 (ddd, *J* = 13.5, 5.3, 5.3 Hz, 1H), 3.55 (ddd, *J* = 13.5, 8.3, 6.4 Hz, 1H), 3.29 (dd, *J* = 6.7, 2.0 Hz, 2H), 2.59 – 2.51 (m, 2H).

^{13}C NMR (101 MHz, $CDCl_3$): δ 149.9, 147.1, 145.6, 144.9, 134.1, 132.9, 130.8 (2C), 129.2, 128.2 (2C), 127.9, 127.1, 126.8, 124.3 (2C), 123.6 (2C), 58.4, 44.3, 40.4, 26.9.

HRMS (ESI): m/z calcd. for $C_{22}H_{19}O_6N_3SNa$ $[M+Na]^+$ 476.0927, found 476.0879.

2-(4-Nitrobenzyl)-1,3-dioxolane **(3f)**

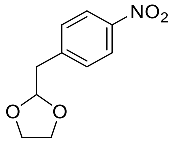

Chemical Formula: $C_{10}H_{11}NO_4$
Exact Mass: 209.07

The general procedure was followed with 2-(4-nitrophenyl)oxirane (33.0 mg, 0.2 mmol) and ethylene glycol (22.4 µL, 0.4 mmol) in the presence of TfOH (1.8 µL, 0.020 mmol, 10 mol%) in HFIP (2.0 mL). The reaction mixture was stirred at 25 °C for 0.25 h. Purification by FC over silica

gel (*n*-pentane/EtOAc 100:0 to 80:20 gradient) afforded **3b** (28.9 mg, 69% yield) as a pale yellow oil. Spectral data are in accordance with those found in the literature.[5]

^{1}H NMR (400 MHz, CDCl$_3$): δ 8.16 (d, *J* = 8.6 Hz, 2H), 7.44 (d, *J* = 8.6 Hz, 2H), 5.11 (t, *J* = 4.4 Hz, 1H), 3.93 – 3.81 (m, 4H), 3.07 (d, *J* = 4.4 Hz, 2H).

^{13}C NMR (126 MHz, CDCl$_3$): δ 147.0, 143.9, 130.9 (2C), 123.5 (2C), 103.7, 65.3 (2C), 40.6.

HRMS (ESI): m/z calcd. for $C_{10}H_{11}O_4NNa$ [M+Na]$^+$ 232.0580, found 232.0574.

2-(4-Nitrobenzyl)-1,3-dioxane **(3g)**

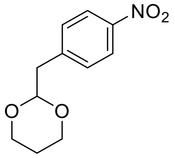

Chemical Formula: $C_{11}H_{13}NO_4$
Exact Mass: 223.08

The general procedure was followed with 2-(4-nitrophenyl)oxirane (33.0 mg, 0.2 mmol) and 1,3-propanediol (29.0 µL, 0.4 mmol) in the presence of TfOH (1.8 µL, 0.020 mmol, 10 mol%) in HFIP (2.0 mL). The reaction mixture was stirred at 25 °C for 0.25 h. Purification by FC over silica gel (*n*-pentane/EtOAc 100:0 to 80:20 gradient) afforded **3c** (32.0 mg, 71% yield) as a white solid.

m.p. = 113 – 115 °C

^{1}H NMR (400 MHz, CDCl$_3$) δ 8.13 (d, *J* = 8.8 Hz, 2H), 7.41 (d, *J* = 8.8 Hz, 2H), 4.72 (t, *J* = 5.1 Hz, 1H), 4.09 (m, 2H), 3.73 (m, 2H), 2.99 (d, *J* = 5.1 Hz, 2H), 2.06 (m, 1H), 1.34 (m, 1H)

^{13}C NMR (101 MHz, CDCl$_3$) δ 146.9, 144.4, 130.8 (2C), 123.5 (2C), 101.6, 67.1 (2C), 41.7, 25.7.

HRMS (ESI): m/z calcd. for $C_{11}H_{13}O_4NNa$ [M+Na]$^+$ 246.0737, found 246.0706.

2-(4-Nitrobenzyl)-3-tosyl-1,3-oxazinane **(3h)**

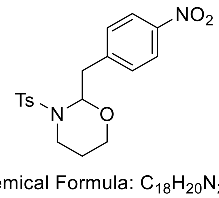

Chemical Formula: $C_{18}H_{20}N_2O_5S$
Exact Mass: 376.11

The general procedure was followed with 2-(4-nitrophenyl)oxirane (33.0 mg, 0.2 mmol) and N-(3-hydroxypropyl)-4-methylbenzenesulfonamide (91.6 mg, 0.4 mmol) in the presence of TfOH (1.8 µL, 0.020 mmol, 10 mol%) in HFIP (2.0 mL). The reaction mixture was stirred at 25 °C for 1 h. Purification by FC over silica gel (DCM) afforded **3d** (47.0 mg, 63% yield) as a white solid.

m.p. = 139 – 141 °C

^{1}H NMR (400 MHz, $CDCl_3$): δ 8.12 (d, *J* = 8.8 Hz, 2H), 7.65 (d, *J* = 8.3 Hz, 2H), 7.37 (d, *J* = 8.8 Hz, 2H), 7.24 (d, *J* = 8.3 Hz, 2H), 5.65 (dd, *J* = 7.4, 6.4 Hz, 1H), 3.96 (m, 1H), 3.81 (dd, *J* = 14.0, 5.3 Hz, 1H), 3.61 – 3.46 (m, 2H), 3.40 (dd, *J* = 14.0, 7.4 Hz, 1H), 3.29 (dd, *J* = 14.0, 6.4 Hz, 1H), 2.40 (s, 3H), 1.52 – 1.38 (m, 1H), 1.32 (m, 1H).

^{13}C NMR (101 MHz, $CDCl_3$): δ 147.0, 144.2, 143.8, 137.6, 130.3 (2C), 129.8 (2C), 127.4 (2C), 123.8 (2C), 84.0, 59.9, 39.3, 36.7, 23.2, 21.6.

HRMS (ESI): m/z calcd. for $C_{18}H_{21}O_5N_2S$ $[M+H]^+$ 377.1166, found 377.1154.

3. Procedures and Characterization of Post-Functionalization Products

***2-(4,5-Dimethoxy-2-(4-nitrophenethyl)phenyl)ethan-1-ol* (4a)**

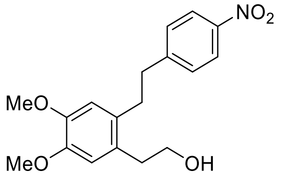

Chemical Formula: $C_{18}H_{21}NO_5$
Exact Mass: 331.14

Synthesized using a known procedure.[6] 6,7-dimethoxy-1-(4-nitrobenzyl)isochromane (69.5 mg, 0.20 mmol) and triethylsilane (63.9 µL, 0.40 mmol) were charged (in air) in a 10 mL screw-cap tube equipped with a Teflon-coated magnetic stir bar. HFIP (2 mL) and TfOH (10 mol%, 1.76 µL, 0.02 mmol) were added (addition of TfOH at 0 °C), and the glass tube was sealed. The reaction mixture was quenched with saturated $NaHCO_3$ (10 mL) and extracted with EtOAc (10 mL × 3). The combined organic layers were washed with brine (10 mL), dried over Na_2SO_4, filtered and concentrated under reduced pressure. Purification by FC over silica gel (*n*-pentane/EtOAc 80:20 to 50:50 gradient) afforded **4a** (49.0 mg, 74% yield) as a yellow solid.

m.p. = 112 – 114 °C

^{1}H NMR (400 MHz, $CDCl_3$): δ 8.10 (d, *J* = 8.7 Hz, 2H), 7.28 (d, *J* = 8.7 Hz, 2H), 6.69 (s, 1H), 6.57 (s, 1H), 3.85 (s, 3H), 3.81 – 3.75 (m, 2H), 3.78 (s, 3H), 3.00 – 2.86 (m, 4H), 2.76 (t, *J* = 6.8 Hz, 2H), 1.71 (brs, 1H).

^{13}C NMR (101 MHz, $CDCl_3$): δ 149.5, 147.6, 147.6, 146.5, 131.2, 129.5 (2C), 128.2, 123.7 (2C), 113.2, 112.9, 63.6, 56.0, 56.0, 37.8, 35.4, 33.9.

HRMS (ESI): m/z calcd. for $C_{18}H_{21}O_5NNa$ $[M+Na]^+$ 354.1312, found 354.1305.

***2-(4,5-Dimethoxy-2-(4-nitrophenethyl)phenethyl)-1,3,5-trimethylbenzene* (4b)**

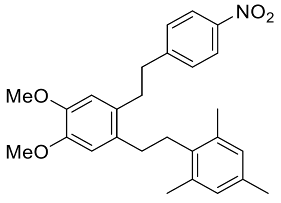

Chemical Formula: $C_{27}H_{31}NO_4$
Exact Mass: 433.23

Synthesized using a known procedure.[7] 2-(4,5-dimethoxy-2-(4-nitrophenethyl)phenyl)ethan-1-ol (66.2 mg, 0.20 mmol) and mesitylene (139.1 µL, 1.00 mmol) were charged (in air) in a 10 mL screw-cap tube equipped with a Teflon-coated magnetic stir bar. HFIP (1 mL) and TfOH (10 mol%, 1.76 µL, 0.02 mmol) were added (addition of TfOH at 0 °C), and the glass tube was sealed. The reaction mixture was quenched with saturated $NaHCO_3$ (10 mL) and extracted with EtOAc (10 mL × 3). The combined organic layers were washed with brine (10 mL), dried over Na_2SO_4,

filtered and concentrated under reduced pressure. Purification by FC over silica gel (*n*-pentane/EtOAc 100:0 to 80:20 gradient) afforded **4b** (79.0 mg, 91% yield) as a white solid.

m.p. = 86 – 89 °C

^{1}H NMR (400 MHz, CDCl$_3$): δ 8.13 (d, *J* = 8.6 Hz, 2H), 7.27 (d, *J* = 8.6 Hz, 2H), 6.84 (s, 1H), 6.84 (s, 1H), 6.61 (s, 1H), 6.56 (s, 1H), 3.82 (s, 3H), 3.81 (s, 3H), 3.00 – 2.79 (m, 6H), 2.68 – 2.62 (m, 2H), 2.25 (s, 3H), 2.24 (s, 6H).

^{13}C NMR (126 MHz, CDCl$_3$): δ 149.6, 147.6, 147.4, 146.6, 136.1, 135.5, 135.3, 132.1, 130.4, 129.4 (2C), 129.2 (2C), 123.8 (2C), 112.9, 112.7, 56.1, 56.0, 37.9, 33.8, 31.9, 31.3, 27.1, 20.9, 20.0 (2C).

HRMS (ESI): m/z calcd. for $C_{27}H_{32}O_4N$ $[M+H]^+$ 434.2326, found 434.2321.

4-((6,7-Dimethoxyisochroman-1-yl)methyl)aniline **(4c)**

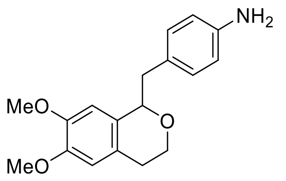

Synthesized using a known procedure.[8] To a solution of 6,7-dimethoxy-1-(4-nitrobenzyl)isochromane (72.7 mg, 0.21 mmol) in 4 mL of EtOAc were added palladium on carbon (10 wt%) (7.3 mg, 0.002 mmol). The reaction mixture was placed under H_2 gas at 30 bar for 24 h. Then, the reaction mixture was filtered over a pad of celite (rinsed with EtOAc (3 × 5 mL). The solvent was removed by rotary evaporation and purification by FC over silica gel (*n*-pentane/EtOAc 70:30 to 50:50 gradient) afforded **4c** (58.0 mg, 92% yield) as a colorless oil.

^{1}H NMR (400 MHz, CDCl$_3$): δ 7.06 (d, *J* = 8.3 Hz, 2H), 6.63 (d, *J* = 8.3 Hz, 2H), 6.59 (s, 1H), 6.50 (s, 1H), 4.90 (dd, *J* = 8.1, 4.7 Hz, 1H), 4.11 (ddd, *J* = 11.2, 4.8, 4.3 Hz, 1H), 3.85 (s, 3H), 3.78 (s, 3H), 3.74 (ddd, *J* = 11.2, 8.5, 4.3 Hz, 1H), 3.41 (brs, 2H), 3.05 (dd, *J* = 14.2, 4.7 Hz, 1H), 2.98 (dd, *J* = 14.2, 8.1 Hz, 1H), 2.82 (ddd, *J* = 15.9, 8.5, 4.8, 1H), 2.62 (ddd, *J* = 15.9, 4.3, 4.3 Hz, 1H).

^{13}C NMR (101 MHz, CDCl$_3$): δ 147.5, 147.1, 144.7, 130.4 (2C), 129.8, 128.7, 126.2, 115.2 (2C), 111.5, 108.5, 76.6, 62.8, 55.9, 55.9, 42.0, 28.7.

HRMS (ESI): m/z calcd. for $C_{18}H_{22}NO_3$ $[M+H]^+$ 300.1594, found 300.1586.

***1-(4-Nitrobenzyl)-3,4-dihydro-1H-isochromene-5,8-dione* (4d)**

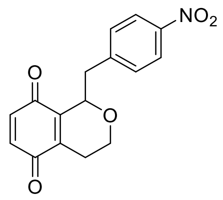

Chemical Formula: $C_{16}H_{13}NO_5$
Exact Mass: 299.08

Synthesized using a known procedure.[9] Under air, a 10 mL tube equipped with a Teflon-coated magnetic stir bar was charged with 5,8-dimethoxy-1-(4-nitrobenzyl)isochromane (98.7 mg, 0.30 mmol), MeCN (3 mL) and H_2O (3 mL). The mixture was cooled down to 0 °C in an ice bath. Then, cerium ammonium nitrate (987.0 mg, 1.8 mmol) was added and the reaction was stirred at 0 °C for 1 h. The reaction mixture was quenched with saturated H_2O (10 mL) and extracted with DCM (10 mL × 3). The combined organic layers were washed with brine (10 mL), dried over Na_2SO_4, filtered and concentrated under reduced pressure. Purification by FC over silica gel (*n*-pentane/EtOAc 100:0 to 80:20 gradient) afforded **4d** (60.1 mg, 67% yield) as a yellow solid.

^{1}H NMR (400 MHz, $CDCl_3$): δ 8.13 (d, *J* = 8.6 Hz, 2H), 7.42 (d, *J* = 8.6 Hz, 2H), 6.79 – 6.70 (m, 2H), 4.83 (dd, *J* = 8.6, 2.8 Hz, 1H), 4.04 (ddd, *J* = 11.4, 5.2, 4.5 Hz, 1H), 3.68 (ddd, *J* = 11.4, 6.5, 4.5 Hz, 1H), 3.30 (dd, *J* = 14.1, 2.8 Hz, 1H), 3.08 (dd, *J* = 14.1, 8.6 Hz, 1H), 2.57 – 2.46 (m, 1H), 2.46 – 2.34 (m, 1H) .

^{13}C NMR (101 MHz, $CDCl_3$): δ 186.1, 185.6, 146.9, 146.2, 142.0, 141.6, 136.7, 136.3, 130.4 (2C), 123.6 (2C), 71.8, 60.3, 39.1, 22.5.

HRMS (ESI): m/z calcd. for $C_{16}H_{13}O_5NNa$ [M+Na] + 322.0686, found 322.0680.

***1-(4-Nitrobenzyl)-3,4-dihydro-1H-benzo[g]isochromene-5,10-dione* (4e)**

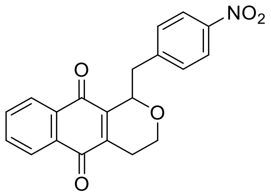

Chemical Formula: $C_{20}H_{15}NO_5$
Exact Mass: 349.10

Synthesized using a known procedure.[10] A solution of 1-(4-Nitrobenzyl)-3,4-dihydro-1H-isochromene-5,8-dione (17 mg, 0.057 mmol) and buta-1,3-dien-1-yl acetate (28.6 mg, 0.257 mmol) in toluene (0.35 mL) was set aside at room temperature for 2 days. The mixture was evaporated to dryness under reduced pressure and the residual oil was dissolved in ethanol (1.5 mL). To the solution was added 1% sodium carbonate (0.15 mL) and the mixture was stirred at room temperature for 5 hours, diluted with EtOAc and washed with water. The organic layer was dried using Na_2SO_4 and the solvent was evaporated under reduced pressure. Purification by FC

over silica gel (*n*-pentane/EtOAc 100:0 to 70:30 gradient) afforded **4e** (18.0 mg, 90% yield) as a white solid.

m.p. = decomposition at 180 °C

^{1}H NMR (400 MHz, $CDCl_3$): δ 8.16 – 8.07 (m, 4H), 7.78 – 7.73 (m, 2H), 7.47 (d, *J* = 8.7 Hz, 2H), 5.02 (dd, *J* = 8.6, 2.8 Hz, 1H), 4.09 (ddd, *J* = 11.4, 5.2, 4.6 Hz, 1H), 3.74 (ddd, *J* = 11.4, 6.4, 4.6 Hz, 1H), 3.41 (dd, *J* = 14.1, 2.8 Hz, 1H), 3.14 (dd, *J* = 14.1, 8.6 Hz, 1H), 2.78 – 2.63 (m, 1H), 2.63 – 2.48 (m, 1H).

^{13}C NMR (126 MHz, $CDCl_3$): δ 183.9, 183.5, 146.9, 146.5, 144.3, 143.9, 134.1, 132.2, 131.9, 130.5 (2C), 126.6, 126.5, 123.6 (2C), 72.2, 60.3, 39.2, 27.1, 23.1.

HRMS (ESI): m/z calcd. for $C_{20}H_{15}O_5NNa$ $[M+Na]^+$ 372.0842, found 372.0838.

References

[1] W. Ding, X. Shi, *Chin. J. Chem.*, **2015**, *33*, 1276-1286.

[2] N. Kapadia, W. Harding, *Tetrahedron*, **2013**, *42*, 8914-8920.

[3] P. King, P. Rutledge, M. Todd, *ChemRxiv*, **2022**. DOI: 10.26434/chemrxiv-2022-3njq6.

[4] I. Ivanov, S. Nikolova, E. Kochovska, S. Statkova-Abeghe, *Arkivoc*, **2007**, 31-44.

[5] I. Kondolff, H. Doucet, M. Santelli, *Eur. J. Org. Chem.*, **2006**, *3*, 765-774.

[6] M. Vayer, S. Zhang, J. Moran, D. Lebœuf, *ACS Catal.* **2022**, *12*, 3309-3316.

[7] S. Zhang, M. Vayer, F. Noël, V. D. Vukovic, A. Golushko, N. Rezajooei, C. N. Rowley, D. Lebœuf, J. Moran, *Chem* **2021**, *7*, 3425-3441.

[8] S. Wang, R. Guillot, J.-F. Carpentier, Y. Sarazin, C. Bour, V. Gandon, D. Lebœuf, *Angew. Chem. Int. Ed.* **2020**, *59*, 1134-1138.

[9] M. Vayer, R. J. Mayer, J. Moran, D. Lebœuf, *ACS Catal*. **2022**, *12*, 10995-11001.

[10] T. Kometani, E. Yoshii, *J. Chem. Soc. Perkin Trans.* 1, **1981**, 1191-1196.

4. NMR Spectra

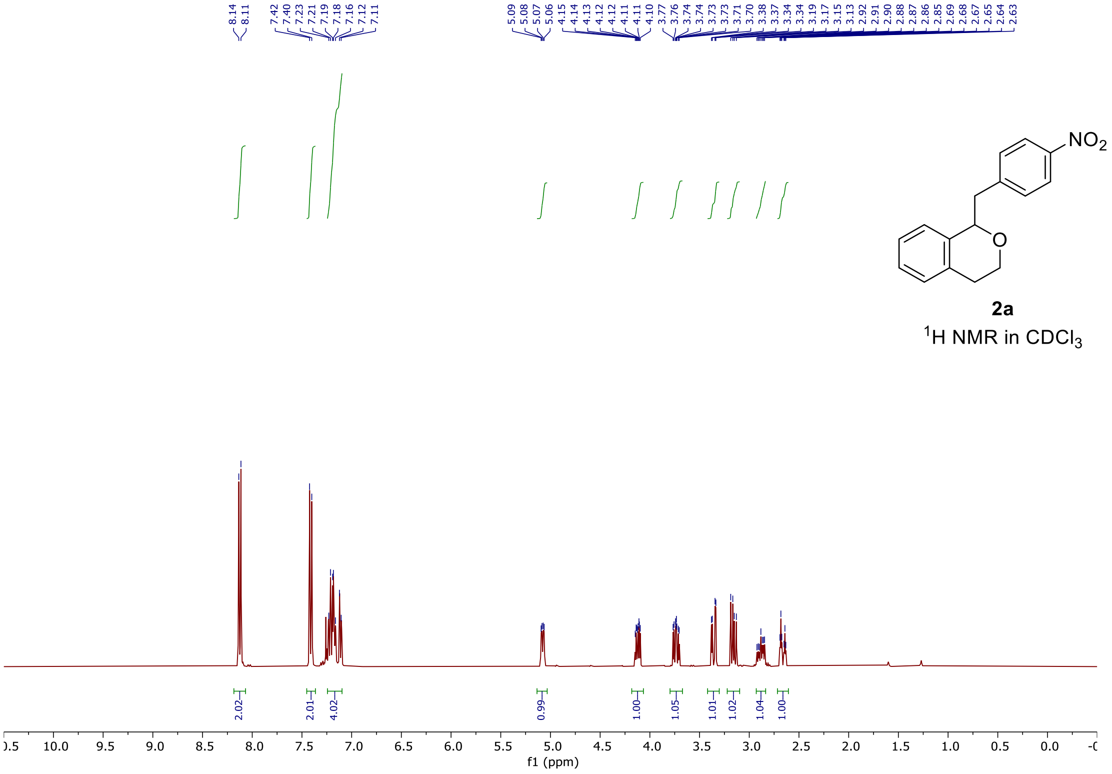

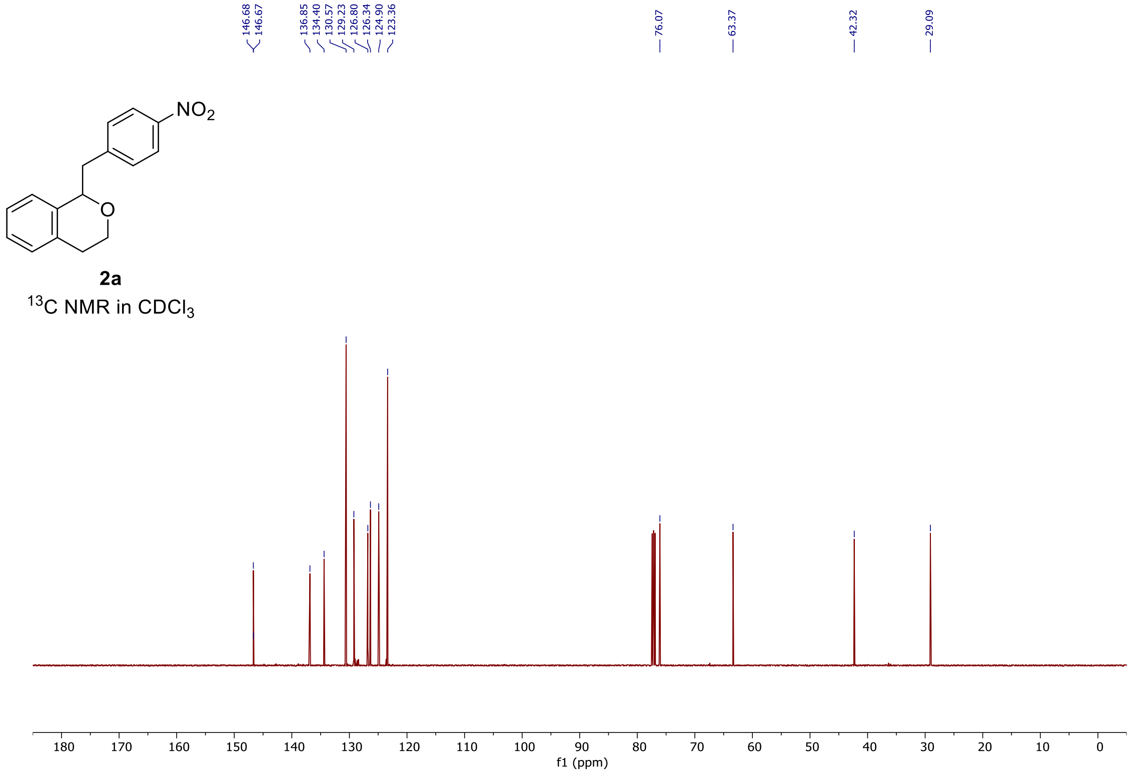

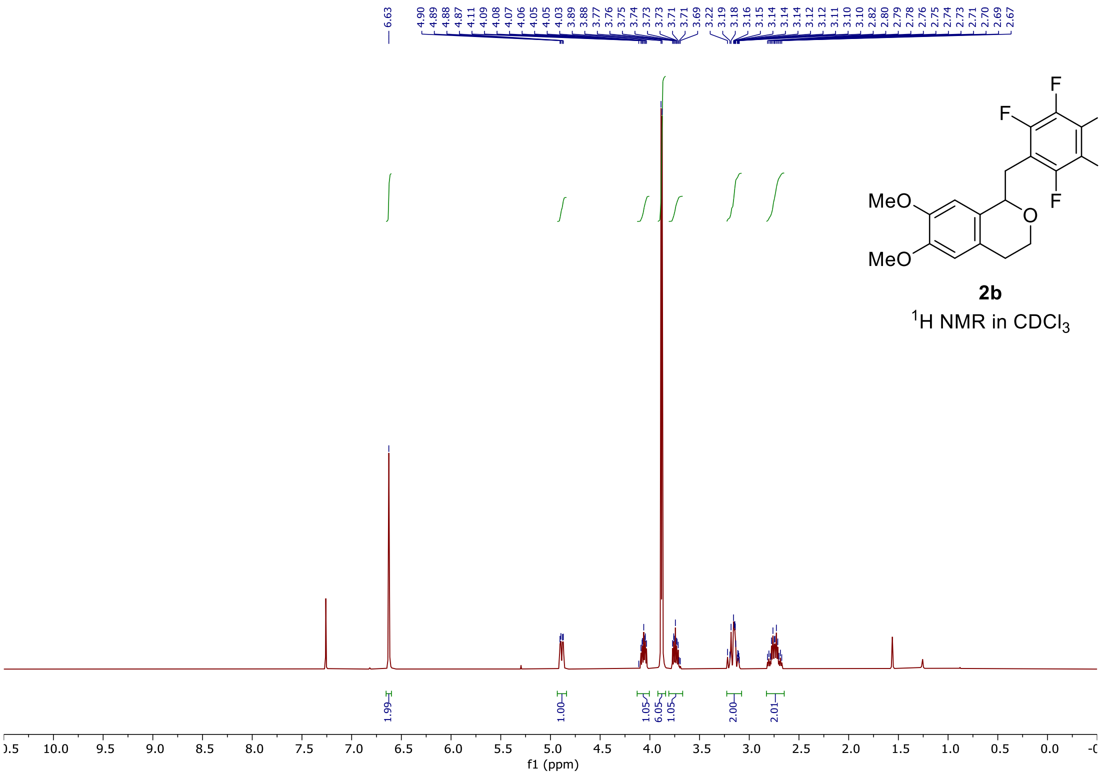

2b

^{1}H NMR in $CDCl_3$

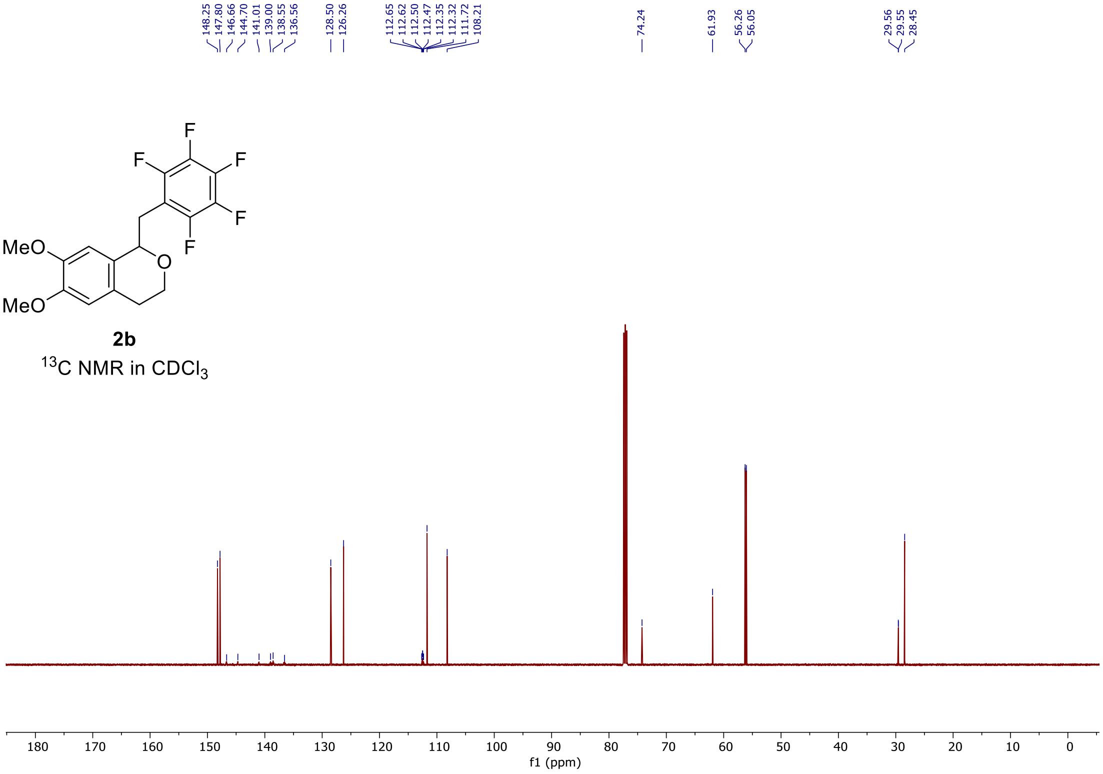

2b

^{13}C NMR in $CDCl_3$

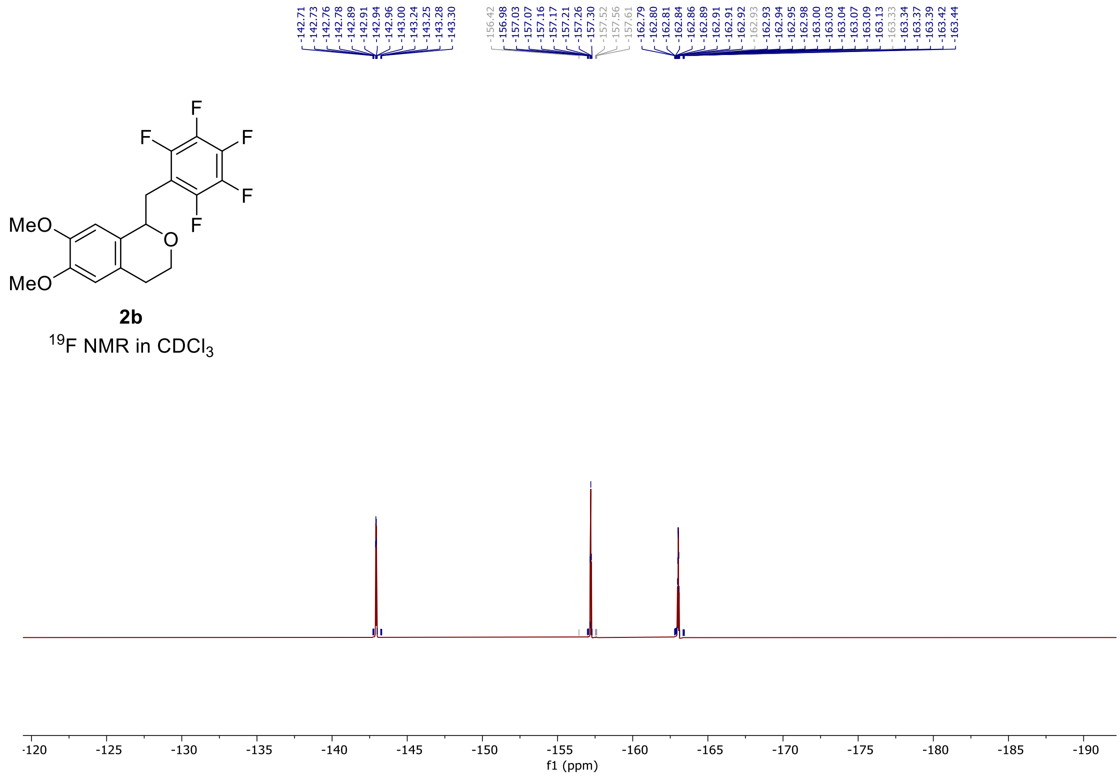

2b
^{19}F NMR in $CDCl_3$

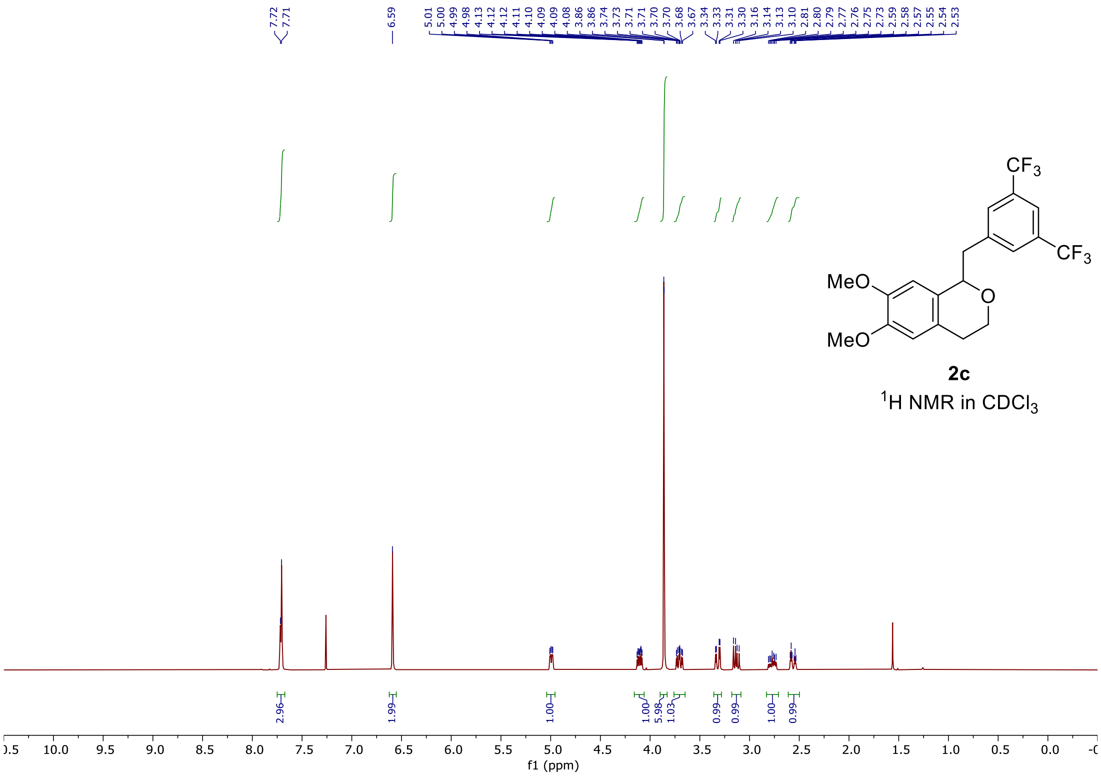

2c
^{1}H NMR in $CDCl_3$

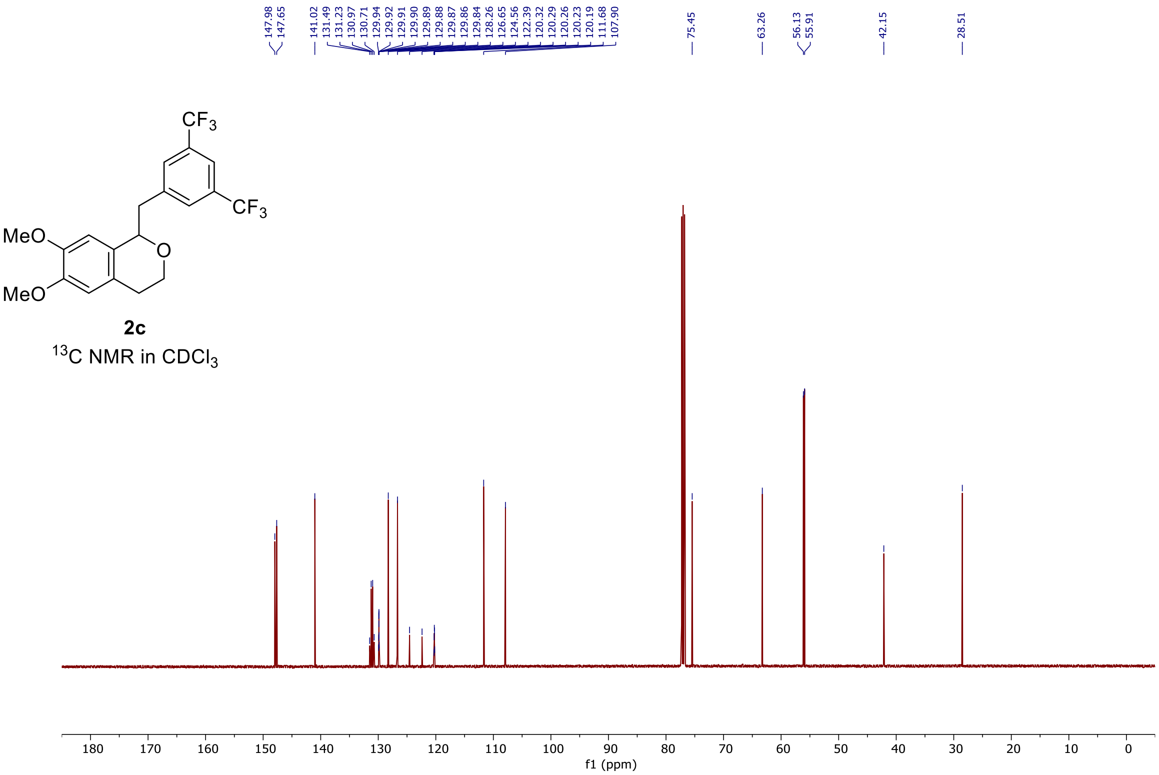

2c

^{13}C NMR in CDCl$_3$

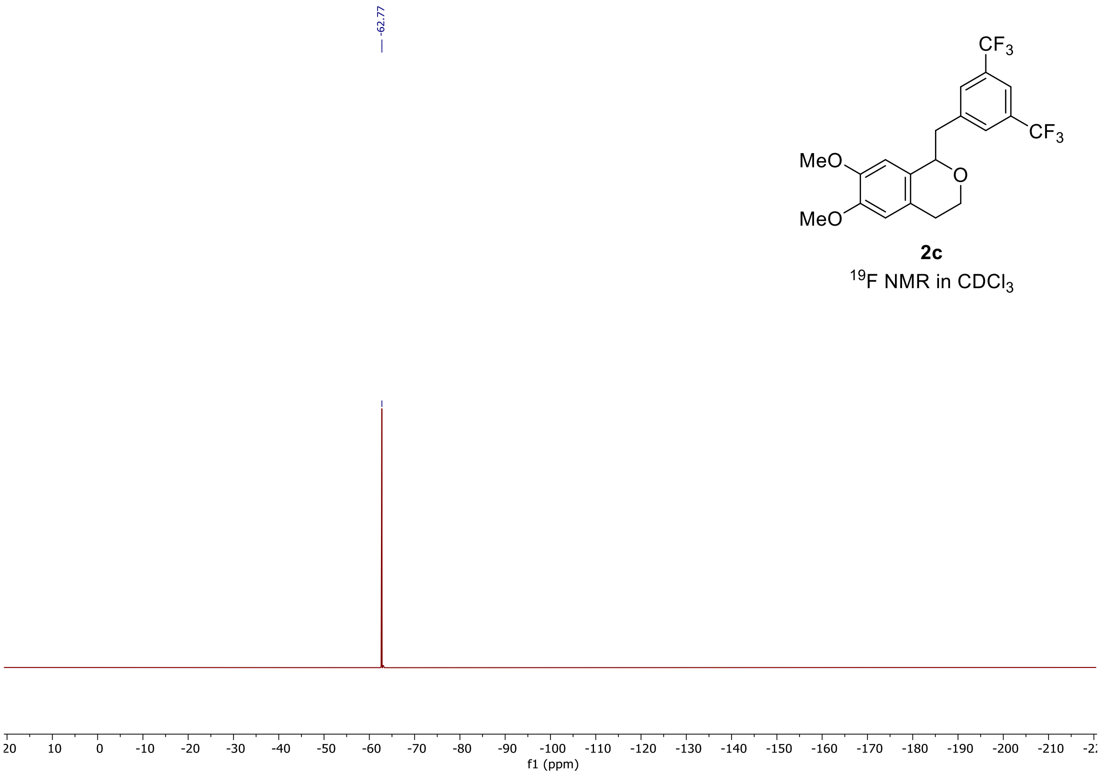

2c

^{19}F NMR in CDCl$_3$

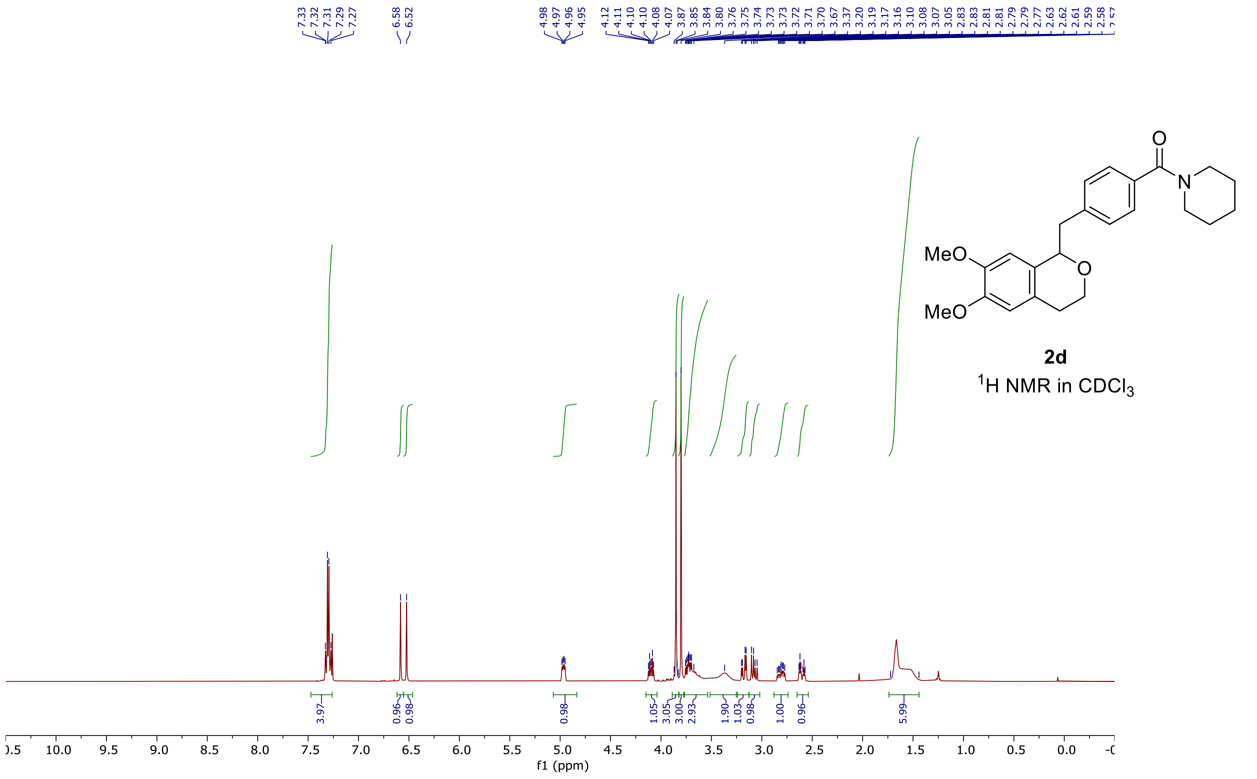

MeO
MeO
2d
^{1}H NMR in $CDCl_3$
f1 (ppm)

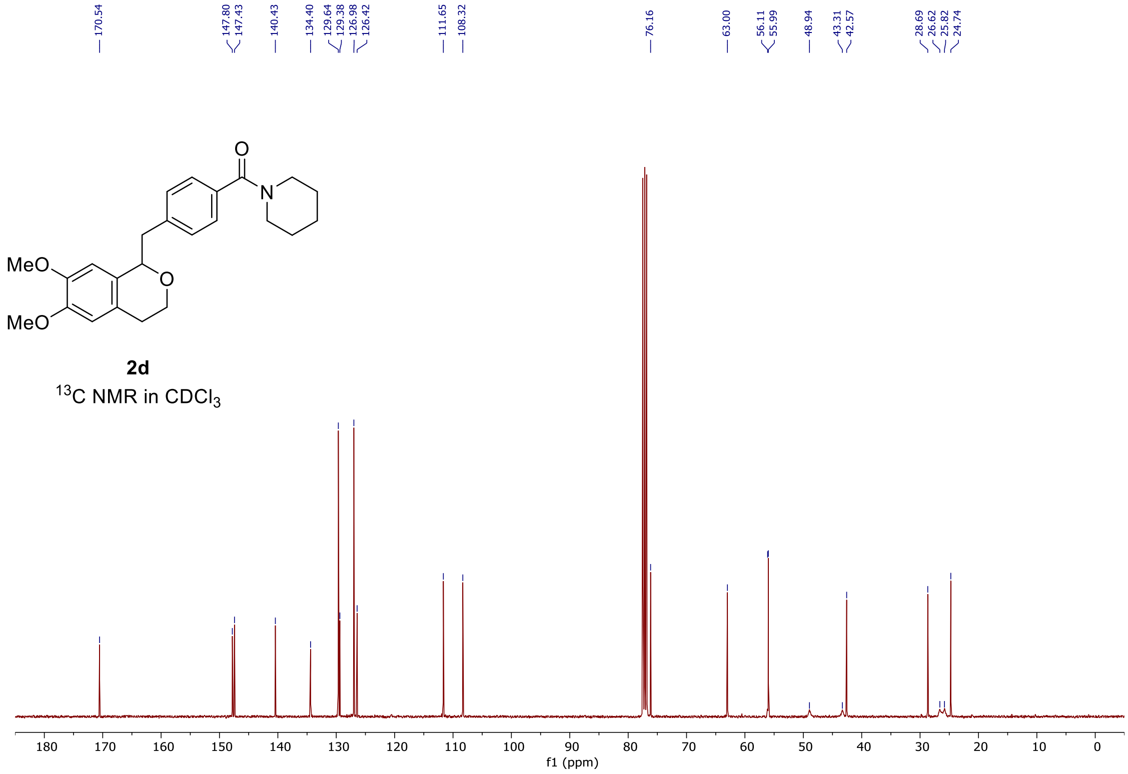

MeO
MeO
2d
^{13}C NMR in $CDCl_3$
f1 (ppm)

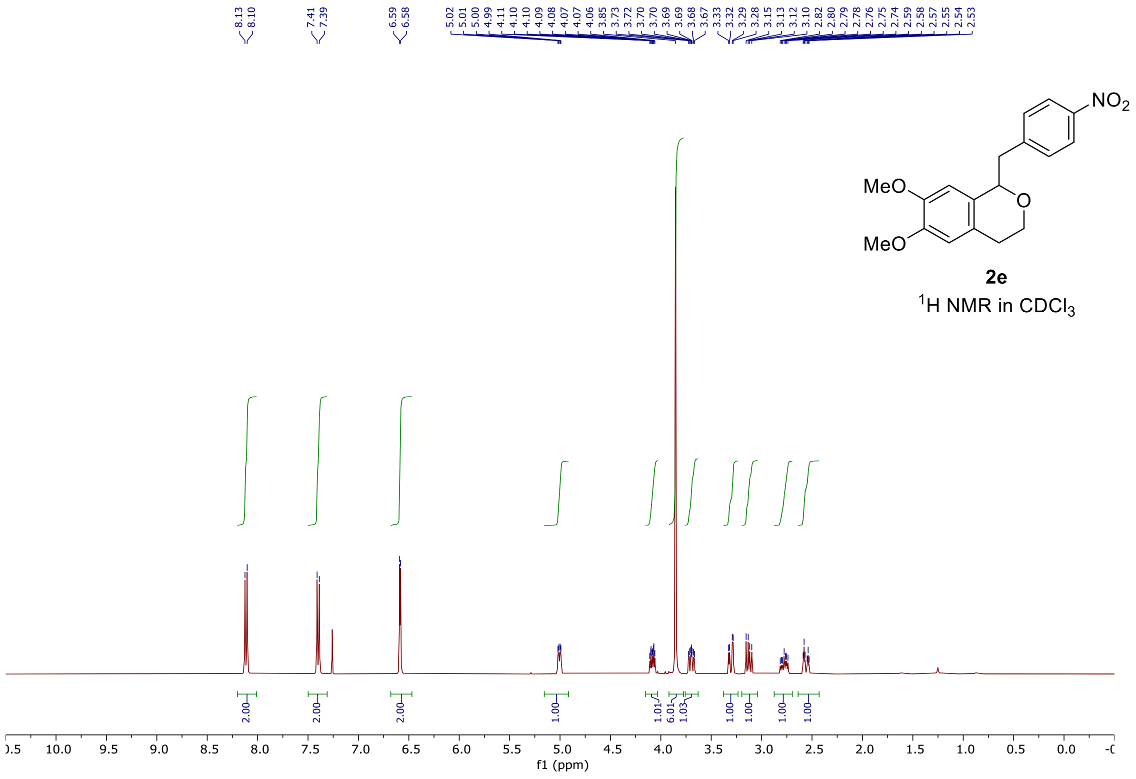
8.13
8.10
7.41
7.39
6.59
6.58
5.02
5.01
5.00
4.99
4.11
4.10
4.10
4.09
4.08
4.07
4.07
4.06
3.85
3.73
3.72
3.70
3.70
3.69
3.69
3.68
3.67
3.33
3.32
3.29
3.28
3.15
3.13
3.12
3.10
2.82
2.80
2.79
2.78
2.76
2.75
2.74
2.59
2.58
2.57
2.55
2.54
2.53
NO2
MeO
O
MeO
2e
1H NMR in CDCl3
2.00
2.00
2.00
1.00
1.01
6.01
1.03
1.00
1.00
1.00
1.00
f1 (ppm)

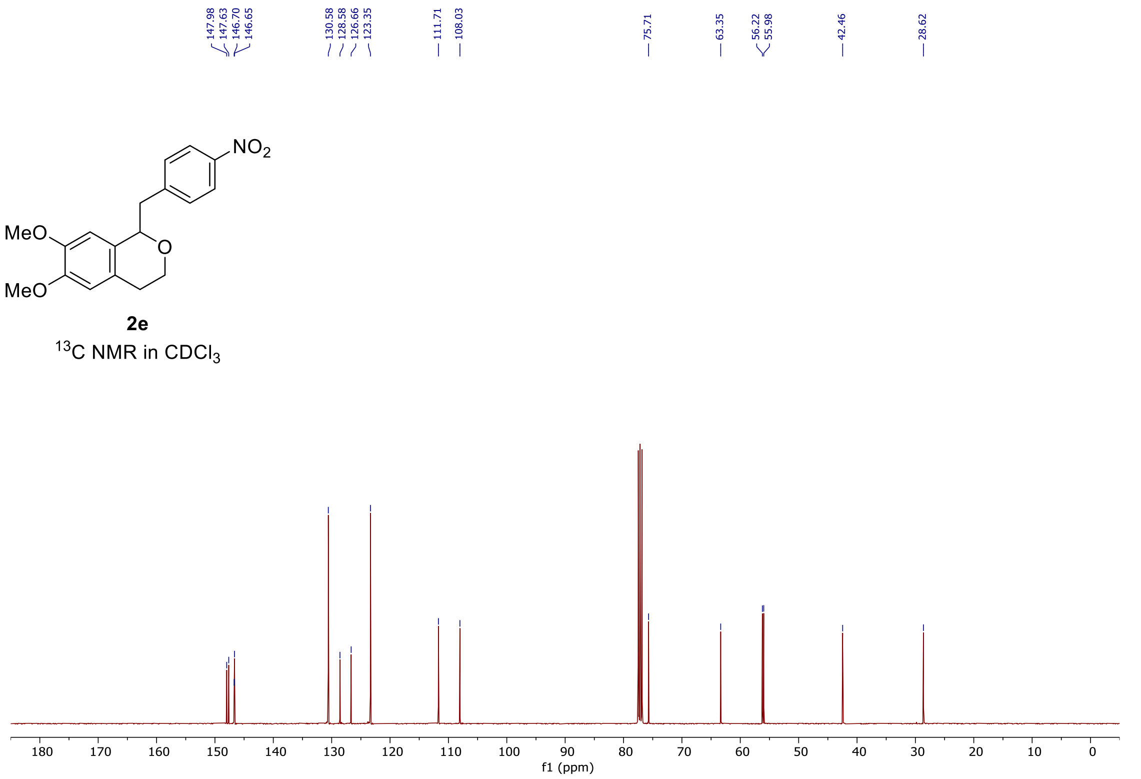
147.98
147.63
146.70
146.65
130.58
128.58
126.66
123.35
111.71
108.03
75.71
63.35
56.22
55.98
42.46
28.62
NO2
MeO
O
MeO
2e
13C NMR in CDCl3
f1 (ppm)

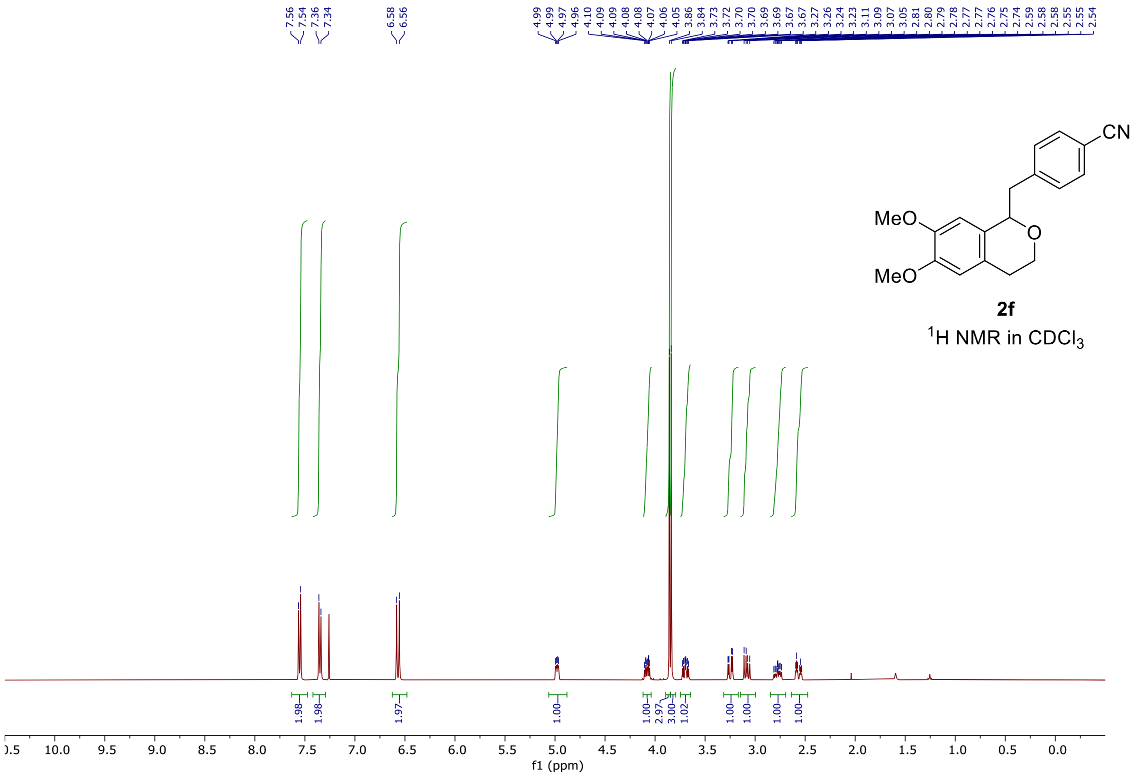
7.56 7.54 7.36 7.34 6.58 6.56 4.99 4.99 4.97 4.96 4.10 4.09 4.09 4.08 4.08 4.07 4.06 4.05 3.86 3.84 3.73 3.72 3.70 3.70 3.69 3.69 3.67 3.67 3.27 3.26 3.24 3.23 3.11 3.09 3.07 3.05 2.81 2.80 2.79 2.78 2.77 2.76 2.75 2.74 2.59 2.58 2.58 2.55 2.55 2.54
CN
MeO
MeO
O
2f
1H NMR in CDCl3
f1 (ppm)

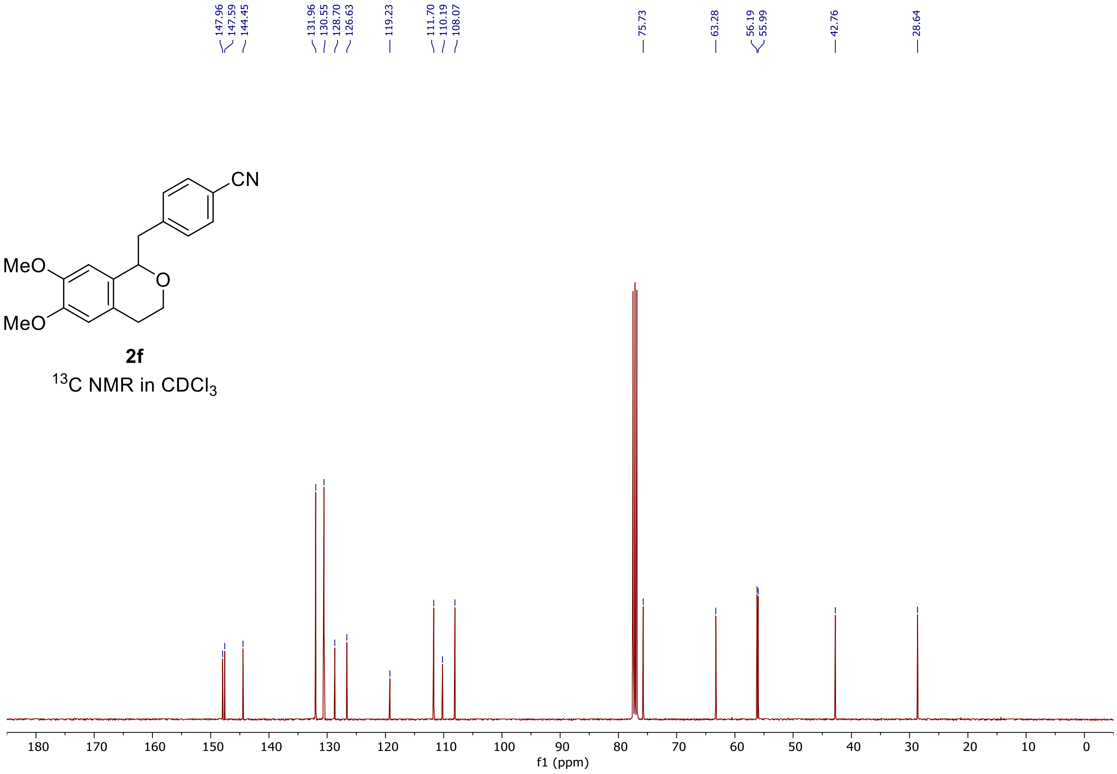
147.96 147.59 144.45 131.96 130.55 128.70 126.63 119.23 111.70 110.19 108.07 75.73 63.28 56.19 55.99 42.76 28.64
CN
MeO
MeO
O
2f
13C NMR in CDCl3
f1 (ppm)

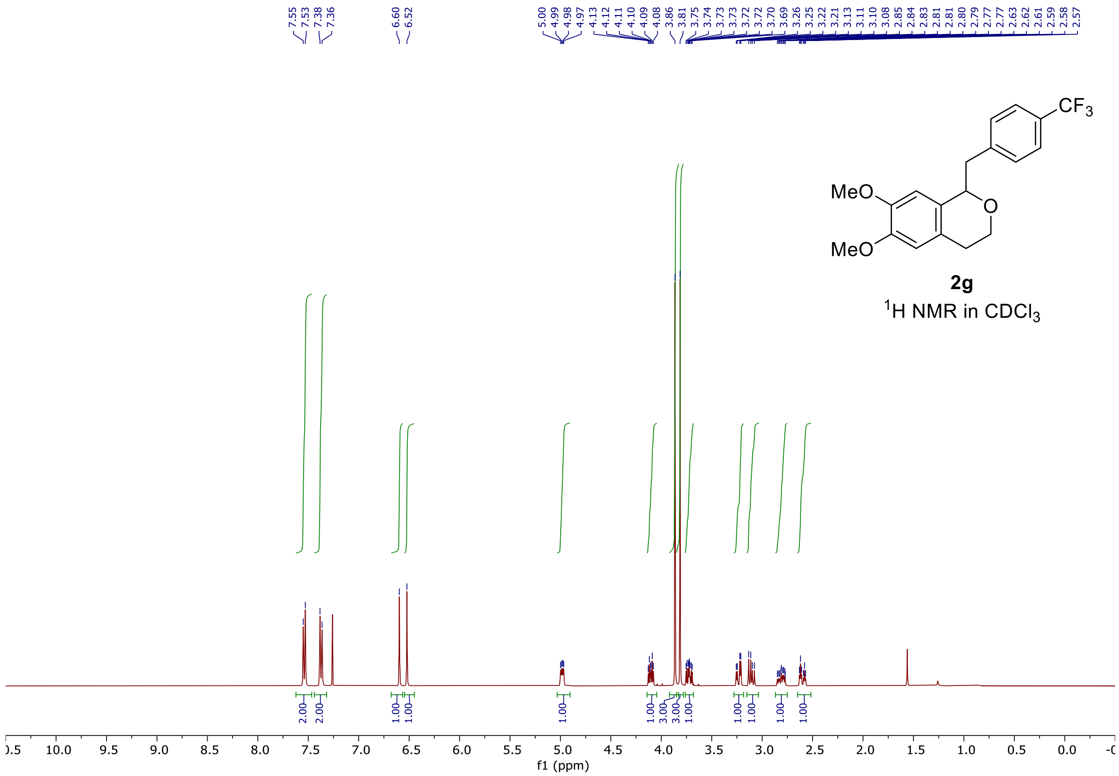

2g

^{1}H NMR in $CDCl_3$

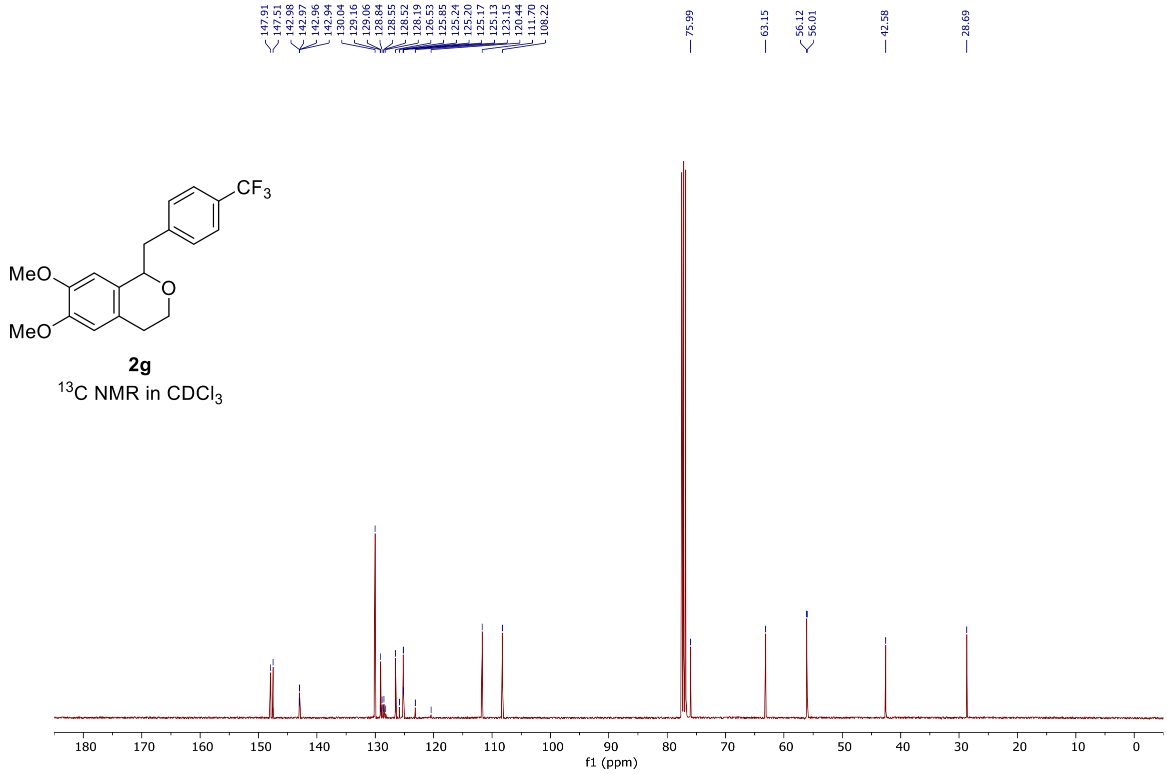

2g

^{13}C NMR in $CDCl_3$

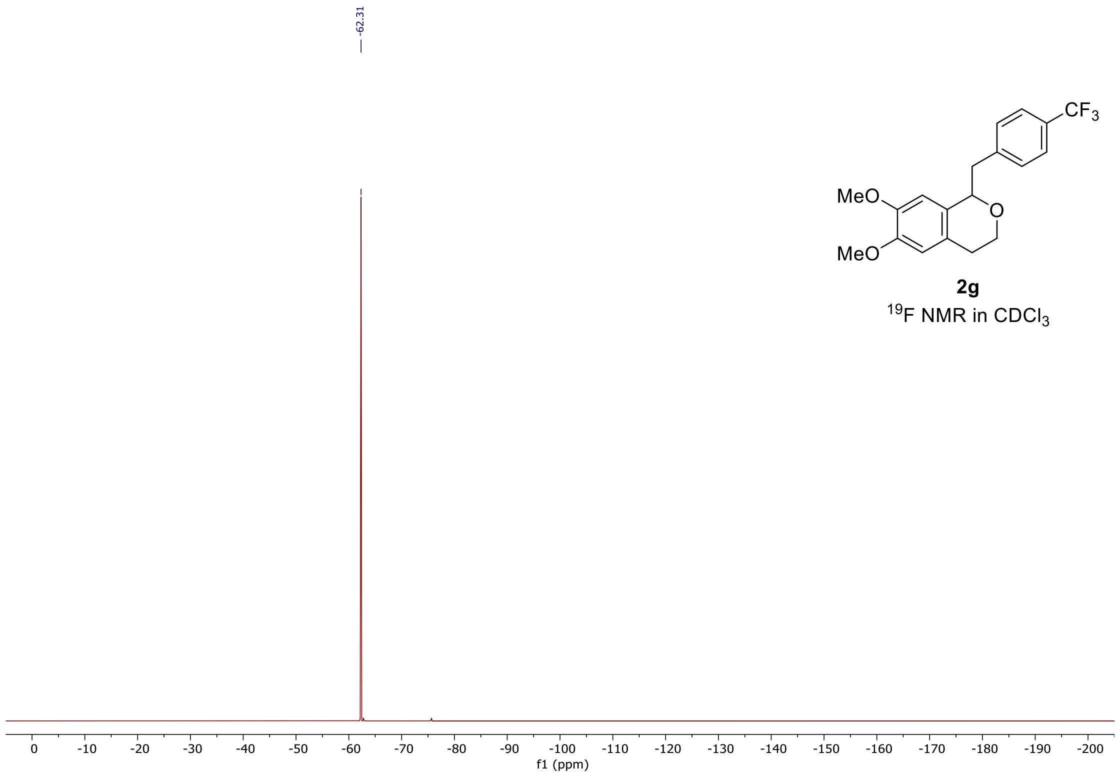

2g
^{19}F NMR in $CDCl_3$

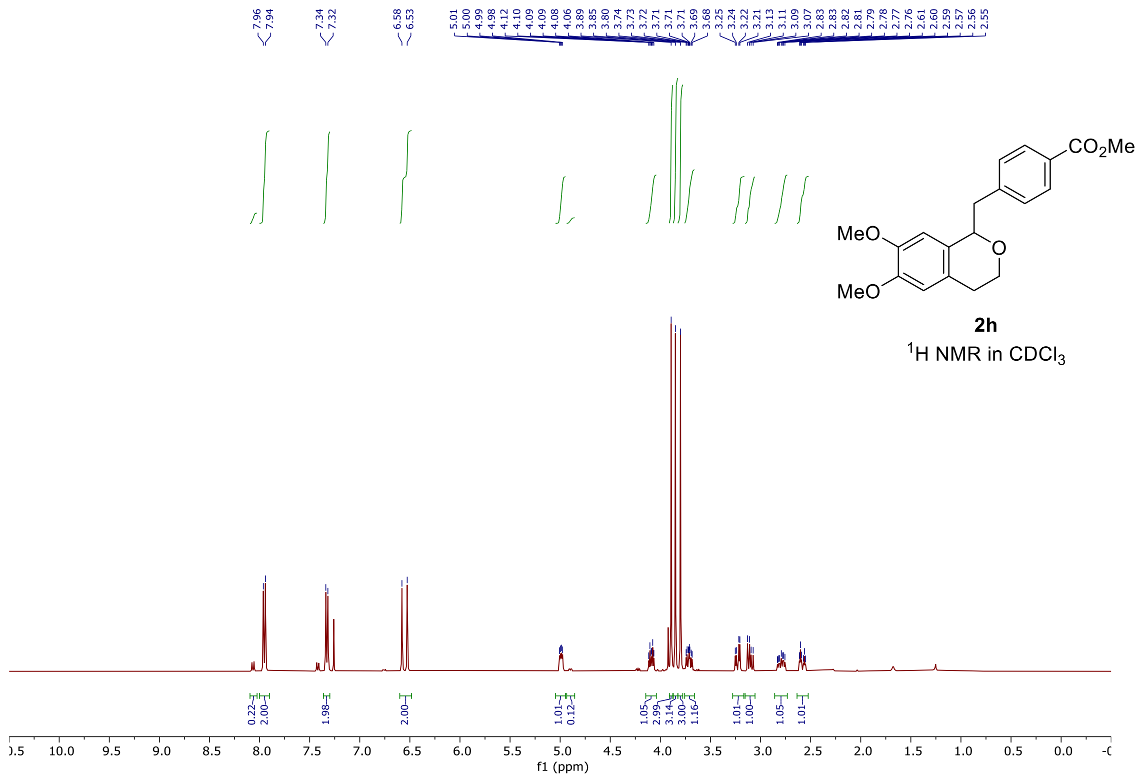

2h
^{1}H NMR in $CDCl_3$

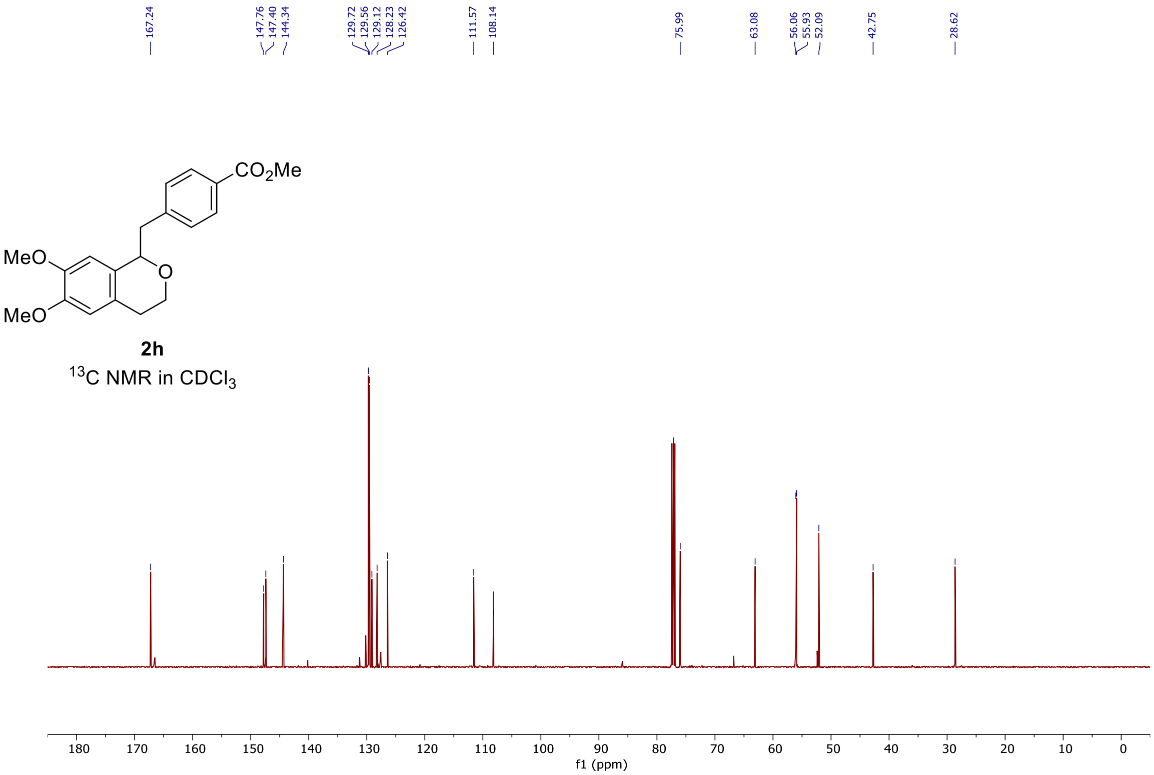

167.24
147.76
147.40
144.34
129.72
129.56
129.12
128.23
126.42
111.57
108.14
75.99
63.08
56.06
55.93
52.09
42.75
28.62
CO2Me
MeO
MeO
O
2h
13C NMR in CDCl3
f1 (ppm)

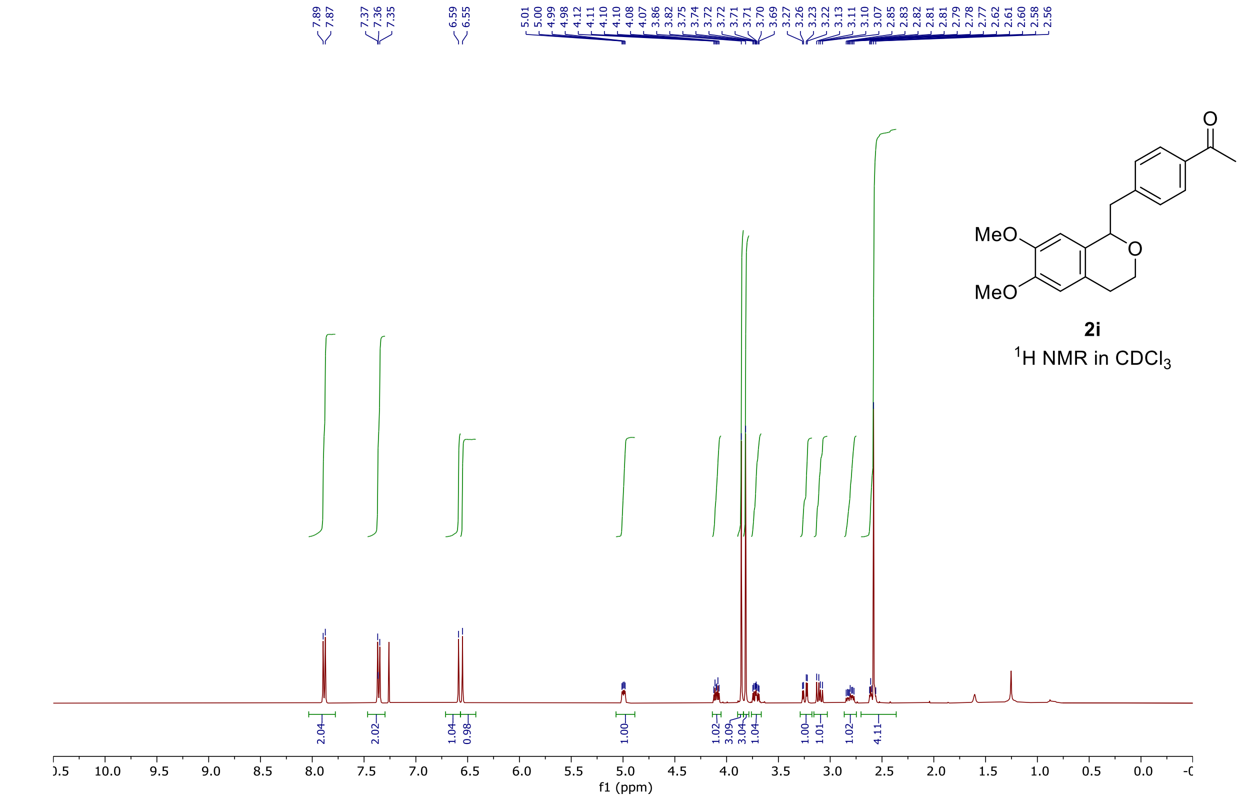

7.89
7.87
7.37
7.36
7.35
6.59
6.55
O
MeO
MeO
O
2i
1H NMR in CDCl3
2.04
2.02
1.04
0.98
1.00
1.02
3.09
3.04
1.04
1.00
1.01
1.02
4.11
f1 (ppm)

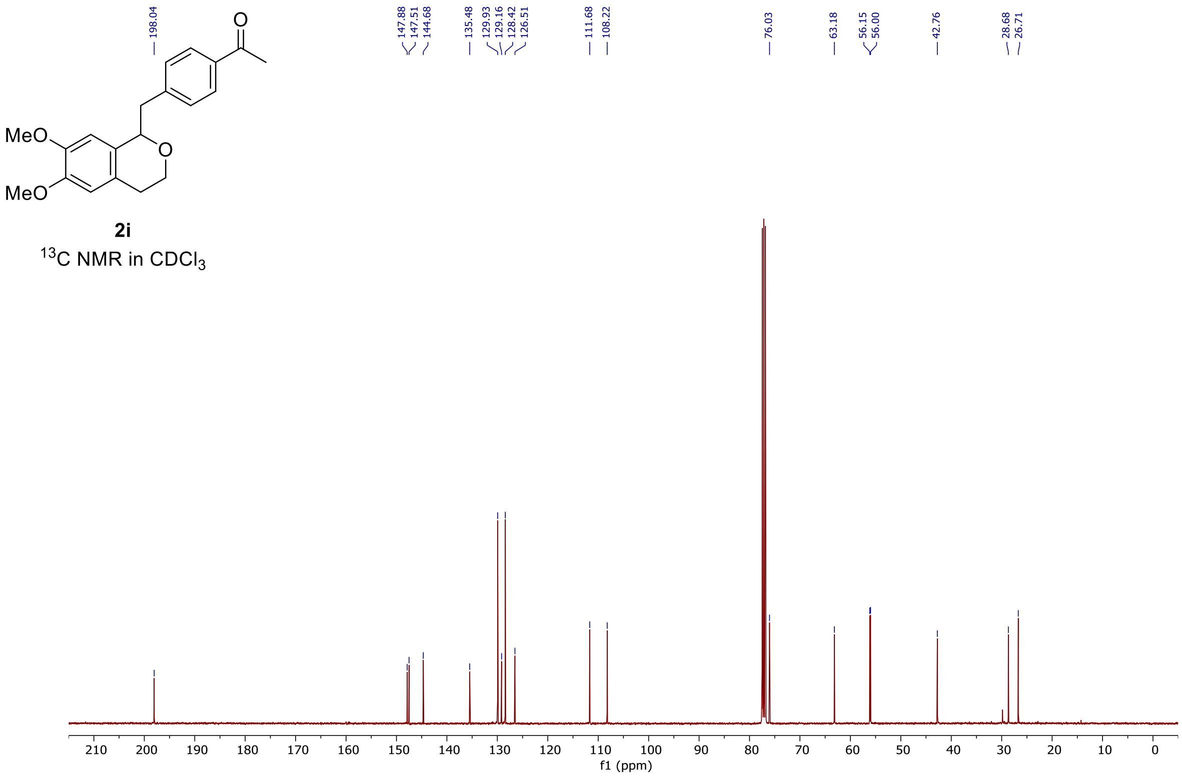

2i

^{13}C NMR in $CDCl_3$

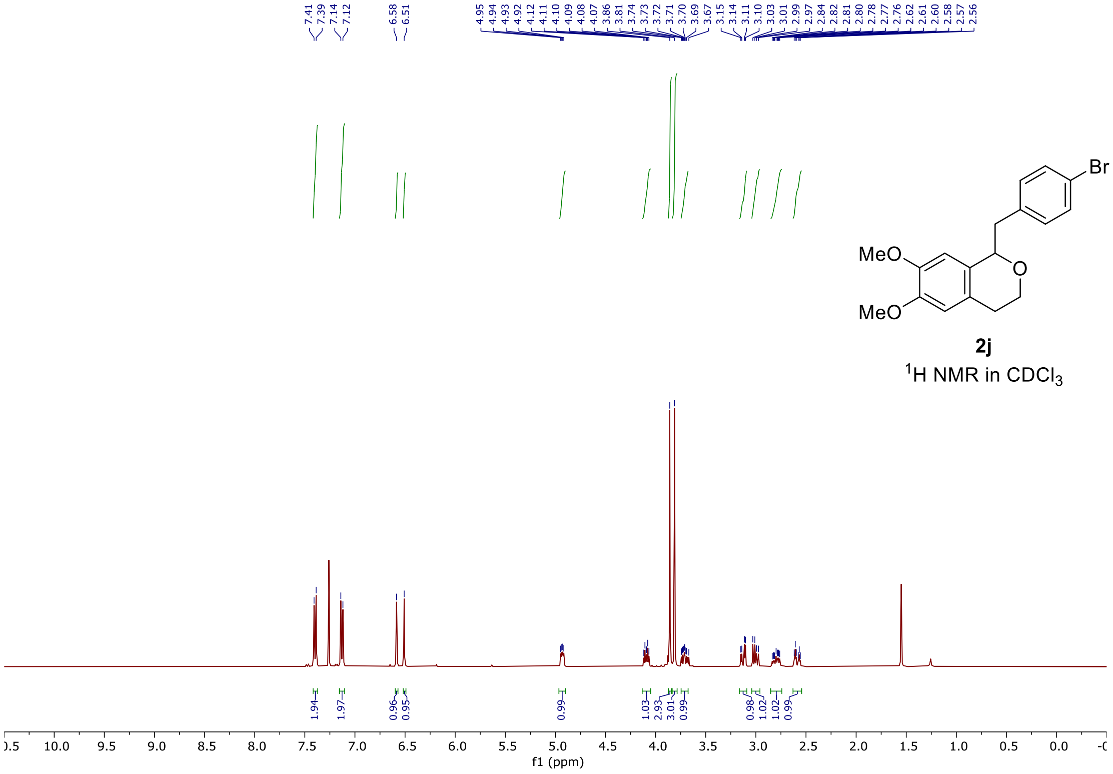

2j

^{1}H NMR in $CDCl_3$

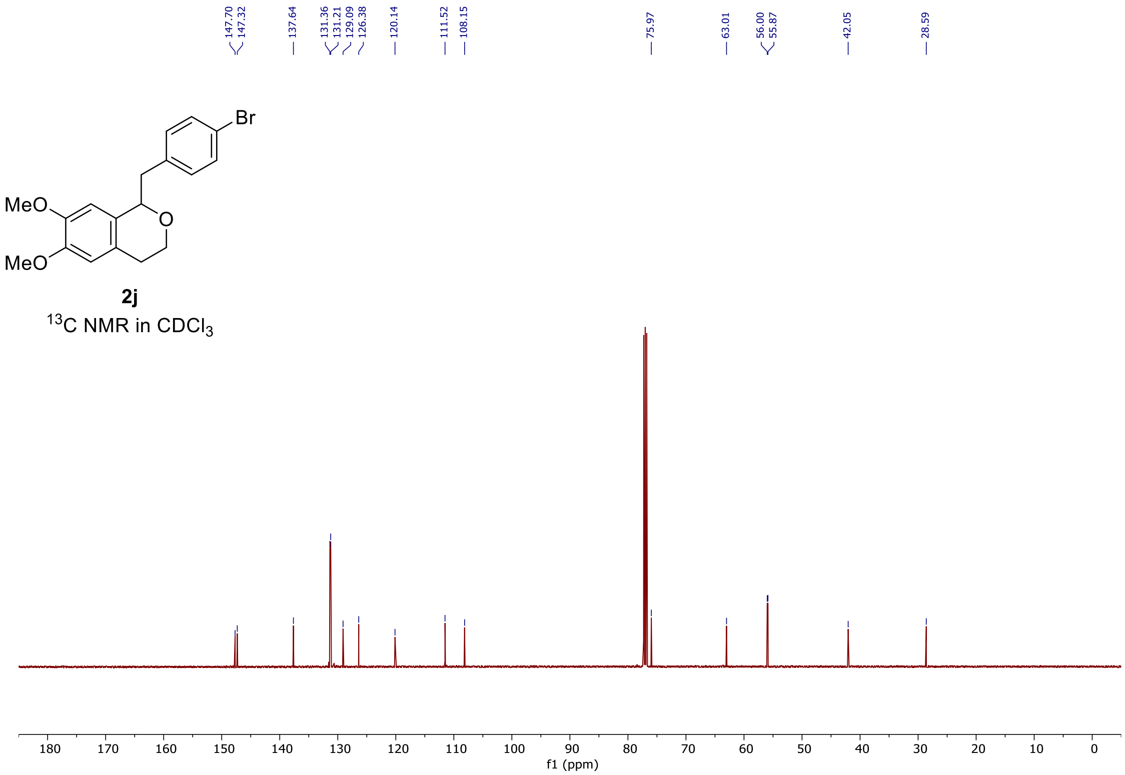

2j

^{13}C NMR in $CDCl_3$

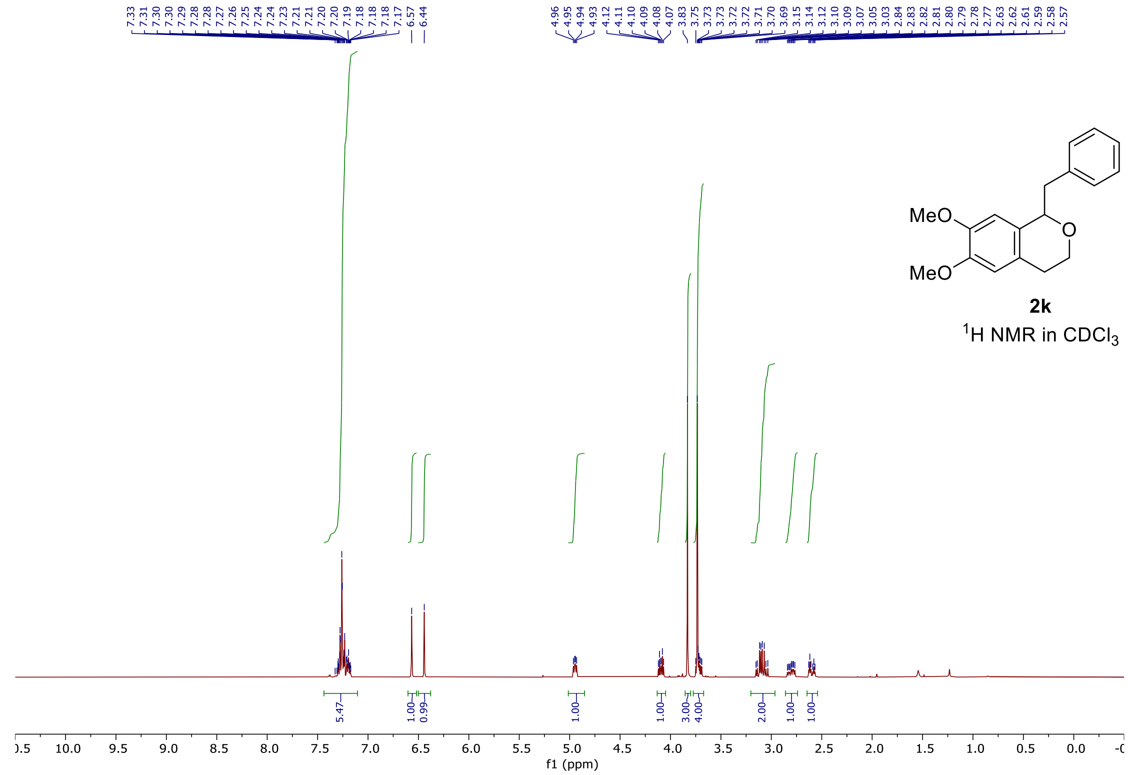

2k

^{1}H NMR in $CDCl_3$

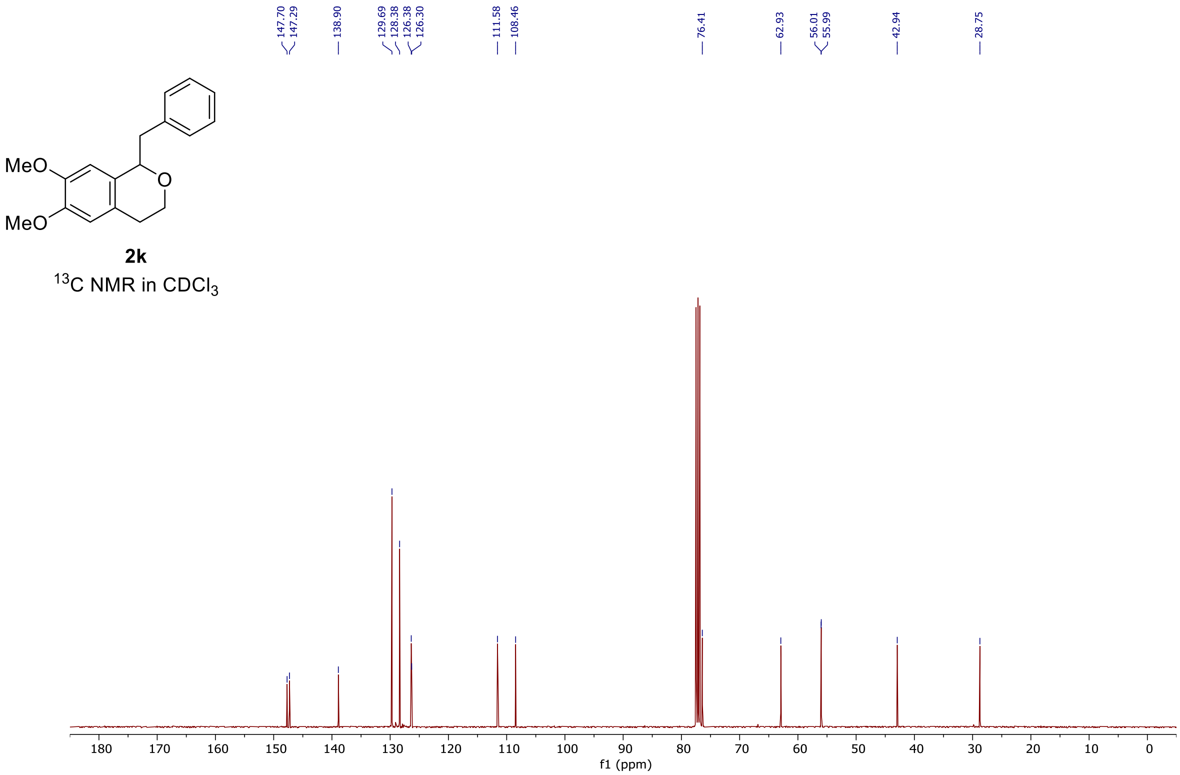

2k

^{13}C NMR in $CDCl_3$

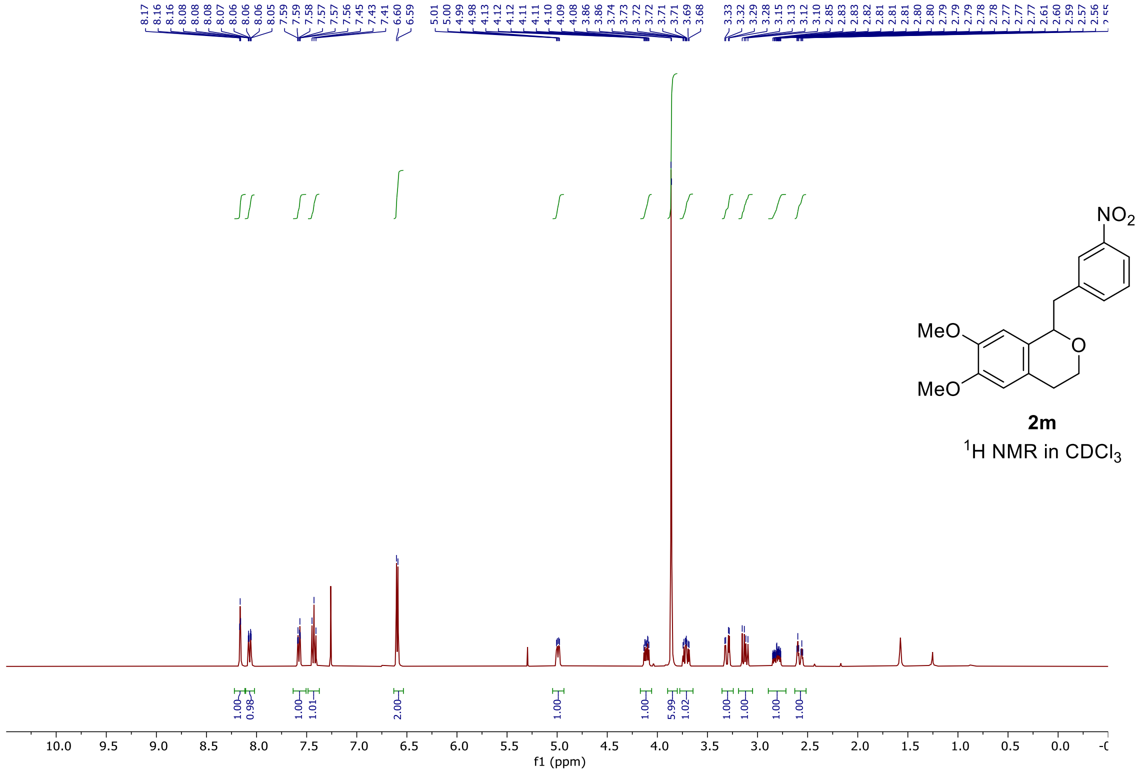

2m

^{1}H NMR in $CDCl_3$

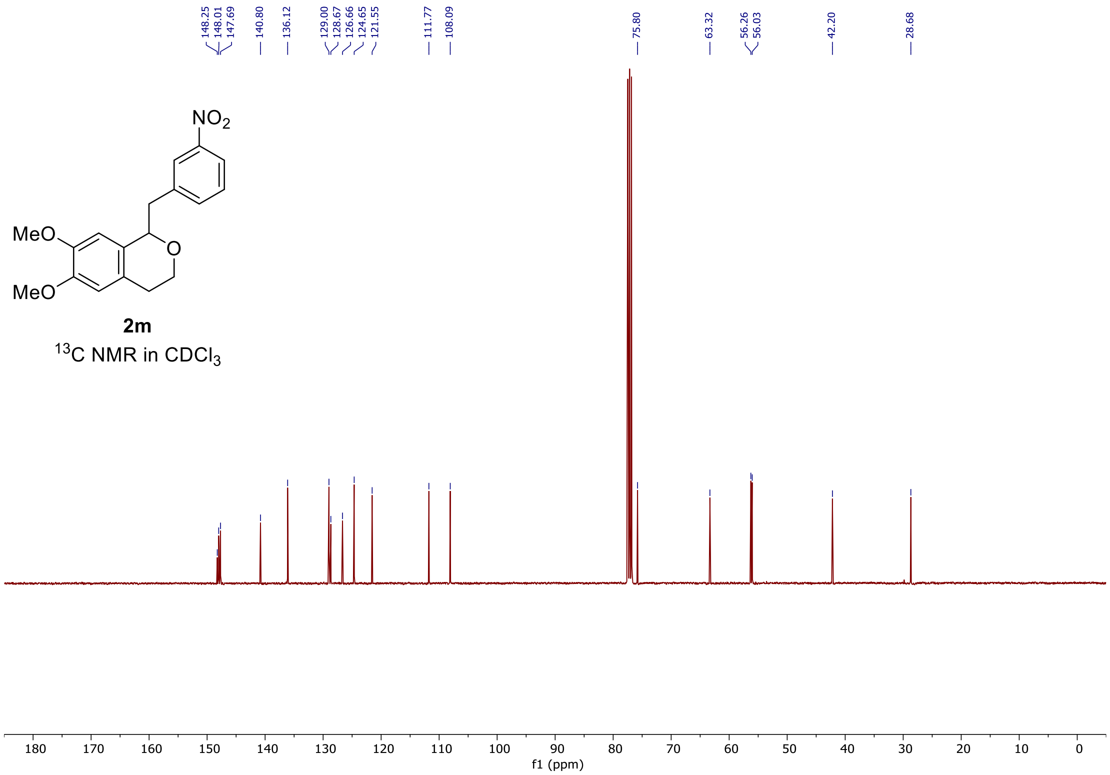

2m

^{13}C NMR in $CDCl_3$

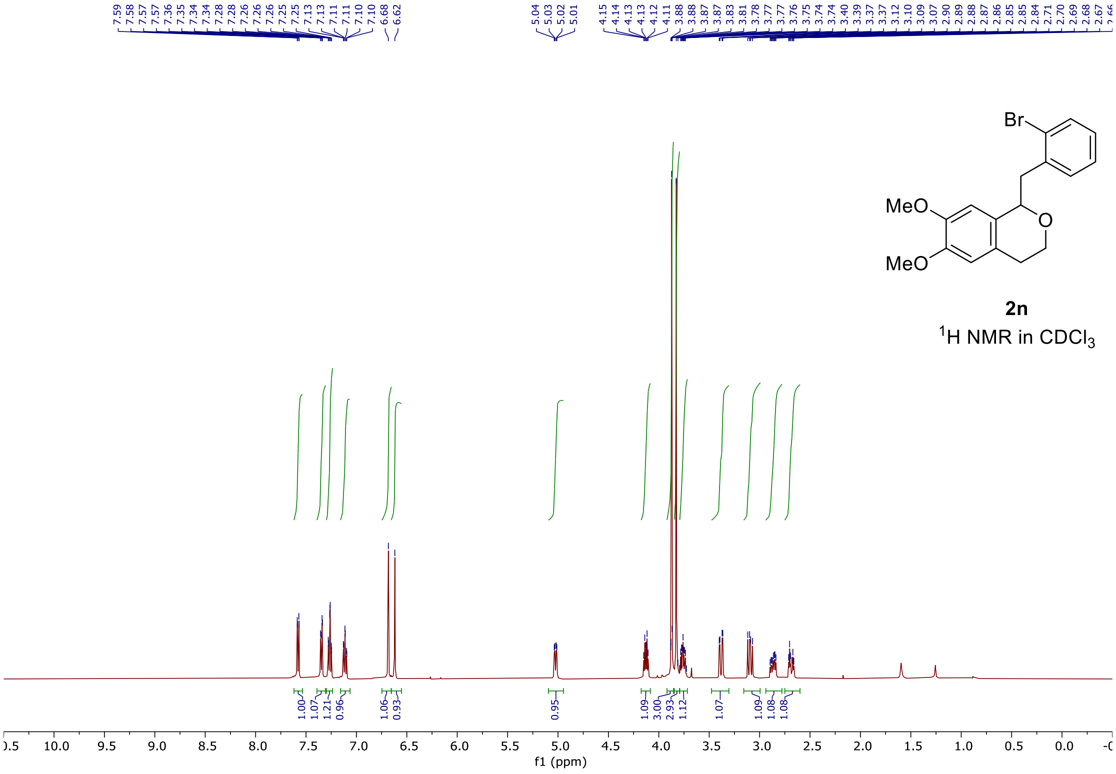

2n

^{1}H NMR in $CDCl_3$

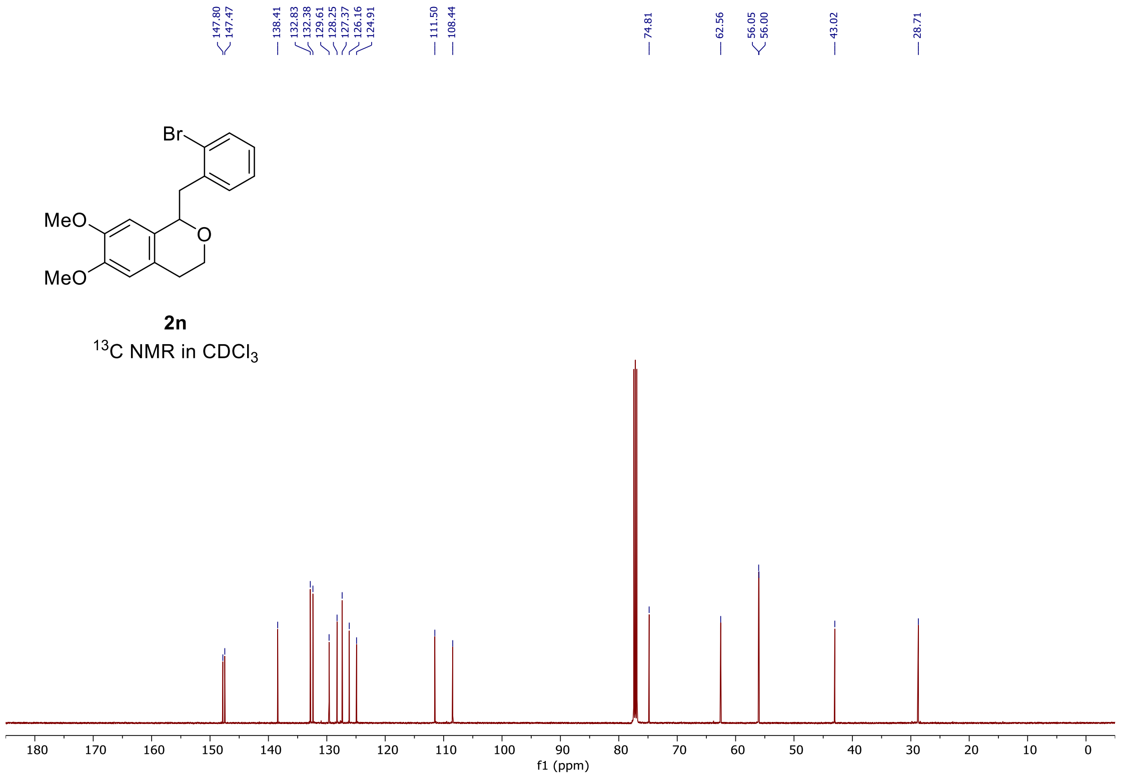

2n
^{13}C NMR in $CDCl_3$

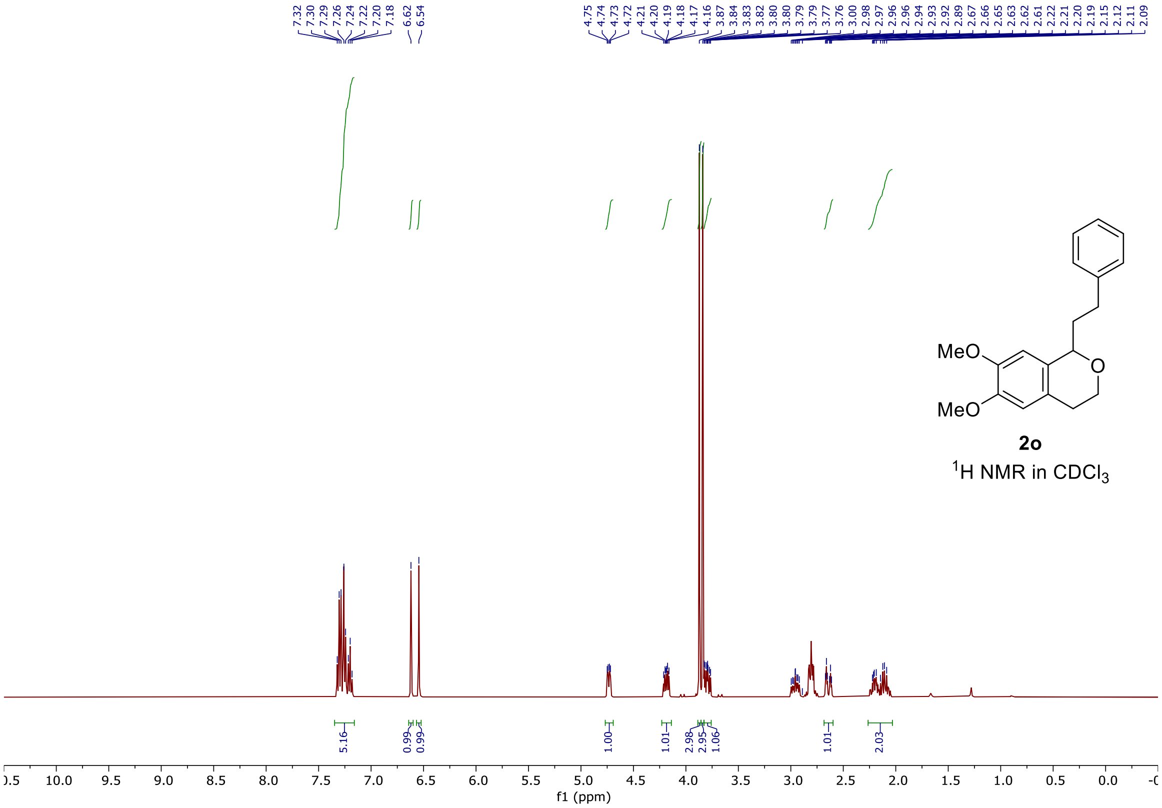

2o
^{1}H NMR in $CDCl_3$

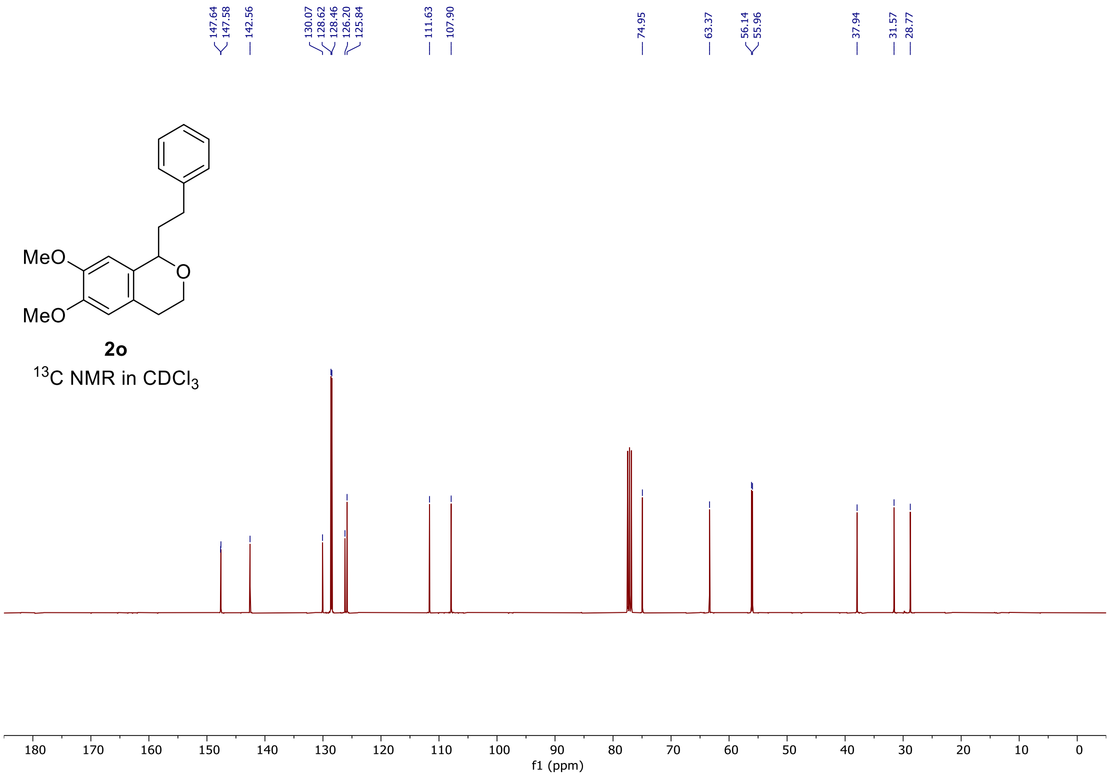
147.64
147.58
142.56
130.07
128.62
128.46
126.20
125.84
111.63
107.90
74.95
63.37
56.14
55.96
37.94
31.57
28.77
MeO
MeO
O
2o
^{13}C NMR in $CDCl_3$
180 170 160 150 140 130 120 110 100 90 80 70 60 50 40 30 20 10 0
f1 (ppm)

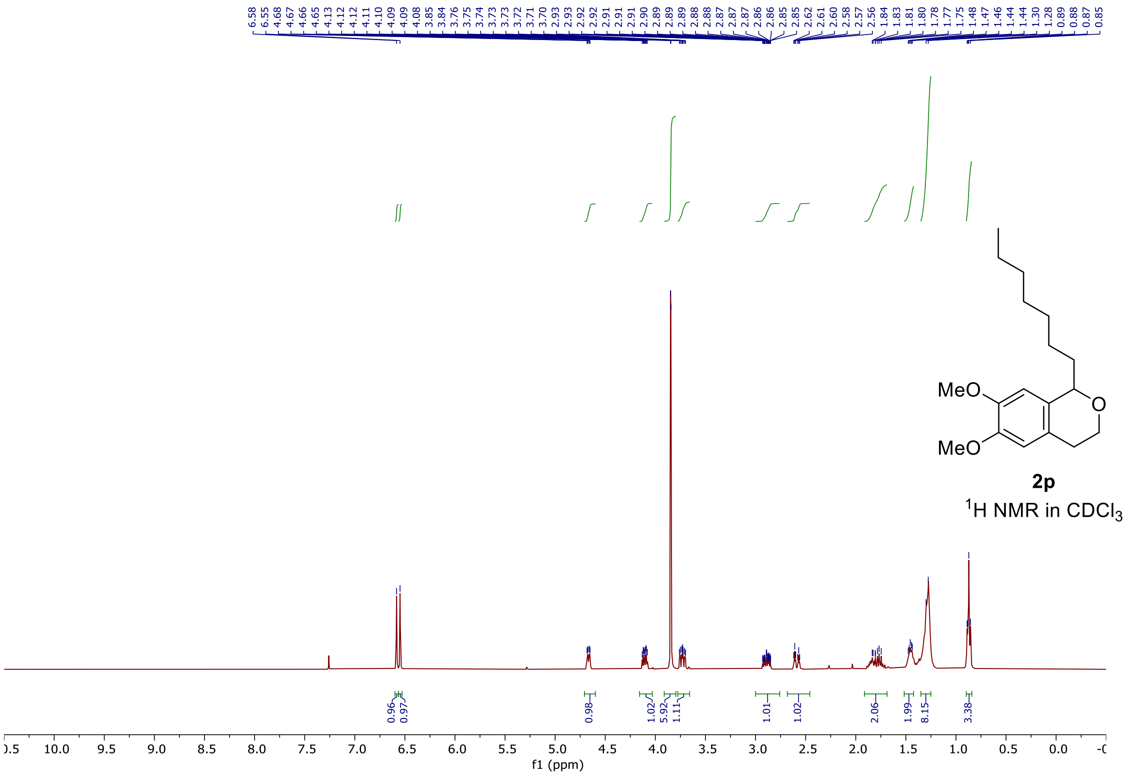
6.58 6.55 4.68 4.67 4.66 4.65 4.13 4.12 4.12 4.11 4.10 4.09 4.08 3.85 3.84 3.76 3.75 3.74 3.73 3.72 3.71 3.70 2.93 2.93 2.92 2.92 2.91 2.91 2.90 2.89 2.89 2.88 2.88 2.87 2.87 2.86 2.86 2.85 2.85 2.62 2.61 2.60 2.58 2.57 2.56 1.84 1.83 1.81 1.80 1.78 1.77 1.75 1.48 1.47 1.46 1.44 1.44 1.30 1.28 0.89 0.88 0.87 0.85
MeO
MeO
O
2p
^{1}H NMR in $CDCl_3$
0.96
0.97
0.98
1.02
5.92
1.11
1.01
1.02
2.06
1.99
8.15
3.38
10.0 9.5 9.0 8.5 8.0 7.5 7.0 6.5 6.0 5.5 5.0 4.5 4.0 3.5 3.0 2.5 2.0 1.5 1.0 0.5 0.0
f1 (ppm)

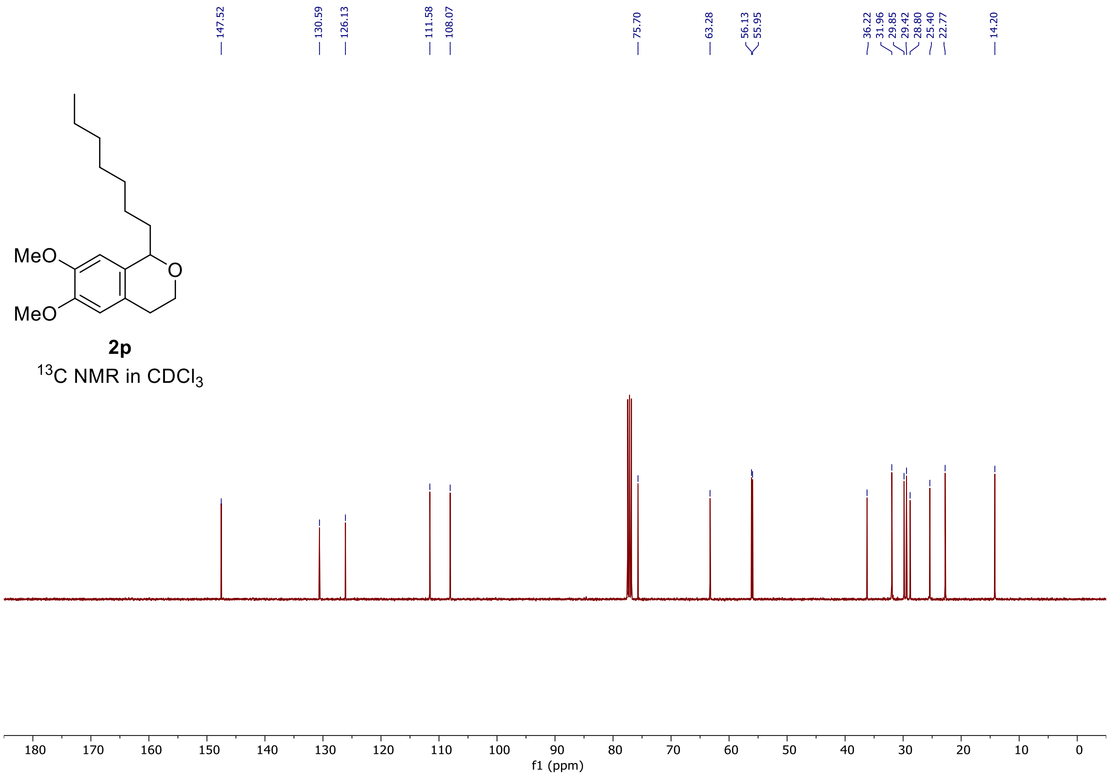

2p
^{13}C NMR in $CDCl_3$

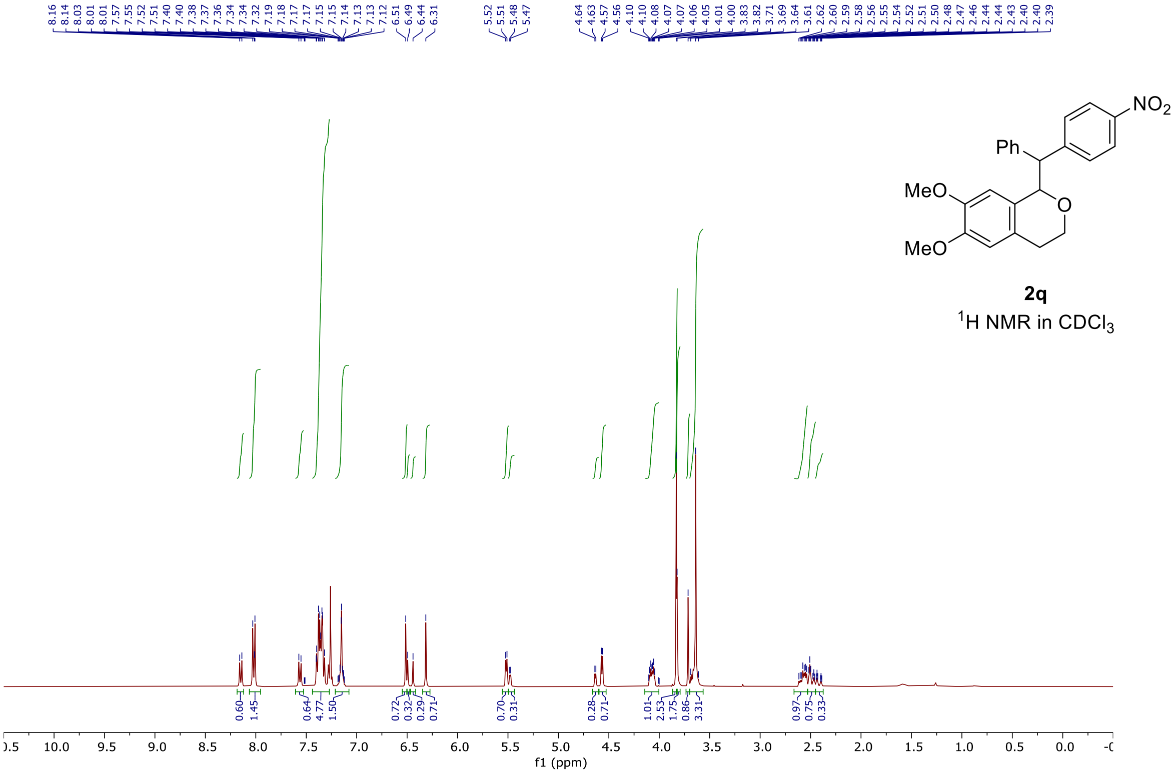

2q
^{1}H NMR in $CDCl_3$

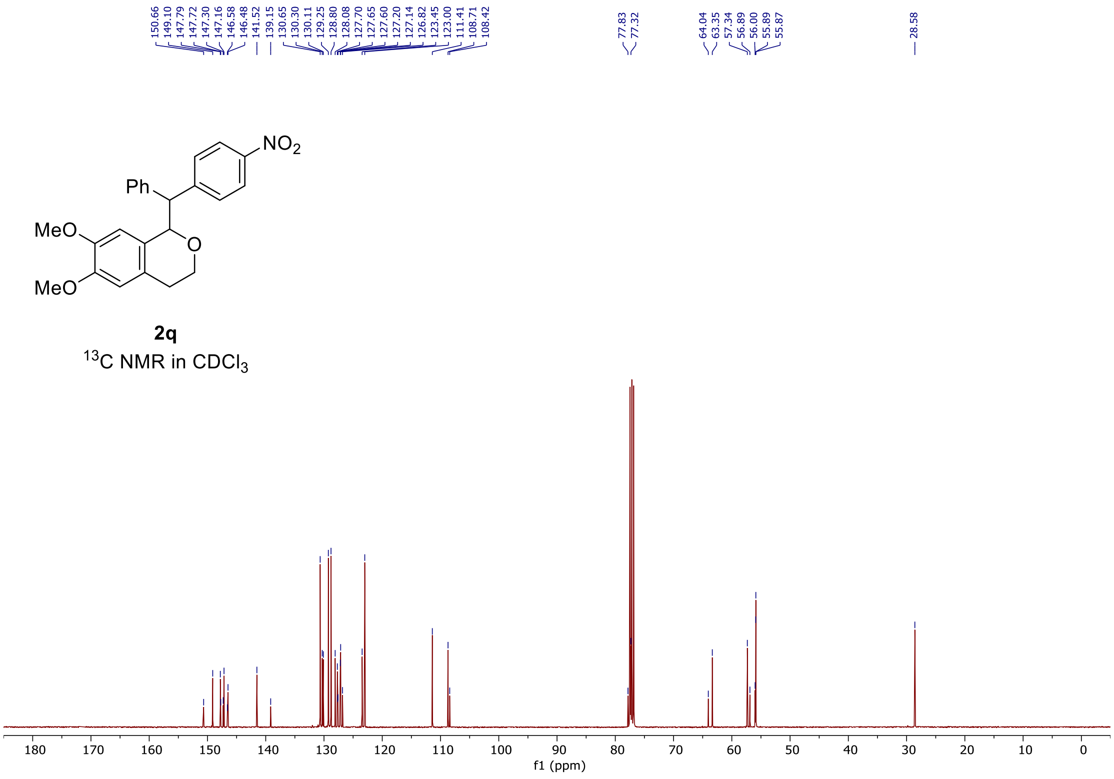
150.66 149.10 147.79 147.72 147.30 147.16 146.58 146.48 141.52 139.15 130.65 130.30 130.11 129.25 128.80 128.08 127.70 127.65 127.60 127.20 127.14 126.82 123.45 123.00 111.41 108.71 108.42
77.83 77.32
64.04 63.35 57.34 56.89 56.00 55.89 55.87
28.58
Ph
NO2
MeO
MeO
O
2q
13C NMR in CDCl3
180 170 160 150 140 130 120 110 100 90 80 70 60 50 40 30 20 10 0
f1 (ppm)

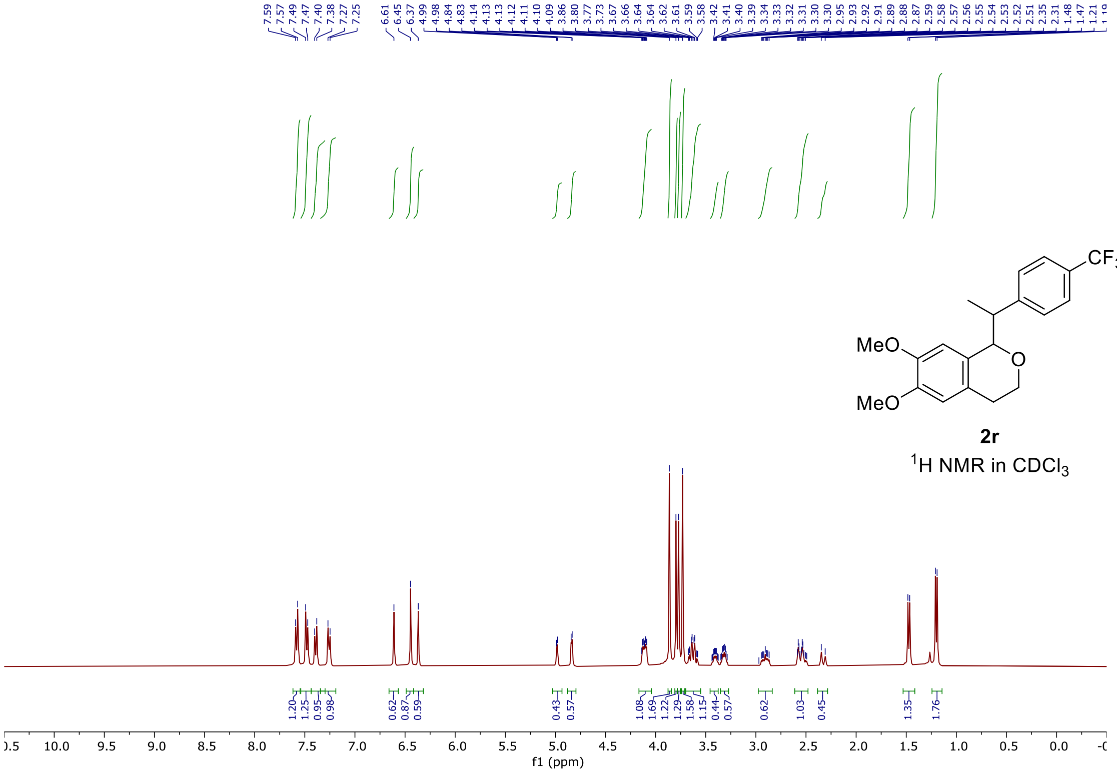
7.59 7.57 7.49 7.47 7.40 7.38 7.27 7.25 6.61 6.45 6.37 4.99 4.98 4.84 4.83 4.14 4.13 4.13 4.12 4.11 4.10 4.09 3.86 3.80 3.77 3.73 3.67 3.66 3.64 3.64 3.62 3.61 3.59 3.58 3.42 3.41 3.40 3.39 3.34 3.33 3.32 3.31 3.30 3.30 2.95 2.93 2.92 2.91 2.89 2.88 2.87 2.59 2.58 2.57 2.56 2.55 2.54 2.53 2.52 2.51 2.35 2.31 1.48 1.47 1.21
CF3
MeO
MeO
O
2r
1H NMR in CDCl3
1.20 1.25 0.95 0.98 0.62 0.87 0.59 0.43 0.57 1.08 1.69 1.22 1.29 1.58 1.15 0.44 0.57 0.62 1.03 0.45 1.35 1.76
10.0 9.5 9.0 8.5 8.0 7.5 7.0 6.5 6.0 5.5 5.0 4.5 4.0 3.5 3.0 2.5 2.0 1.5 1.0 0.5 0.0
f1 (ppm)

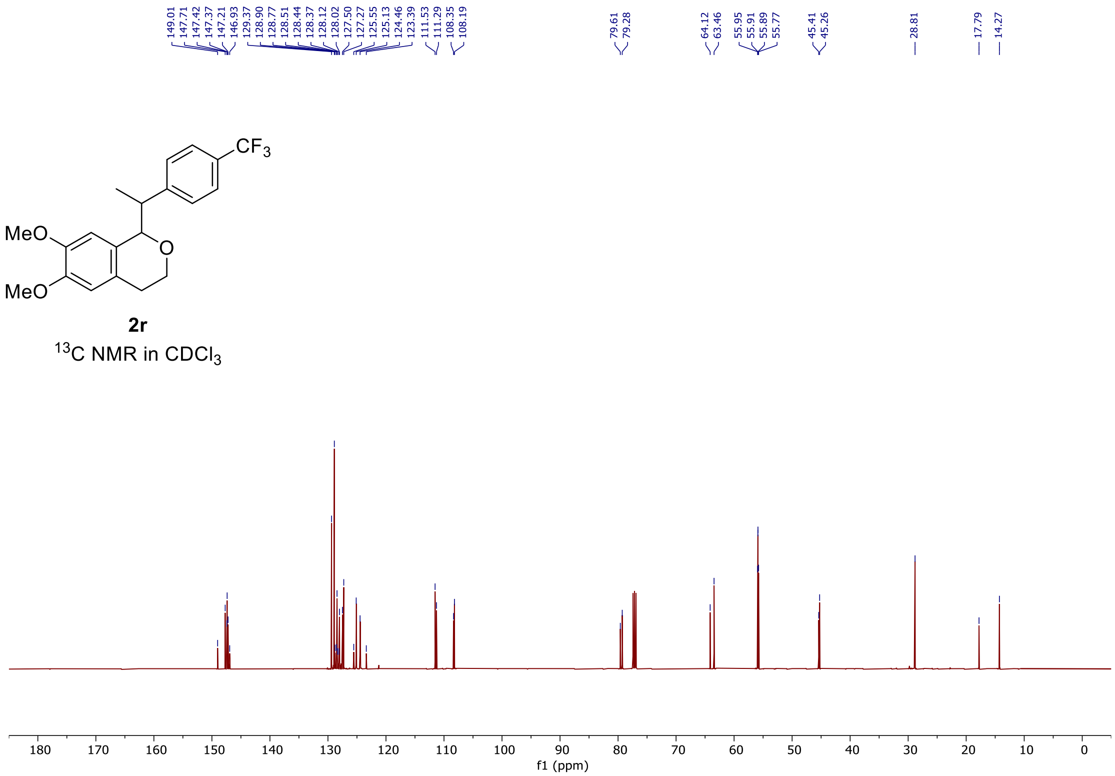
149.01
147.71
147.42
147.37
147.21
146.93
129.37
129.37
128.90
128.77
128.51
128.44
128.37
128.12
128.02
127.50
127.27
125.55
125.13
124.46
123.39
111.53
111.29
108.35
108.19
79.61
79.28
64.12
63.46
55.95
55.91
55.89
55.77
45.41
45.26
28.81
17.79
14.27
CF3
MeO
MeO
O
2r
13C NMR in CDCl3
f1 (ppm)

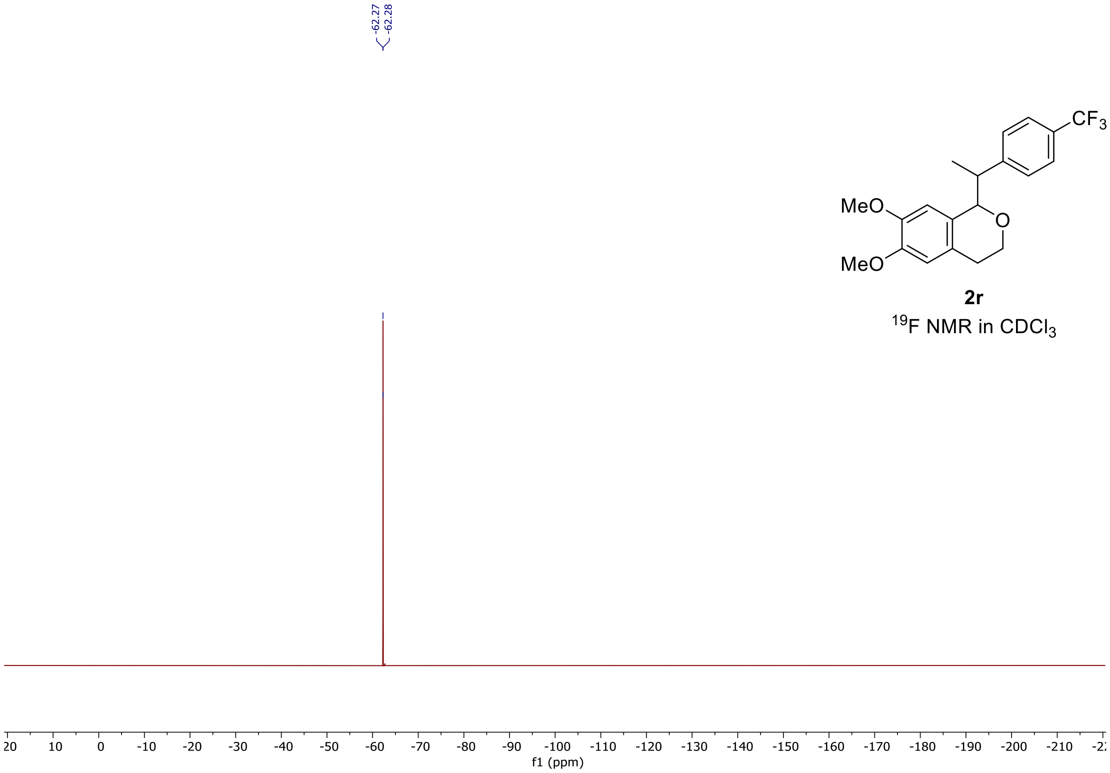
-62.27
-62.28
CF3
MeO
MeO
O
2r
19F NMR in CDCl3
f1 (ppm)

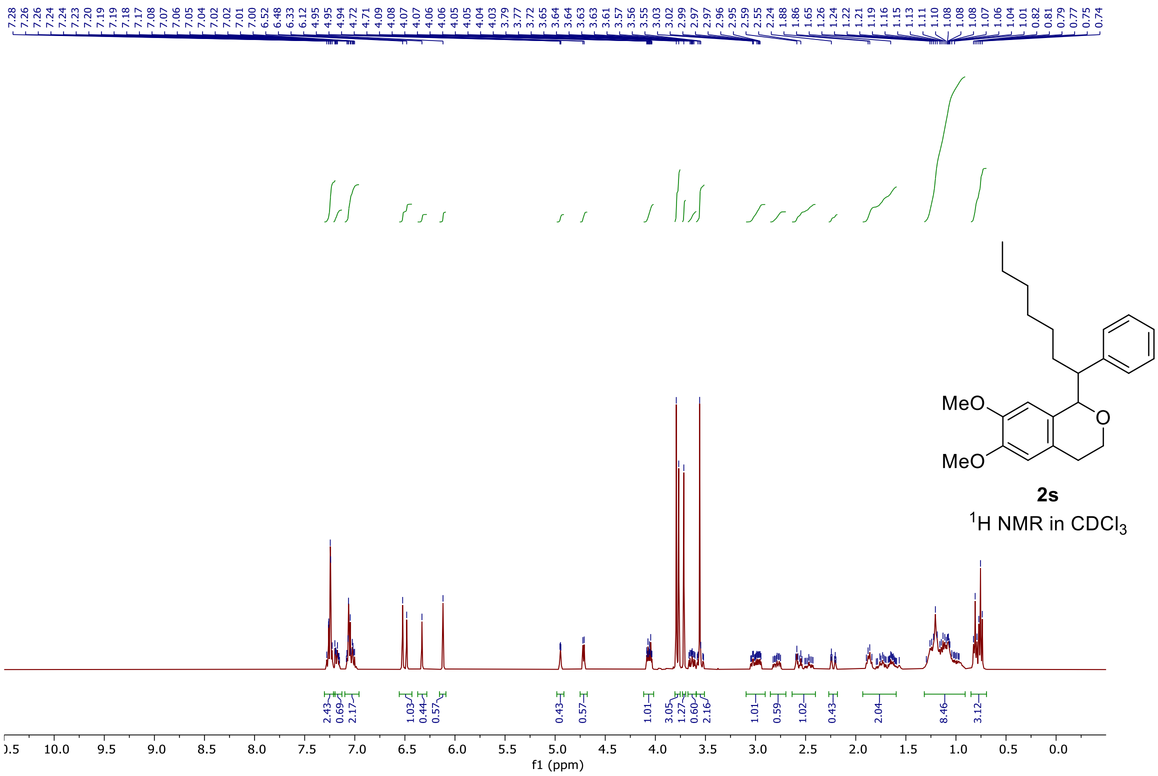

2s

^{1}H NMR in $CDCl_3$

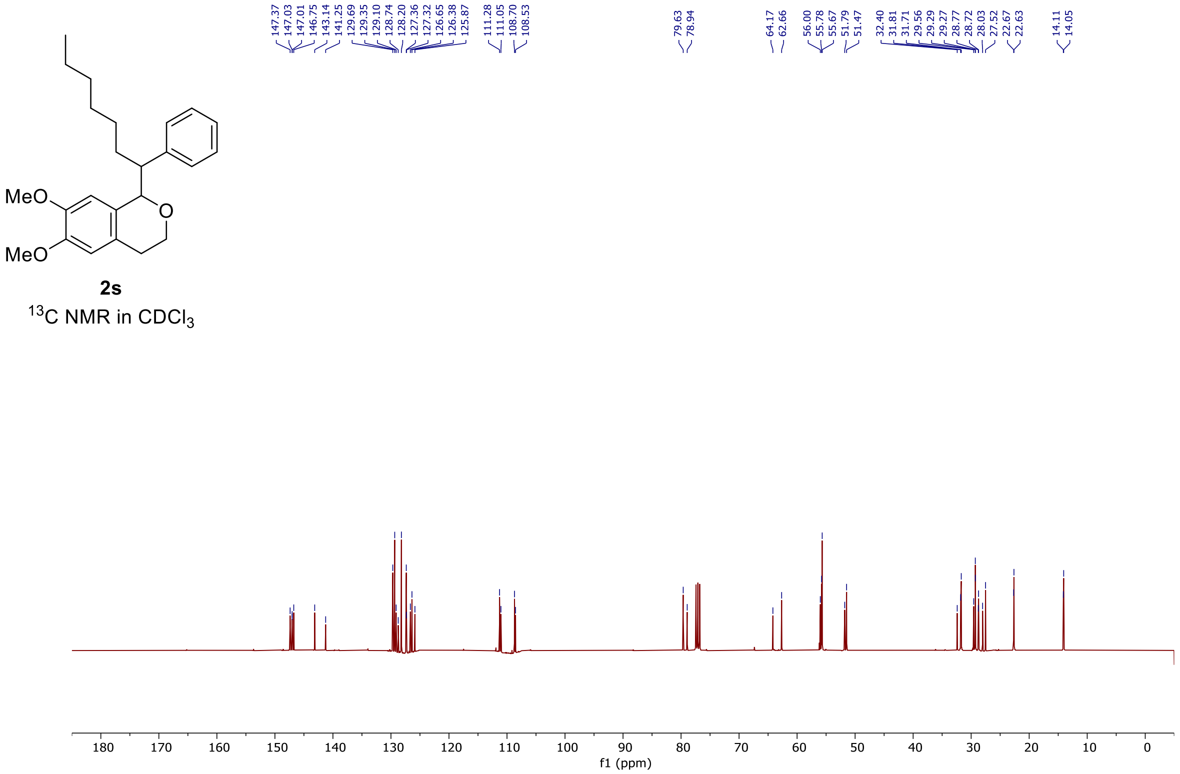

2s

^{13}C NMR in $CDCl_3$

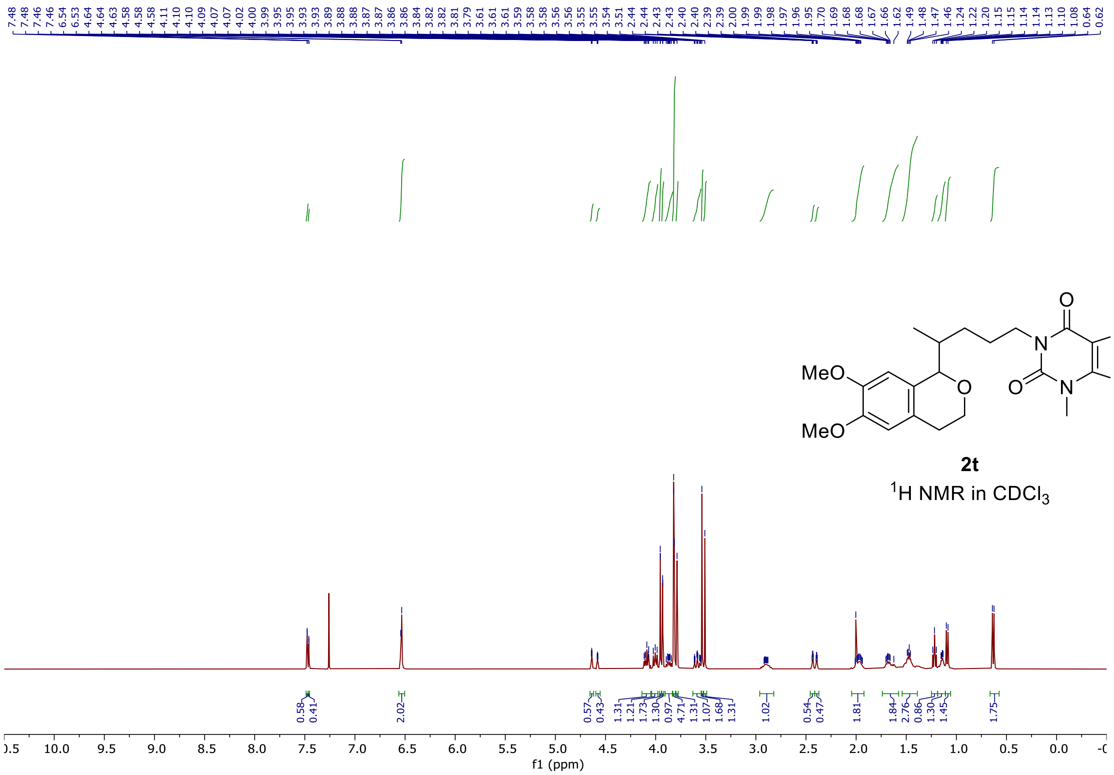

2t

^{1}H NMR in $CDCl_3$

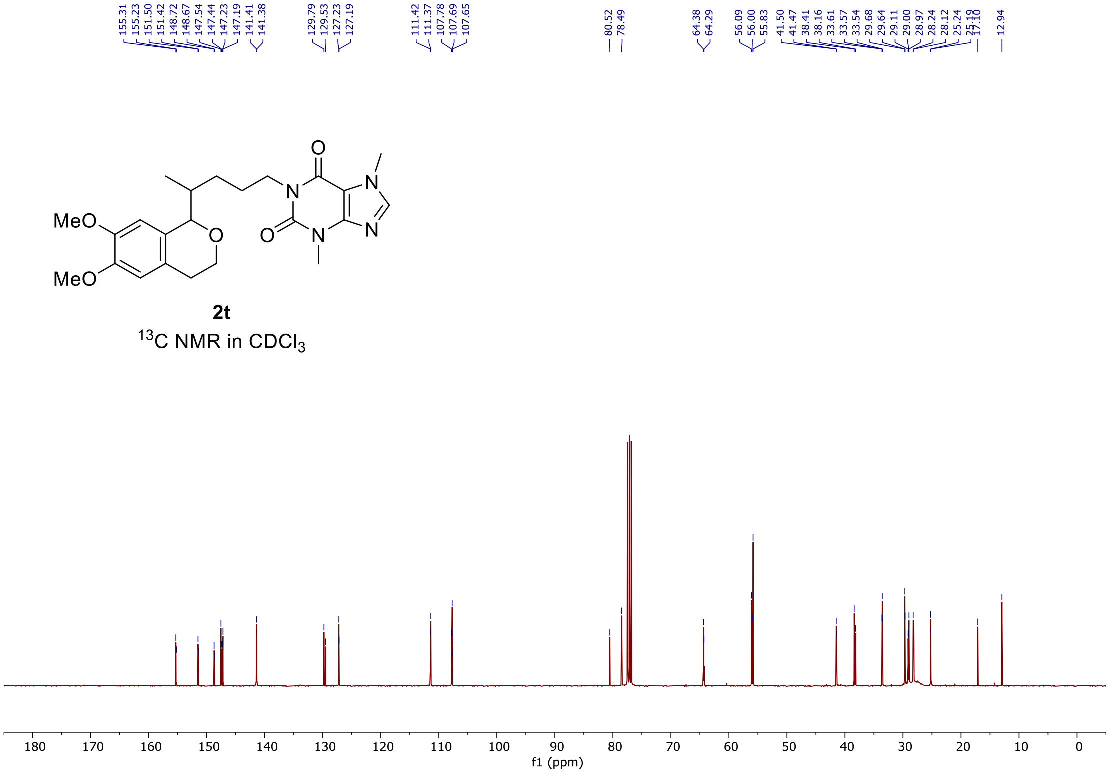

2t

^{13}C NMR in $CDCl_3$

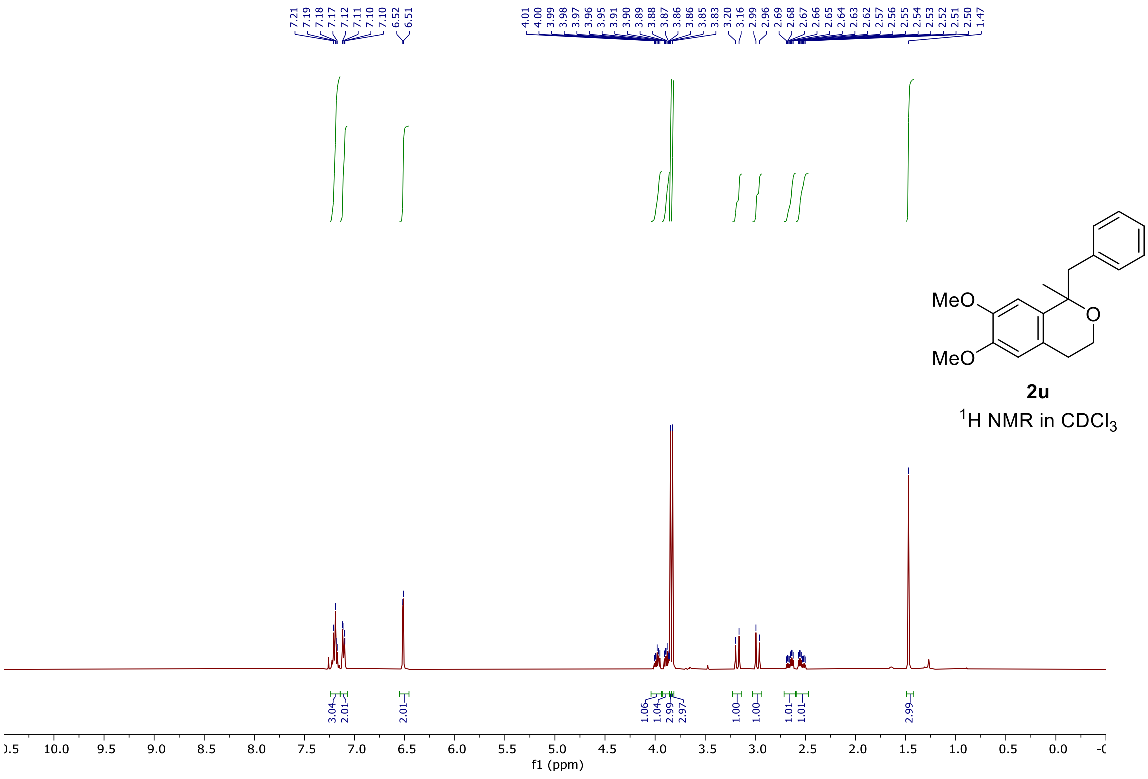
7.21 7.19 7.18 7.17 7.12 7.11 7.10 6.52 6.51
4.01 4.00 3.99 3.98 3.97 3.96 3.95 3.91 3.90 3.89 3.88 3.87 3.86 3.86 3.85 3.83 3.20 3.16 2.99 2.96 2.69 2.68 2.67 2.66 2.65 2.64 2.63 2.62 2.57 2.56 2.55 2.54 2.53 2.52 2.51 2.50 1.47
MeO
MeO
O
2u
1H NMR in CDCl3
3.04 2.01 2.01 1.06 1.04 2.99 2.97 1.00 1.00 1.01 1.01 2.99
f1 (ppm)

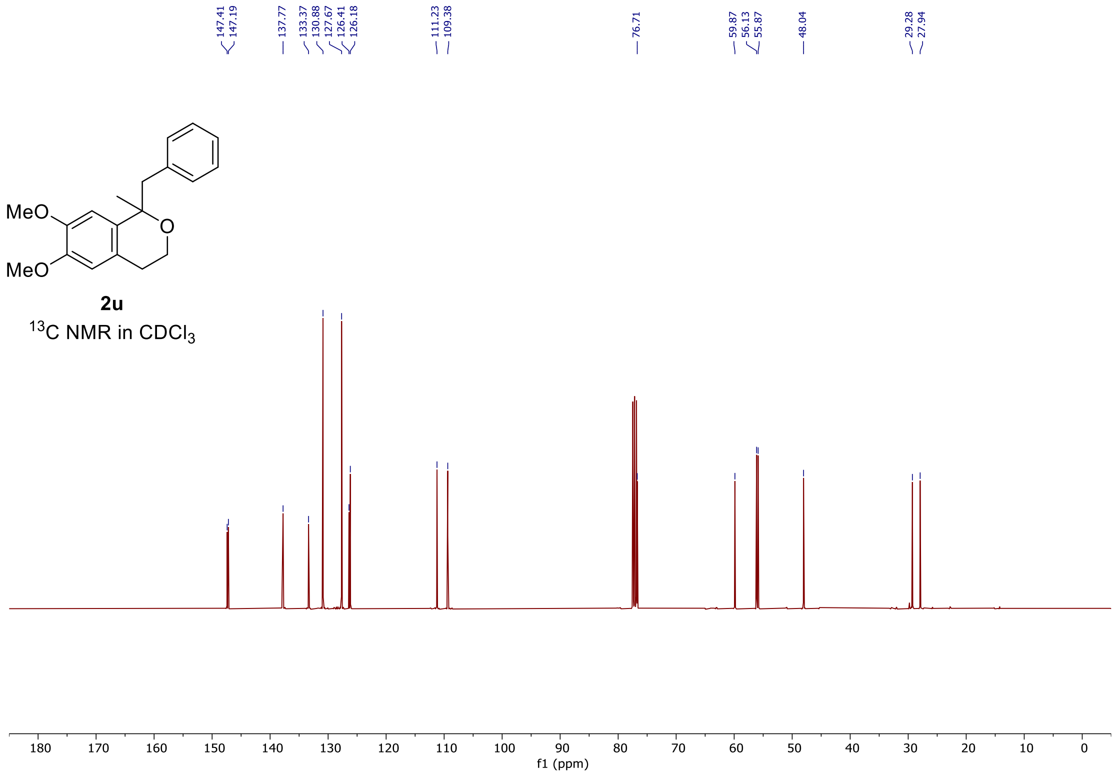
147.41 147.19 137.77 133.37 130.88 127.67 126.41 126.18 111.23 109.38 76.71 59.87 56.13 55.87 48.04 29.28 27.94
MeO
MeO
O
2u
13C NMR in CDCl3
f1 (ppm)

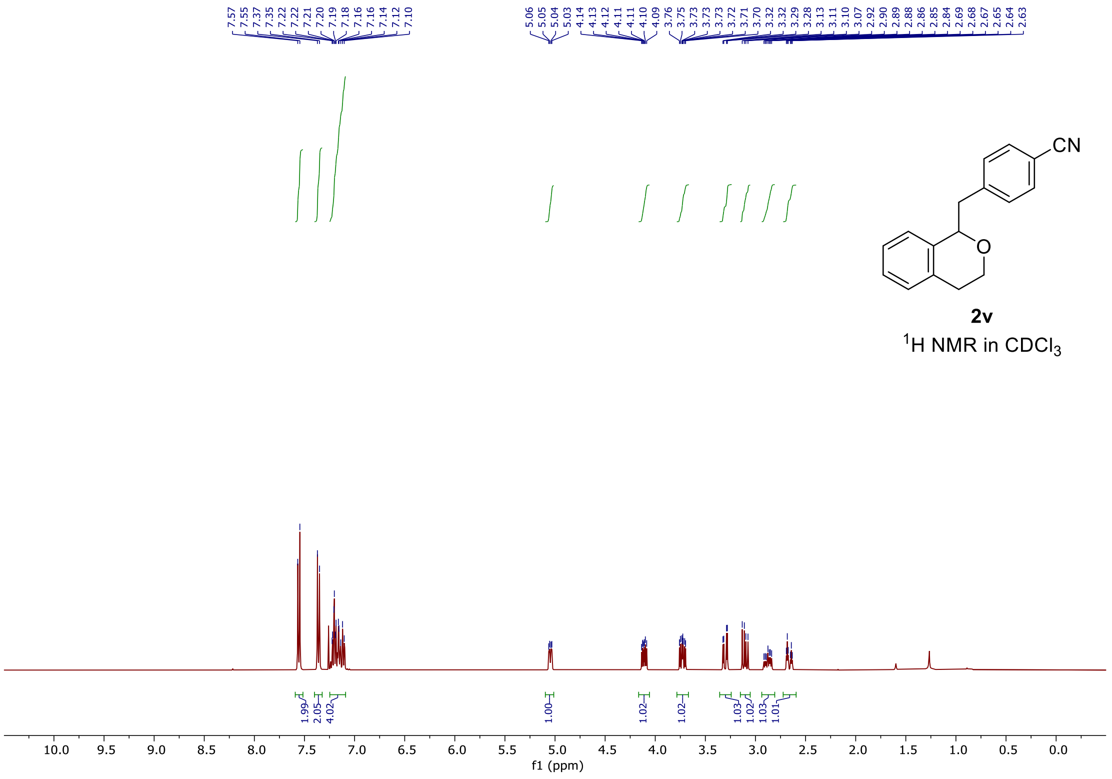

2v
^1H NMR in $CDCl_3$

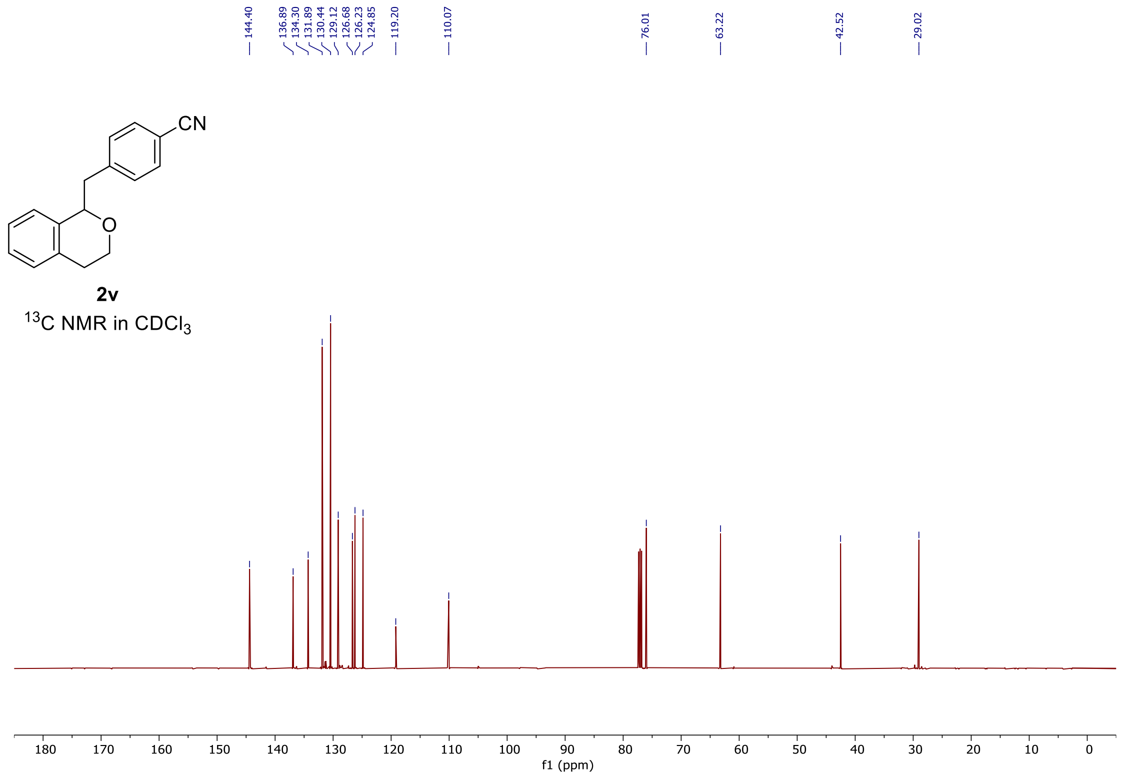

2v
^{13}C NMR in $CDCl_3$

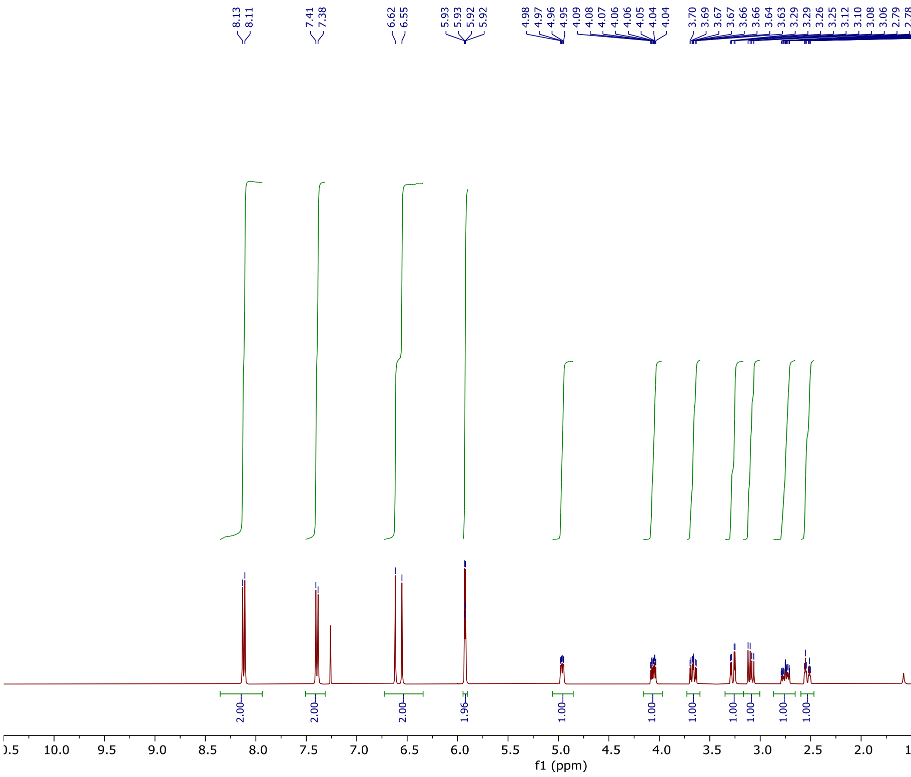

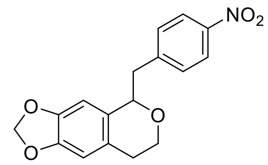

2w

^{1}H NMR in $CDCl_3$

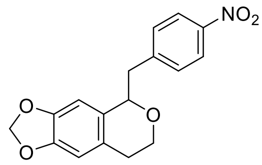

2w

^{13}C NMR in $CDCl_3$

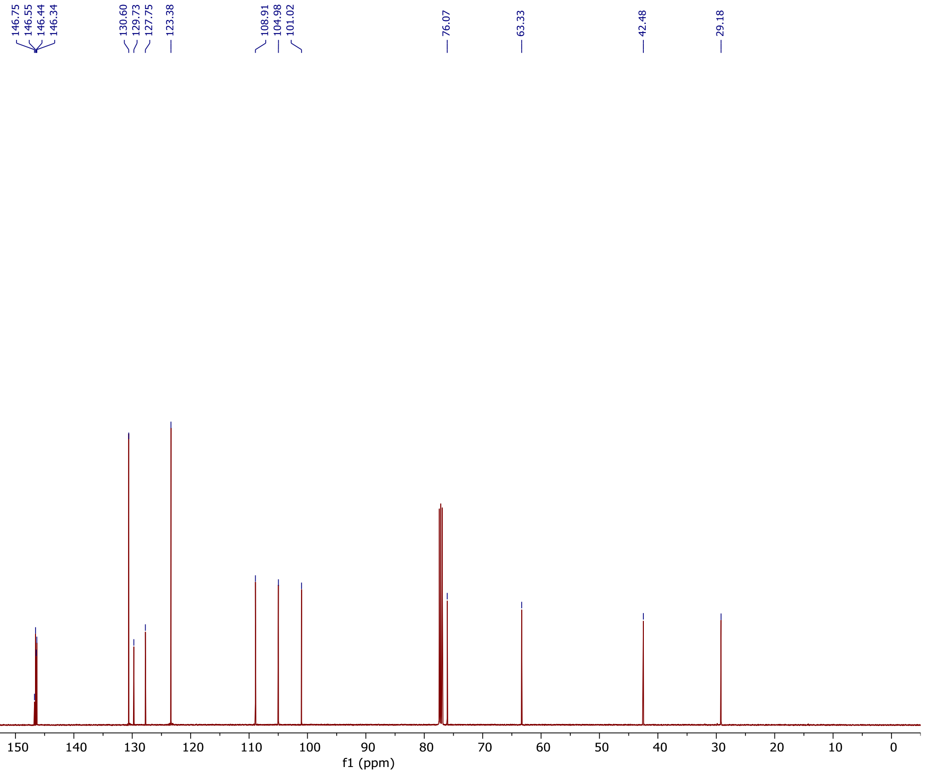

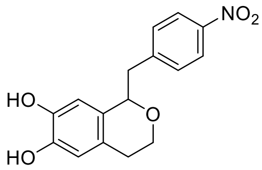

2x

^{1}H NMR in acetone-d_6

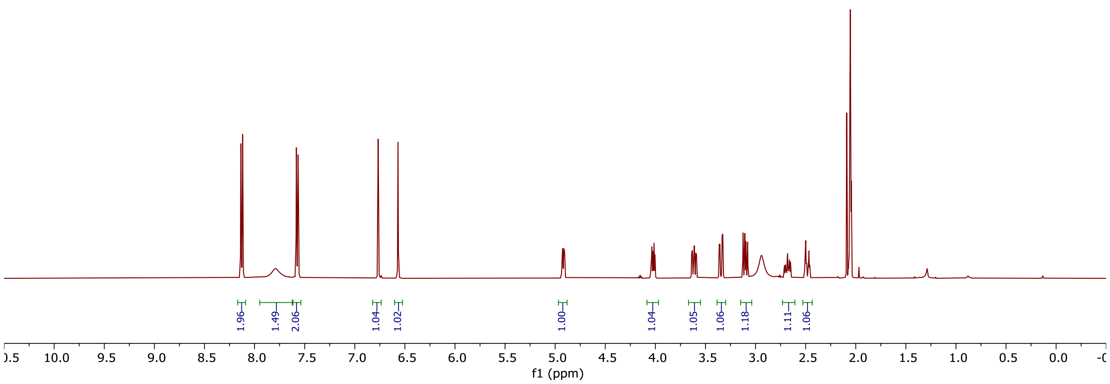

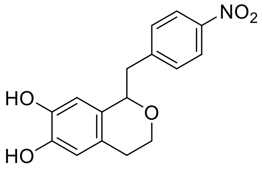

2x

^{13}C NMR in acetone-d_6

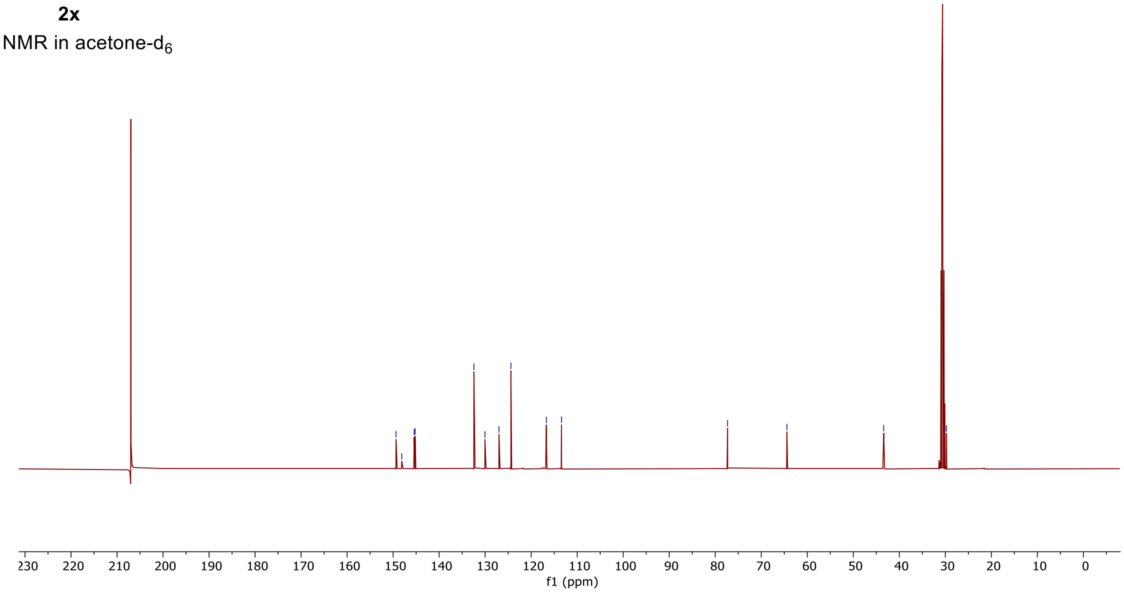

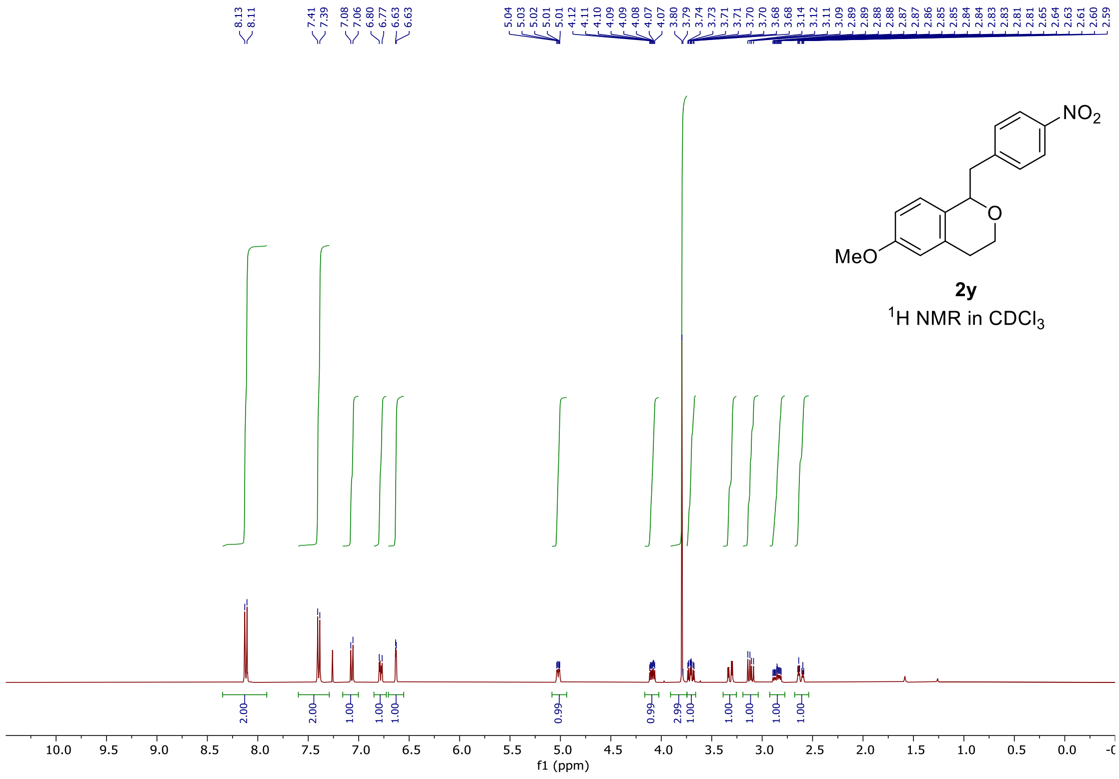

2y

^{1}H NMR in $CDCl_3$

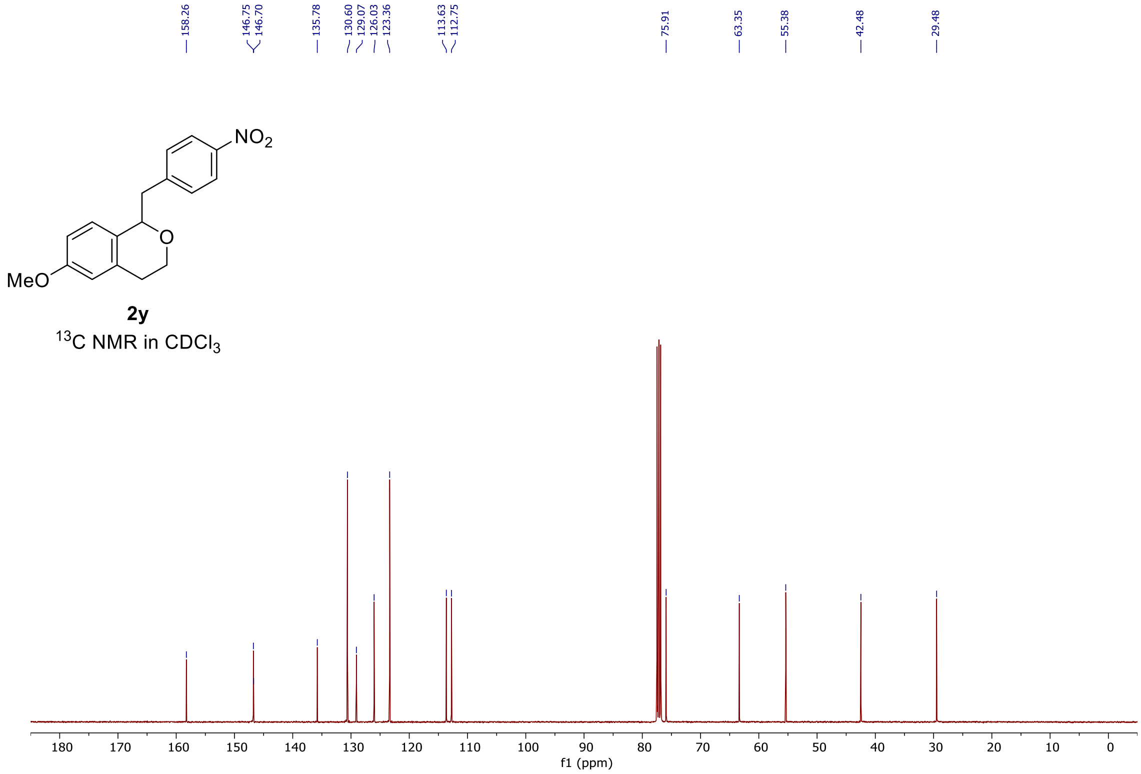

2y

^{13}C NMR in $CDCl_3$

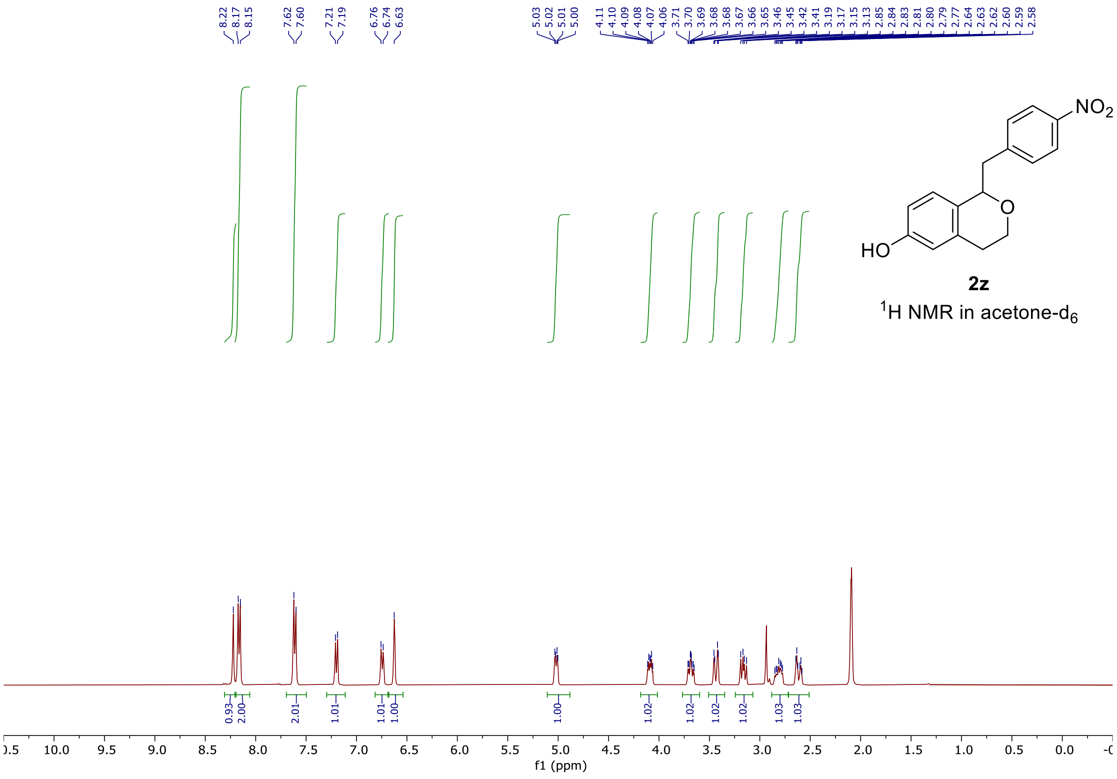

2z

^1H NMR in acetone-d_6

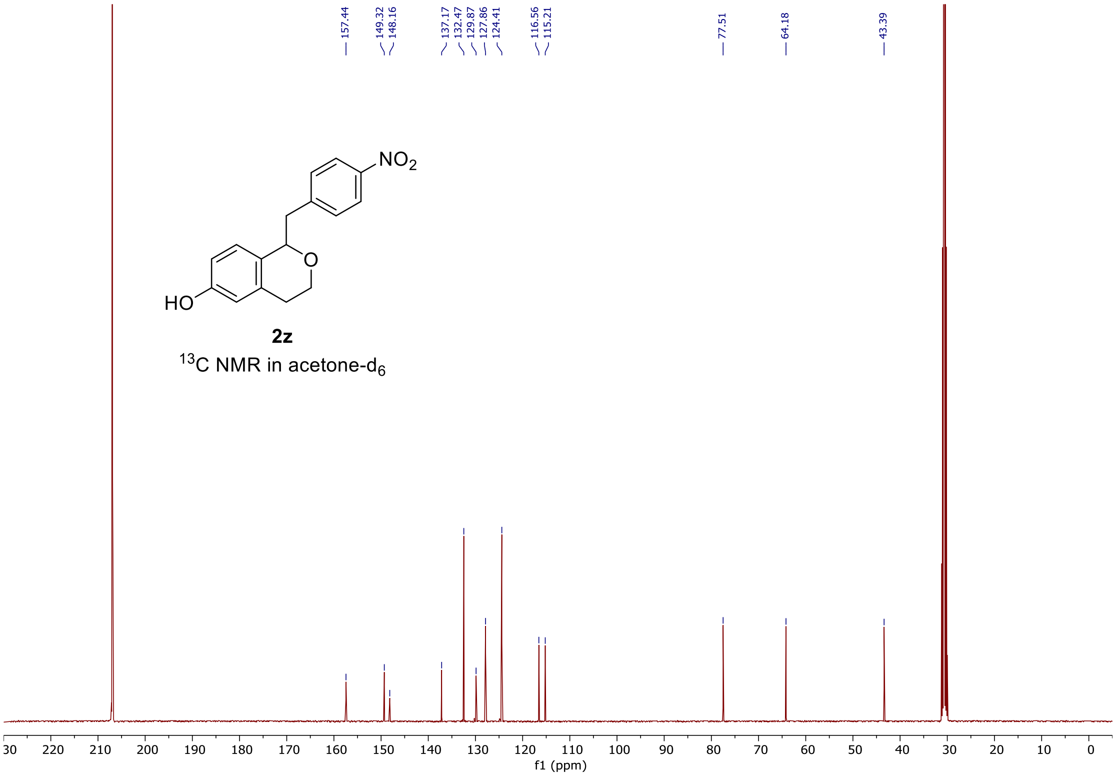

2z

^{13}C NMR in acetone-d_6

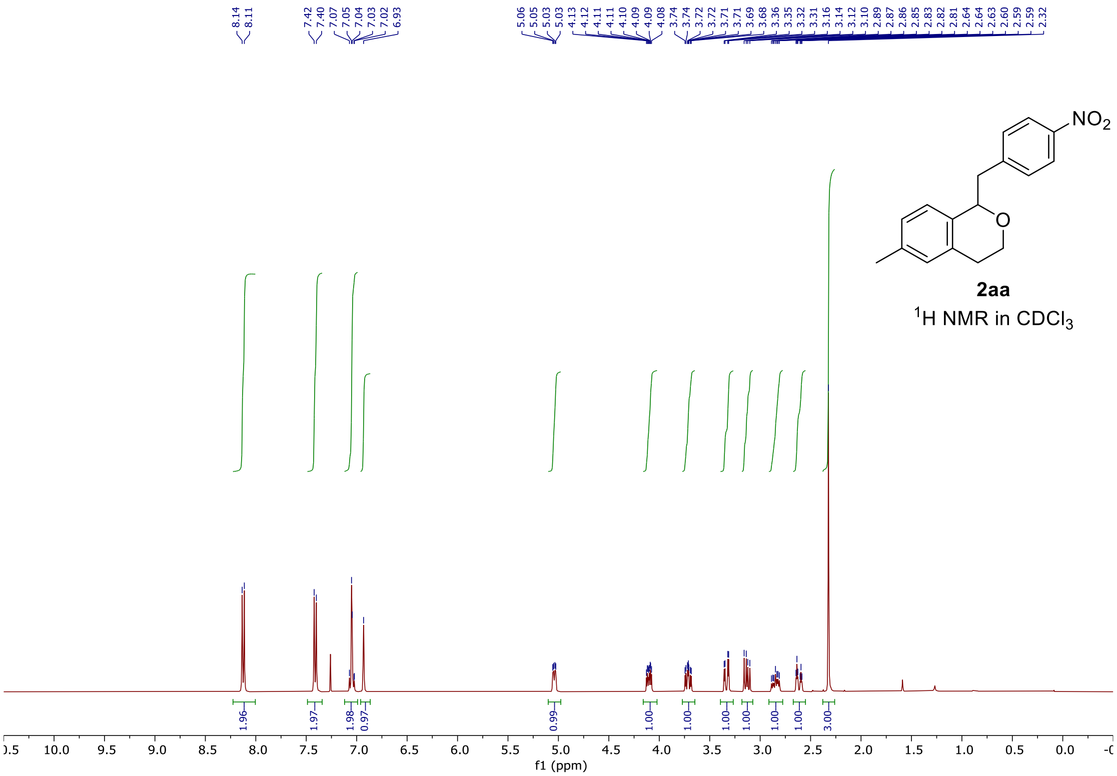

2aa

^{1}H NMR in $CDCl_3$

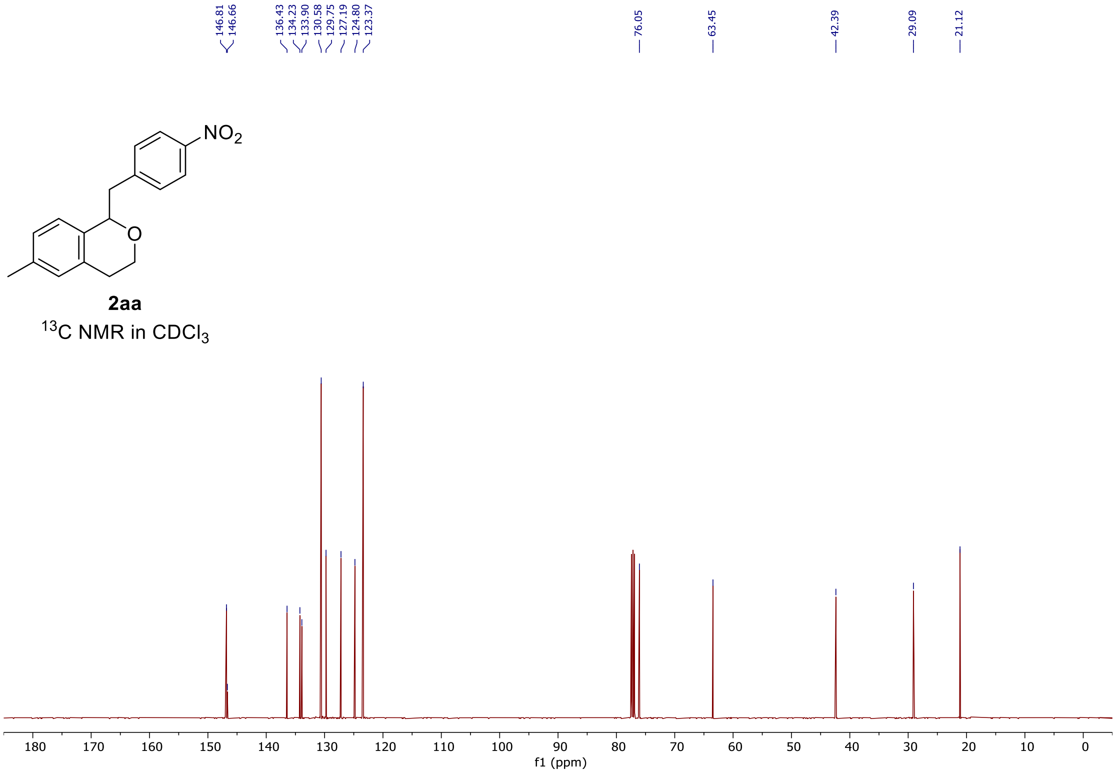

2aa

^{13}C NMR in $CDCl_3$

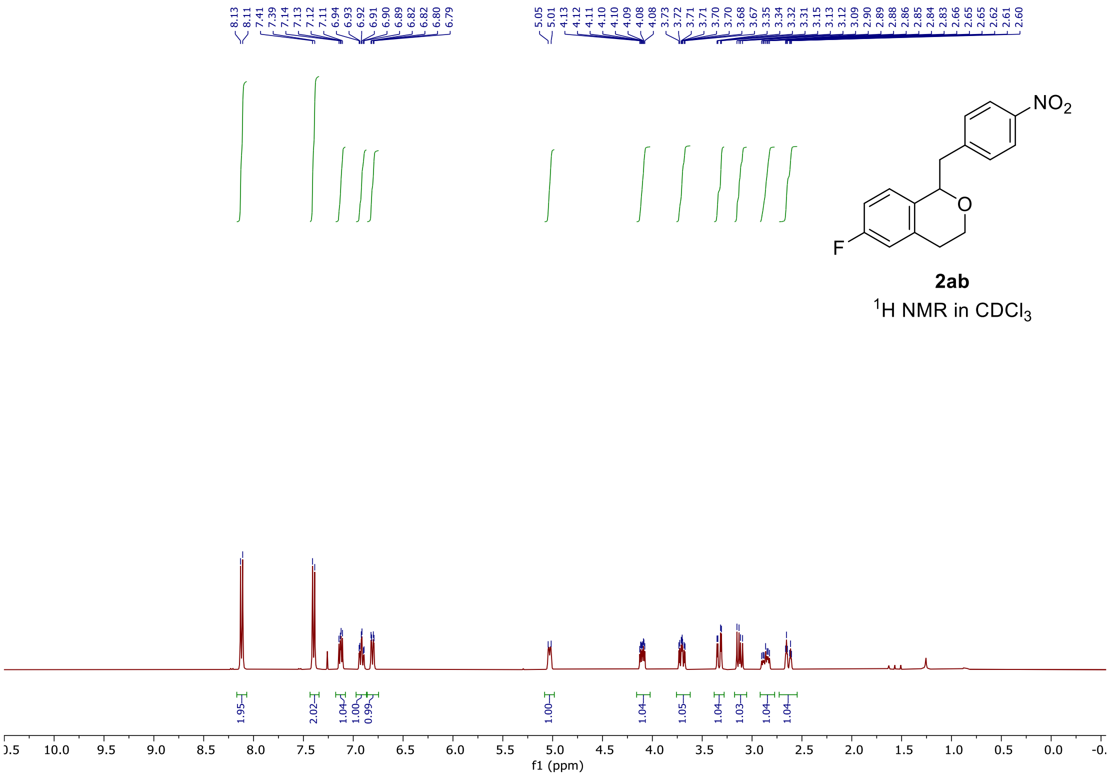

2ab

^{1}H NMR in $CDCl_3$

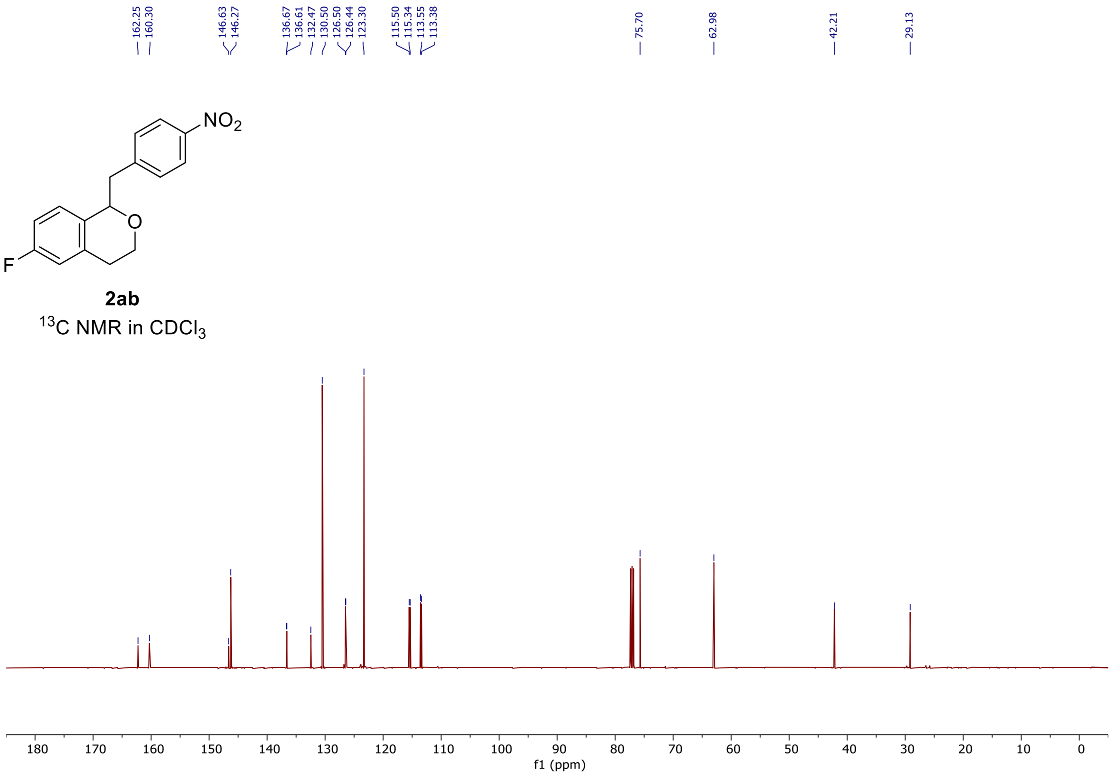

2ab

^{13}C NMR in $CDCl_3$

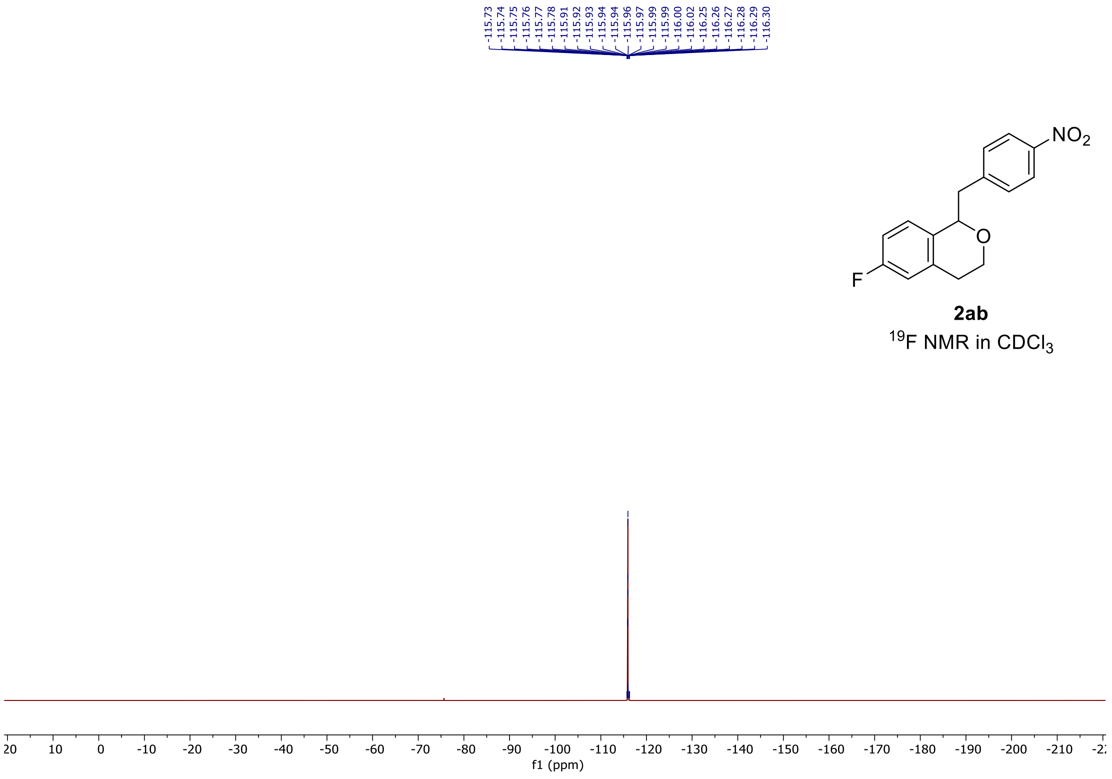

2ab

^{19}F NMR in $CDCl_3$

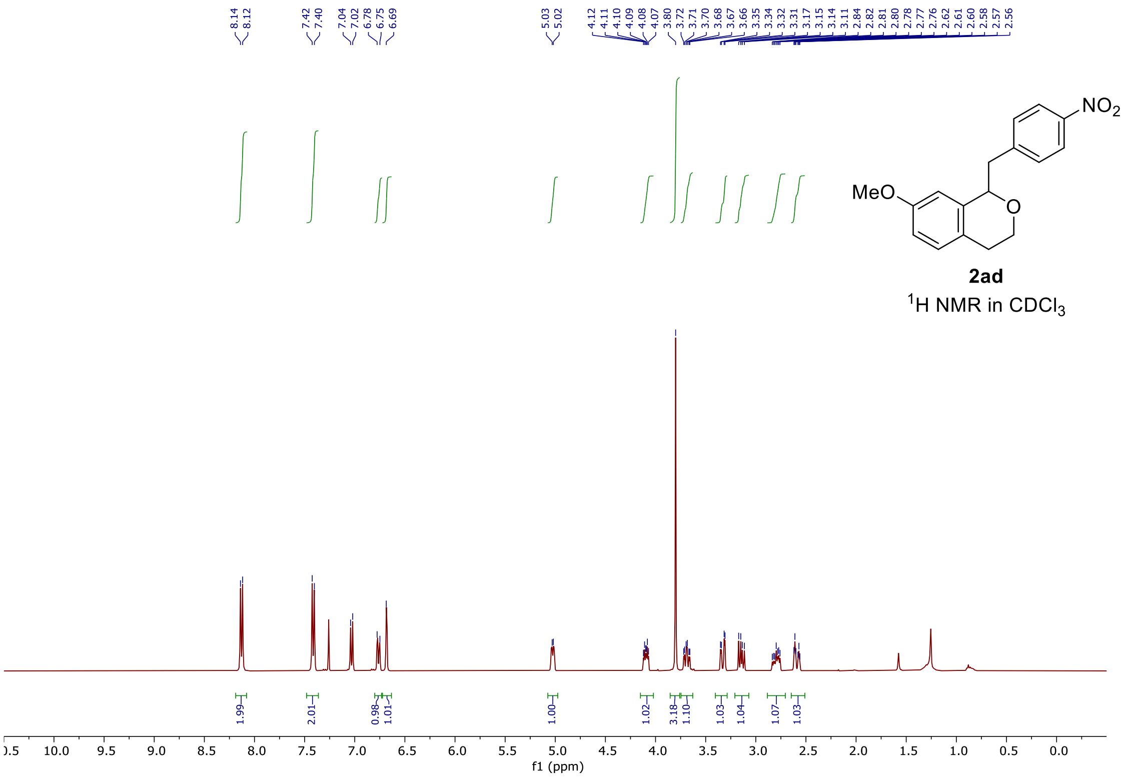

2ad

^{1}H NMR in $CDCl_3$

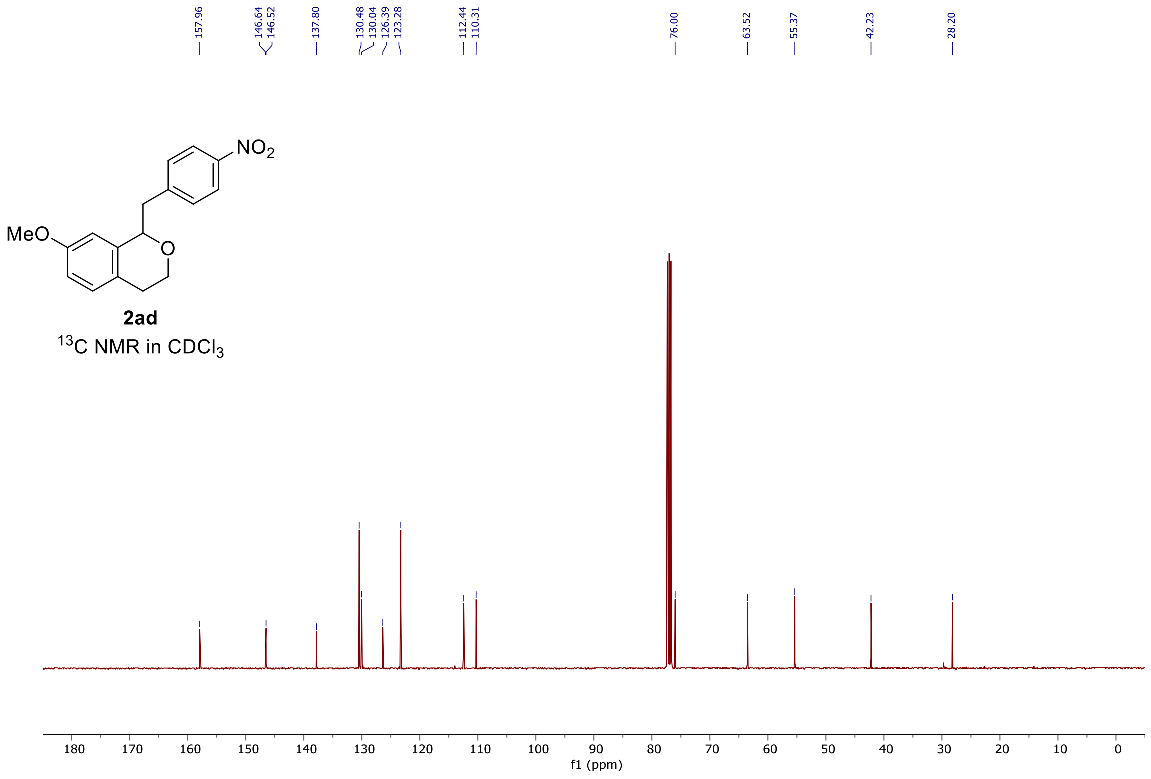

2ad

^{13}C NMR in $CDCl_3$

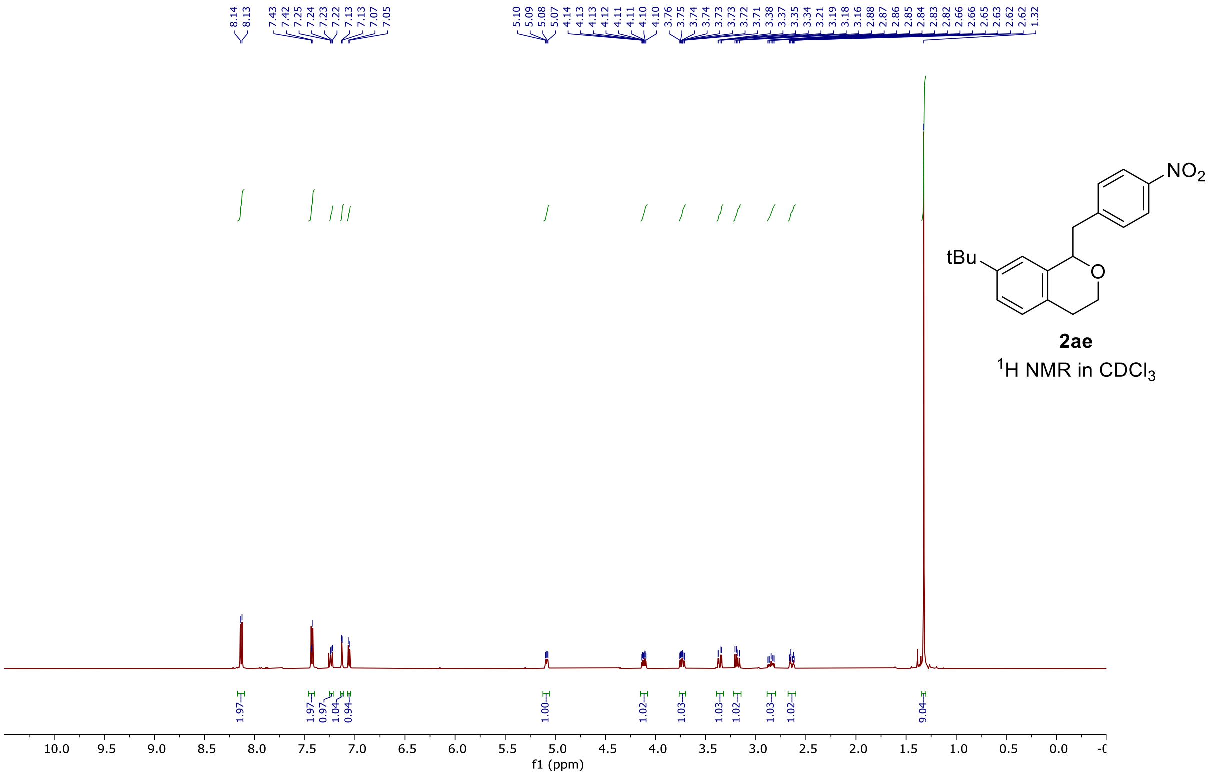

2ae

^{1}H NMR in $CDCl_3$

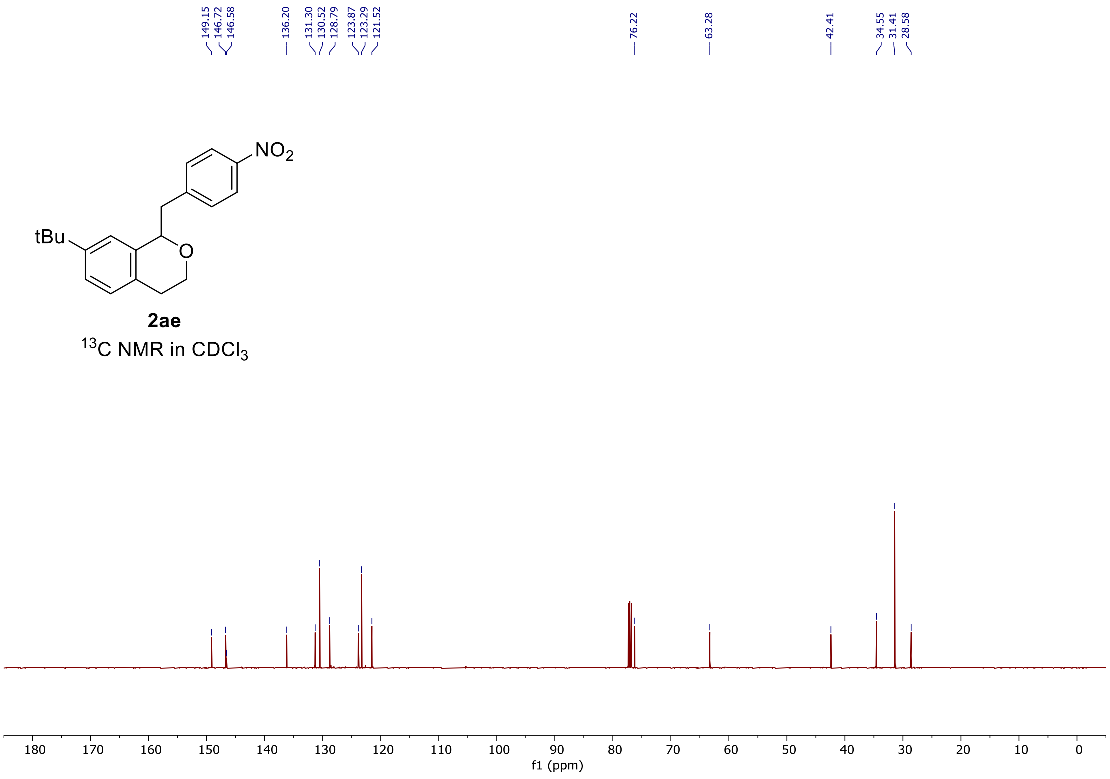

2ae

^{13}C NMR in $CDCl_3$

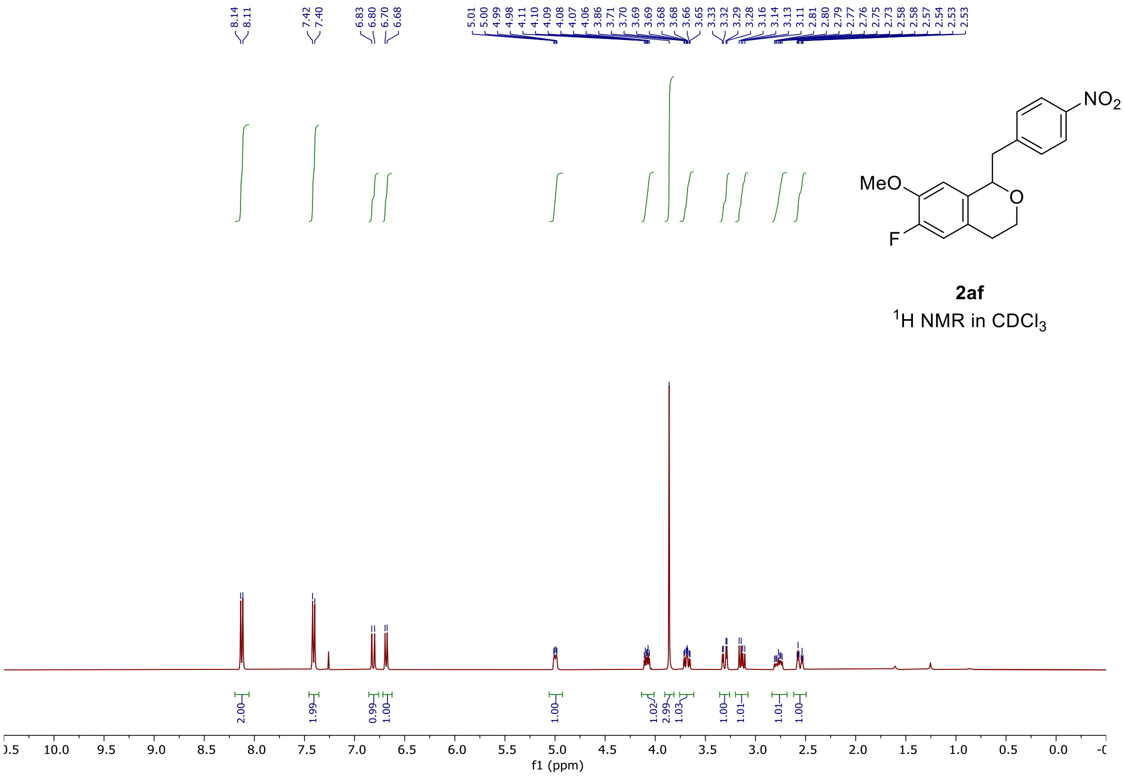

2af

^{1}H NMR in $CDCl_3$

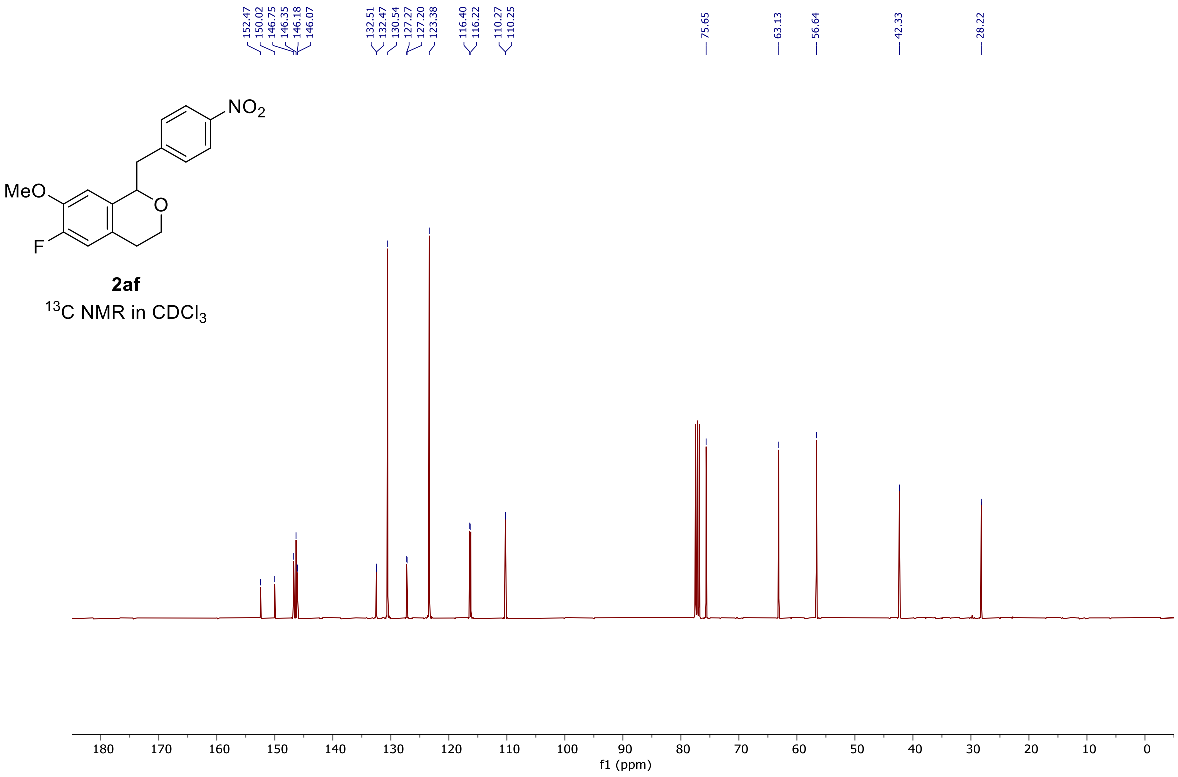

2af
^{13}C NMR in $CDCl_3$

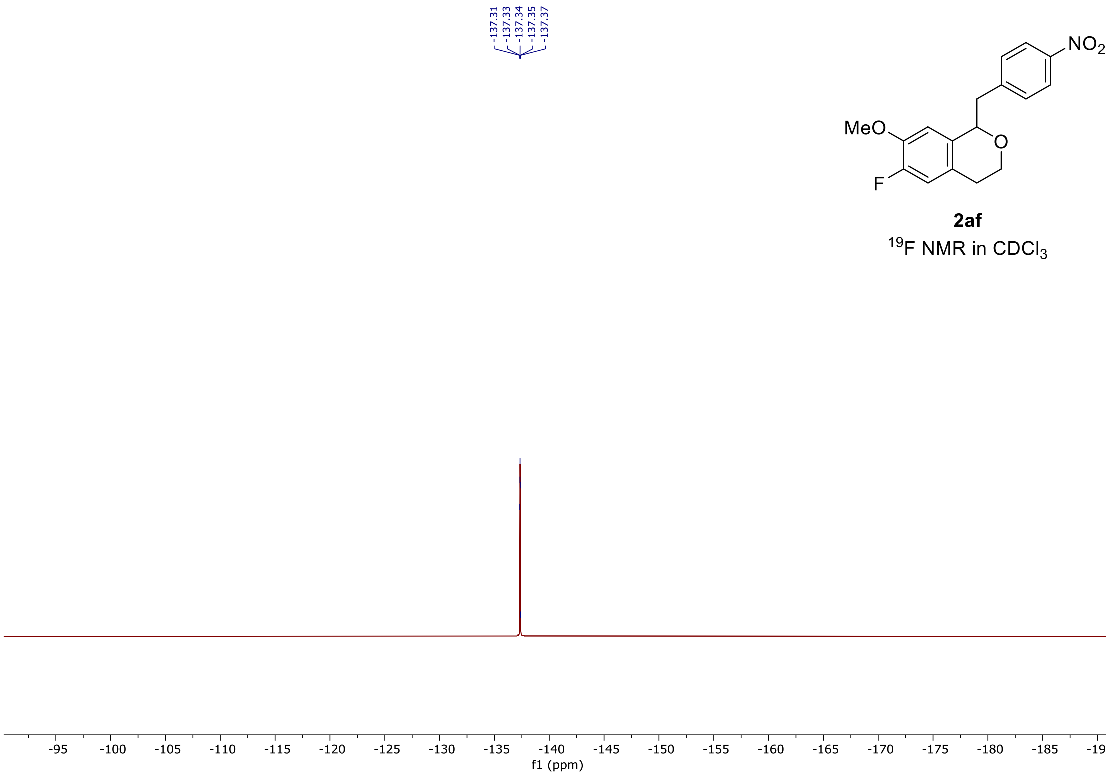

2af
^{19}F NMR in $CDCl_3$

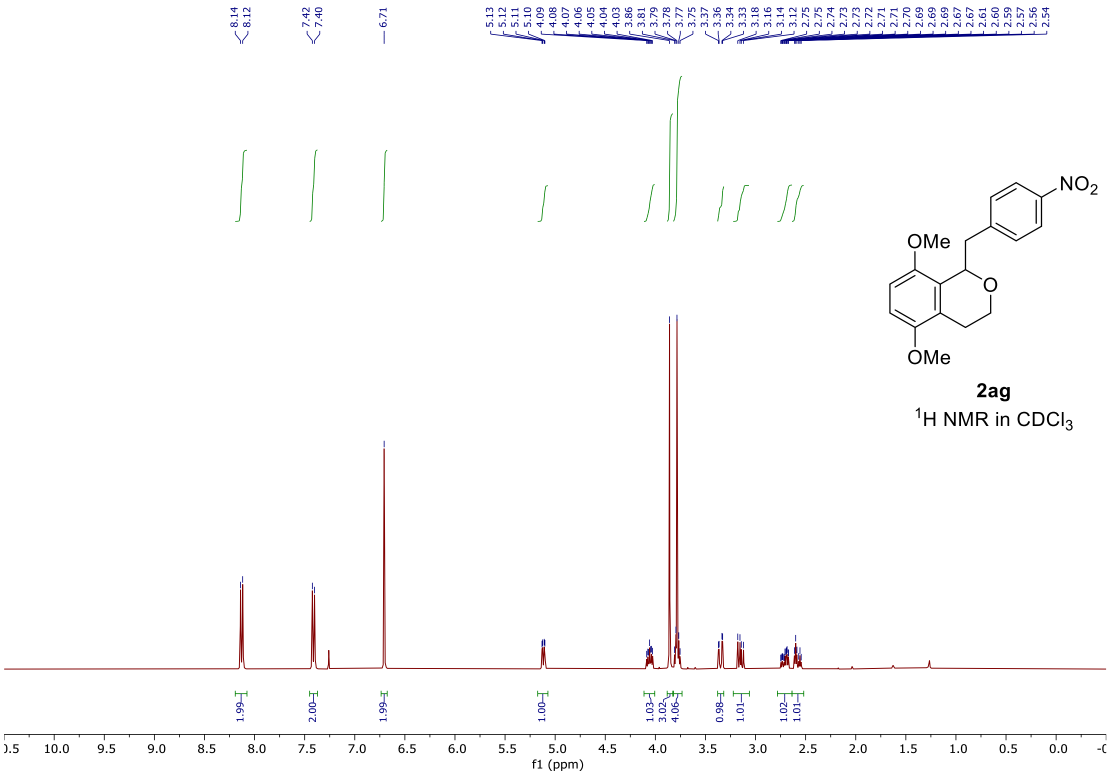

2ag
^{1}H NMR in $CDCl_3$

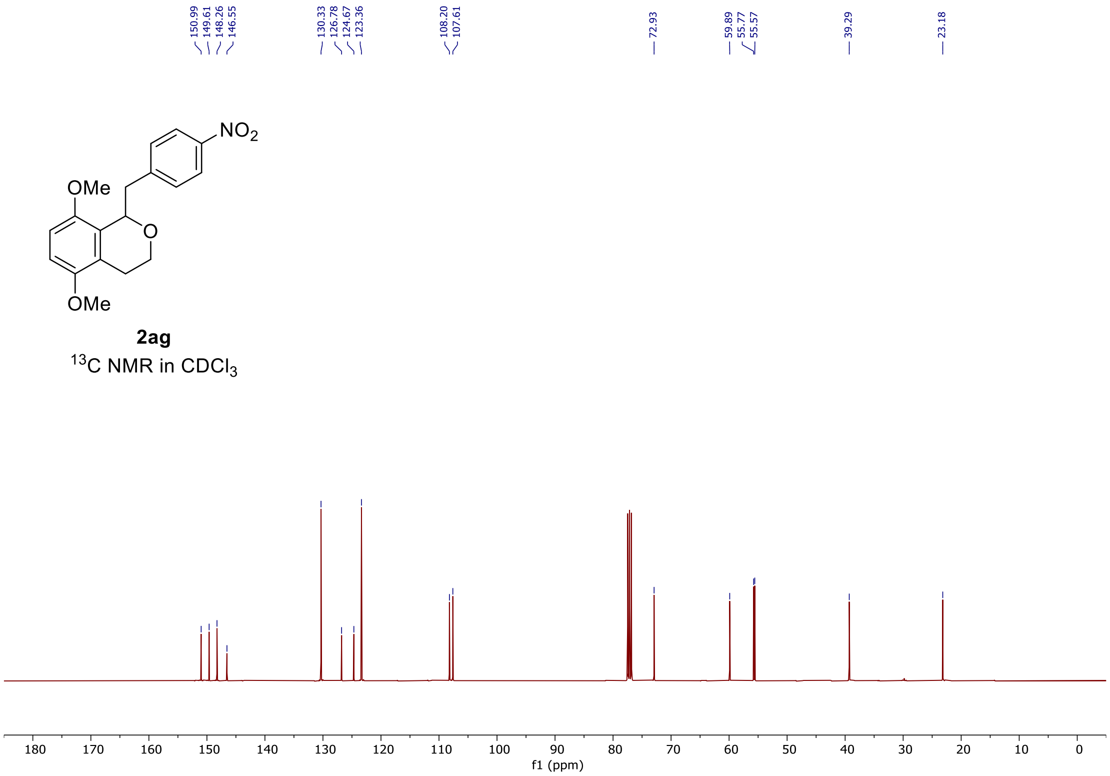

2ag
^{13}C NMR in $CDCl_3$

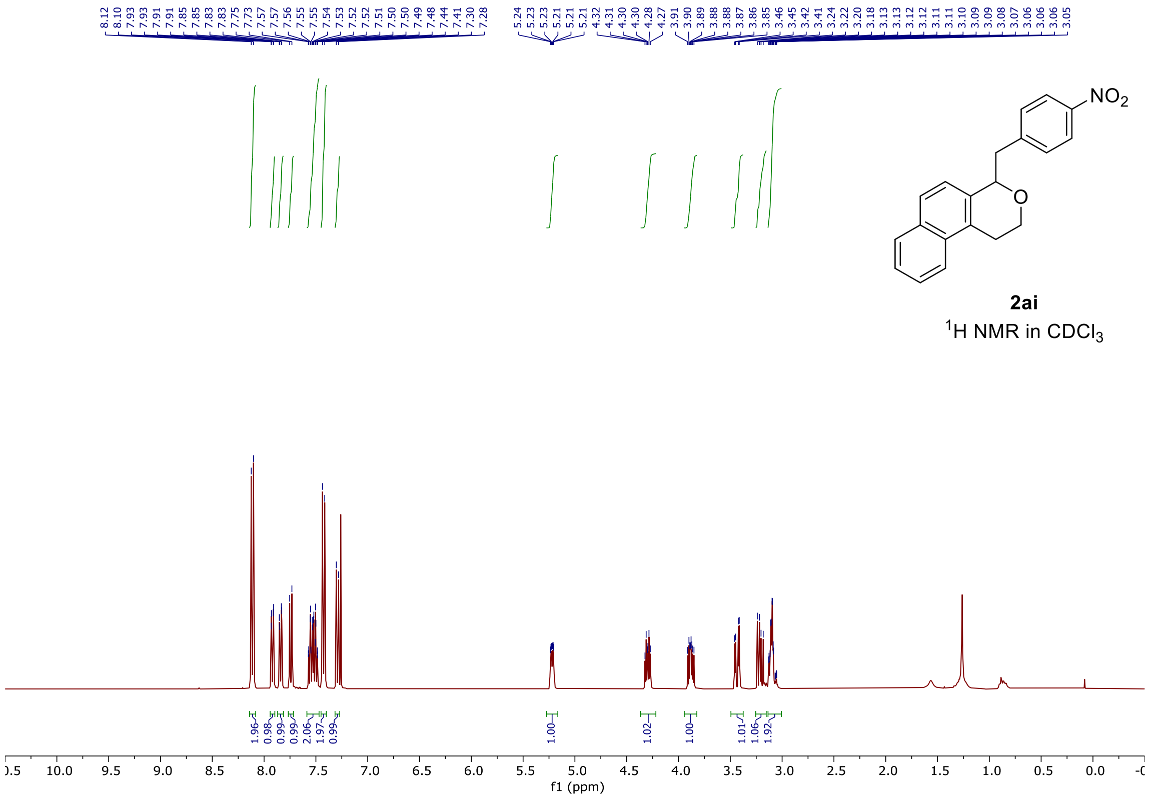

2ai

^{1}H NMR in $CDCl_3$

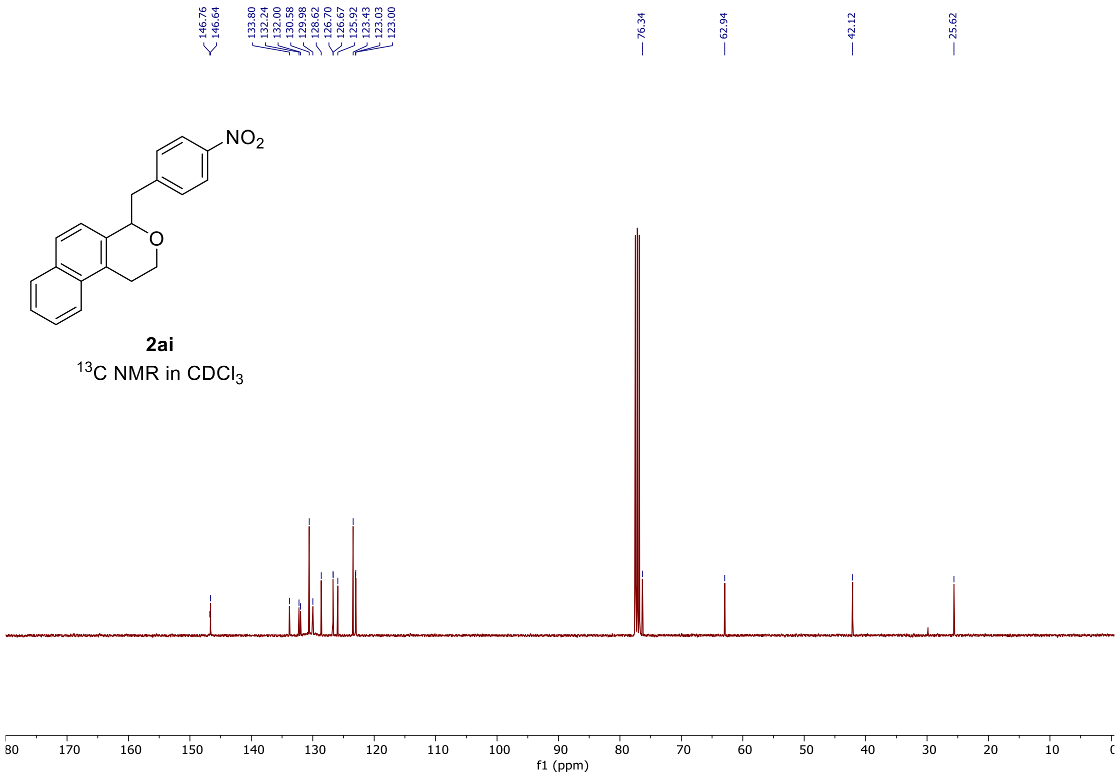

2ai

^{13}C NMR in $CDCl_3$

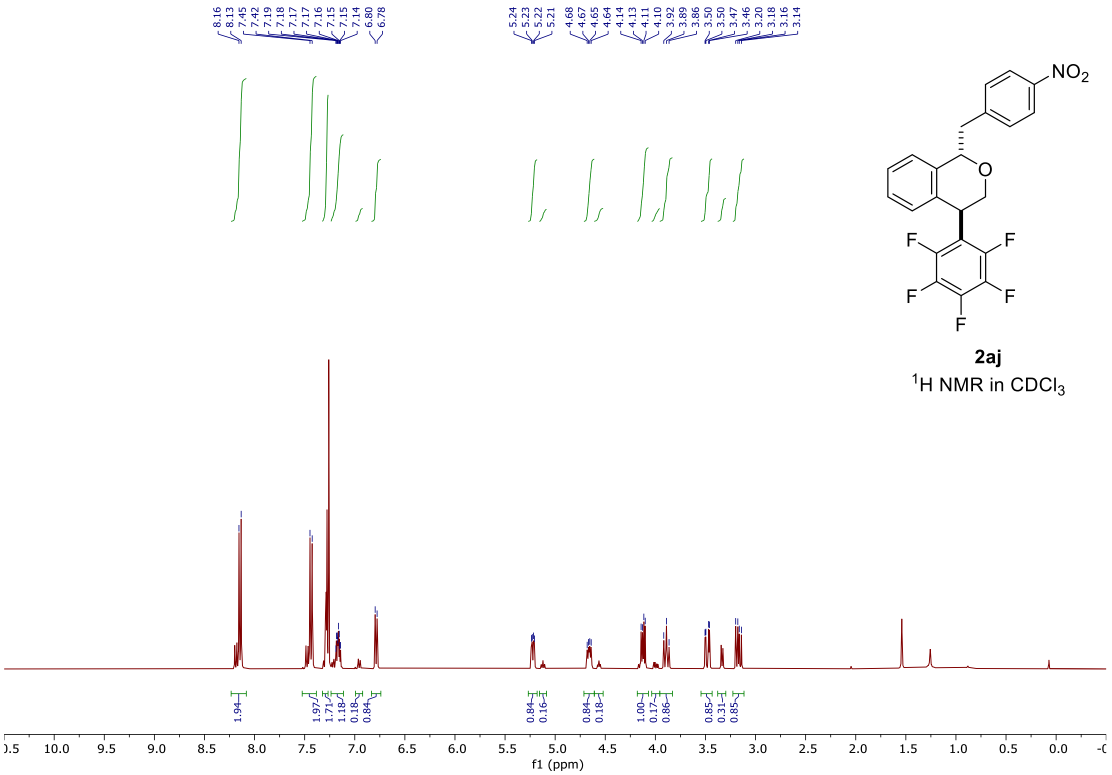

2aj

^{1}H NMR in $CDCl_3$

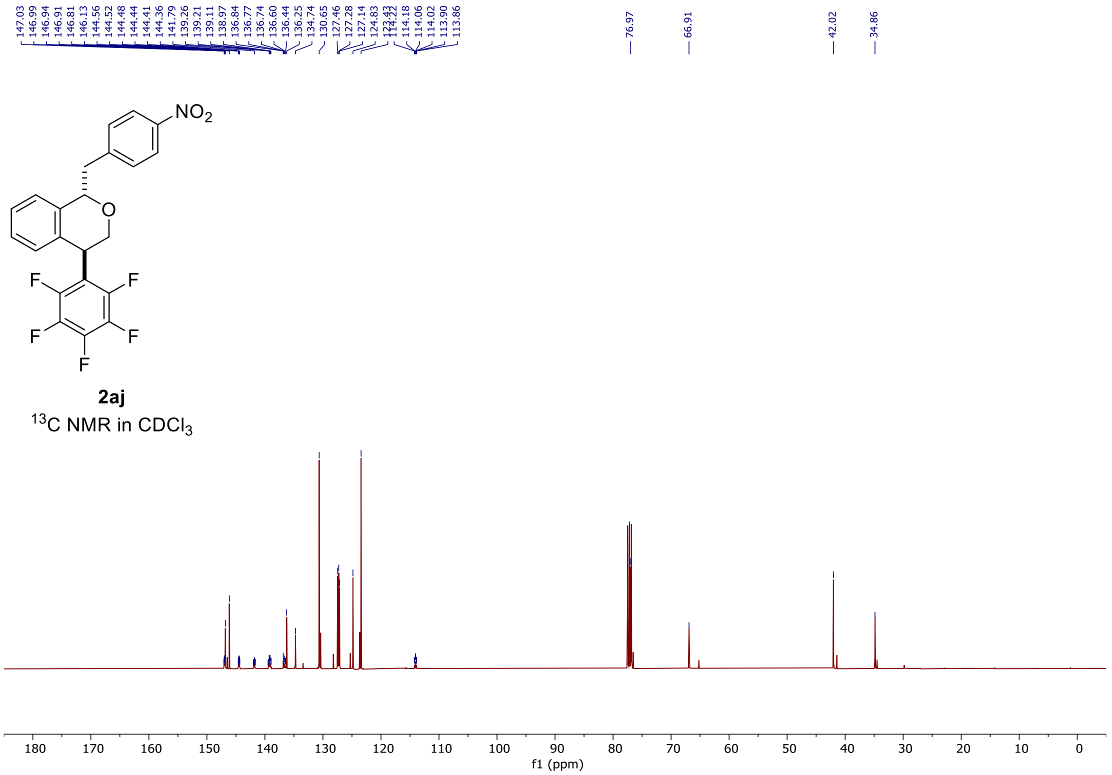

2aj

^{13}C NMR in $CDCl_3$

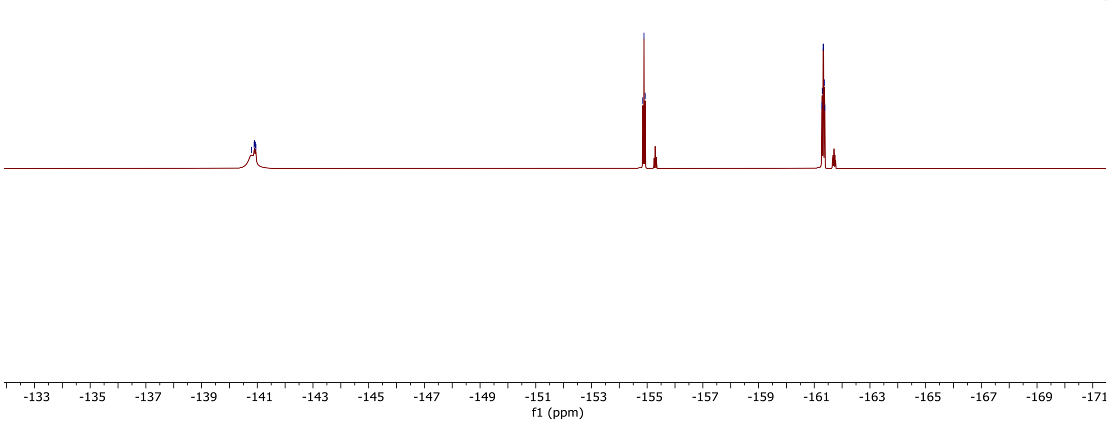

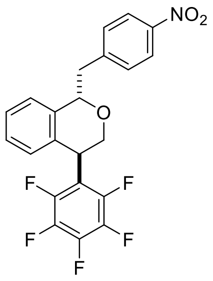

2aj

^{19}F NMR in $CDCl_3$

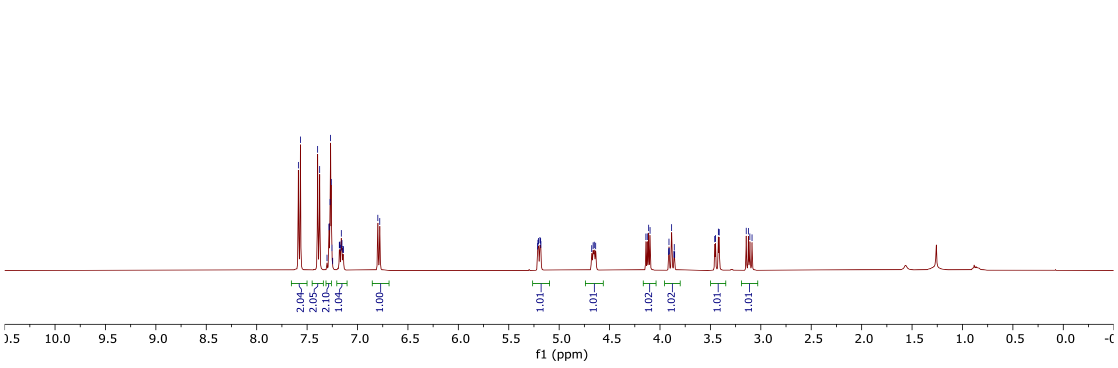

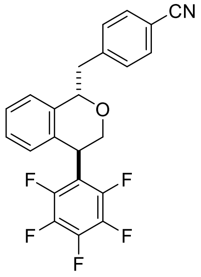

2ak

^{1}H NMR in $CDCl_3$

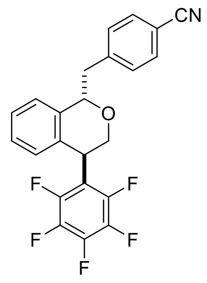

2ak

^{19}F NMR in $CDCl_3$

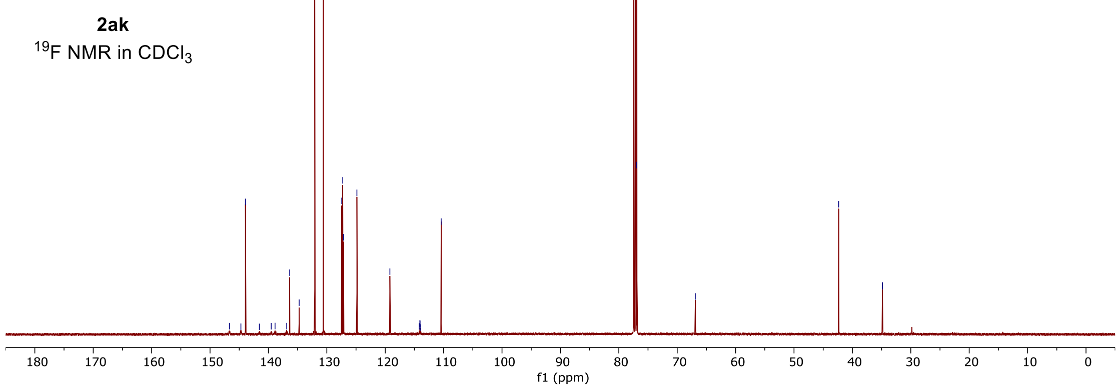

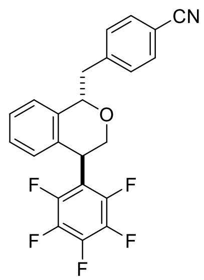

2ak

^{13}C NMR in $CDCl_3$

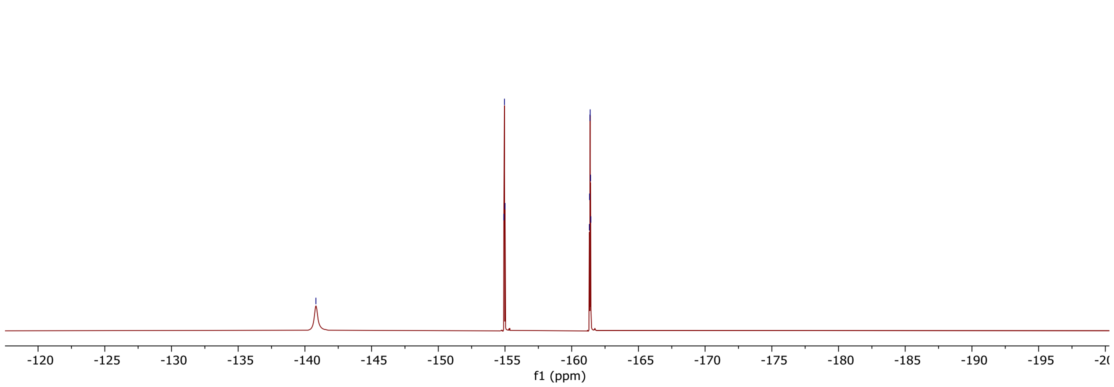

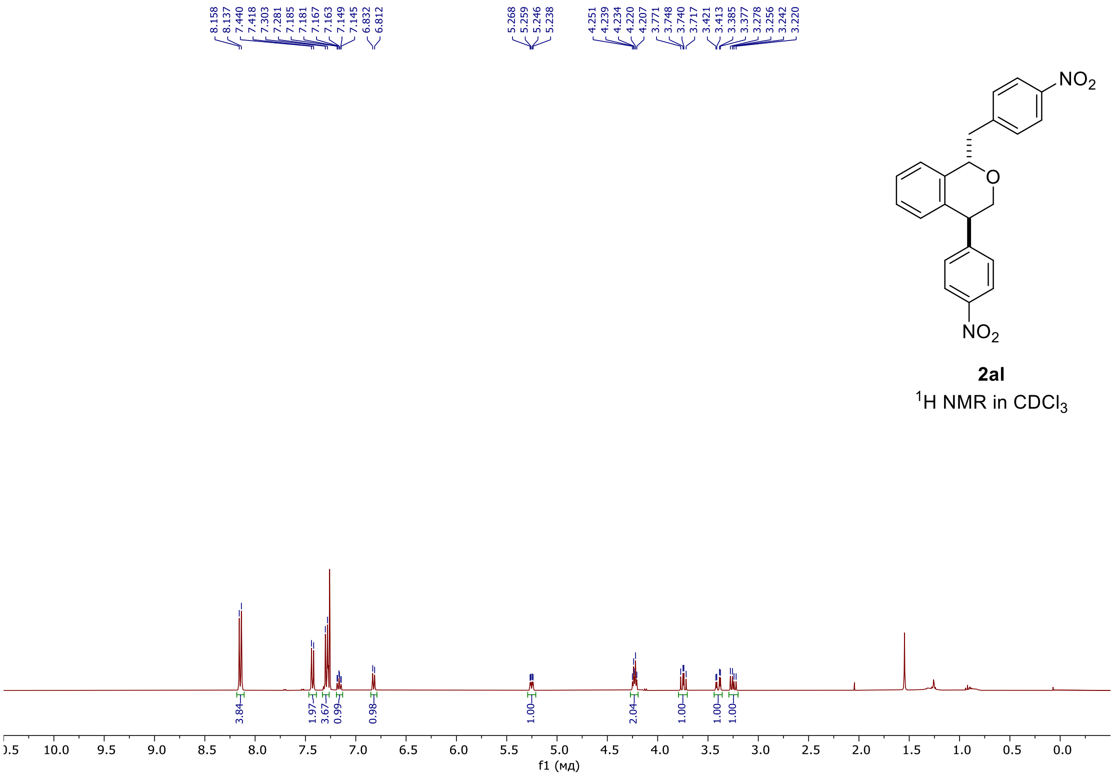

2al

^{1}H NMR in $CDCl_3$

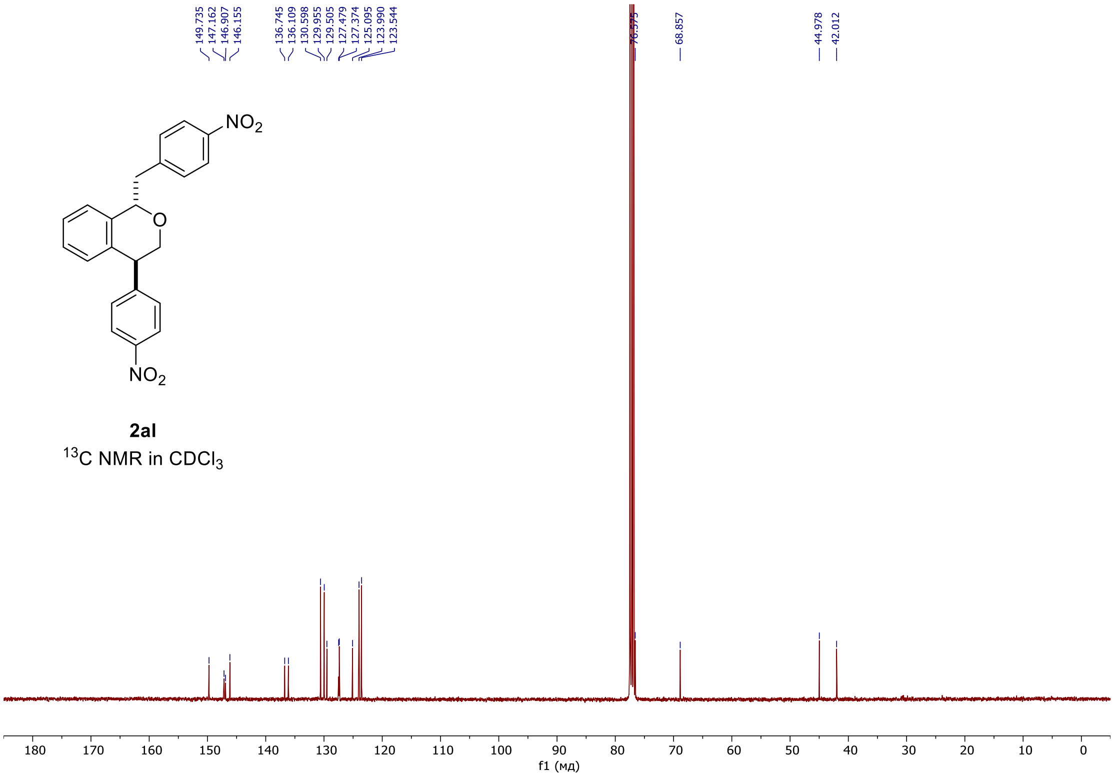

2al

^{13}C NMR in $CDCl_3$

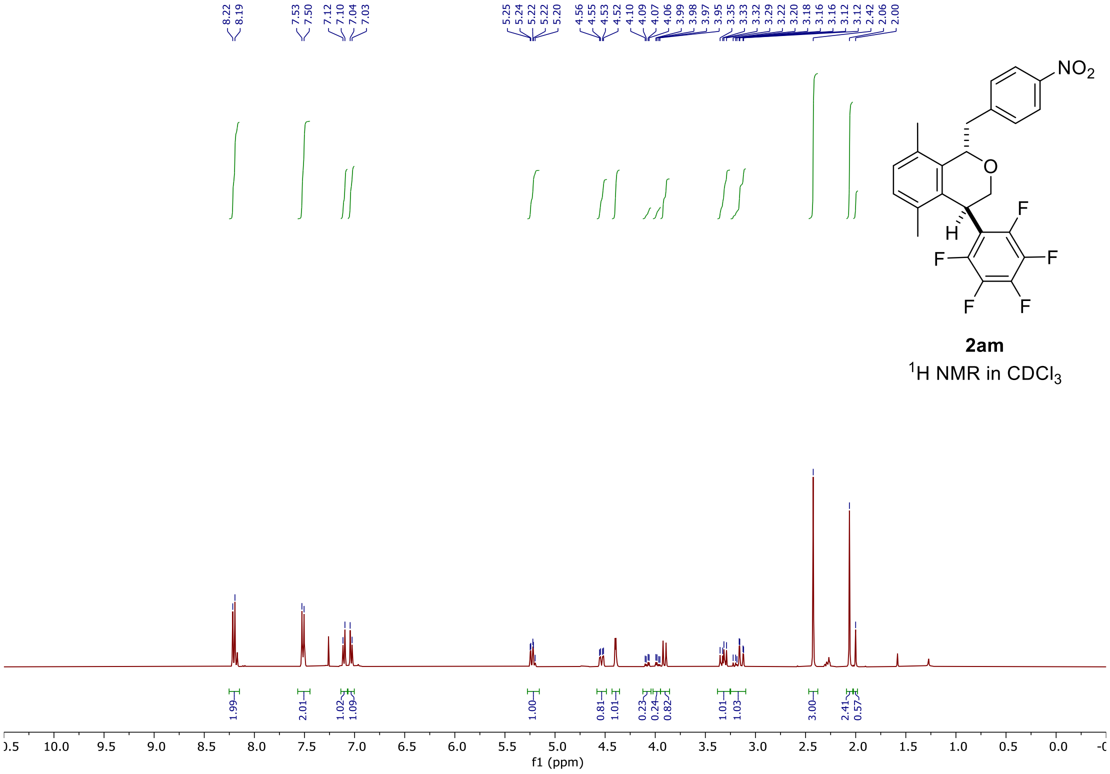

2am

^{1}H NMR in $CDCl_3$

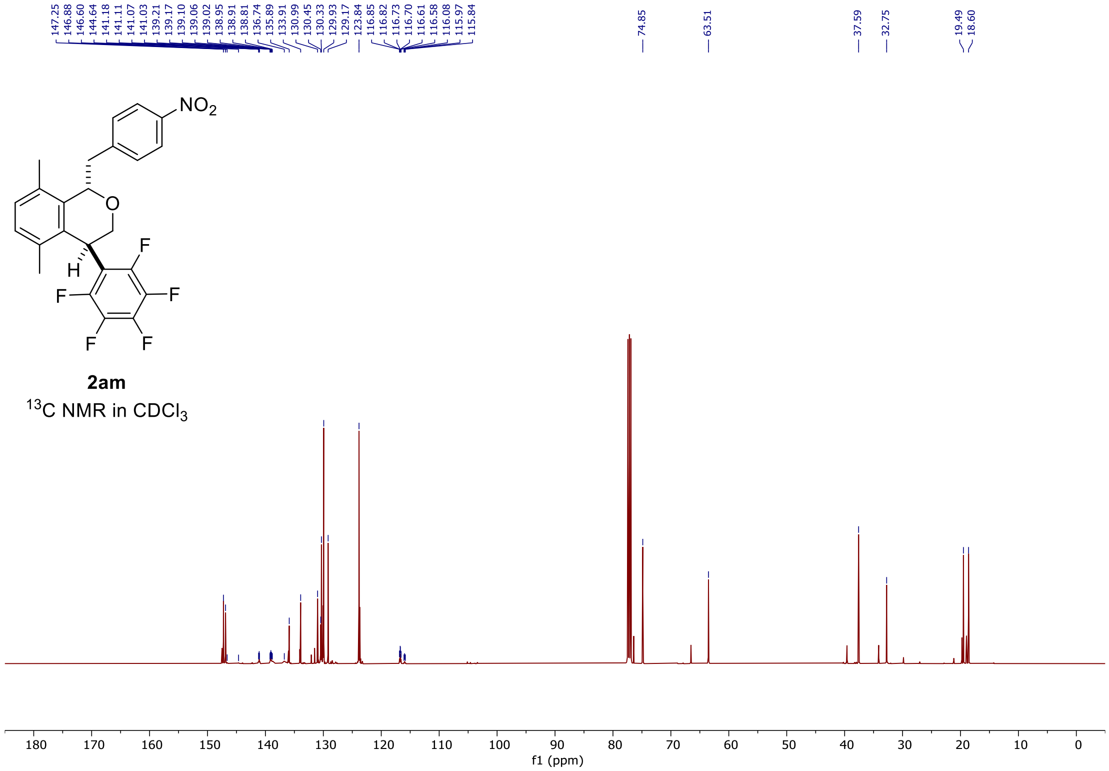

2am

^{13}C NMR in $CDCl_3$

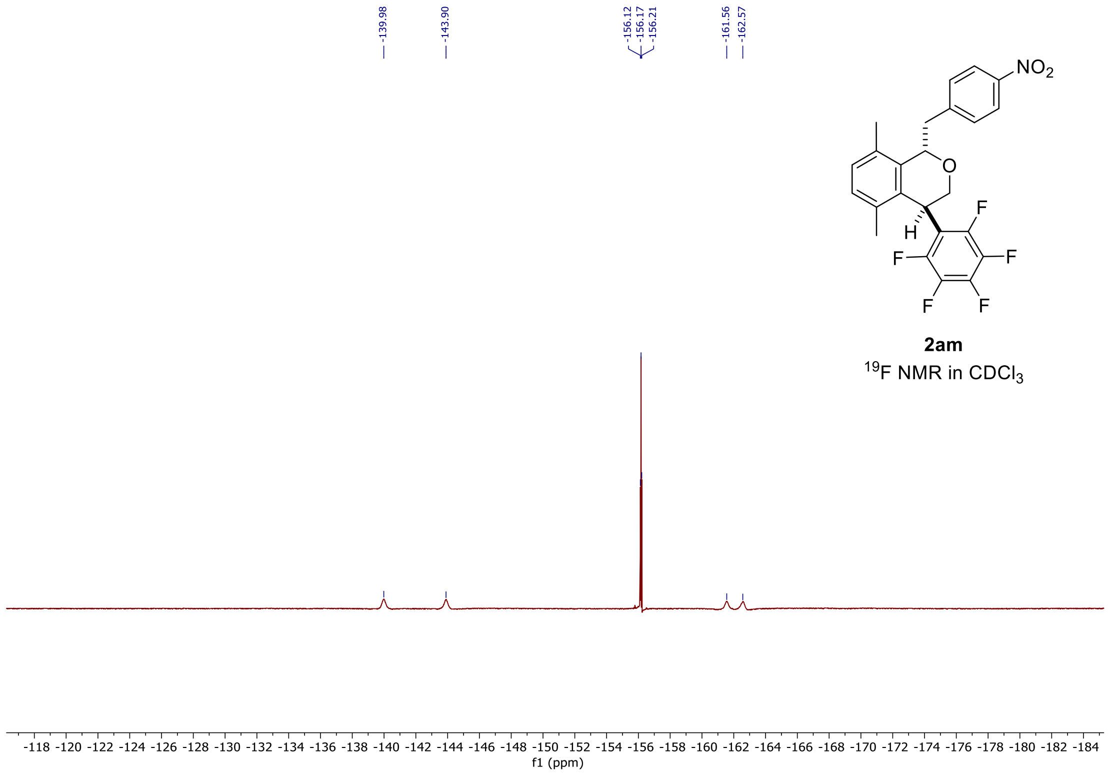

2am

^{19}F NMR in $CDCl_3$

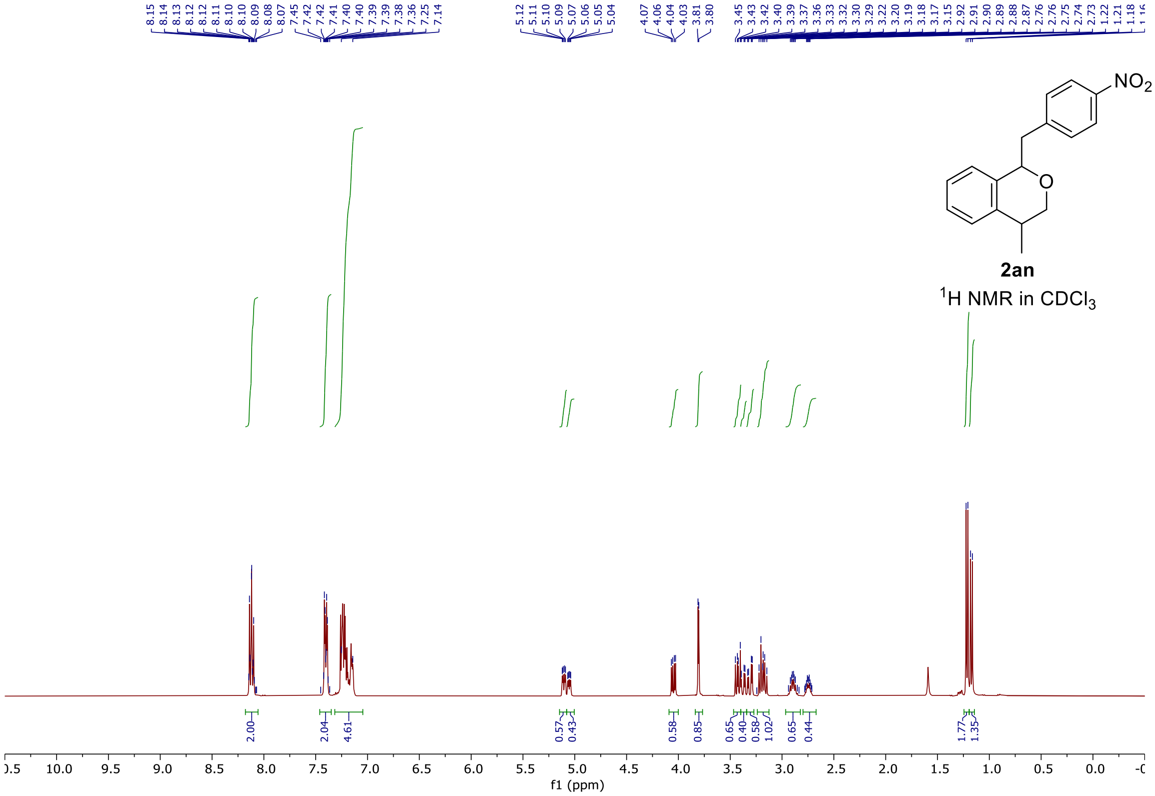

2an

^{1}H NMR in $CDCl_3$

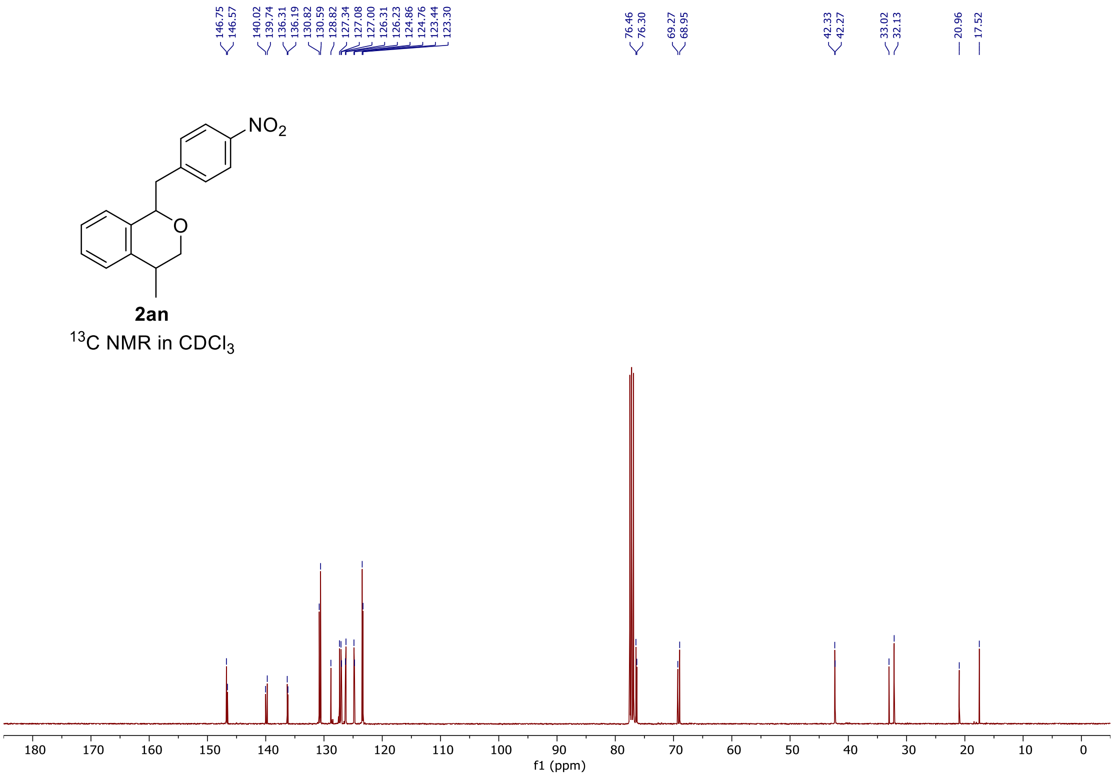
146.75
146.57
140.02
139.74
136.31
136.19
130.82
130.59
128.82
127.34
127.08
127.00
126.31
126.23
124.86
124.76
123.44
123.30
76.46
76.30
69.27
68.95
42.33
42.27
33.02
32.13
20.96
17.52
NO_2
O
2an
^{13}C NMR in $CDCl_3$
180 170 160 150 140 130 120 110 100 90 80 70 60 50 40 30 20 10 0
f1 (ppm)

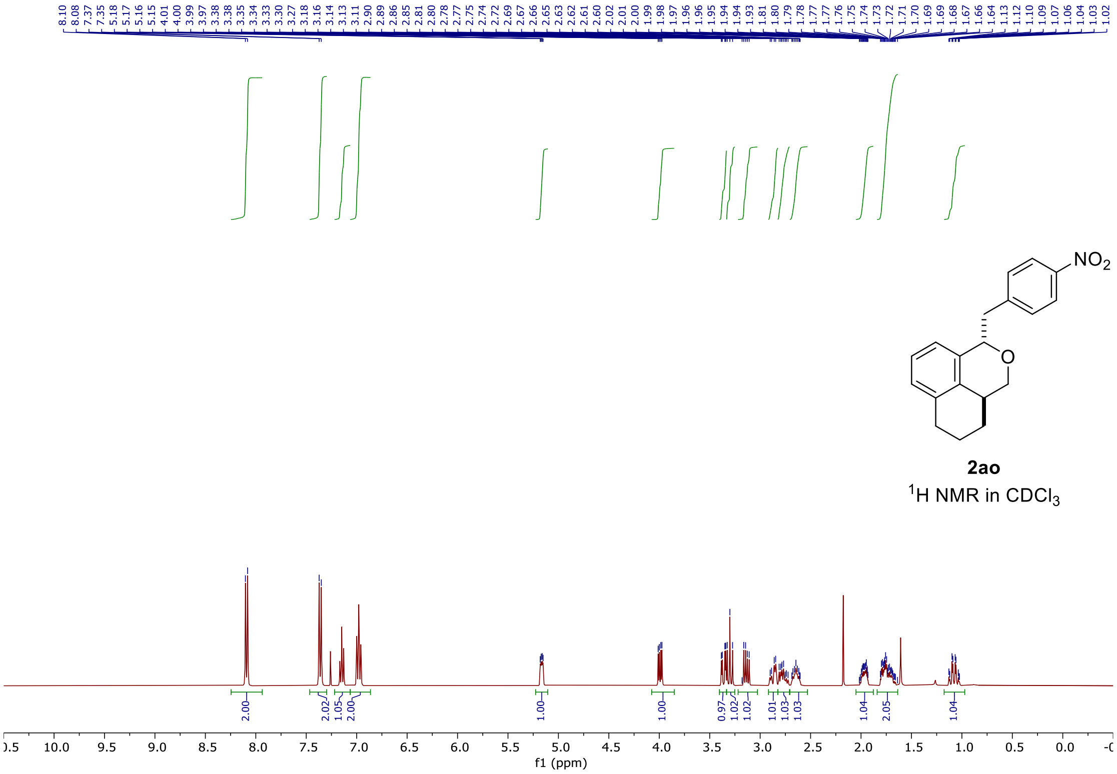
8.10
8.08
7.37
7.35
5.18
5.17
5.16
5.15
4.01
4.00
3.99
3.97
3.38
3.38
3.35
3.34
3.33
3.30
3.27
3.18
3.16
3.14
3.13
3.11
2.90
2.89
2.86
2.85
2.81
2.80
2.78
2.77
2.75
2.74
2.72
2.69
2.67
2.66
2.65
2.63
2.62
2.61
2.60
2.02
2.01
2.00
1.99
1.98
1.97
1.96
1.96
1.95
1.94
1.94
1.93
1.81
1.80
1.79
1.78
1.77
1.77
1.76
1.75
1.74
1.73
1.72
1.71
1.70
1.69
1.69
1.68
1.67
1.66
1.64
1.13
1.12
1.10
1.09
1.07
1.06
1.04
1.03
1.02
NO_2
O
2ao
^1H NMR in $CDCl_3$
2.00
2.02
1.05
2.00
1.00
1.00
0.97
1.02
1.02
1.01
1.03
1.03
1.04
2.05
1.04
10.0 9.5 9.0 8.5 8.0 7.5 7.0 6.5 6.0 5.5 5.0 4.5 4.0 3.5 3.0 2.5 2.0 1.5 1.0 0.5 0.0
f1 (ppm)

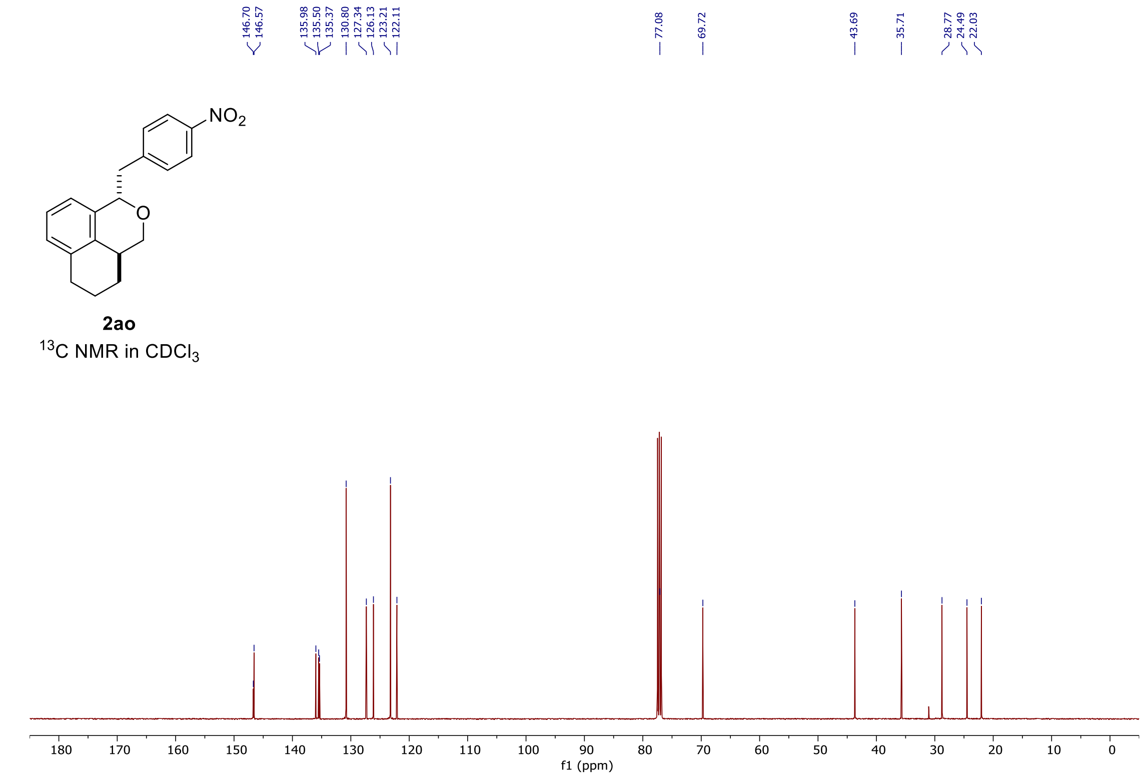
146.70
146.57
135.98
135.50
135.37
130.80
127.34
126.13
123.21
122.11
77.08
69.72
43.69
35.71
28.77
24.49
22.03
NO_2
2ao
^{13}C NMR in $CDCl_3$
180 170 160 150 140 130 120 110 100 90 80 70 60 50 40 30 20 10 0
f1 (ppm)

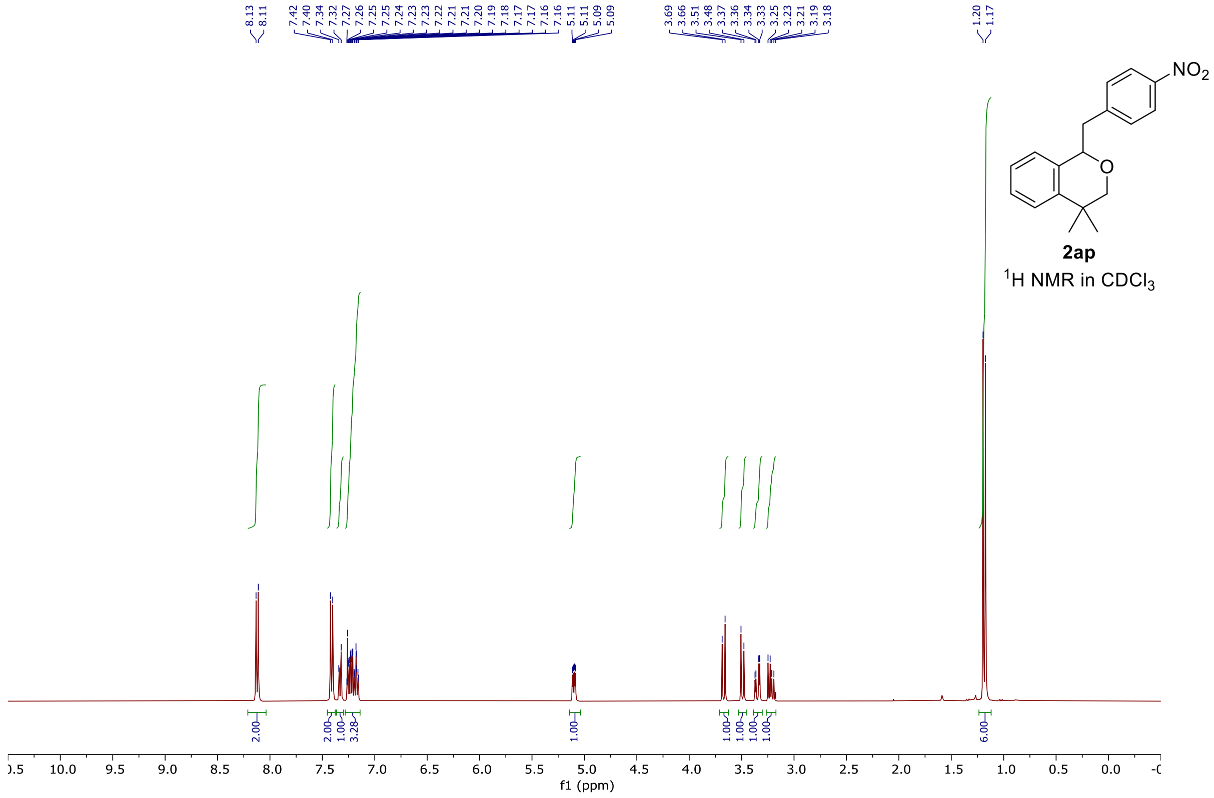
8.13
8.11
7.42
7.40
7.34
7.32
7.27
7.26
7.25
7.25
7.24
7.23
7.23
7.22
7.22
7.21
7.21
7.20
7.19
7.18
7.17
7.17
7.16
5.11
5.11
5.09
5.09
3.69
3.66
3.51
3.48
3.37
3.36
3.34
3.33
3.25
3.23
3.21
3.19
3.18
1.20
1.17
NO_2
2ap
^{1}H NMR in $CDCl_3$
2.00
2.00
1.00
3.28
1.00
1.00
1.00
1.00
1.00
6.00
10.0 9.5 9.0 8.5 8.0 7.5 7.0 6.5 6.0 5.5 5.0 4.5 4.0 3.5 3.0 2.5 2.0 1.5 1.0 0.5 0.0
f1 (ppm)

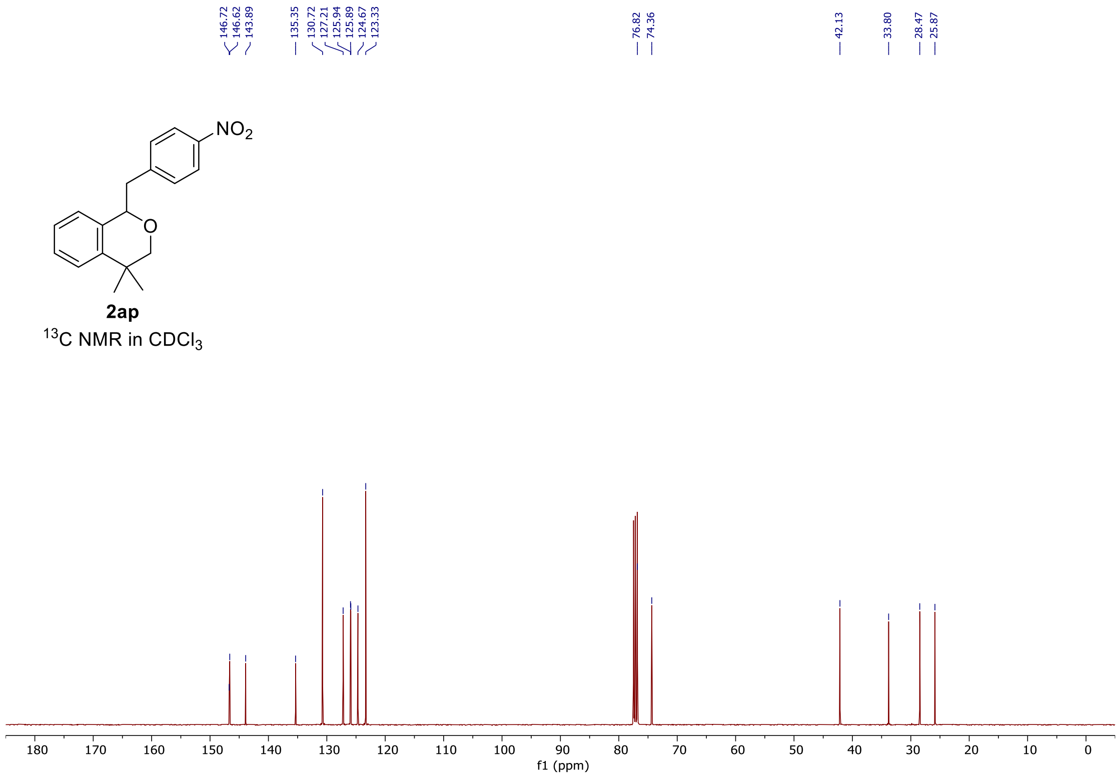
146.72
146.62
143.89
135.35
130.72
127.21
125.94
125.89
124.67
123.33
76.82
74.36
42.13
33.80
28.47
25.87
NO2
O
2ap
13C NMR in CDCl3
f1 (ppm)

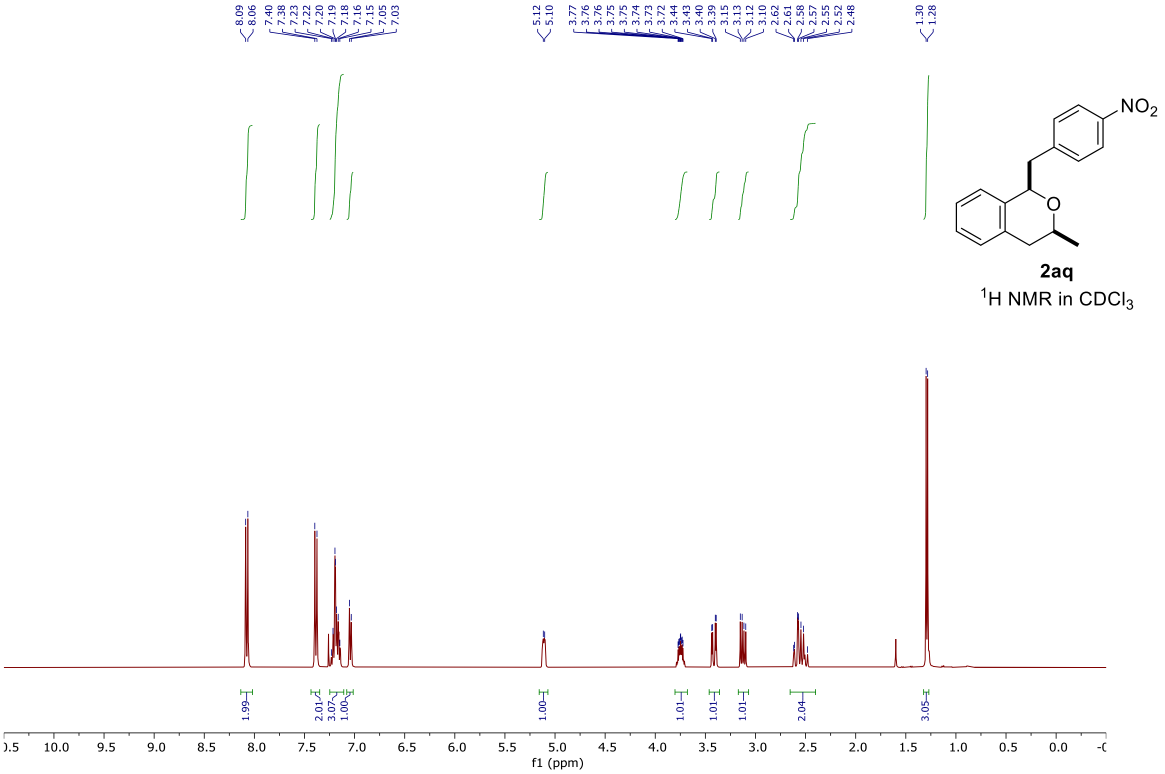
8.09
8.06
7.40
7.38
7.23
7.22
7.20
7.19
7.18
7.16
7.15
7.05
7.03
5.12
5.10
3.77
3.76
3.75
3.75
3.74
3.73
3.72
3.44
3.43
3.40
3.39
3.15
3.13
3.12
3.10
2.62
2.61
2.58
2.57
2.55
2.52
2.48
1.30
1.28
NO2
O
2aq
1H NMR in CDCl3
1.99
2.01
3.07
1.00
1.00
1.01
1.01
1.01
2.04
3.05
f1 (ppm)

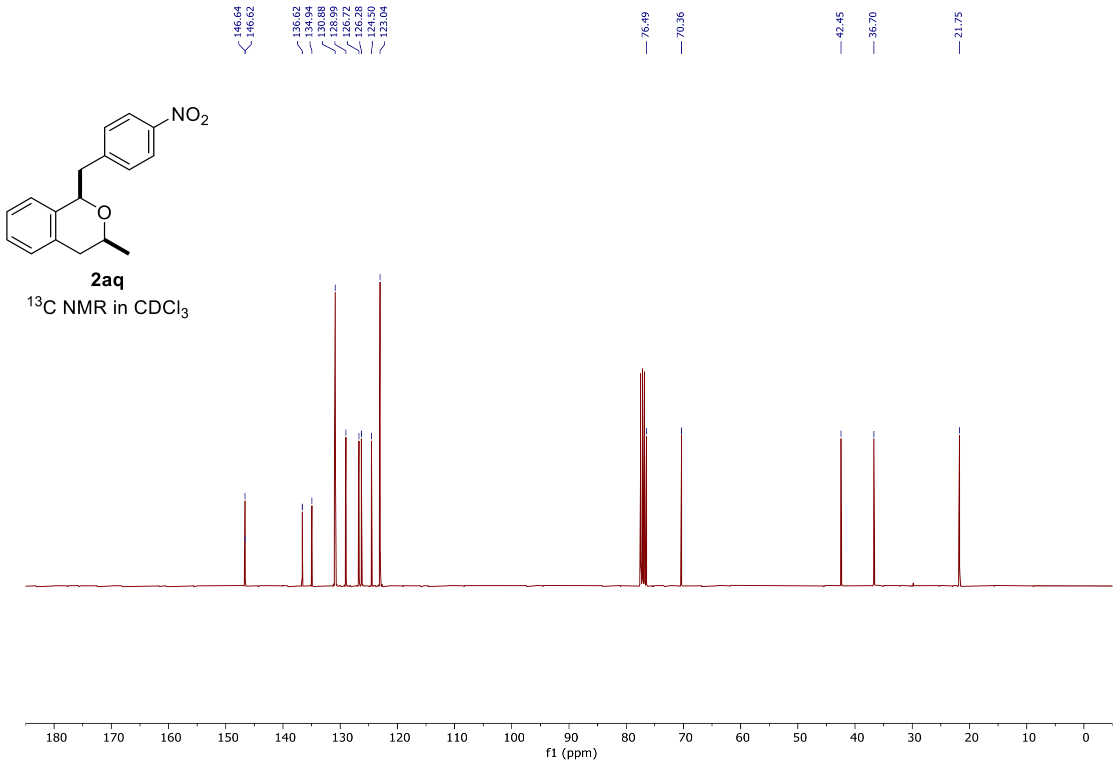
146.64
146.62
136.62
134.94
130.88
128.99
126.72
126.28
124.50
123.04
76.49
70.36
42.45
36.70
21.75
NO2
O
2aq
13C NMR in CDCl3
f1 (ppm)

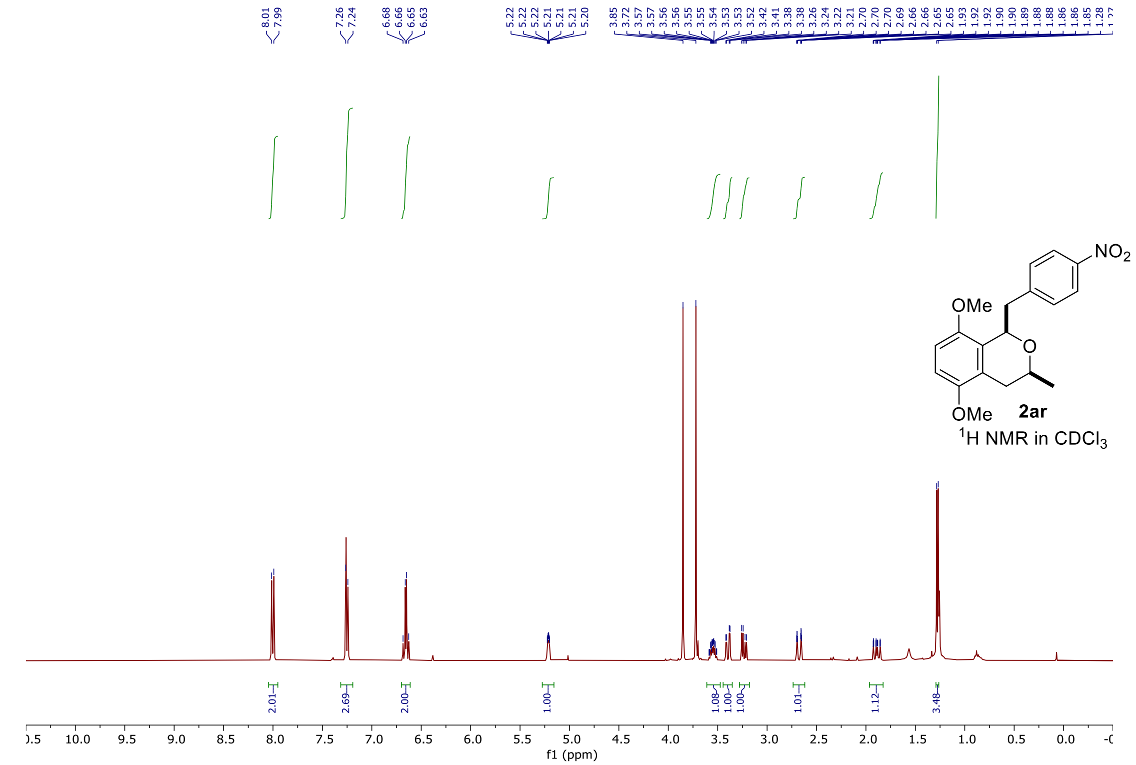
OMe
NO2
O
OMe
2ar
1H NMR in CDCl3
2.01
2.69
2.00
1.00
1.08
1.00
1.00
1.01
1.12
3.48
f1 (ppm)

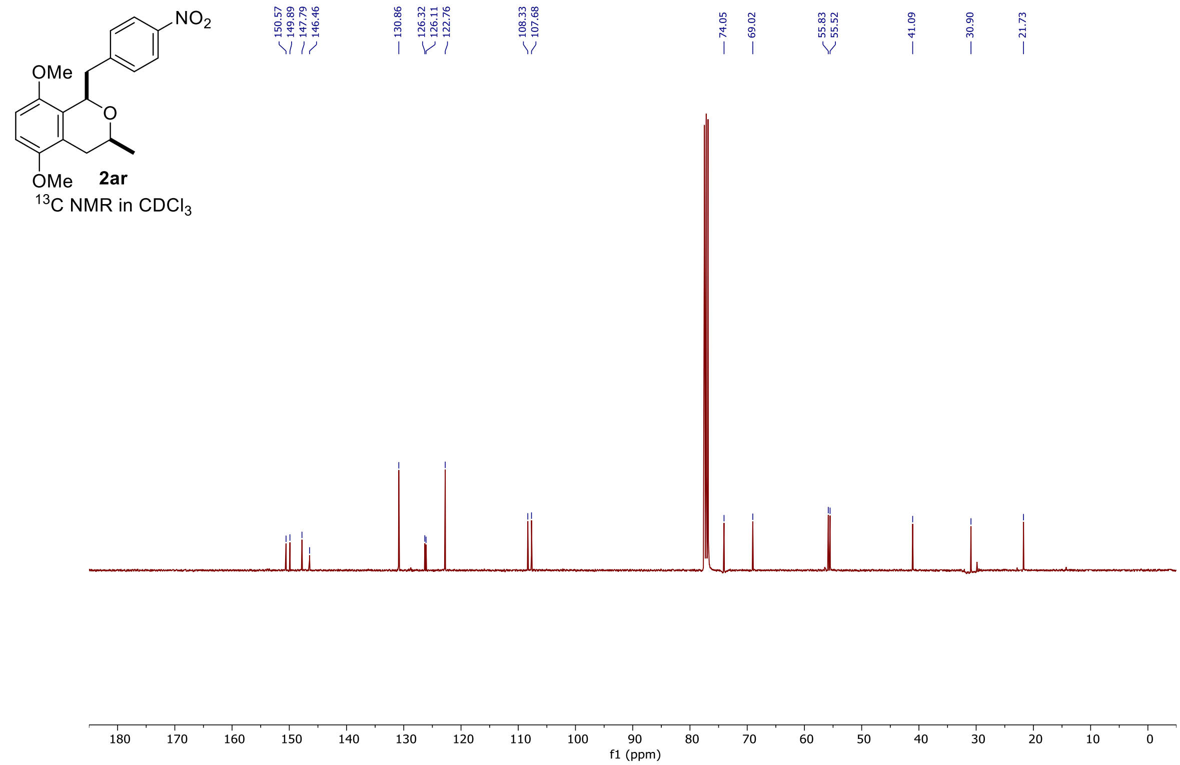
NO2
OMe
O
OMe
2ar
13C NMR in CDCl3
150.57
149.89
147.79
146.46
130.86
126.32
126.11
122.76
108.33
107.68
74.05
69.02
55.83
55.52
41.09
30.90
21.73
180 170 160 150 140 130 120 110 100 90 80 70 60 50 40 30 20 10 0
f1 (ppm)

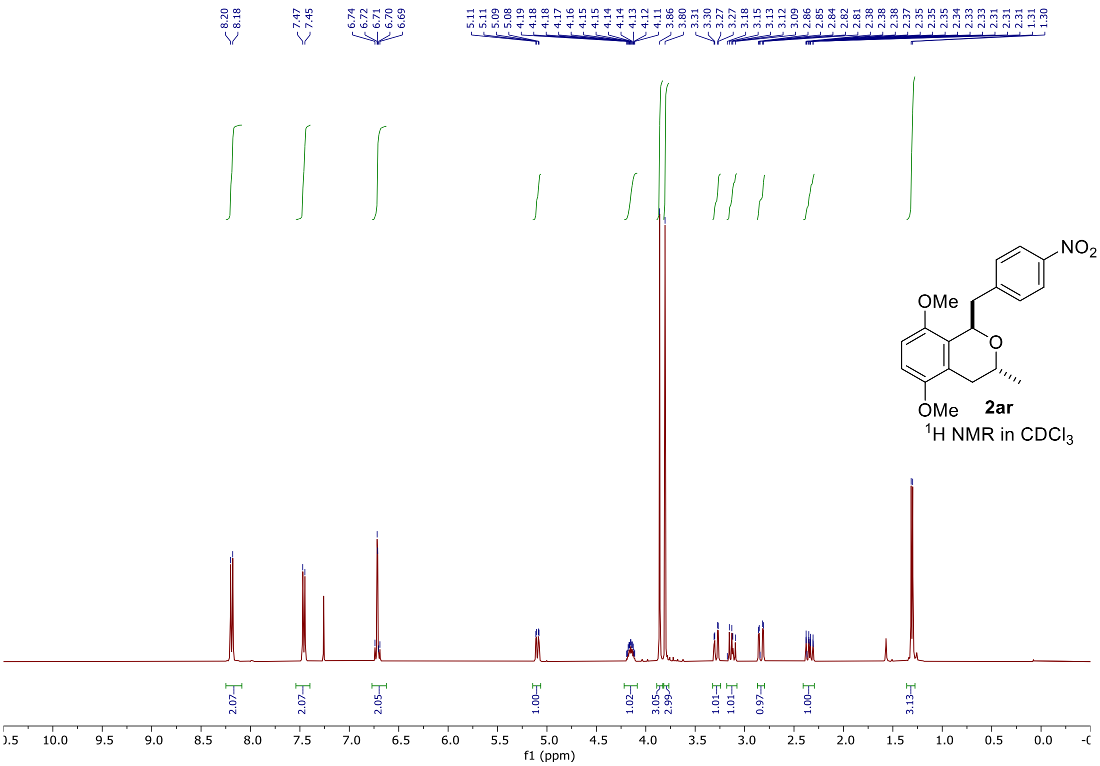
8.20
8.18
7.47
7.45
6.74
6.72
6.71
6.70
6.69
5.11
5.11
5.09
5.08
4.19
4.18
4.18
4.17
4.16
4.15
4.15
4.14
4.13
4.12
4.11
3.86
3.80
3.31
3.30
3.27
3.27
3.18
3.15
3.13
3.12
3.09
2.86
2.85
2.84
2.82
2.81
2.38
2.38
2.38
2.37
2.35
2.35
2.34
2.33
2.33
2.31
2.31
1.31
1.30
NO2
OMe
O
OMe
2ar
1H NMR in CDCl3
2.07
2.07
2.05
1.00
1.02
3.05
2.99
1.01
1.01
0.97
1.00
3.13
10.0 9.5 9.0 8.5 8.0 7.5 7.0 6.5 6.0 5.5 5.0 4.5 4.0 3.5 3.0 2.5 2.0 1.5 1.0 0.5 0.0
f1 (ppm)

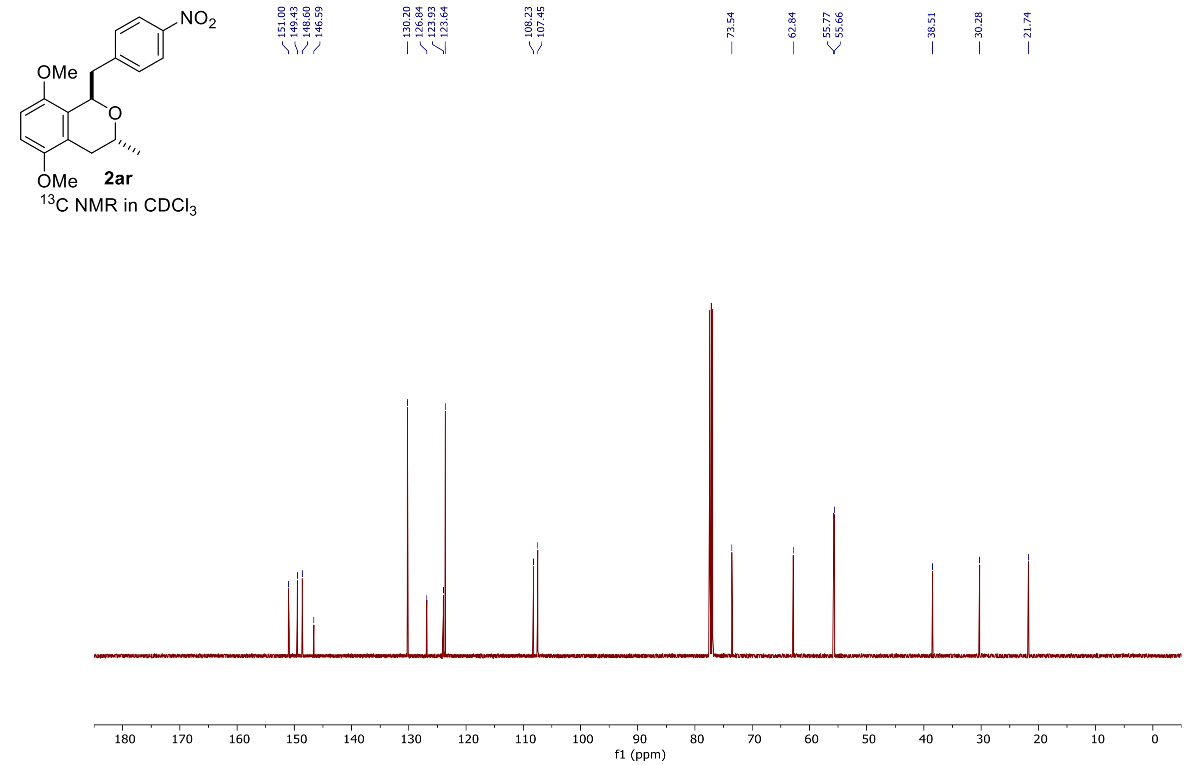
NO2
OMe
O
OMe
2ar
13C NMR in CDCl3
151.00
149.43
148.60
146.59
130.20
126.84
123.93
123.64
108.23
107.45
73.54
62.84
55.77
55.66
38.51
30.28
21.74
180 170 160 150 140 130 120 110 100 90 80 70 60 50 40 30 20 10 0
f1 (ppm)

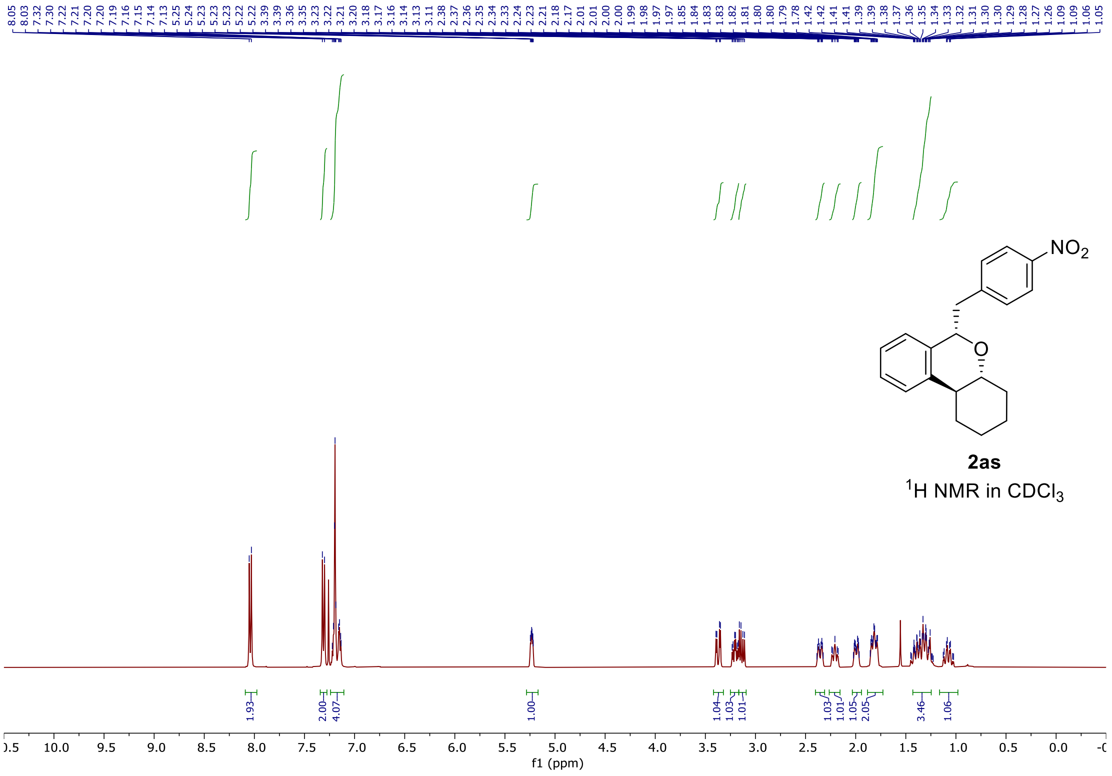
8.05 8.03 7.32 7.30 7.22 7.21 7.20 7.19 7.16 7.15 7.14 7.13 5.25 5.24 5.23 5.23 5.23 5.22 5.22 3.39 3.39 3.36 3.35 3.23 3.22 3.21 3.20 3.18 3.17 3.16 3.14 3.13 3.11 2.38 2.37 2.36 2.35 2.34 2.33 2.24 2.23 2.21 2.18 2.17 2.01 2.00 2.00 1.99 1.98 1.97 1.97 1.85 1.84 1.83 1.82 1.81 1.80 1.79 1.78 1.42 1.41 1.41 1.39 1.39 1.38 1.37 1.36 1.35 1.34 1.33 1.32 1.31 1.30 1.29 1.28 1.27 1.26 1.09 1.06 1.05
NO2
O
2as
1H NMR in CDCl3
1.93
2.00
4.07
1.00
1.04
1.03
1.01
1.03
1.01
1.05
2.05
3.46
1.06
10.0 9.5 9.0 8.5 8.0 7.5 7.0 6.5 6.0 5.5 5.0 4.5 4.0 3.5 3.0 2.5 2.0 1.5 1.0 0.5 0.0
f1 (ppm)

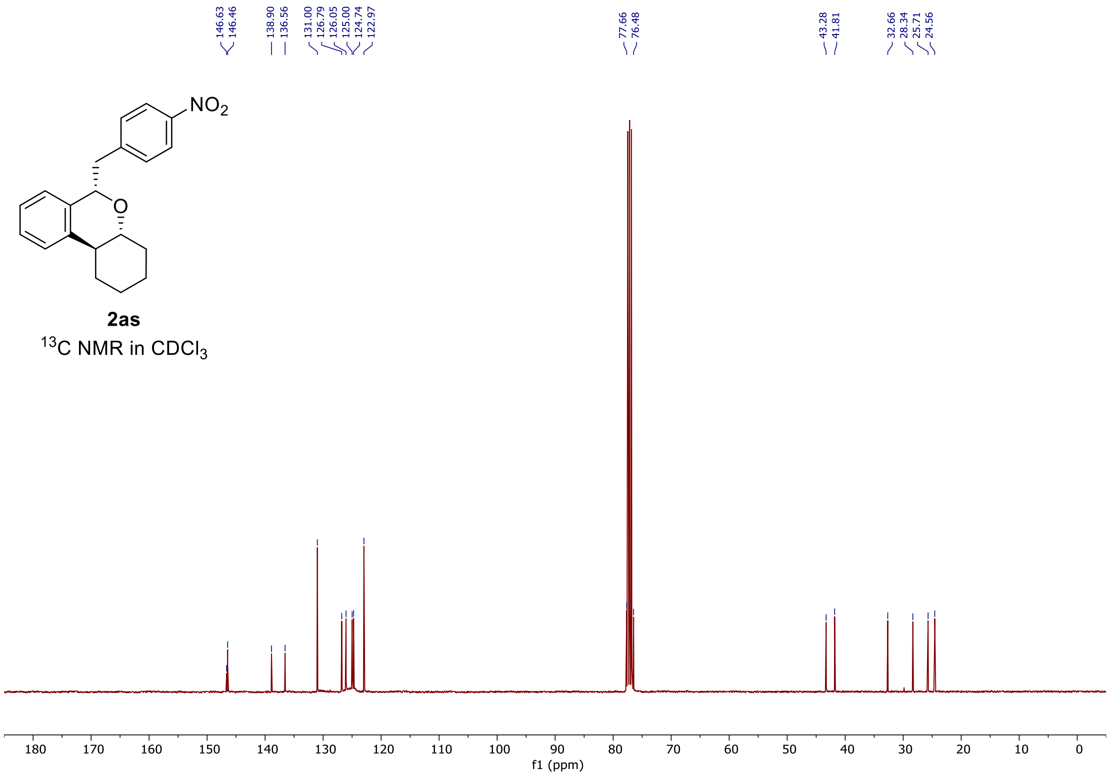
146.63
146.46
138.90
136.56
131.00
126.79
126.05
125.00
124.74
122.97
77.66
76.48
43.28
41.81
32.66
28.34
25.71
24.56
NO2
O
2as
13C NMR in CDCl3
f1 (ppm)

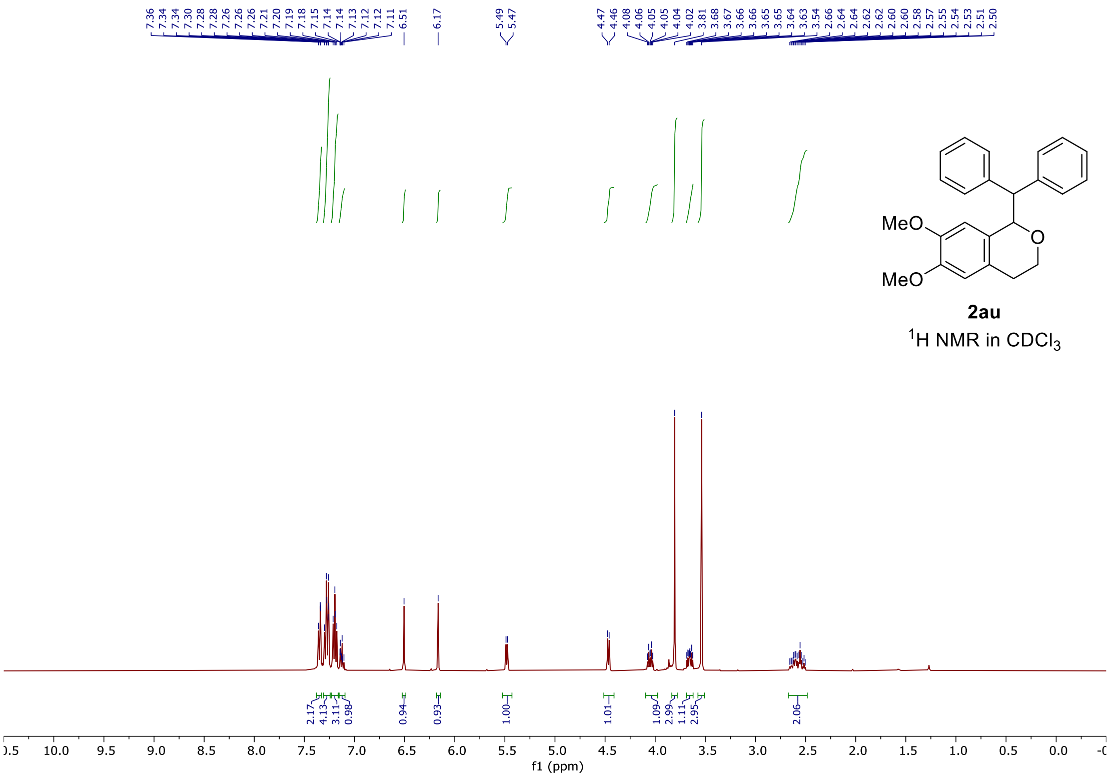
7.36
7.34
7.34
7.30
7.28
7.28
7.26
7.26
7.26
7.21
7.20
7.19
7.18
7.15
7.14
7.14
7.13
7.12
7.11
6.51
6.17
5.49
5.47
4.47
4.46
4.08
4.06
4.05
4.05
4.04
4.02
3.81
3.68
3.67
3.66
3.66
3.65
3.65
3.64
3.63
3.54
2.66
2.64
2.64
2.62
2.62
2.60
2.60
2.58
2.57
2.55
2.54
2.53
2.51
2.50
MeO
MeO
O
2au
1H NMR in CDCl3
2.17
4.13
3.11
0.98
0.94
0.93
1.00
1.01
1.09
2.99
1.11
2.95
2.06
f1 (ppm)

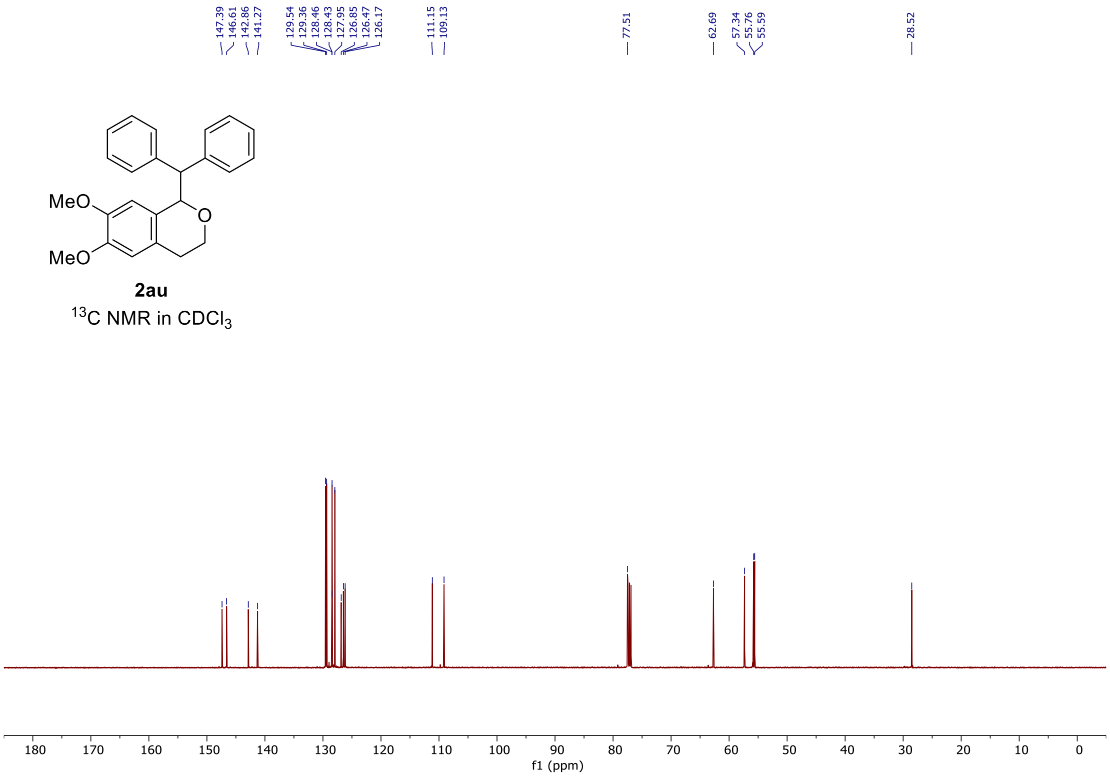
147.39
146.61
142.86
141.27
129.54
129.36
128.46
128.43
127.95
126.85
126.47
126.17
111.15
109.13
77.51
62.69
57.34
55.76
55.59
28.52
MeO
MeO
O
2au
13C NMR in CDCl3
f1 (ppm)

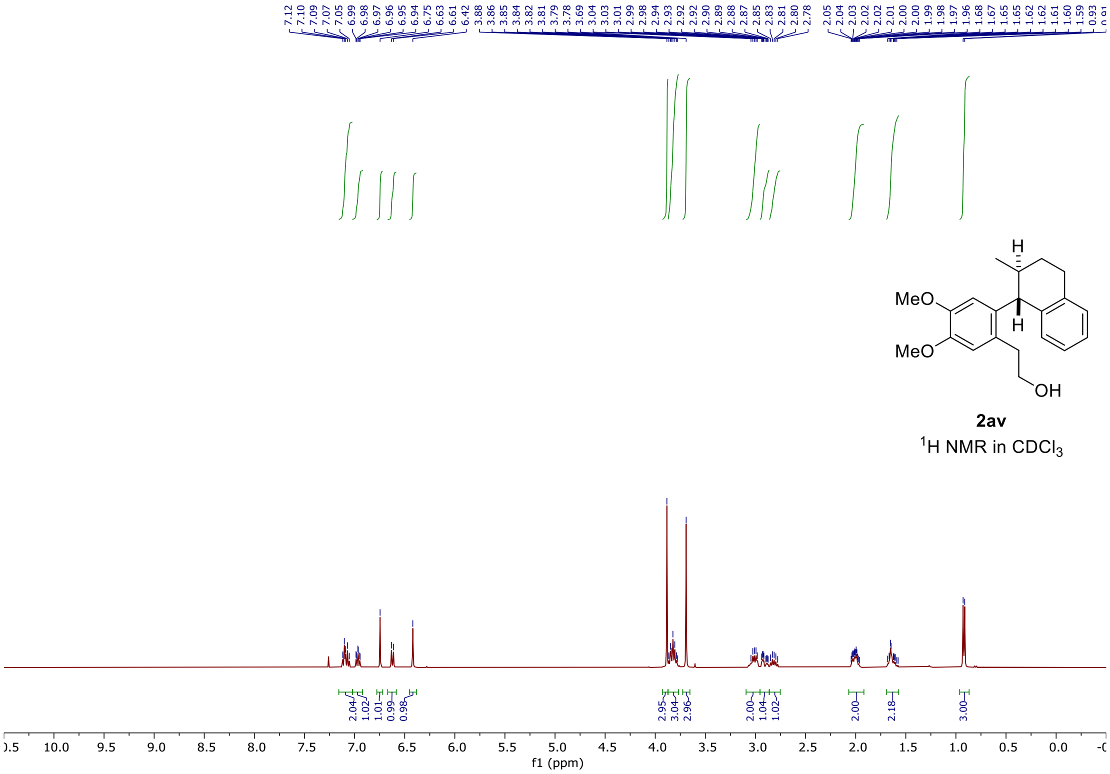
7.12
7.10
7.09
7.07
7.05
6.99
6.98
6.97
6.96
6.95
6.94
6.75
6.63
6.61
6.42
3.88
3.86
3.85
3.84
3.82
3.81
3.79
3.78
3.69
3.04
3.03
3.01
2.99
2.98
2.94
2.93
2.92
2.92
2.90
2.89
2.88
2.87
2.85
2.83
2.81
2.80
2.78
2.05
2.04
2.03
2.02
2.02
2.01
2.00
2.00
1.99
1.98
1.97
1.96
1.68
1.67
1.65
1.65
1.62
1.62
1.61
1.60
1.59
0.93
MeO
MeO
H
H
OH
2av
1H NMR in CDCl3
2.04
1.02
1.01
0.99
0.98
2.95
3.04
2.96
2.00
1.04
1.02
2.00
2.18
3.00
f1 (ppm)

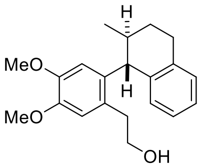

2av

^{13}C NMR in $CDCl_3$

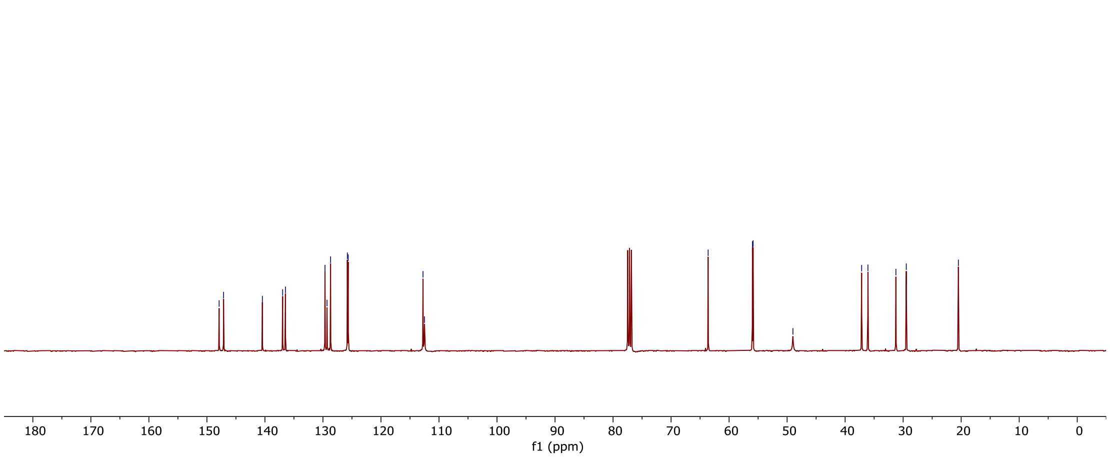

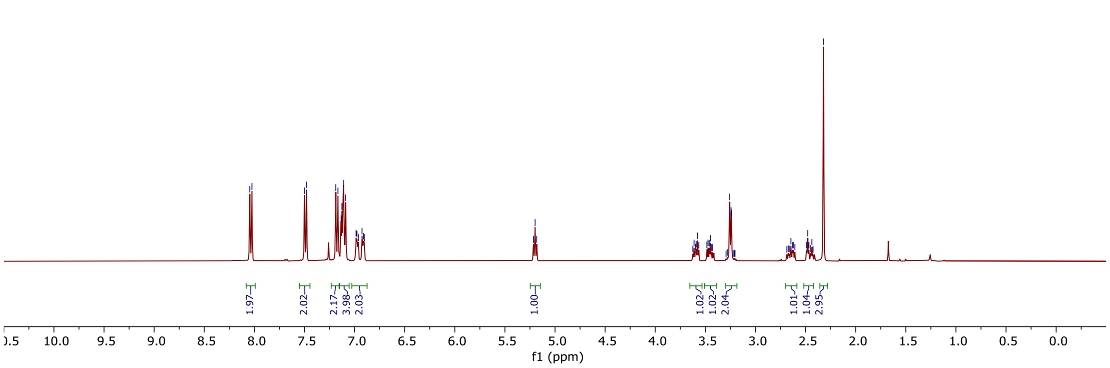

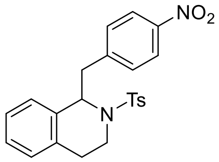

3a

^{1}H NMR in $CDCl_3$

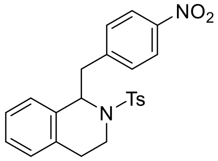

3a

^{13}C NMR in $CDCl_3$

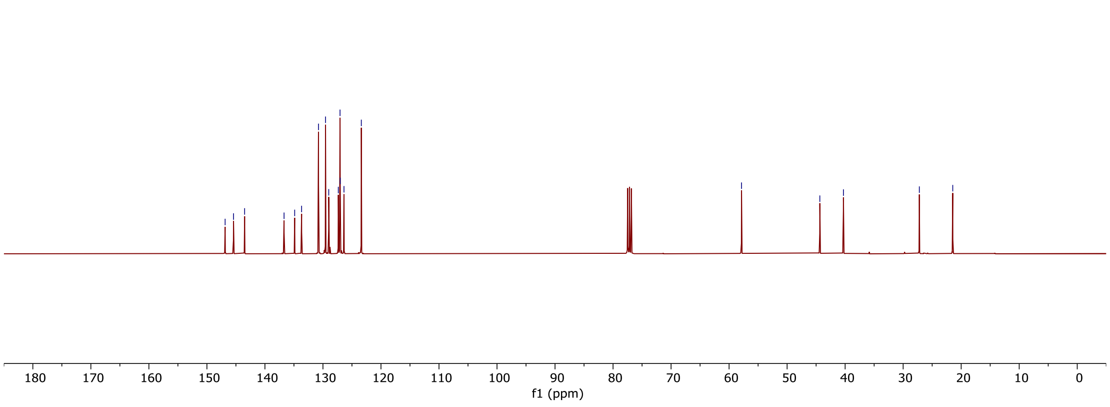

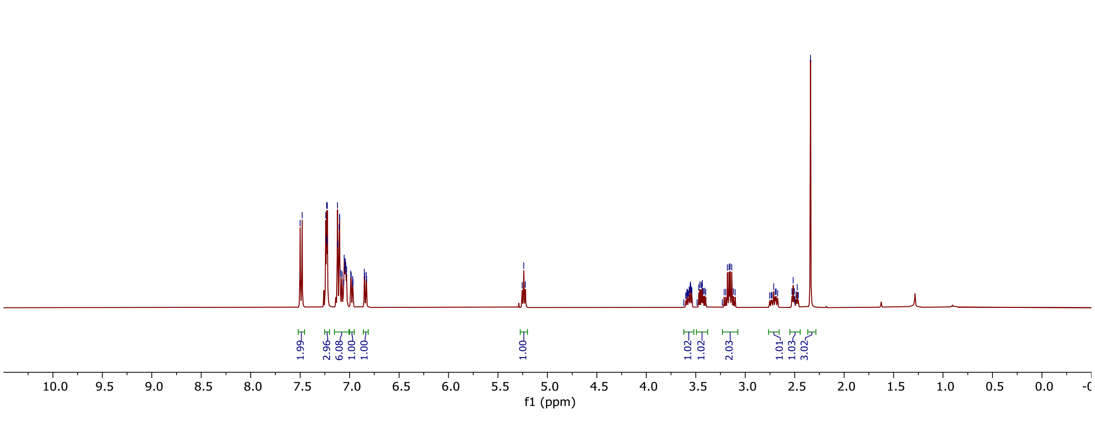

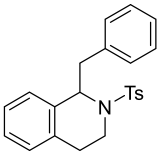

3b

^{1}H NMR in $CDCl_3$

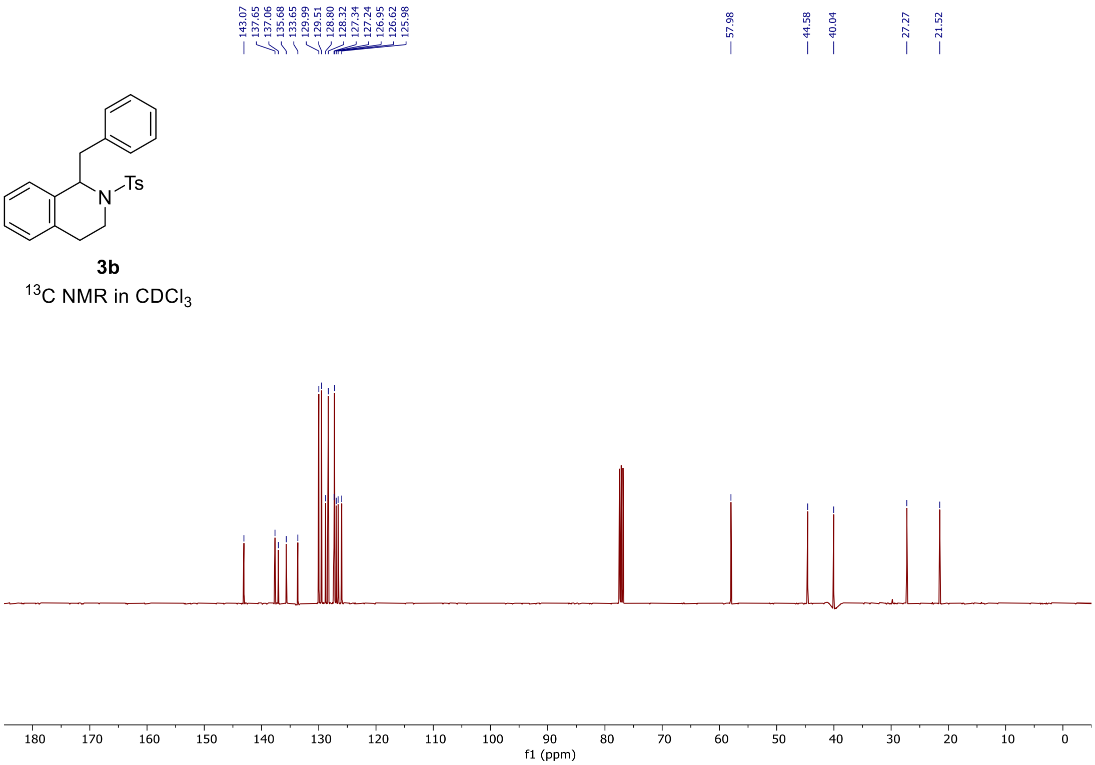

3b

^{13}C NMR in $CDCl_3$

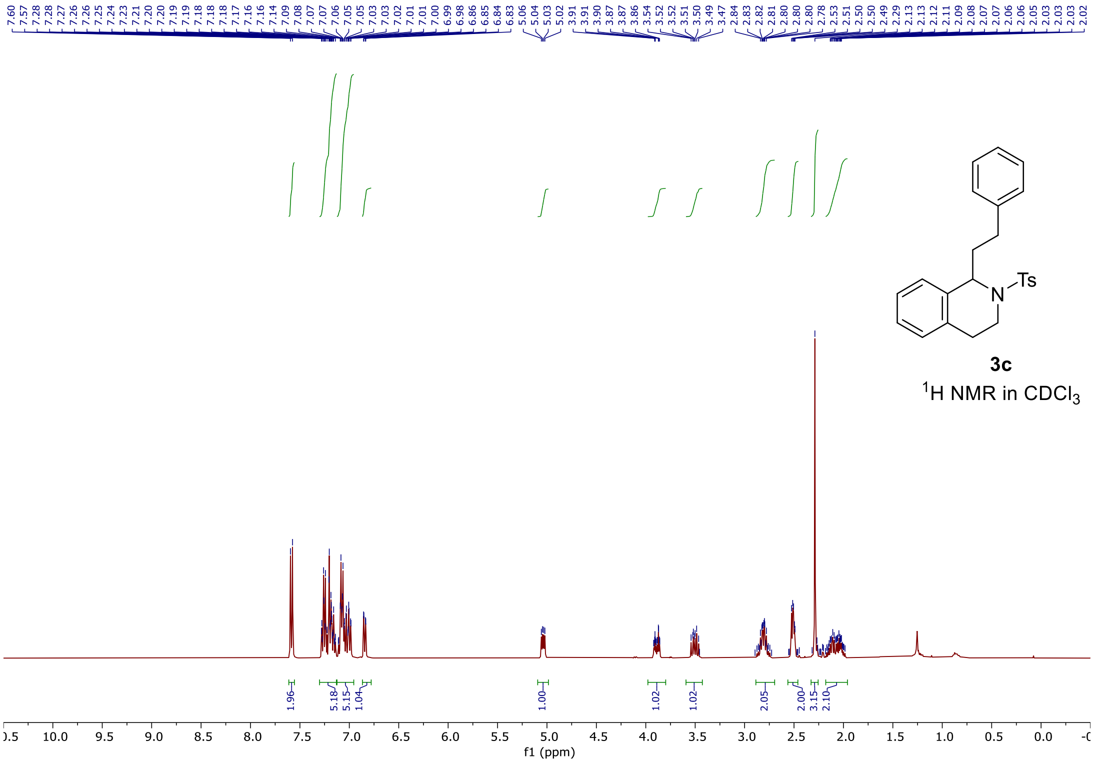

3c

^{1}H NMR in $CDCl_3$

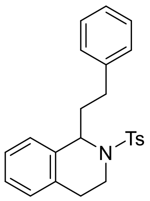

3c

^{13}C NMR in $CDCl_3$

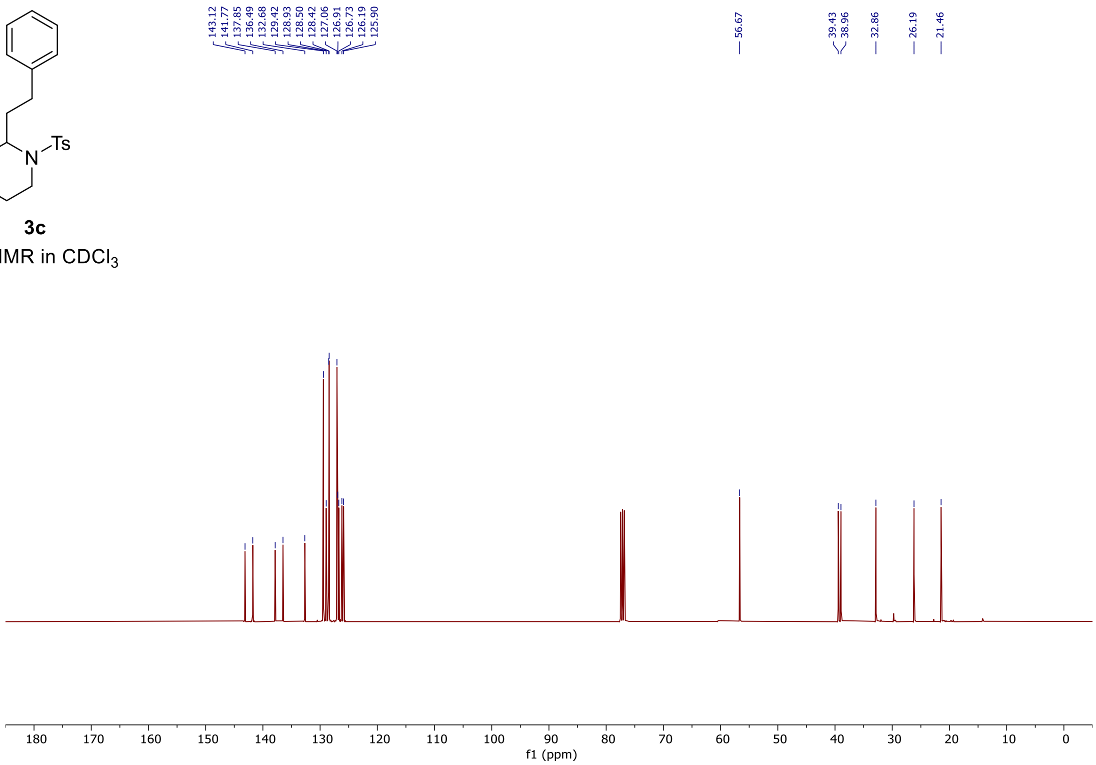

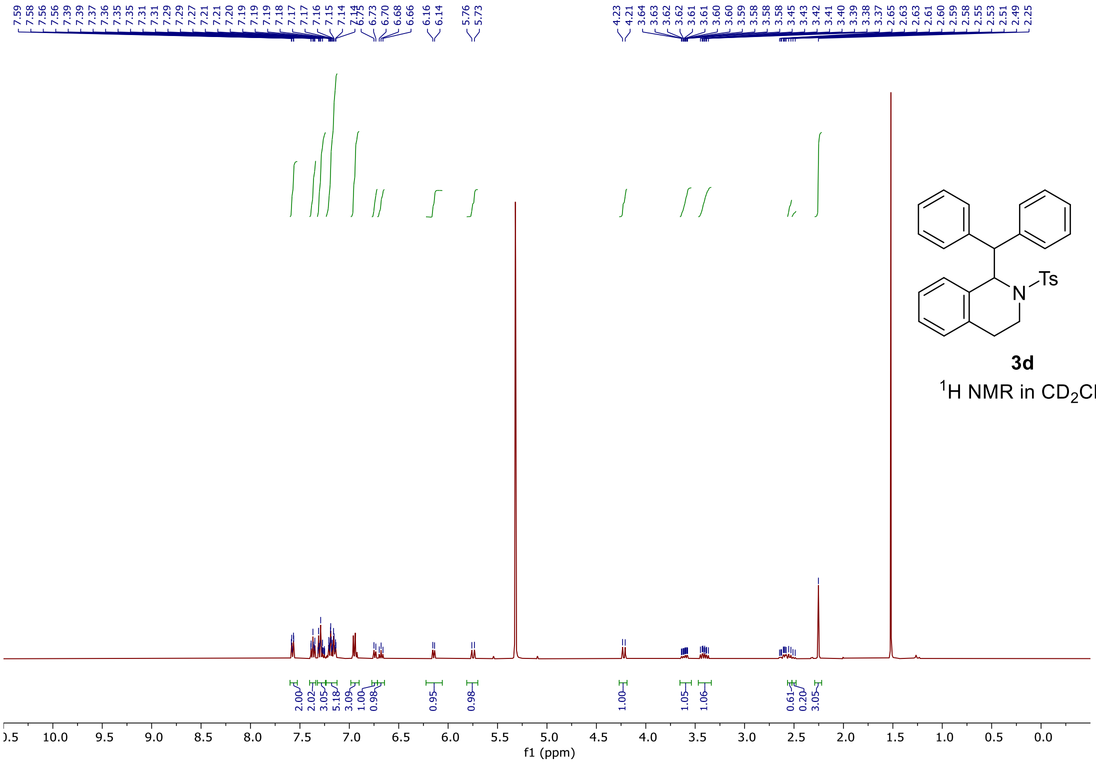

3d

^1H NMR in CD_2Cl_2

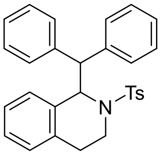

3d

^{13}C NMR in CD_2Cl_2

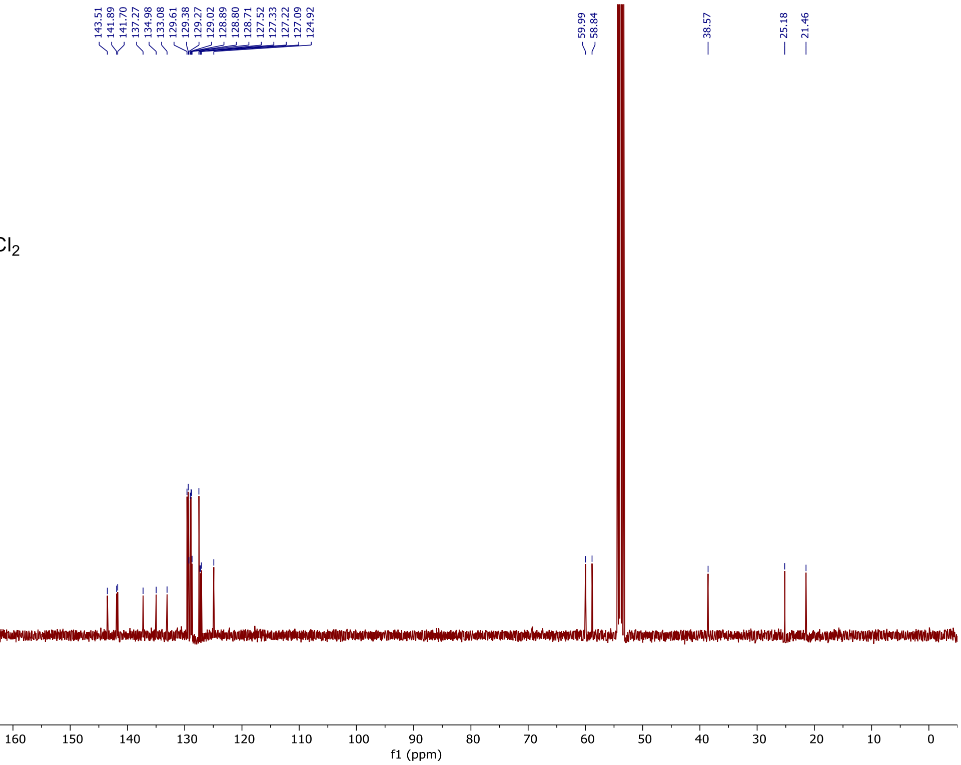

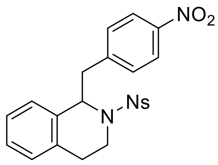

3e

^{1}H NMR in $CDCl_3$

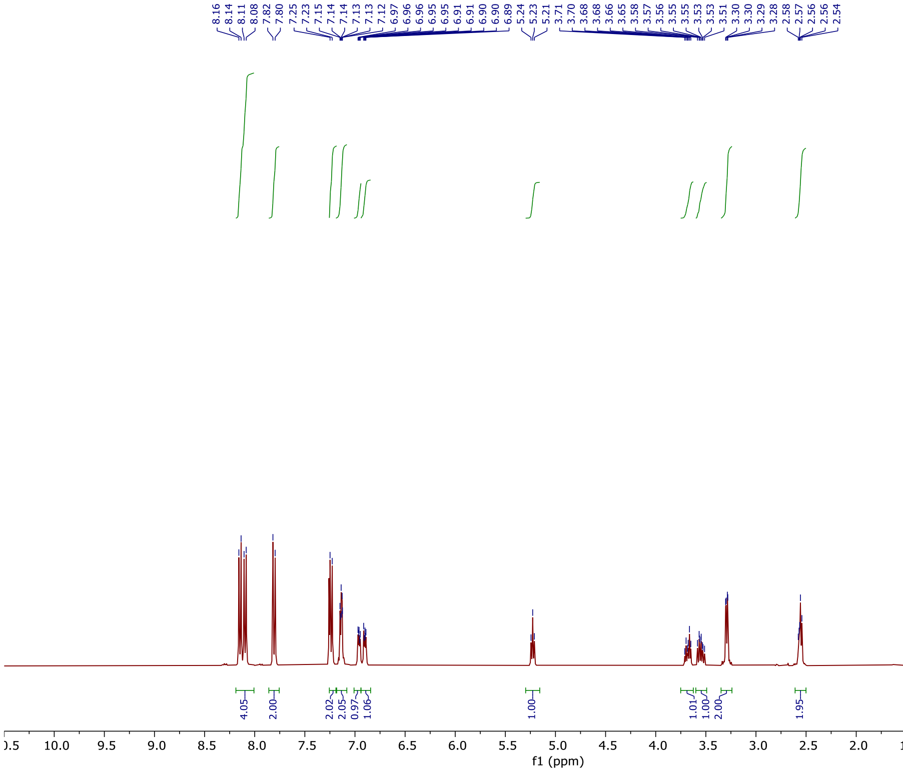

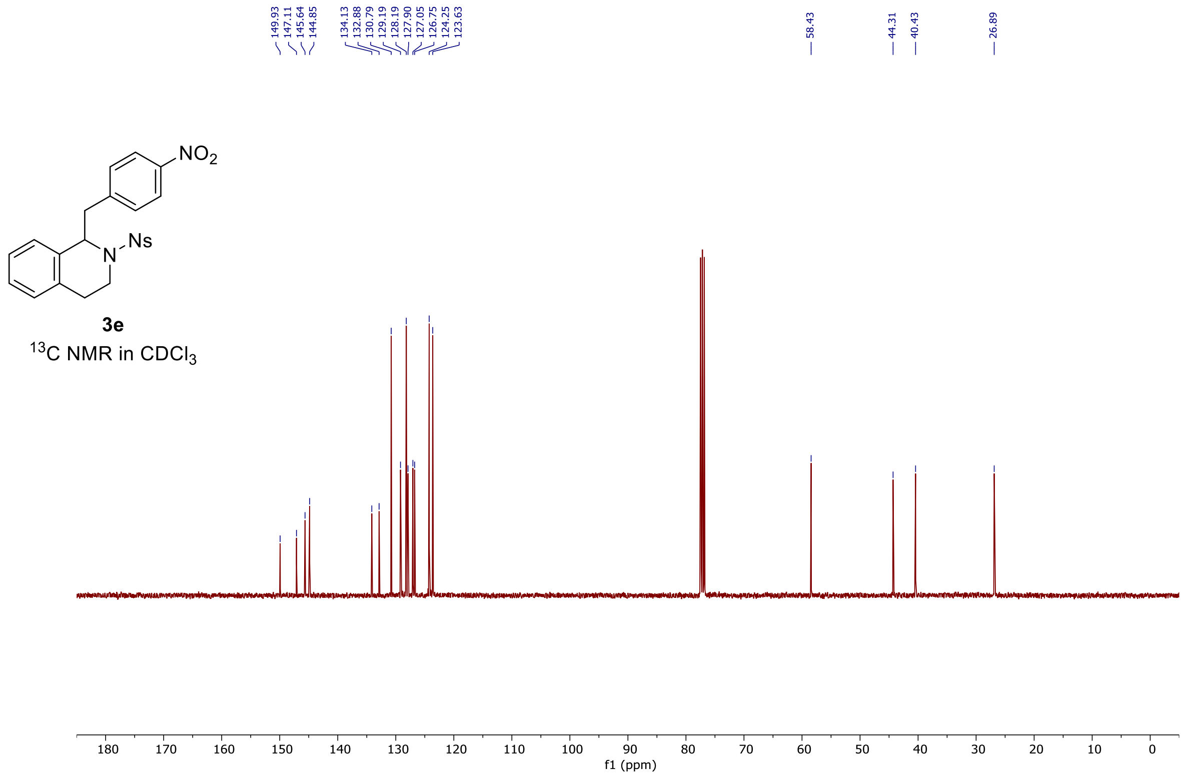

3e

^{13}C NMR in $CDCl_3$

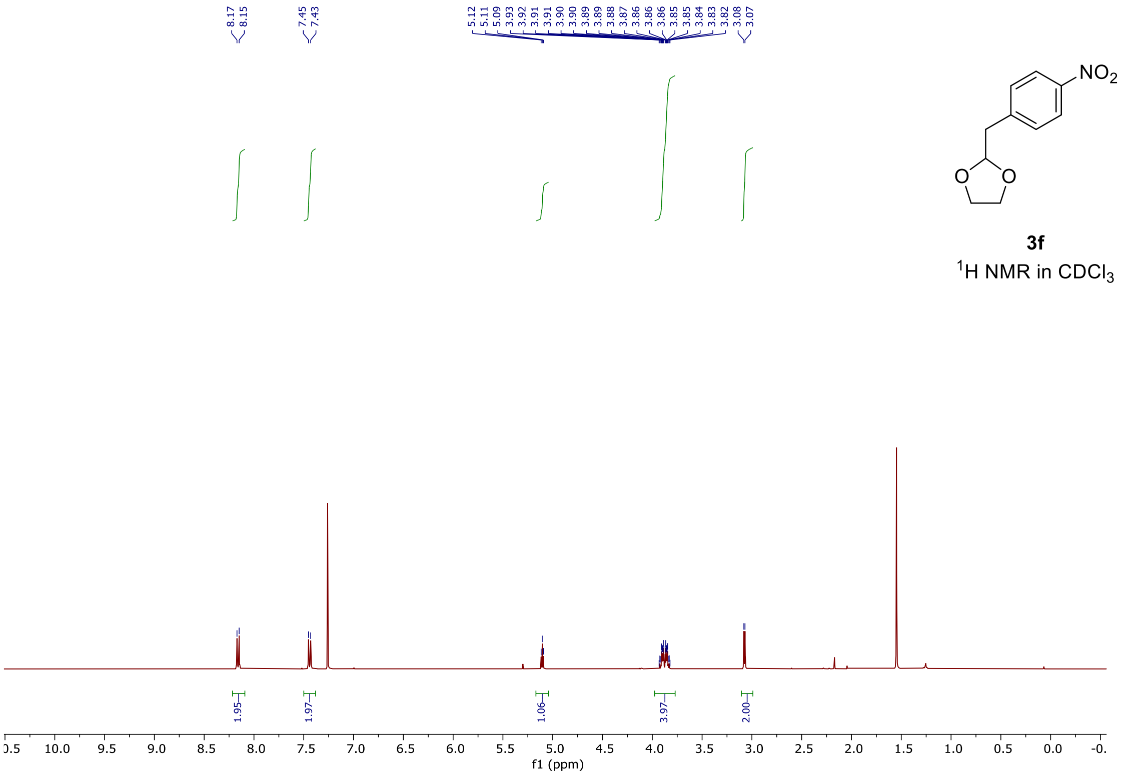

3f

^{1}H NMR in $CDCl_3$

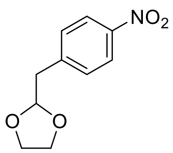

3f

^{13}C NMR in $CDCl_3$

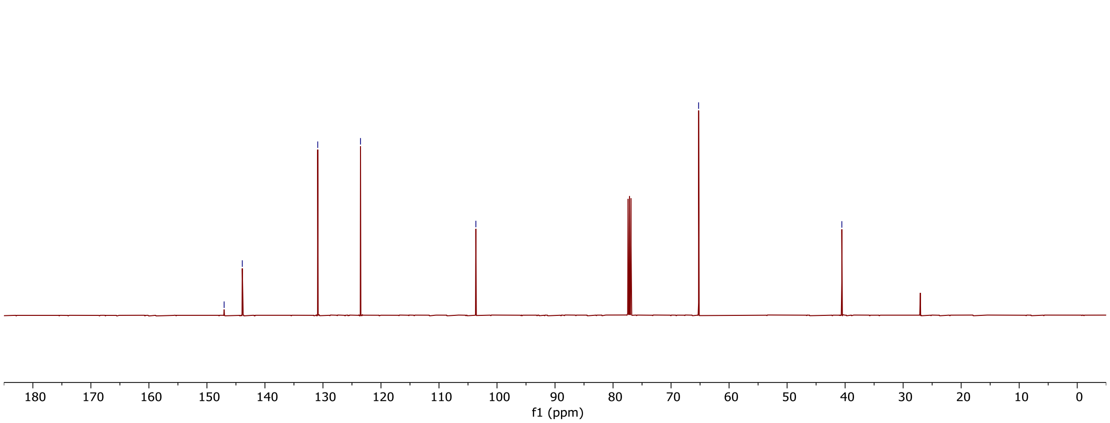

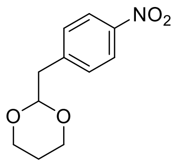

3g

^{1}H NMR in $CDCl_3$

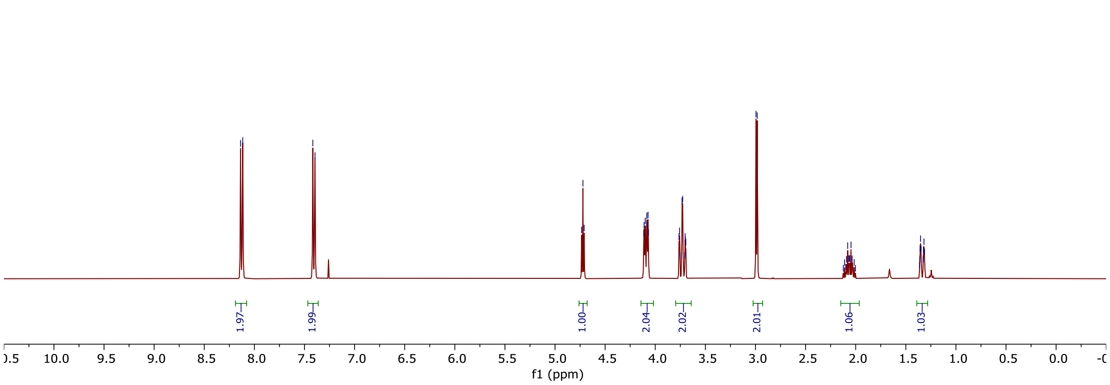

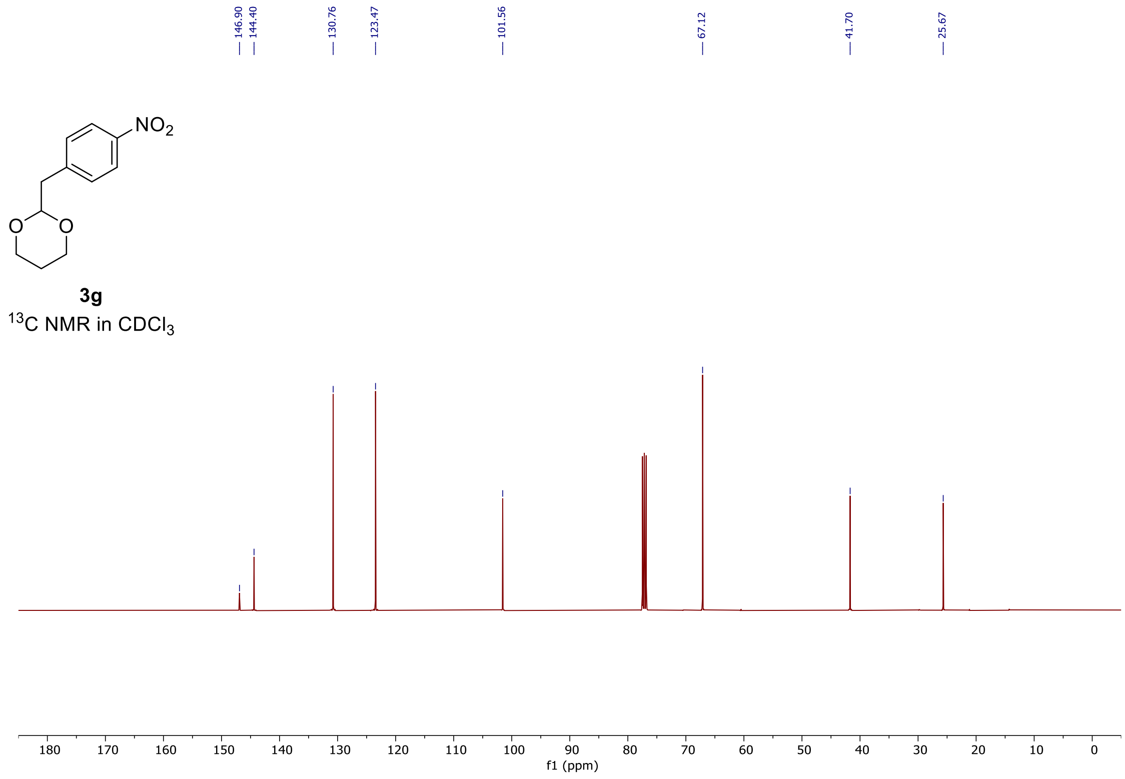

146.90
144.40
130.76
123.47
101.56
67.12
41.70
25.67
NO_2
O
O
3g
^{13}C NMR in $CDCl_3$
180 170 160 150 140 130 120 110 100 90 80 70 60 50 40 30 20 10 0
f1 (ppm)

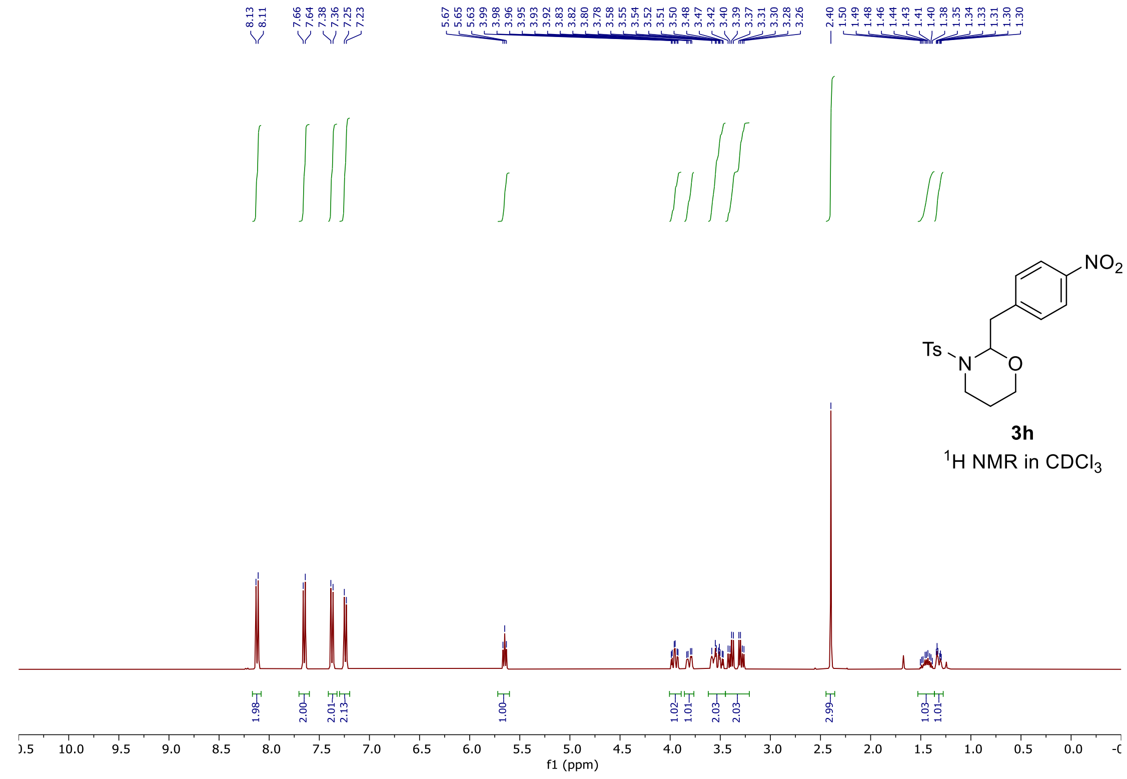

8.13
8.11
7.66
7.64
7.38
7.36
7.25
7.23
5.67
5.65
5.63
3.99
3.98
3.96
3.95
3.93
3.92
3.83
3.82
3.80
3.78
3.58
3.55
3.54
3.52
3.51
3.50
3.48
3.47
3.42
3.40
3.39
3.37
3.31
3.30
3.28
3.26
2.40
1.50
1.49
1.48
1.46
1.44
1.43
1.41
1.40
1.38
1.35
1.34
1.33
1.31
1.30
1.30
NO_2
Ts
N
O
3h
^{1}H NMR in $CDCl_3$
1.98
2.00
2.01
2.13
1.00
1.02
1.01
2.03
2.03
2.99
1.03
1.01
10.0 9.5 9.0 8.5 8.0 7.5 7.0 6.5 6.0 5.5 5.0 4.5 4.0 3.5 3.0 2.5 2.0 1.5 1.0 0.5 0.0
f1 (ppm)

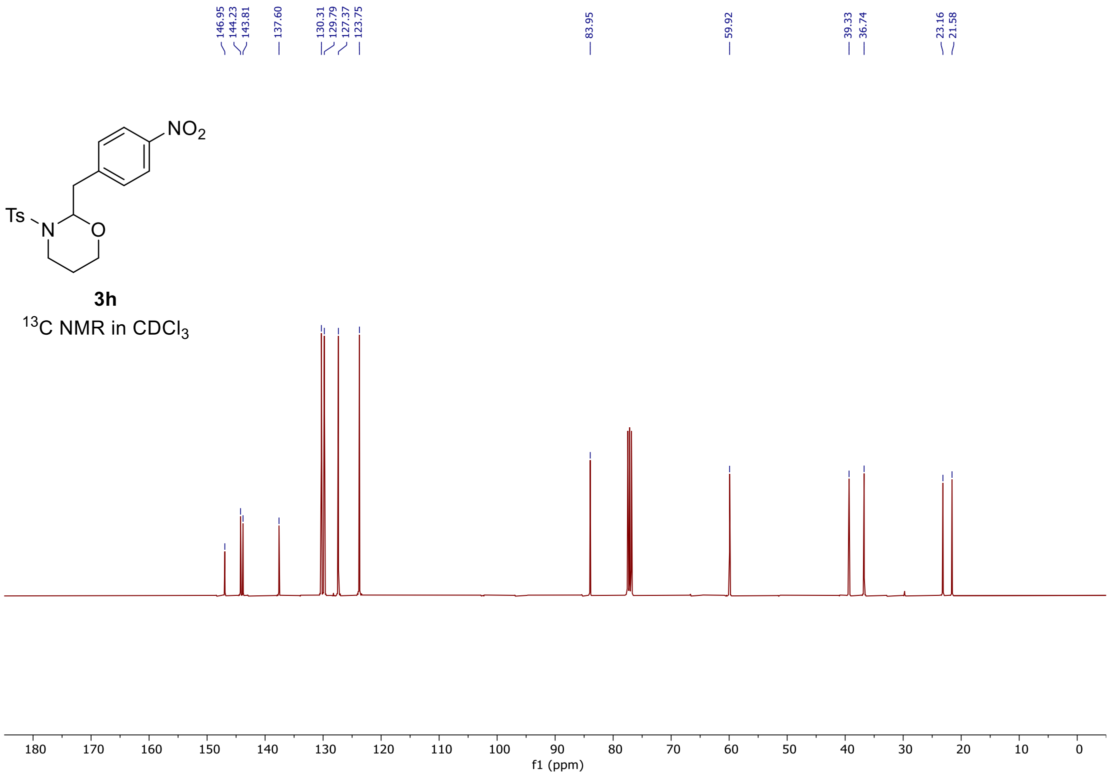

146.95
144.23
143.81
137.60
130.31
129.79
127.37
123.75
83.95
59.92
39.33
36.74
23.16
21.58
NO2
Ts
N
O
3h
13C NMR in CDCl3
f1 (ppm)

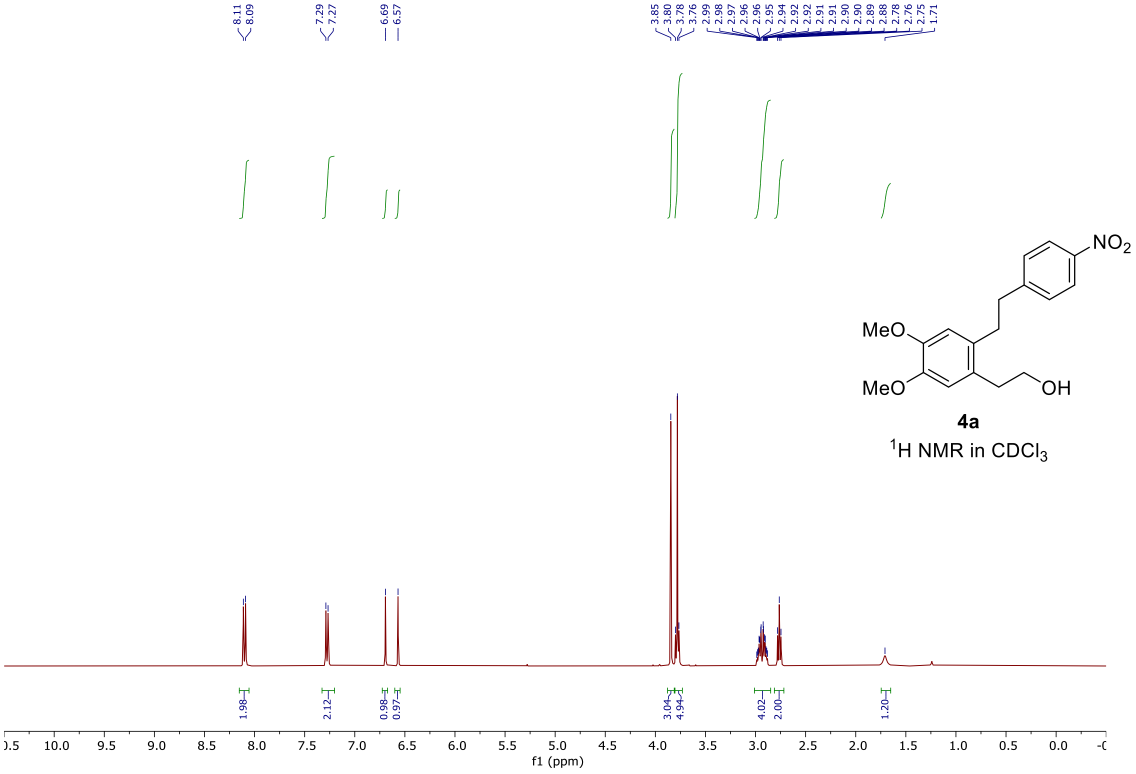

8.11
8.09
7.29
7.27
6.69
6.57
3.85
3.80
3.78
3.76
2.99
2.98
2.97
2.96
2.96
2.95
2.94
2.92
2.92
2.91
2.91
2.90
2.90
2.89
2.88
2.78
2.76
2.75
1.71
NO2
MeO
MeO
OH
4a
1H NMR in CDCl3
1.98
2.12
0.98
0.97
3.04
4.94
4.02
2.00
1.20
f1 (ppm)

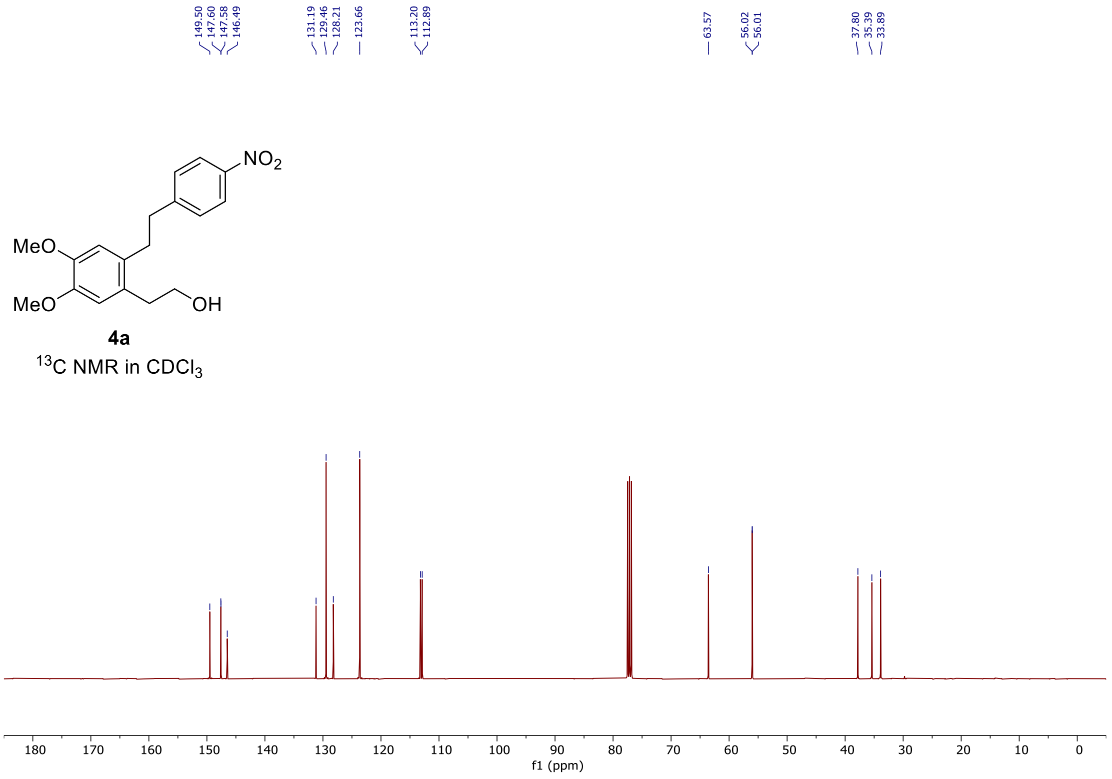

4a

^{13}C NMR in $CDCl_3$

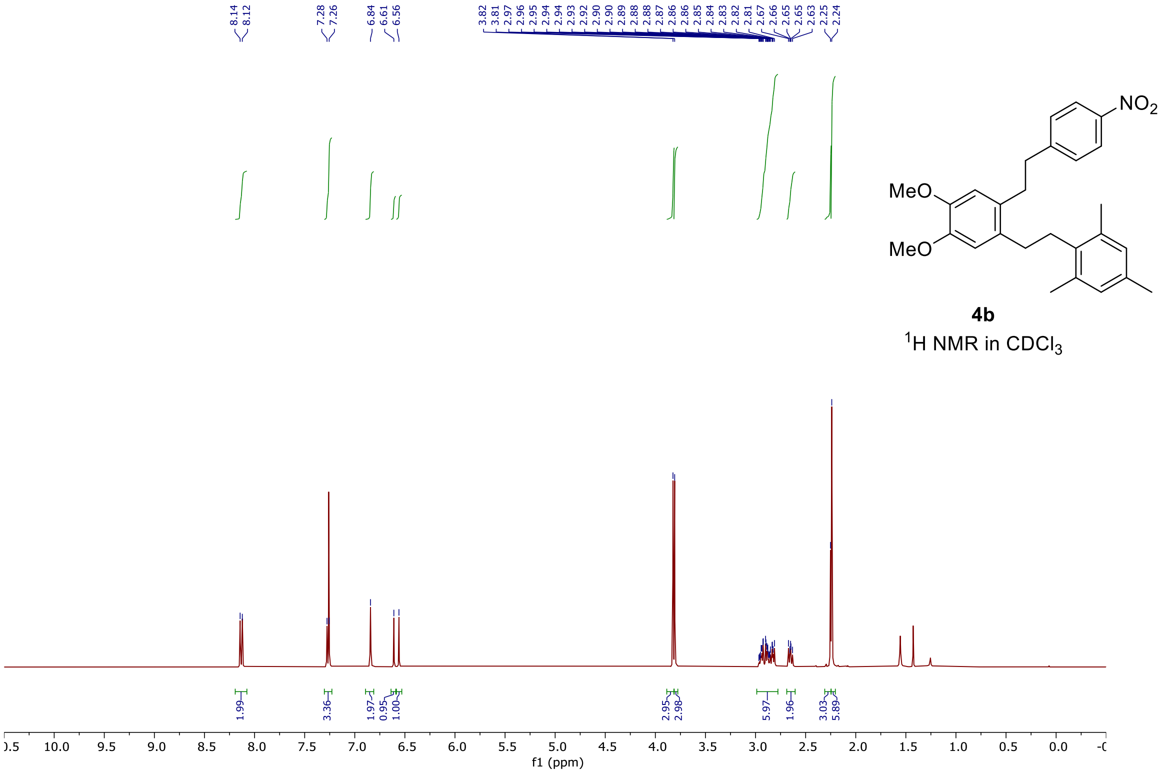

4b

^{1}H NMR in $CDCl_3$

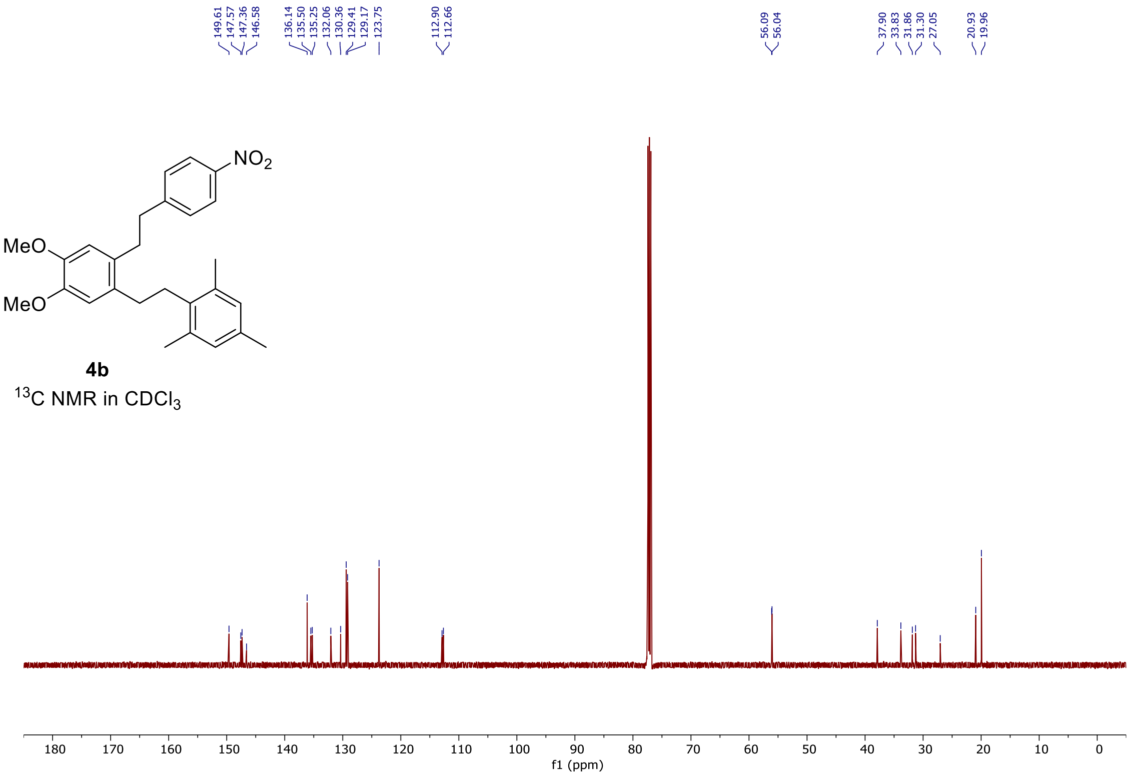
149.61
147.57
147.36
146.58
136.14
135.50
135.25
132.06
130.36
129.41
129.17
123.75
112.90
112.66
56.09
56.04
37.90
33.83
31.86
31.30
27.05
20.93
19.96
NO_2
MeO
MeO
4b
^{13}C NMR in $CDCl_3$
180 170 160 150 140 130 120 110 100 90 80 70 60 50 40 30 20 10 0
f1 (ppm)

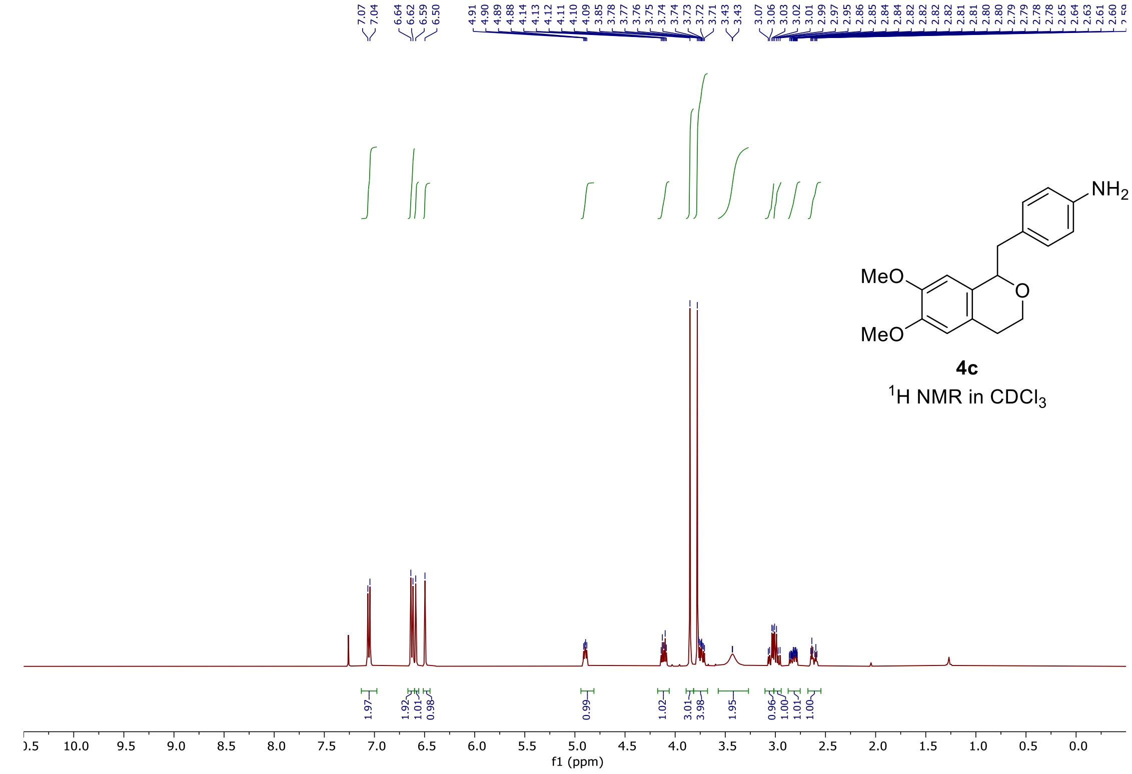
7.07
7.04
6.64
6.62
6.59
6.50
4.91
4.90
4.89
4.88
4.14
4.13
4.12
4.11
4.10
4.09
3.85
3.78
3.77
3.76
3.75
3.74
3.74
3.73
3.72
3.71
3.43
3.43
3.07
3.06
3.03
3.02
3.01
2.99
2.97
2.95
2.86
2.85
2.84
2.82
2.82
2.82
2.82
2.81
2.80
2.79
2.78
2.65
2.64
2.63
2.61
2.60
NH_2
MeO
MeO
O
4c
^{1}H NMR in $CDCl_3$
1.97
1.92
1.01
0.98
0.99
1.02
3.01
3.98
1.95
0.96
1.00
1.01
1.00
10.0 9.5 9.0 8.5 8.0 7.5 7.0 6.5 6.0 5.5 5.0 4.5 4.0 3.5 3.0 2.5 2.0 1.5 1.0 0.5 0.0
f1 (ppm)

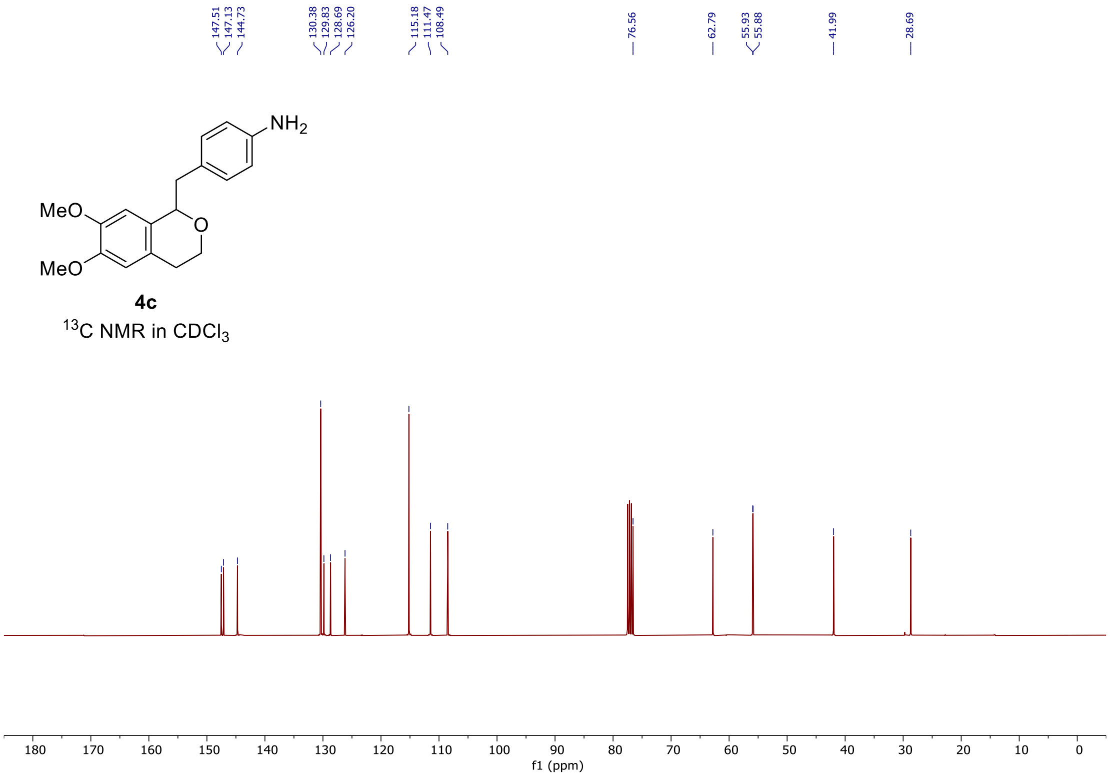
147.51
147.13
144.73
130.38
129.83
128.69
126.20
115.18
111.47
108.49
76.56
62.79
55.93
55.88
41.99
28.69
NH2
MeO
MeO
O
4c
13C NMR in CDCl3
f1 (ppm)

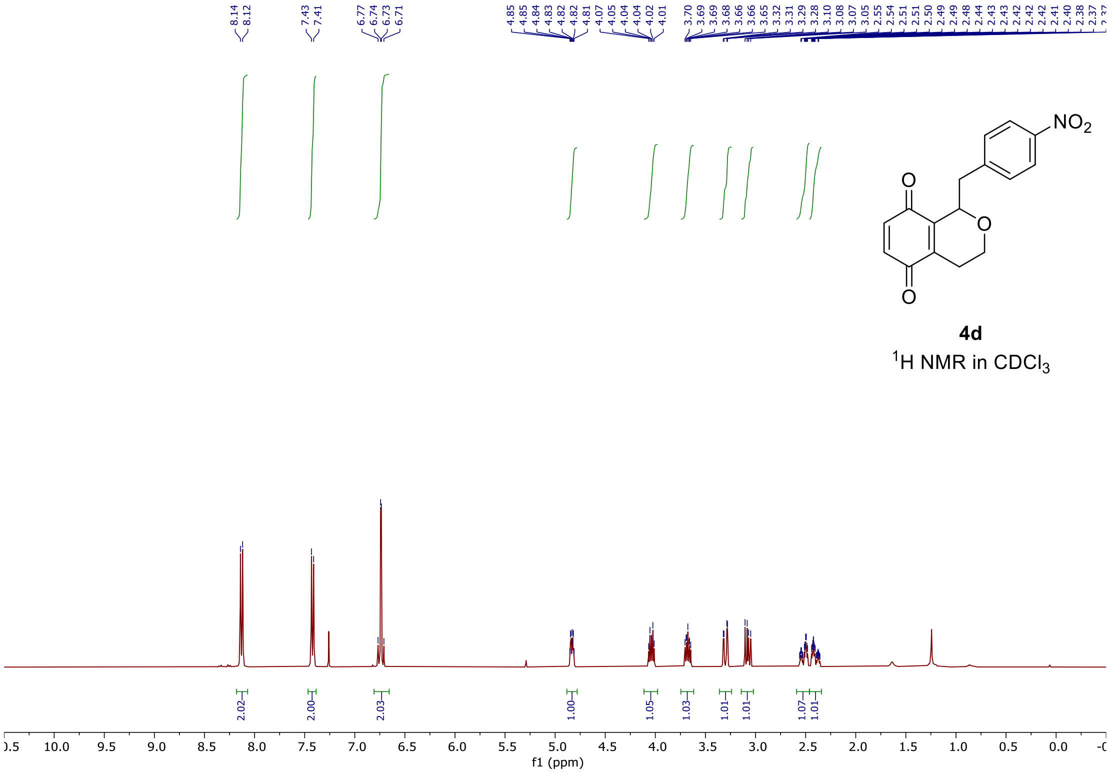
NO2
O
O
O
4d
1H NMR in CDCl3
f1 (ppm)

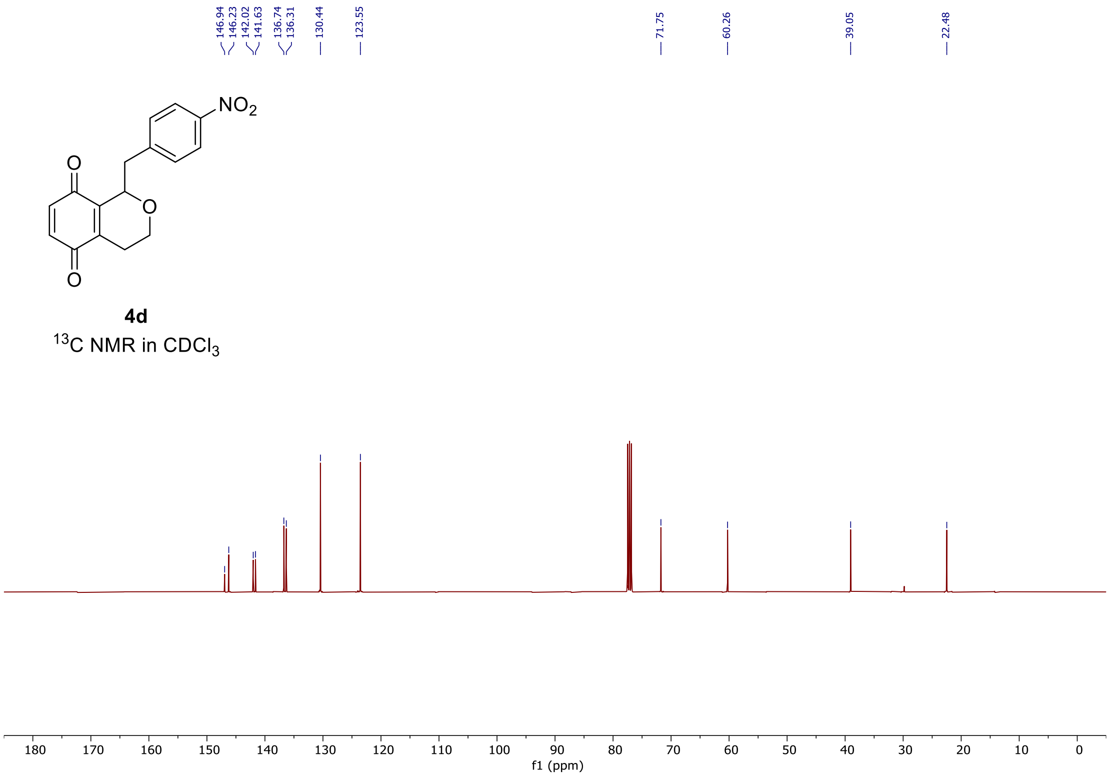
146.94
146.23
142.02
141.63
136.74
136.31
130.44
123.55
71.75
60.26
39.05
22.48
NO2
O
O
O
4d
13C NMR in CDCl3
180 170 160 150 140 130 120 110 100 90 80 70 60 50 40 30 20 10 0
f1 (ppm)

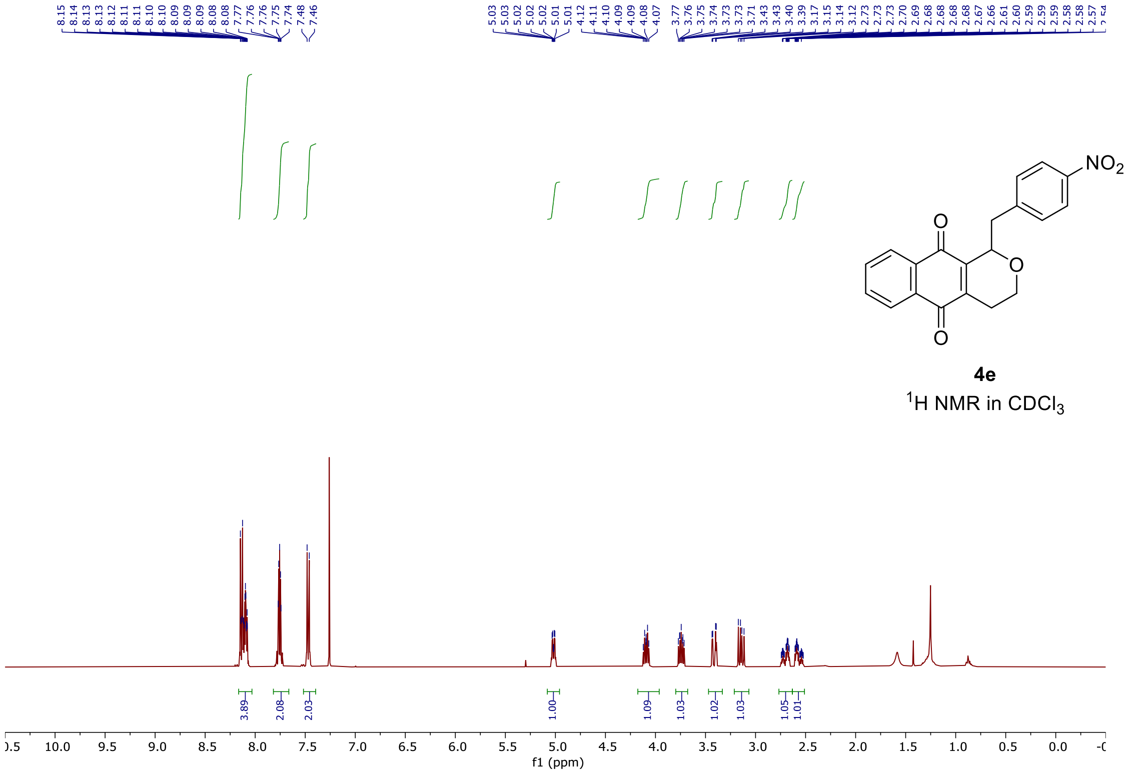
8.15 8.14 8.13 8.13 8.12 8.11 8.11 8.10 8.10 8.09 8.09 8.09 8.08 8.08 7.77 7.76 7.76 7.75 7.74 7.48 7.46
5.03 5.03 5.02 5.02 5.01 5.01 4.12 4.11 4.10 4.09 4.09 4.08 4.07 3.77 3.76 3.75 3.74 3.73 3.73 3.71 3.43 3.40 3.39 3.17 3.15 3.14 3.12 2.73 2.73 2.73 2.70 2.69 2.68 2.68 2.68 2.68 2.67 2.66 2.61 2.60 2.59 2.59 2.58 2.58 2.57 [illegible]
NO2
O
O
O
4e
1H NMR in CDCl3
3.89
2.08
2.03
1.00
1.09
1.03
1.02
1.03
1.05
1.01
10.0 9.5 9.0 8.5 8.0 7.5 7.0 6.5 6.0 5.5 5.0 4.5 4.0 3.5 3.0 2.5 2.0 1.5 1.0 0.5 0.0
f1 (ppm)

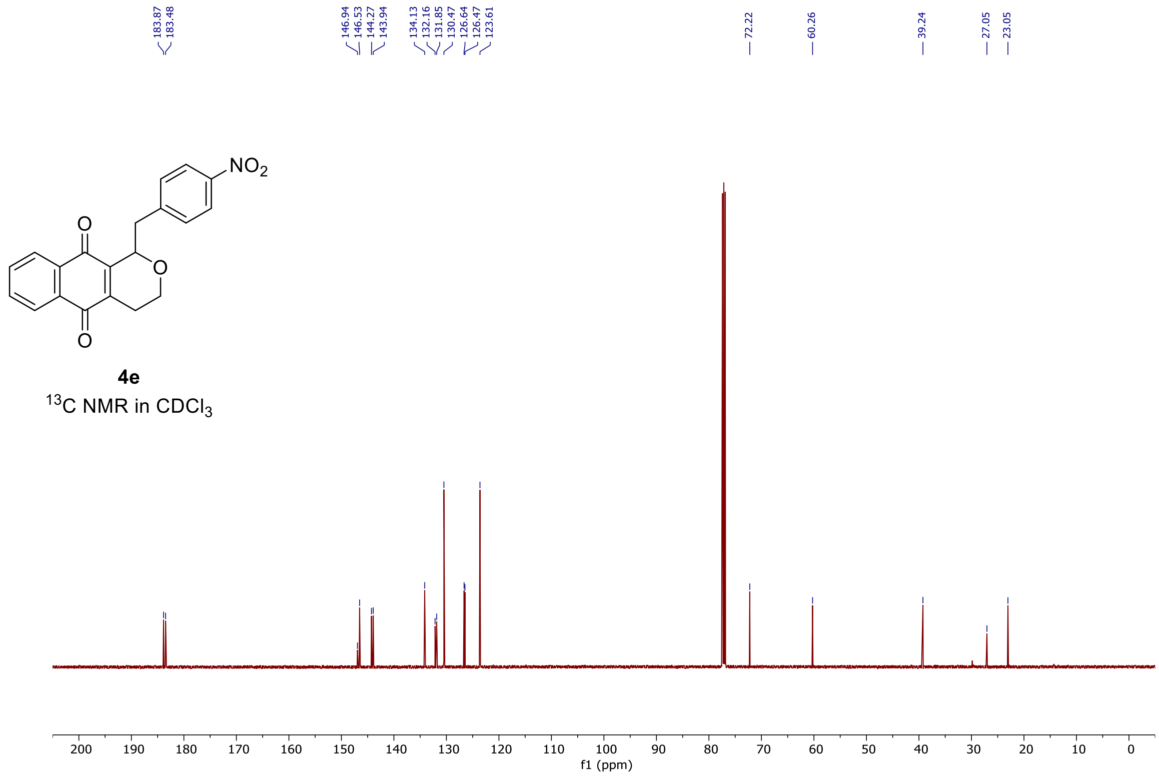

183.87
183.48
146.94
146.53
144.27
143.94
134.13
132.16
131.85
130.47
126.64
126.47
123.61
72.22
60.26
39.24
27.05
23.05
NO2
O
O
O
4e
13C NMR in CDCl3
200
190
180
170
160
150
140
130
120
110
100
90
80
70
60
50
40
30
20
10
0
f1 (ppm)

5. NOESY Analyses

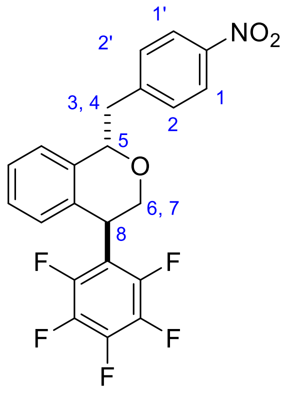

2aj

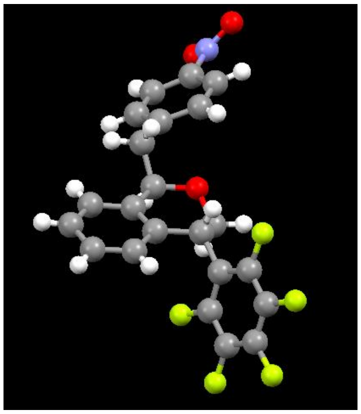

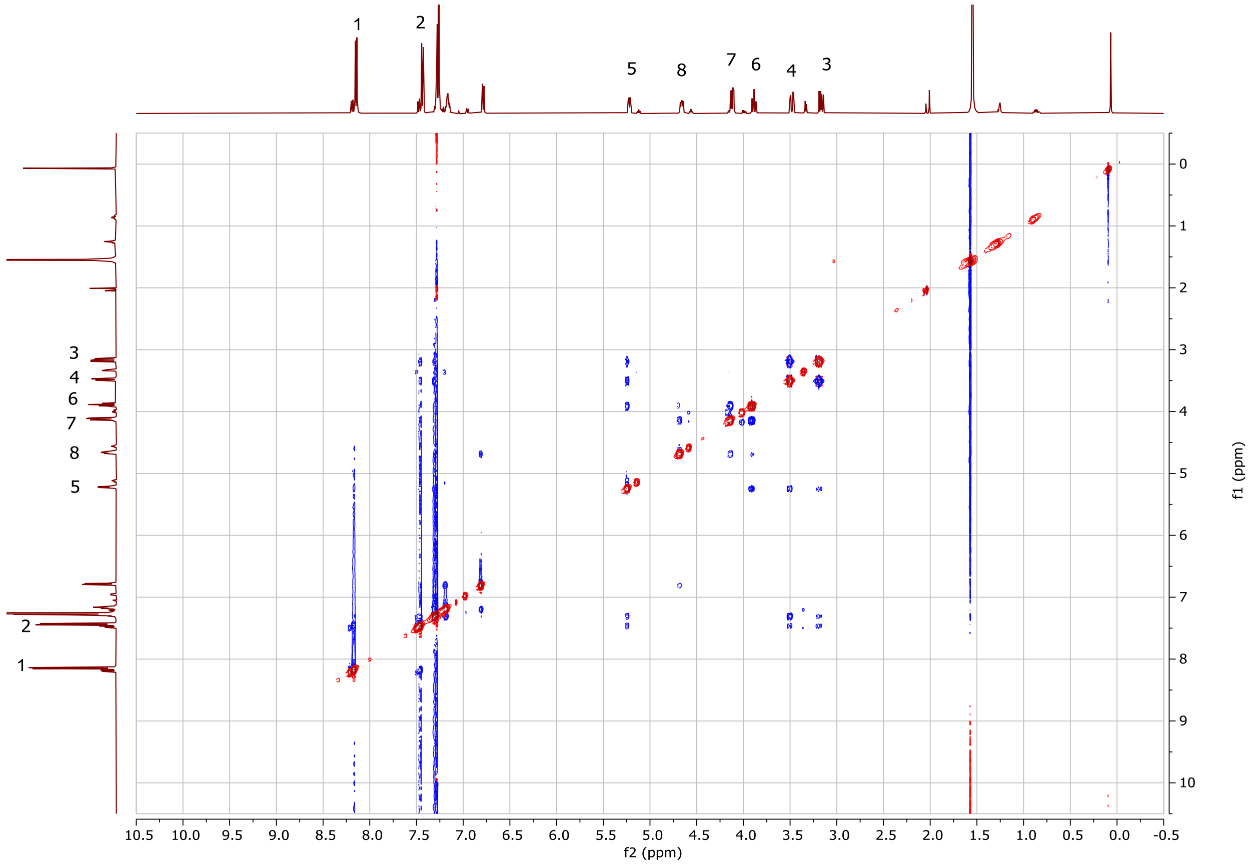

Analysis: Protons 5 and 8 do not correlate. In addition, proton 5 correlates with proton 6, but not with proton 7. Oppositely, proton 8 correlates more intensely with proton 7 than with proton 6. Put together, these observations show a *trans* relative configuration. This conclusion is also consistent with the XRD structure that was found.

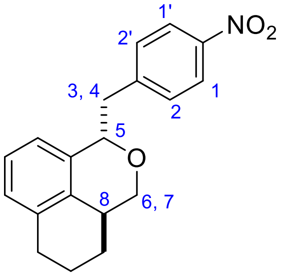

2ao

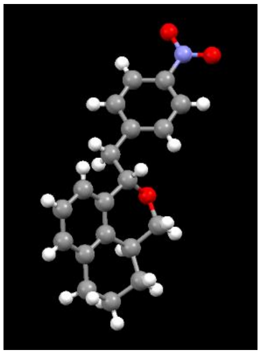

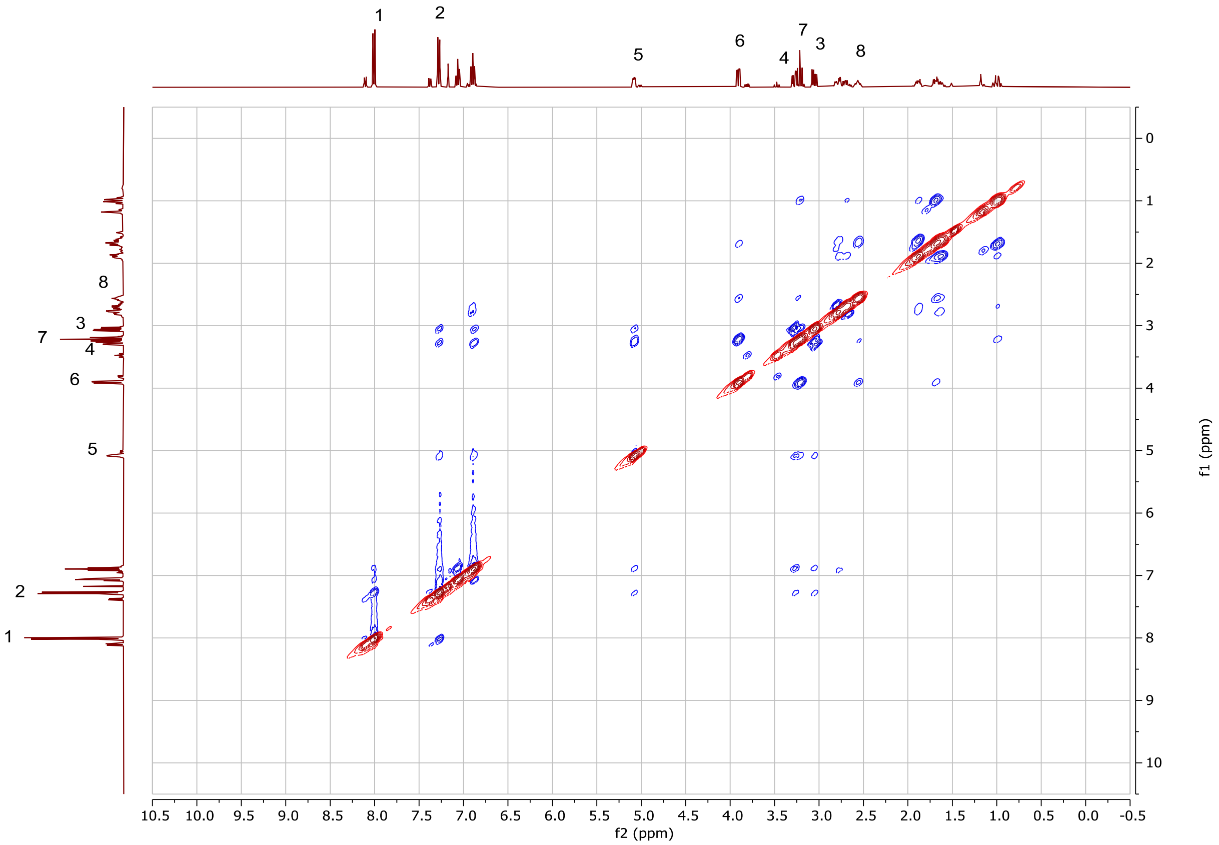

Analysis: Protons 5 and 8 do not correlate. Proton 5 correlates with proton 7 but not with proton 6. Proton 8 correlates with proton 6 more intensely than with proton 7. Put together, these observations show a *trans* relative configuration. This conclusion is also consistent with the XRD structure that was found.

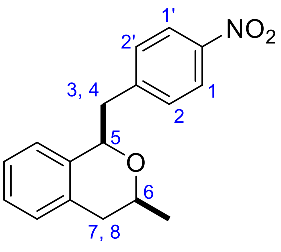

2aq

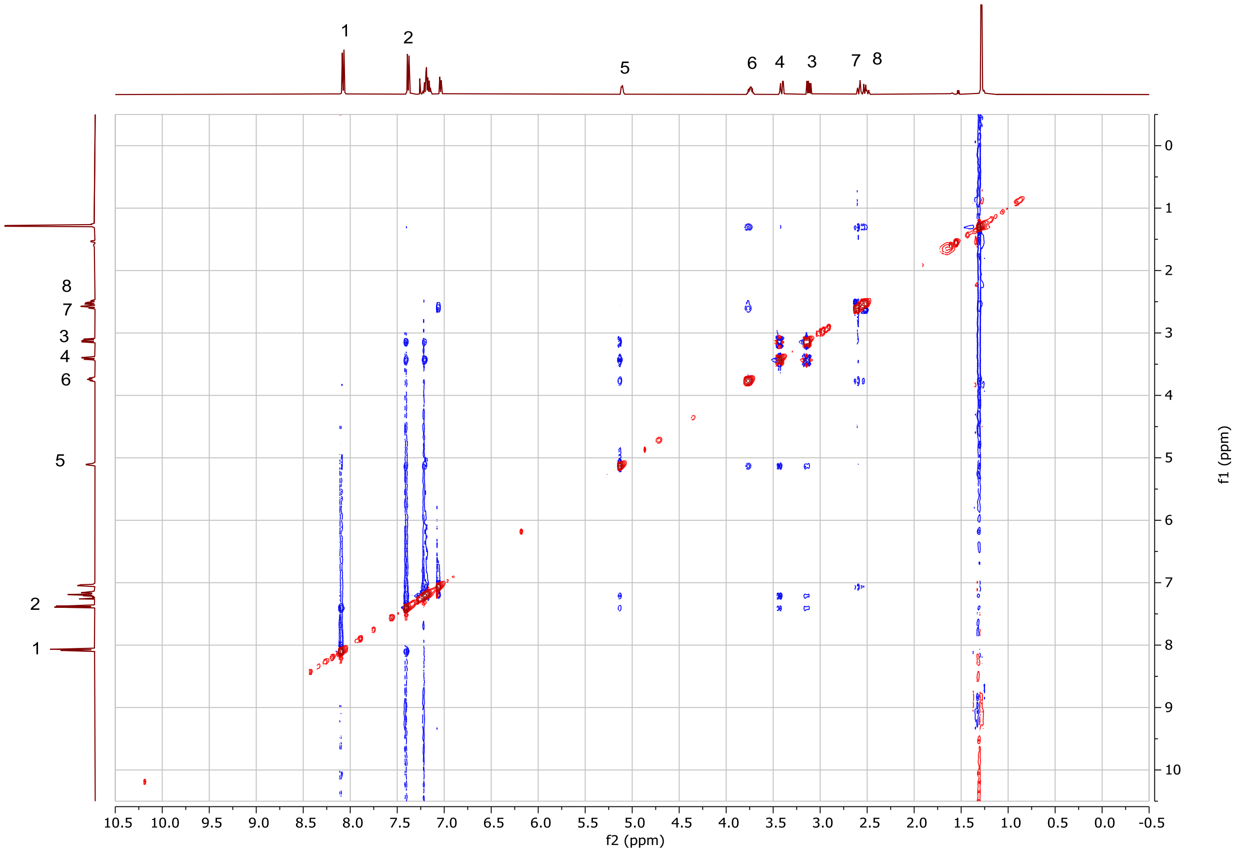

Analysis: Protons 5 and 6 correlate. Proton 6 correlates more intensely with proton 7 than with proton 8. Similarly, proton 5 correlates slightly with proton 7 but not with proton 8. Put together, these observations show a *cis* relative configuration.

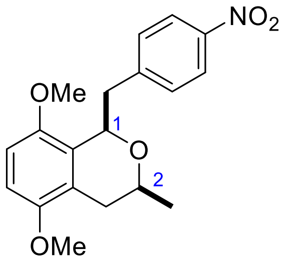

2ar

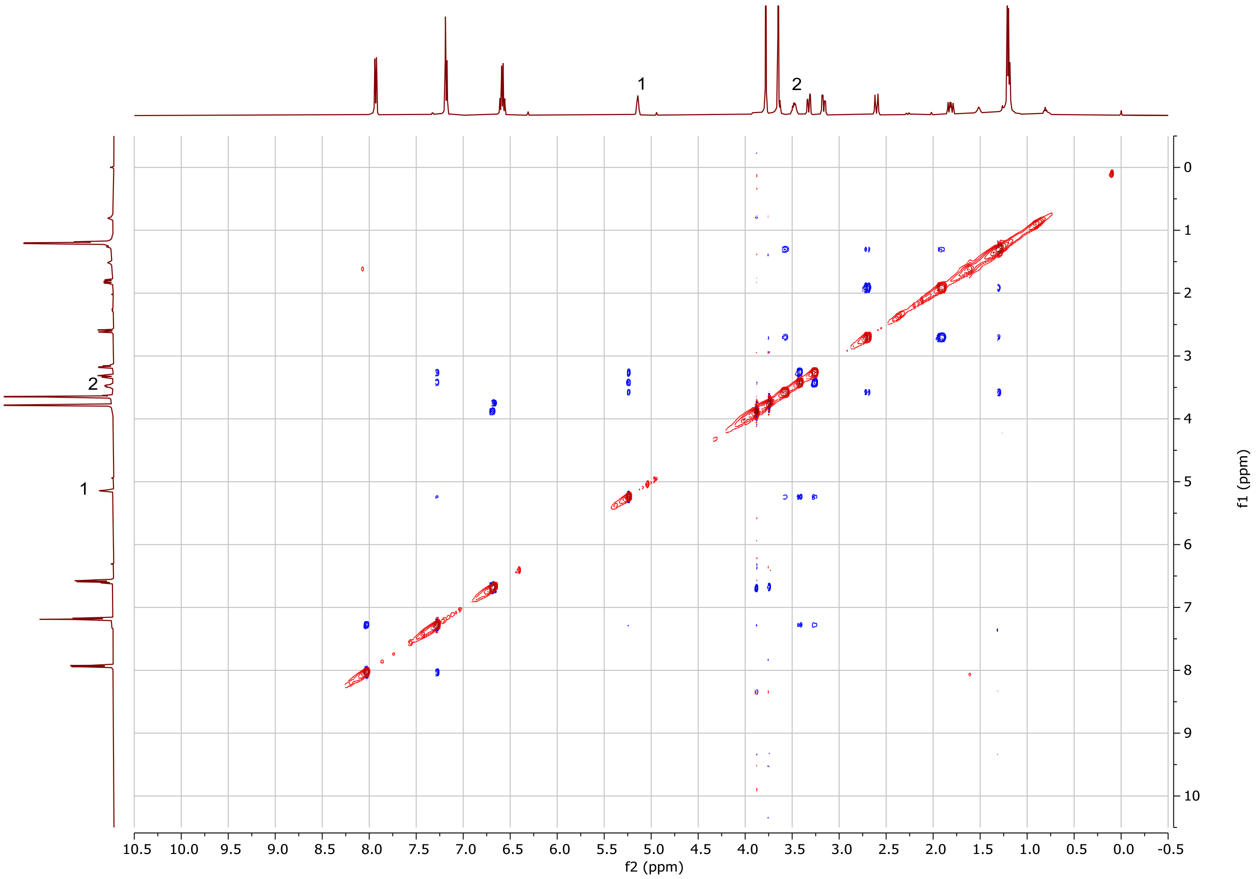

Analysis: Protons 1 and 2 correlate. This observation shows a *cis* relative configuration.

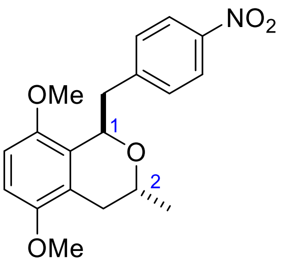

2ar

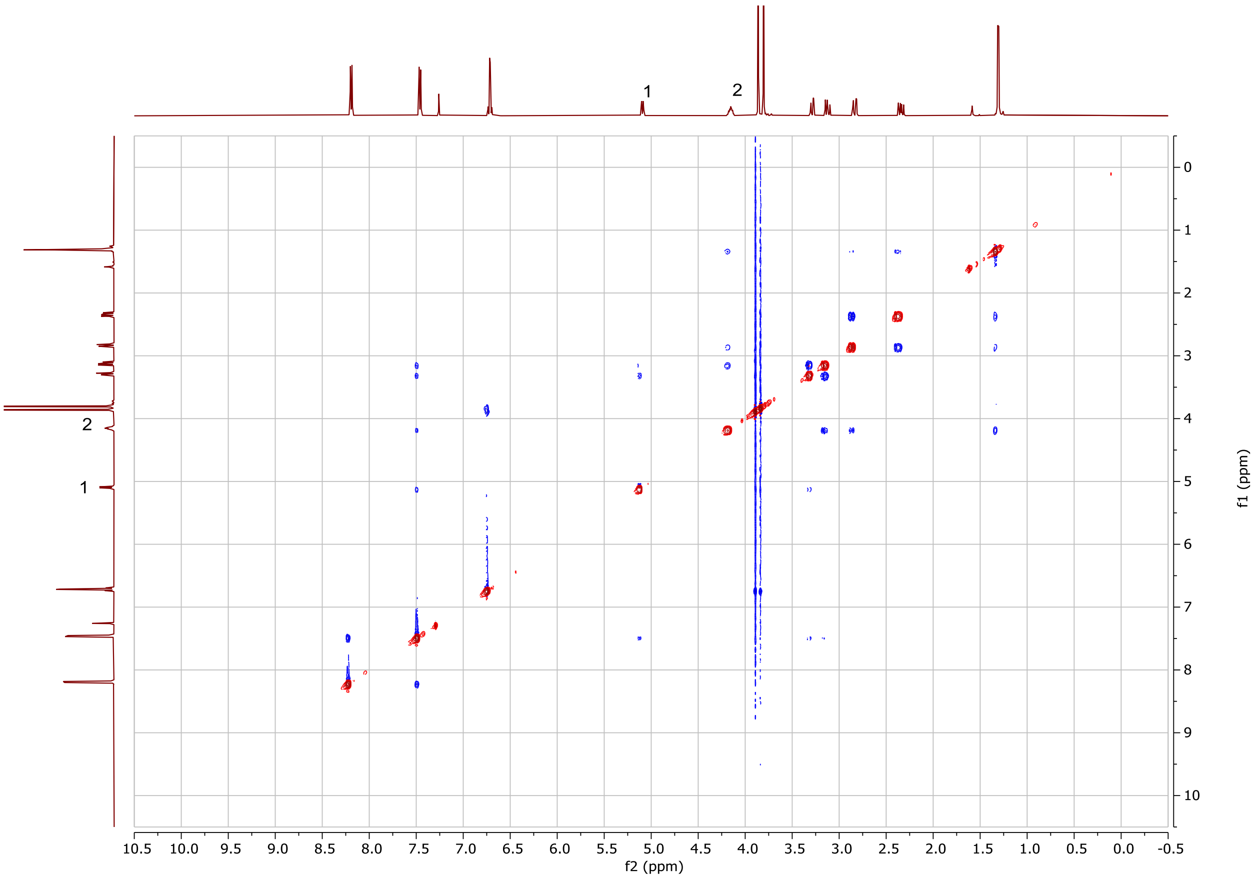

Analysis: Protons 1 and 2 do not correlate. This observation shows a *trans* relative configuration.

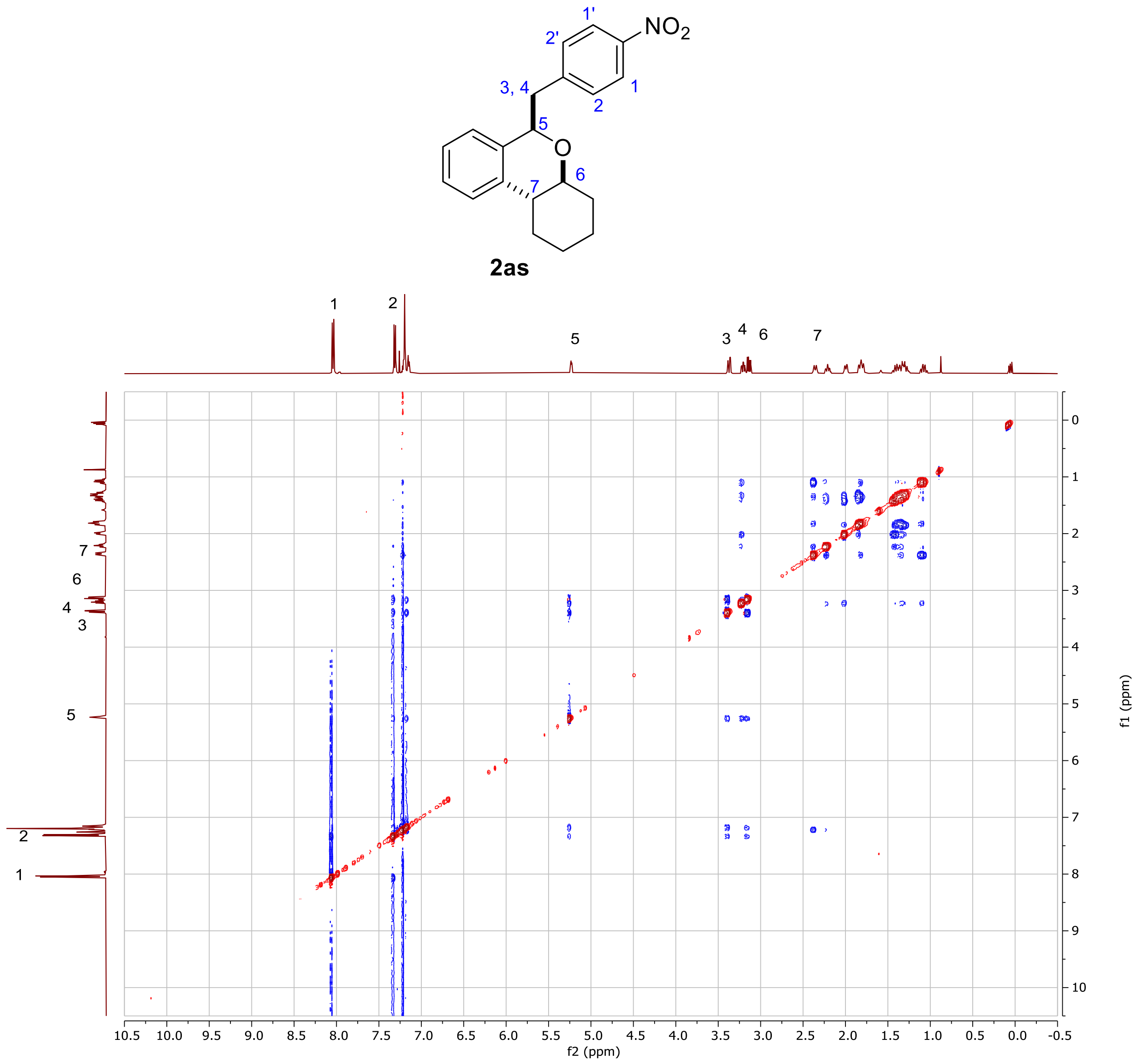

Analysis: Protons 5 and 6 correlate. Oppositely, protons 5 and 7 do not correlate. Given the original *trans* configuration of the starting alcohol, these observations show a *cis* relative configuration between protons 5 and 6.

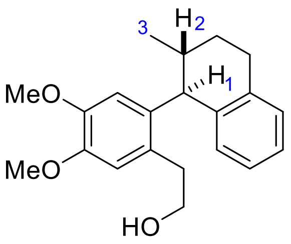

2au

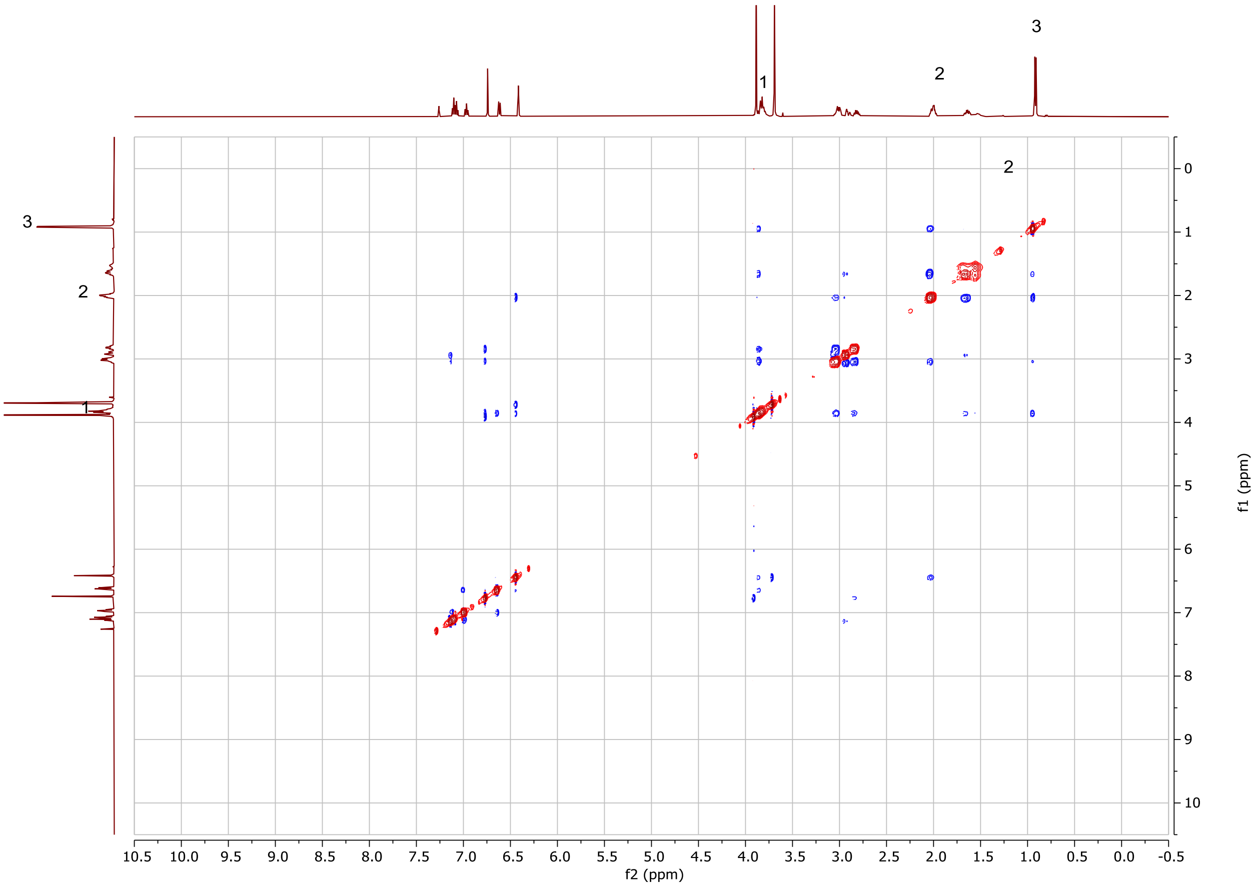

Analysis: Protons 1 and 2 do not correlate. On the contrary, protons 3 and 1 correlate strongly. Put together, these observations show a *trans* relative configuration.

6. XRD

X-ray crystallography (jmcm220131, jmcm220204, jmcm220530, jmcm220531)

The crystals were placed in oil, and a single crystal was selected, mounted on a glass fibre and placed in a low-temperature N_2 stream.

For compound **2aj**, X-Ray diffraction data collection was carried out on a Bruker PHOTON-III DUO CPAD diffractometer equipped with an Oxford Cryosystem liquid N_2 device, using Cu-Kα radiation (λ = 1.54178 Å). The crystal-detector distance was 40 mm. The cell parameters were determined (APEX4 software) [1] from reflections taken from one set of 180 frames, each at 1s exposure. The structure was solved using the program SHELXT-2014 [2]. The refinement and all further calculations were carried out using SHELXL-2014 [3]. The H-atoms were included in calculated positions and treated as riding atoms using SHELXL default parameters. The non-H atoms were refined anisotropically, using weighted full-matrix least-squares on F^2. A semi-empirical absorption correction was applied using SADABS in APEX4 [1]; transmission factors: T_{min}/T_{max} = 0.6091/0.7528.

For compounds **2a, 2aj, 2ao**, X-Ray diffraction data collection was carried out on a Bruker PHOTON-III DUO CPAD diffractometer equipped with an Oxford Cryosystem liquid N_2 device, using Mo-Kα radiation (λ = 0.71073 Å). The crystal-detector distance was 37 mm. The cell parameters were determined (APEX4 software) [1] from reflections taken from one set of 180 frames, each at 1s exposure. The structures were solved using the program SHELXT-2014 [2]. The refinement and all further calculations were carried out using SHELXL-2014 [3]. The H-atoms were included in calculated positions and treated as riding atoms using SHELXL default parameters. The non-H atoms were refined anisotropically, using weighted full-matrix least-squares on F^2. A semi-empirical absorption correction was applied using SADABS in APEX4 [1]; transmission factors: T_{min}/T_{max} = 0.7128/0.7463; T_{min}/T_{max} = 0.7130/0.7456; T_{min}/T_{max} = 0.7261/0.7456, respectively for jmcm220204, jmcm220530, jmcm220531.

[1] "M86-EXX278V1 APEX4 User Manual", Bruker Corporation, 2021.

[2] G. M. Sheldrick, *Acta Cryst.* **2015**, A71, 3-8.

[3] G. M. Sheldrick, *Acta Cryst.* **2015**, C71, 3-8.

Table S4. Crystal data and structure refinement.

Compound	**2a**	**2c**	**2aj**	**2ao**
Empirical Formula	$C_{16}H_{15}NO_3$	$C_{20}H_{18}F_6O_3$	$C_{22}H_{14}F_5NO_3$	$C_{19}H_{19}NO_3$
M_r	269.29	420.34	435.34	309.35
Crystal size, mm^3	0.40 x 0.28 x 0.16	0.12 x 0.08 x 0.04	0.18 x 0.14 x 0.06	0.18 x 0.16 x 0.12
Crystal system	Monoclinic	Monoclinic	Monoclinic	Triclinic
Space group	P c	P 21/n	P 21/c	P -1
a, Å	7.9798(4)	8.6103(7)	41.4191(17)	7.9718(3)
b, Å	10.8805(5)	7.9713(5)	6.5104(3)	9.2217(3)
c, Å	8.4210(4)	27.483(2)	14.0699(6)	11.1636(4)
α, °	90	90	90	100.8750(10)
β, °	117.053(2)	91.244(3)	98.700(3)	90.0200(10)
γ, °	90	90	90	110.5260(10)
Cell volume, Å^3	651.15(5)	1885.9(2)	3750.4(3)	752.80(5)
Z ; Z'	2, 1.373 Mg/m^3	4, 1.480 Mg/m^3	8, 1.542 Mg/m^3	2, 1.365 Mg/m^3
T, K	120(2)	120(2)	120(2)	120(2)
Radiation type ; wavelength Å	Mo-Kα λ = 0.71073 Å	Mo-Kα λ = 0.71073 Å	Cu-Kα λ = 1.54178 Å	Mo-Kα λ = 0.71073 Å
F_{000}	284	864	1776	328
μ, mm^{-1}	0.095	0.137	1.186	0.092
θ range, °	2.866 – 32.024	2.464 – 28.011	2.158 – 66.527	1.862 – 28.044
Reflection collected	21584	78056	72332	39094
Reflections unique	4510	4574	6631	3652
R_{int}	0.0231	0.0421	0.0940	0.0240
GOF	1.063	1.055	1.032	1.066
Refl. obs. (I>2σ(I))	4510	4574	6631	3652
Parameters	181	264	560	208
wR_2 (all data)	0.0819	0.1214	0.2430	0.1088
R value (I>2σ(I))	0.0306	0.0441	0.0826	0.0387
Largest diff. peak and hole (e-.Å^{-3})	0.291; -0.176	0.541; -0.459	0.817; -0.429	0.346; -0.231
